# Photoinduced Site-Selective Aryl C-H Borylation with Electron-Donor-Acceptor Complex Derived from B_2_Pin_2_ and Isoquinoline

**DOI:** 10.3390/molecules29081783

**Published:** 2024-04-14

**Authors:** Manhong Li, Yi-Hui Deng, Qianqian Chang, Jinyuan Li, Chao Wang, Leifeng Wang, Tian-Yu Sun

**Affiliations:** 1 Key Lab of Computational Chemistry and Drug Design, State Key Laboratory of Chemical Oncogenomics, School of Chemical Biology and Biotechnology, Peking University Shenzhen Graduate School, Shenzhen 518055, China; e1328001@u.nus.edu (M.L.); dyh@stu.pku.edu.cn (Y.-H.D.); wangchao@pku.edu.cn (C.W.); 2 School of Pharmaceutical Sciences (Shenzhen), Sun Yat-sen University, No. 66, Gongchang Road, Shenzhen 518107, China; changqq3@mail2.sysu.edu.cn; 3 Institute of Molecular Chemical Biology, Shenzhen Bay Laboratory, Shenzhen 518132, China; 4 Department of Pharmacy and Pharmaceutical Sciences, Faculty of Science, National University of Singapore, Block S4A, Level 3, 18 Science Drive 4, Singapore 117543, Singapore; 5 Shanghai Institute of Organic Chemistry, University of Chinese Academy of Sciences, Chinese Academy of Sciences, 345 Lingling Lu, Shanghai 200032, China; lijinyuan@mail.sioc.ac.cn

**Keywords:** photocatalysis, C-H bond, borylation, electron-donor-acceptor, B_2_Pin_2_, isoquinoline

## Abstract

Due to boron’s metalloid properties, aromatic boron reagents are prevalent synthetic intermediates. The direct borylation of aryl C-H bonds for producing aromatic boron compounds offers an appealing, one-step solution. Despite significant advances in this field, achieving regioselective aryl C-H bond borylation using simple and readily available starting materials still remains a challenge. In this work, we attempted to enhance the reactivity of the electron-donor-acceptor (EDA) complex by selecting different bases to replace the organic base (NEt_3_) used in our previous research. To our delight, when using NH_4_HCO_3_ as the base, we have achieved a mild visible-light-mediated aromatic C-H bond borylation reaction with exceptional regioselectivity (rr > 40:1 to single isomers). Compared with our previous borylation methodologies, this protocol provides a more efficient and broader scope for aryl C-H bond borylation through the use of N-Bromosuccinimide. The protocol’s good functional-group tolerance and excellent regioselectivity enable the functionalization of a variety of biologically relevant compounds and novel cascade transformations. Mechanistic experiments and theoretical calculations conducted in this study have indicated that, for certain arenes, the aryl C-H bond borylation might proceed through a new reaction mechanism, which involves the formation of a novel transient EDA complex.

## 1. Introduction

Aromatic boron reagents are widely used as synthetic intermediates in the production of pharmaceuticals and natural products through Suzuki–Miyaura/Chan-Lam cross-coupling [1,2,3,4,5], oxidation [6], halogenation [7,8] and homologation [9] benefiting from the unique metalloid properties of boron [10,11,12]. In principle, the direct borylation of aryl C-H bonds for producing aromatic boron compounds offers an appealing, one-step solution compared to traditional strategies [13,14,15,16]; however, this approach has proven to be quite challenging.

**Scheme 1:**
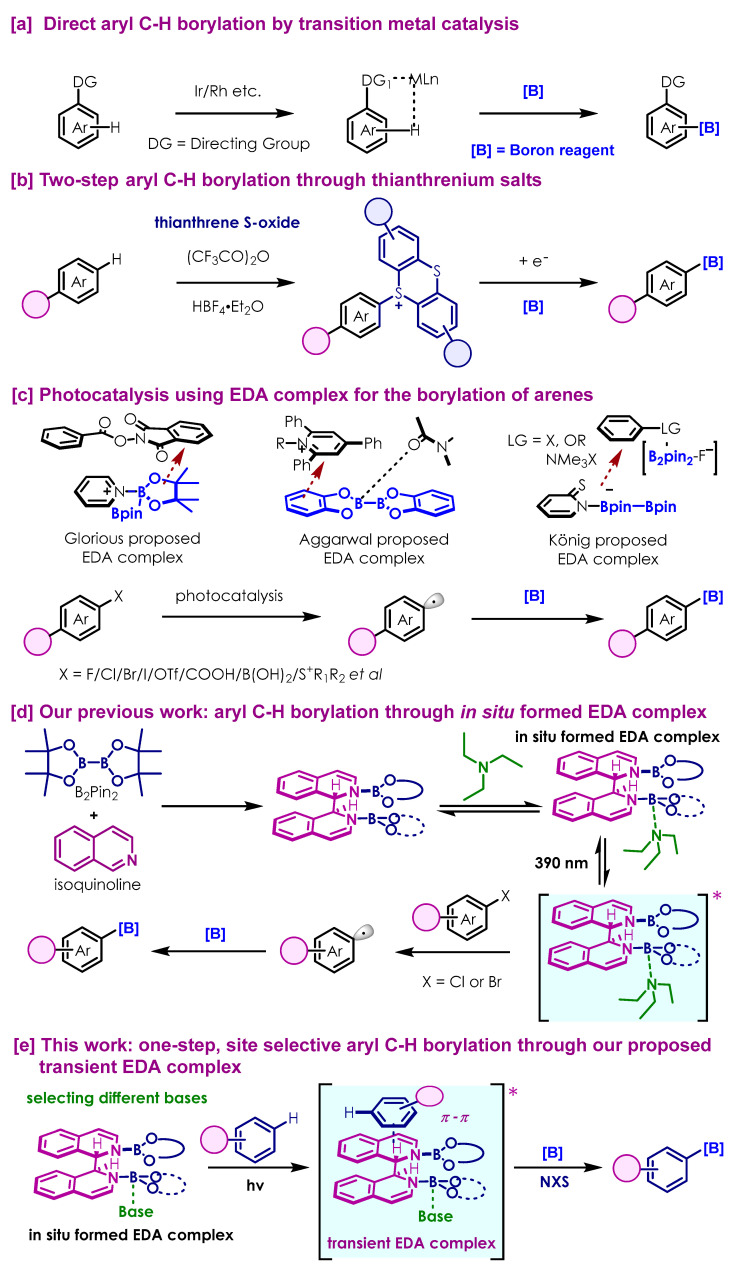
Synthetic approaches to aryl boronic esters. (The symbol * denotes excited state).

Transition metal-promoted directed C-H bond activation, especially using Ir/Rh, has emerged as a powerful tool for the construction of C-B bonds [17,18,19] (Scheme 1a), commonly relying on precious metal catalysts and appropriate directing groups (DG). Moreover, the presence of multiple functional groups in complex bioactive molecules often poses a challenge for their late-stage borylation, particularly under harsh reaction conditions.

Ritter developed a series of elegant late-stage C-H bond functionalization protocols [20,21,22] through site-selective thianthrenation, utilizing thianthrene S-oxide as a versatile pre-functionalization reagent. Triggered by a single electron transfer (SET) progress, the C-S bond of aryl thianthrenium salts can be cleaved to form thianthrene and aryl radical [23,24], which is then spontaneously captured by the diboron reagent (Scheme 1b) [25]. Despite these pioneering advancements, achieving regioselective aryl C-H bond borylation using simple and readily available starting materials still remains a challenge.

In recent years, photocatalysis [26,27,28,29,30,31] using the electron-donor-acceptor (EDA) complex [32,33,34,35] has gained popularity, offering new methods for radical-mediated transformations. Due to boron’s metalloid properties [10,11,12], there is increasing interest in using boron reagents as alternatives to prevalent transition metals for photocatalysis. The notably high reactivity of diboron reagents makes them excellent precursors for constructing the EDA complex, as they can effectively combine with electron donor partners, such as amines and N-containing heterocycles (NCHs) (Scheme 1c). Glorius [36] disclosed a photoinduced decarboxylative borylation of aryl N-hydroxyphthalimide esters via the use of Katritzky salts as acceptor in the EDA complex. Aggarwal [37] proposed an EDA complex formed by 2-iodophrnyl thiocarbonate, bis(pinacolato)diboron (B_2_Pin_2_) and *N*,*N*-dimethylformamide, resulting in the formation of boronic esters with high stereocontrol. König [38] achieved an ipso-borylation of substituted arenes by utilizing an EDA complex formed between thiolate, B_2_Pin_2_ and boryl anion activated substrates.

Our group has recently described several strategies for generating aryl radicals under visible-light catalysis [39,40,41], and our previous studies have demonstrated that the reductive coupling of isoquinoline and B_2_Pin_2_ can produce a dimer. This dimer can coordinate with an organic base (NEt_3_) in situ to form an EDA complex (Scheme 1d). Under visible-light irradiation, the excited state of the in situ-formed EDA complex is a highly active reductant and can facilitate SET with aryl halides to produce aryl radical intermediates [40].

Inspired by our earlier work and other elegant conceptions [42,43,44,45], we wonder whether selecting different bases could facilitate the photoinduced formation of a transient EDA complex between the in situ-formed EDA complex and certain arenes through π–π interaction. This transient EDA complex could then undergo direct C-H borylation upon treatment with regioselective oxidative additives (e.g., N-Halosuccinimides/NXS: X = Cl, Br, I) [46,47] (Scheme 1e).

Our mechanistic experiments and theoretical calculations in this study have indicated that, when using NH_4_HCO_3_ as the base, certain arenes may indeed yield borylation products through the transient EDA complex formation pathway. Furthermore, the yield is significantly increased compared to the organic base (NEt_3_) used in our previous work [40]. This research holds the potential to enhance the highly efficient, site-selective, direct C-H borylation of more challenging arenes.

## 2. Results

### 2.1. Optimization of Reaction Conditions for the Aryl C-H Bond Borylation

We optimized the reaction conditions for the borylation of methyl 4-amino-2-methoxybenzoate using a mixture of B_2_Pin_2_, isoquinoline (a N-containing heterocycle, NCH) and a base under visible-light irradiation (Table 1). Extensive screening of the reaction conditions revealed that treating methyl 4-amino-2-methoxybenzoate with NBS (0.22 mmol), isoquinoline (20 mol%), B_2_Pin_2_ (0.8 mmol), and NH_4_HCO_3_ in acetonitrile resulted in the borylated product with a 72% NMR yield (68% isolated yield, entry **1**). The choice of the base had a great influence on the efficiency of borylation (entries 2–5). Organic bases such as *N*,*N*-Diisopropylethylamine (DIEPA) and triethylamine (NEt_3_) did not perform well under standard condition compared to NH_4_HCO_3_ (see Supporting Information Table S1). These results indicate that using NH_4_HCO_3_ as a base provides a clear advantage over our previous work using organic base (NEt_3_) [40]. A total of 2.0 eq NBS led to reduced quantities of the desired product (54%; entry 6). Not surprisingly, no desired product was observed without the addition of NBS (entry 7). Finally, the photochemical nature of this borylation strategy was confirmed, as the reaction did not occur either in the absence of light (entry 8) or in the absence of the EDA complex precursor (entry 9).

### 2.2. Substrate Scope of the Photoinduced Aryl C-H Borylation

**Figure 1:**
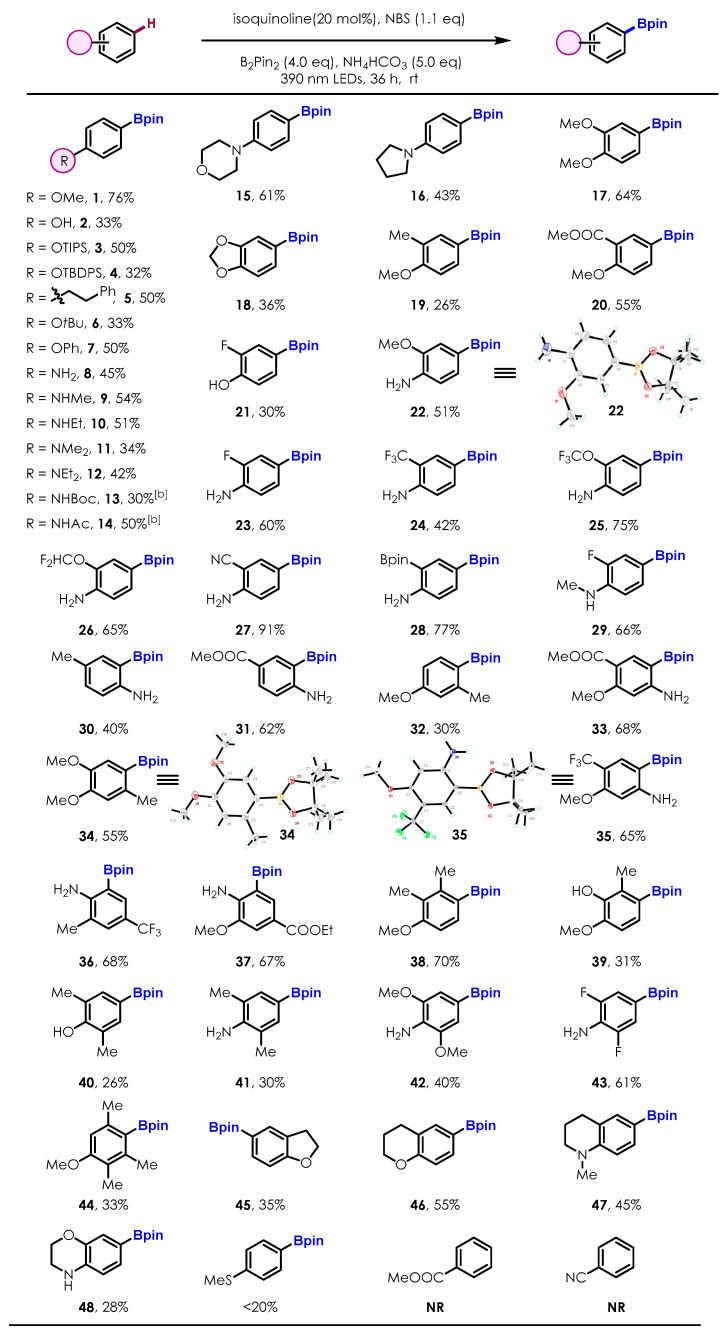
Substrate scope of arenes. Reactions were run on 0.2 mmol scales, isolated yield. ^[b]^ 0.2 mmol isoquinoline, 72 h.

Having demonstrated the validity of our proposal, we turned our attention to the substrate scope of arenes utilizing NH_4_HCO_3_ as the additive. A variety of aromatic substrates were subjected to the C-H borylation procedure using B_2_Pin_2_ as the aryl radical trap in the photochemical progress (Figure 1).

Phenol, aniline, and their electron donating/withdrawing group-protected derivatives (**1**–**14**) were smoothly converted to the corresponding arylboronates in moderate-to-good yields (30–76%), with excellent selectivity for the para-selective products (rr > 40:1 for single isomers). Heterocycles such as morpholine (**15**) and pyrrolidine (**16**) were also competent substrates.

Electron-rich and electron-deficient group di-substituted arenes, such as 1,2-, 1,3-, and 1,4-substituted arenes can also undergo the C-H borylation reaction in moderate-to-good yields (**17**–**32**), showcasing the compatibility of our method with a broad array of functionalities (e.g., difluoromethoxyl-, trifluoromethyl-, trifluoromethoxy-, cyano-, methoxycarbonyl-, cyclohexyl- and naked hydroxyl/amino). A dual borylation product (**28**) was successfully synthesized using boronic ester aniline. Medically relevant fluorine-containing anilines led to C-H borylation products with high efficiencies (**21**, **23**–**26**, **29**).

Of particular note is the capability of this method to regioselectively and efficiently construct the C-B bond from the hindered aryl C-H bond, as shown by the reaction of substrates **33**–**44**, yielding the desired borylation products in good yield and with excellent chemoselectivities.

Remarkably, the borylation reaction of bicyclic aromatic substrates, such as 2,3-dihydrobenzofuran (**45**), chromane (**46**), and 1,2,3,4-tetrahydroisoquinoline derivative (**47**), all proceeded smoothly and afforded the corresponding borylation products as single regio-isomers in accordance with the high reactivity of these positions for electrophilic substitution.

However, thioanisole and strong electron-withdrawing-group substituted arenes were not tolerated. Methyl benzoate and benzonitrile did not deliver the desired products under standard reaction conditions.

The regioselectivity of the C-H borylation reaction is governed by both the electronic and steric effects of the substituents, similar to the nucleophilic substitution reactions induced by triptycenyl sulfide, as reported by Miura and co-workers [46].

### 2.3. Late-Stage Functionalization and Novel Cascade Transformation

**Figure 2:**
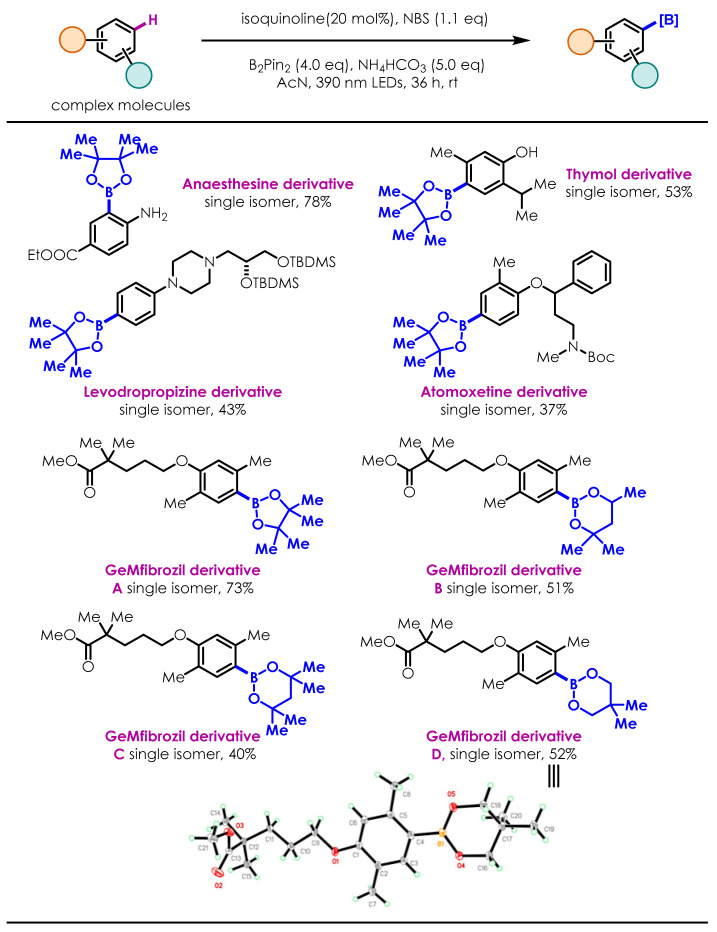
Late-stage functionalization of complex molecules. Reactions were run on 0.2 mmol scales, isolated yield.

To assess the applicability of this aryl C-H borylation strategy for late-stage functionalization, a variety of representative active pharmaceutical ingredients (APIs) and natural products, including anaesthesine, gemfibrozil, levodropropizine, atomoxetine, and natural phenol thymol, were subjected to this protocol. These compounds were found to be tolerant in this protocol, yielding the corresponding borylated derivatives in good yields in a single-isomer manner (Figure 2). These transformations accommodated various functional groups, including sensitive esters, amides, and N-heterocycles, highlighting the mildness and practicality of this protocol. Notably, this methodology also worked well with a series of diboron reagents to produce gemfibrozil derivatives A–D with useful efficiencies (e.g., **D**, 52% yield).

**Figure 3:**
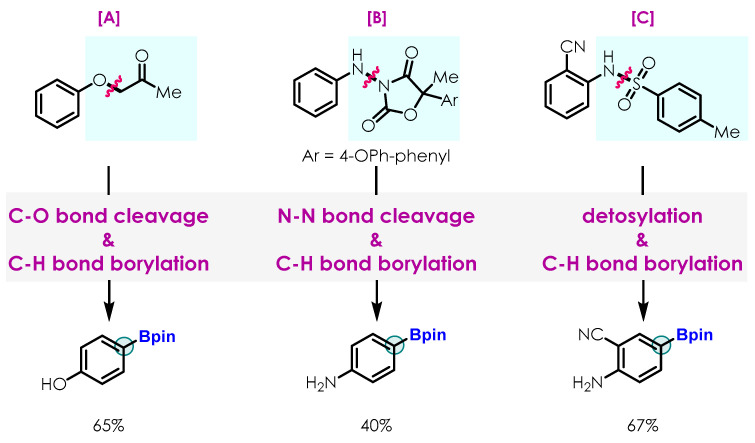
Novel cascade transformation. Reactions were performed with 0.2 mmol substrate, 1.0 mmol NH_4_HCO_3_, 20 mol% N-containing heterocycle (NCH), 0.8 mmol B_2_Pin_2_, 0.22 mmol NBS in 0.5 mL acetonitrile under 390 nm LEDs irradiation for 36 h, isolated yield. (**A**) Reductive C-O bond cleavage and C-H bond borylation. (**B**) Reductive N-N bond cleavage and C-H bond borylation. (**C**) Reductive N-S bond cleavage and C-H bond borylation.

To further demonstrate the utility of this novel borylation protocol (Figure 3), more challenging cascade transformations were studied. Reductive cleavages of unusual N-N/C-O bonds and detosylation followed by direct C-H bond borylation under standard conditions afforded the borylated aniline and phenol derivatives with good efficiencies. Each of these transformations is highly important, as site-selective cascade transformation of the aromatic C-H bond remains a major challenge.

## 3. Discussion

**Scheme 2:**
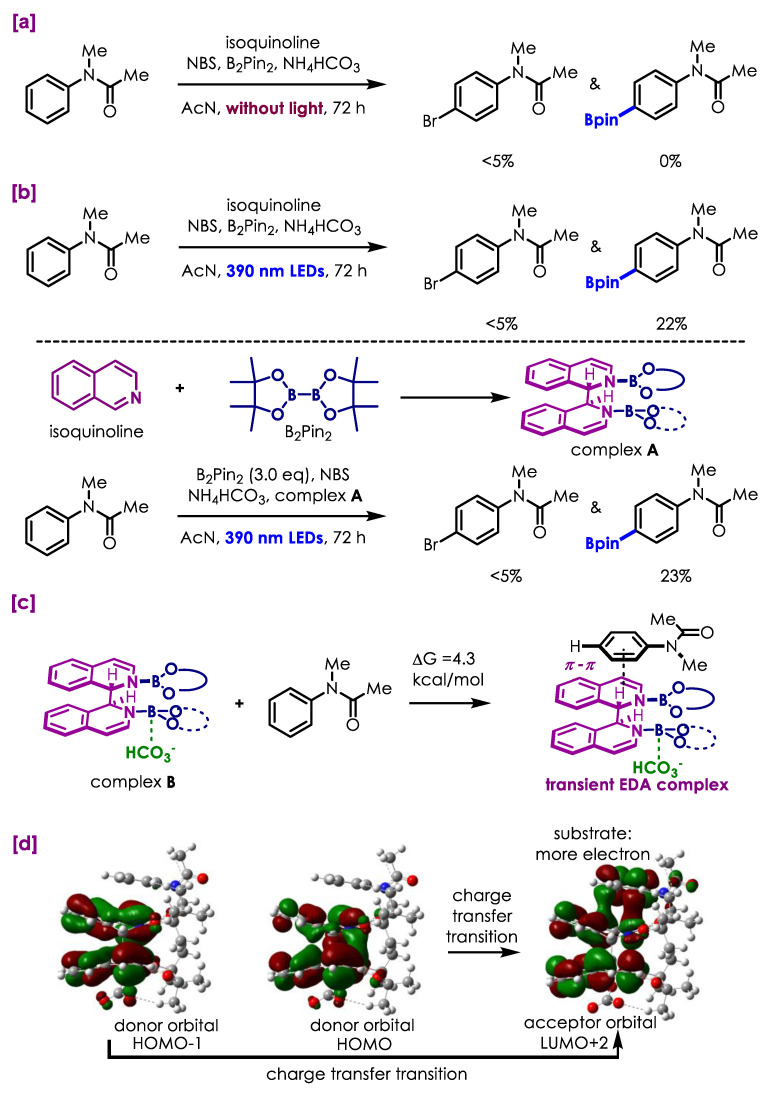
Mechanistic and computational experiments: (**a**) mechanistic experiments using isoquinoline (0.04 mmol)/4-N-methylacetanilide (0.2 mmol)/NH_4_HCO_3_ (1.0 mmol)/B_2_Pin_2_ (0.8 mmol)/NBS (0.22 mmol) without 390 nm LEDs irradiation for 72 h; (**b**) mechanistic experiments using complex **A** (0.2 mmol)/4-N-methylacetanilide (0.2 mmol)/NH_4_HCO_3_ (1.0 mmol)/B_2_Pin_2_ (0.6 mmol)/NBS (0.22 mmol) with 2 × 390 nm LEDs irradiation for 72 h; (**c**) the binding Gibbs free energy between complex **B** and the arene substrate to form transient EDA complex; (**d**) TDDFT-computed donor and acceptor orbitals of charge transfer transitions in the transient EDA complex.

To gain more insight into the mechanism for this C-H bond borylation reaction, mechanistic and computational experiments were performed (Scheme 2). Scheme 2a shows that the N-methylacetanilide substrate cannot directly undergo the bromination reaction with NBS to afford N-(4-Bromophenyl)-N-methylacetamide without photoirradiation, and the borylation also cannot occur. However, the borylation of the N-methylacetanilide can occur under the standard condition, as well as under the condition where isoquinoline is replaced by complex **A** (complex **A** is formed by isoquinoline and B_2_Pin_2_) (Scheme 2b). Based on these observations, we infer that the arene substrate may interact with complex **B** (complex **A** + base) to form a new transient EDA complex, thereby being excited by visible light to increase the electron density of the arene, and then react with NBS and the diboron reagent to produce the borylation product. Density functional theory (DFT) calculations [48] show that the Gibbs free energy is uphill by 4.3 kcal/mol when complex **B** interacts with arene substrate via π–π stacking, indicating that the existence of the transient EDA complex is feasible (see Scheme 2c). Furthermore, the time-dependent DFT (TD-DFT) method was used to study the charge-transfer-type transitions of the transient EDA complex. The involved molecular orbitals (HOMO-1, HOMO and LUMO + 2) are depicted in Scheme 2d. Two significant excitations (HOMO-1 to LUMO + 2, and HOMO to LUMO + 2) can be assigned to the corresponding charge transfer from the complex **B** to the substrate, and the arene substrate with more electron density will facilitate the subsequent transformations.

**Scheme 3:**
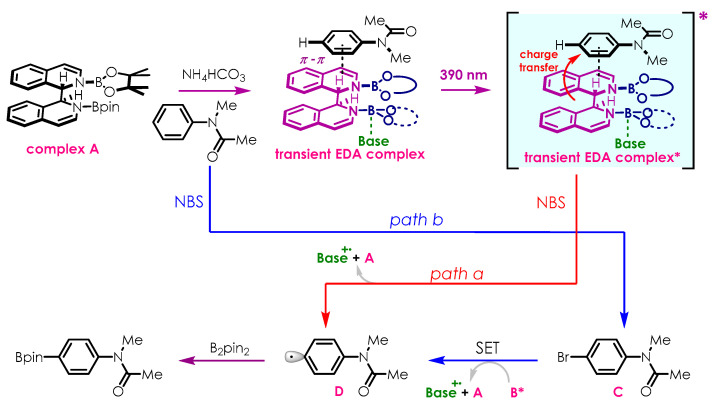
The plausible mechanism. (The symbol * denotes excited state).

With these mechanistic insights, as well as the related literature, a plausible mechanism is proposed in Scheme 3. Complex **A**, generated through the reductive coupling of isoquinoline and B_2_Pin_2_, coordinates with the substrate and base to afford a transient EDA complex, which is then excited by 390 nm light, yielding a highly reactive excited state. Oxidation by NBS may afford N-(4-Bromophenyl)-N-methylacetamide **C** (path b in Scheme 3) or directly afford aryl radical **D** (path a in Scheme 3) and regenerate complex **A**. Subsequently, aryl radical **D** could be trapped by the diboron reagent, furnishing the desired borylation products.

Compared with our previous reports of borylation methodologies, this concise strategy provides more efficient and a broader scope for aryl C-H bond borylation, achieved directly from simple arenes rather than the two-step one-pot synthesis from electron-rich arenes, presenting a new reaction mechanism.

## 4. Methods and Materials

### 4.1. Photo Reaction Setup

Irradiation of the photochemical reaction was carried out using Kessil PR160L-390 nm ultraviolet lamps from both sides at 1–2 cm, with an average intensity of 159 mW/cm^2^ (measured from 6 cm distance). Reactions were cooled using a 20 W clamp fan placed on the top of the reactor. Stirring was achieved by placing the assembled reactor on IKA C-MAG HS 7 control magnetic stir bars. All reactions were performed in 2 mL vial and were run under air.

### 4.2. Thin-Layer Chromatography (TLC)

Analytical TLC was performed on silica gel GF254 plates. The TLC plates were visualized by ultraviolet light (λ = 254 nm). Organic solutions were concentrated using a rotary evaporator with a diaphragm vacuum pump purchased from EYELA and Heidolph. Fresh silica-gel chromatography was performed using 300–400 mesh silica gel (Qingdao, China).

### 4.3. Nuclear Magnetic Resonance Spectroscopy (NMR)

Proton and carbon magnetic resonance spectra (^1^H NMR, ^13^C NMR, ^11^B NMR and ^19^F NMR) were recorded on a Bruker AVANCE III (^1^H NMR at 400 MHz or 600 MHz, ^13^C NMR at 101 MHz or 151 MHz, ^11^B NMR at 128 MHz or 193 MHz, ^19^F NMR at 377 MHz) spectrometer, with solvent resonance as the internal standard (^1^H NMR: CDCl_3_ at 7.26 ppm; ^13^C NMR: CDCl_3_ at 77.16 ppm). NMR yield using pyrazine or hexamethyldisiloxane (HMDSO) was used as the internal standard.

### 4.4. Materials

Commercially available reagents were purchased from Sigma-Aldrich, Adamas-beta, TCI, and Bidepharm were used as received unless otherwise noted. Super Dry solvents such as acetonitrile (AcN), dimethylformamide (DMF), tetrahydrofuran (THF), dichloromethane (DCM), 1,2-dichloroethane (DCE) and dimethyl sulfoxide (DMSO) were purchased from Adamas-beta. Other common solvents such as petroleum ether (PE) and ethyl acetate (EtOAc) were rectification grade for flash chromatography on silica gel, purchased from General-reagent.

## 5. Conclusions

In summary, we have developed a novel diboron-type EDA-complex catalyzed C-H borylation of arenes, which occurs under ambient temperature and mild conditions. This operationally simple protocol exhibits exceptional regioselectivity and tolerates a wide range of functional groups, allowing convenient modification of complex natural products and APIs. Furthermore, the key role of the transient EDA complex formed in situ was confirmed through control experiments and computations, and a plausible mechanism was proposed. Perhaps even more useful, this method offers a versatile strategy to achieve novel one-pot multi-transformations, such as challenging reductive C-O/N-N/N-S bond cleavages followed by cascade aromatic C-H bond borylation.

## Figures and Tables

**Table 1:** Optimization of the reaction conditions ^[a]^.

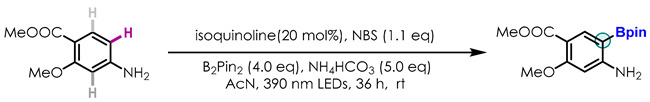		
**Entry**	**Deviations from Standard Condition**	**Yield (%) ^[b]^**
1	none	72(68) ^[c]^
2	NEt_3_ instead of NH_4_HCO_3_	31
3	KOH instead of NH_4_HCO_3_	trace
4	Cs_2_CO_3_ instead of NH_4_HCO_3_	54
5	NaHCO_3_ instead of NH_4_HCO_3_	19
6	2.0 eq NBS instead of 1.1 eq NBS	54
7	NO NBS instead of 1.1 eq NBS	0
8	No light	0
9	No isoquinoline	0

^[a]^ Reactions were performed with 0.2 mmol methyl 4-amino-2-methoxybenzoate, 1.0 mmol base, 20 mol% N-containing heterocycle (NCH), 0.8 mmol B_2_Pin_2_, and 0.22 mmol NBS in 0.5 mL acetonitrile under 390 nm LEDs irradiation for 36 h. ^[b]^ NMR yields using pyrazine or hexamethyl disiloxane as internal standard. ^[c]^ Isolated yields.

## Data Availability

The data are contained in the article and Supplementary Materials.

## Supplementary Materials

The following supporting information can be downloaded at: https://www.mdpi.com/article/10.3390/molecules29081783/s1. Scheme S1: Photo reaction setup; Table S1: Optimization of reaction conditions; Scheme S2: Substrates with low yield under optimized conditions; Scheme S3: Novel cascade transformation; Table S2: Oxidative additives screen and control experiments; Table S3: Reaction with complex A; Figures S1 and S2: The computational results; Tables S4–S7: Crystal data and structure refinement. References [49,50,51,52,53,54,55,56,57,58,59,60,61,62,63,64,65,66,67,68,69,70,71,72,73,74,75,76,77,78,79,80,81,82,83,84,85] are cited in Supplementary Materials.
