# Supplementary material for: Photoinduced Site-Selective Aryl C-H Borylation with Electron-Donor-Acceptor Complex Derived from B2Pin2 and Isoquinoline
Source: Molecules. 2024 Apr 14;29(8):1783. doi: 10.3390/molecules29081783 (PMC11052414; doi:10.3390/molecules29081783)
Supplement: Supplementary file 1 [file molecules-29-01783-s001.zip › molecules-2959216-supplementary.pdf]

Photoinduced site-selective aryl C-H borylation with Electron-Donor-Acceptor complex derived from B2Pin2 and isoquinoline

Manhong Li,†[1,2,3,4] Yi-Hui Deng,†[1] Qianqian Chang,†[2] Jinyuan Li,[5] Chao Wang,[1] Leifeng Wang,*[2] Tian-Yu Sun,*[1,3]

1 Key Lab of Computational Chemistry and Drug Design, State Key Laboratory of Chemical Oncogenomics, School of Chemical Biology and Biotechnology, Peking University Shenzhen Graduate School, Shenzhen 518055, P. R. China; e1328001@u.nus.edu; dyh@stu.pku.edu.cn; wangchao@pku.edu.cn

2 School of Pharmaceutical Sciences (Shenzhen), Sun Yat-Sen University, No.66, Gongchang Road, Shenzhen 518107, P. R. China; Changqq3@mail2.sysu.edu.cn.

3 Institute of Molecular Chemical Biology, Shenzhen Bay Laboratory, Shenzhen 518132, P. R. China

4 Department of Pharmacy and Pharmaceutical Sciences, Faculty of Science, National University of Singapore, Block S4A, Level 3, 18 Science Drive 4, Singapore 117543, Singapore

5 Shanghai Inst Organ Chem, Univ Chinese Acad Sci, Chinese Acad Sci, Shanghai 200032, 345 Lingling Lu, P. R. of China; lijinyuan@mail.sioc.ac.cn.

† These authors contributed equally to this work.

* Correspondence: wanglf33@mail.sysu.edu.cn; Tian-Yu_Sun@pku.edu.cn

Table of Contents

1.) General Information **S2**

2.) Optimization Studies **S3**

3.) Substrate Scope of Arenes and Characterization Data **S4**

4.) Substrate with Low Yield under Optimized Conditions **S25**

5.) Novel Cascade Transformation of Arenes **S26**

6.) Mechanistic Study **S26**

6.1 Preparation of Complex A S26

6.2 Oxidant Screen and Control Experiments S26

6.3 Computational Experiment S28

7.) X-ray Crystallographic Data **S30**

8.) References **S36**

9.) NMR Spectroscopic Data **S38**

1.) General Information

Methods and Materials: Irradiation of the photochemical reaction was carried out using Kessil PR160L-390 nm ultraviolet lamps from both sides at 1–2 cm, with an average intensity of 159mW/cm^2 (measured from 6 cm distance). Reactions were cooled using a 20W clamp fan placed on the top of the reactor. Stirring was achieved by placing the assembled reactor on IKA C-MAG HS 7 control magnetic stir bars. All reactions were performed in 2mL vials and were run under air (see the picture below for the reaction setup).

Analytical TLC was performed on silica gel GF254 plates. The TLC plates were visualized by ultraviolet light (λ = 254 nm). Organic solutions were concentrated using a rotary evaporator with a diaphragm vacuum pump purchased from EYELA and Heidolph. Frash silica-gel chromatography was performed using 300–400 mesh silica gel (Qingdao, China).

Proton and carbon magnetic-resonance spectra (^{1}H NMR, ^{13}C NMR, ^{11}B NMR and ^{19}F NMR) were recorded on a Bruker AVANCE III (^{1}H NMR at 400 MHz or 600 MHz, ^{13}C NMR at 101 MHz or 151 MHz, ^{11}B NMR at 128 MHz or 193MHz, ^{19}F NMR at 377 MHz) spectrometer with solvent resonance as the internal standard (^{1}H NMR: $CDCl_3$ at 7.26 ppm; ^{13}C NMR: $CDCl_3$ at 77.16 ppm). NMR yield using pyrazine or hexamethyldisiloxane (HMDSO) was used as the internal standard. ^{1}H NMR data are reported as follows: chemical shift, multiplicity (s = singlet, d = doublet, t = triplet, dd = doublet of doublets, ddd = doublet of doublets of doublets, ddddd = doublet of doublets of doublets of doublets of doublets, dt = doublet of triplets, ddt = doublet of doublets of triplets, td = triplet of doublets, tt = triplet of triplets, m = multiplet, q = quartet), coupling constants (Hz), and integration.

Commercially available reagents were purchased from Sigma-Aldrich, Adamas-beta, TCI, and Bidepharm and were used as received unless otherwise noted. Super Dry solvents such as acetonitrile (AcN), dimethylformamide (DMF), tetrahydrofuran (THF), dichloromethane (DCM), 1,2-dichloroethane (DCE) and dimethyl sulfoxide (DMSO) were purchased from Adamas-beta. Other common solvents such as petroleum ether (PE) and ethyl acetate (EtOAc) were rectification grade for flash chromatography on silica gel purchased from General-reagent.

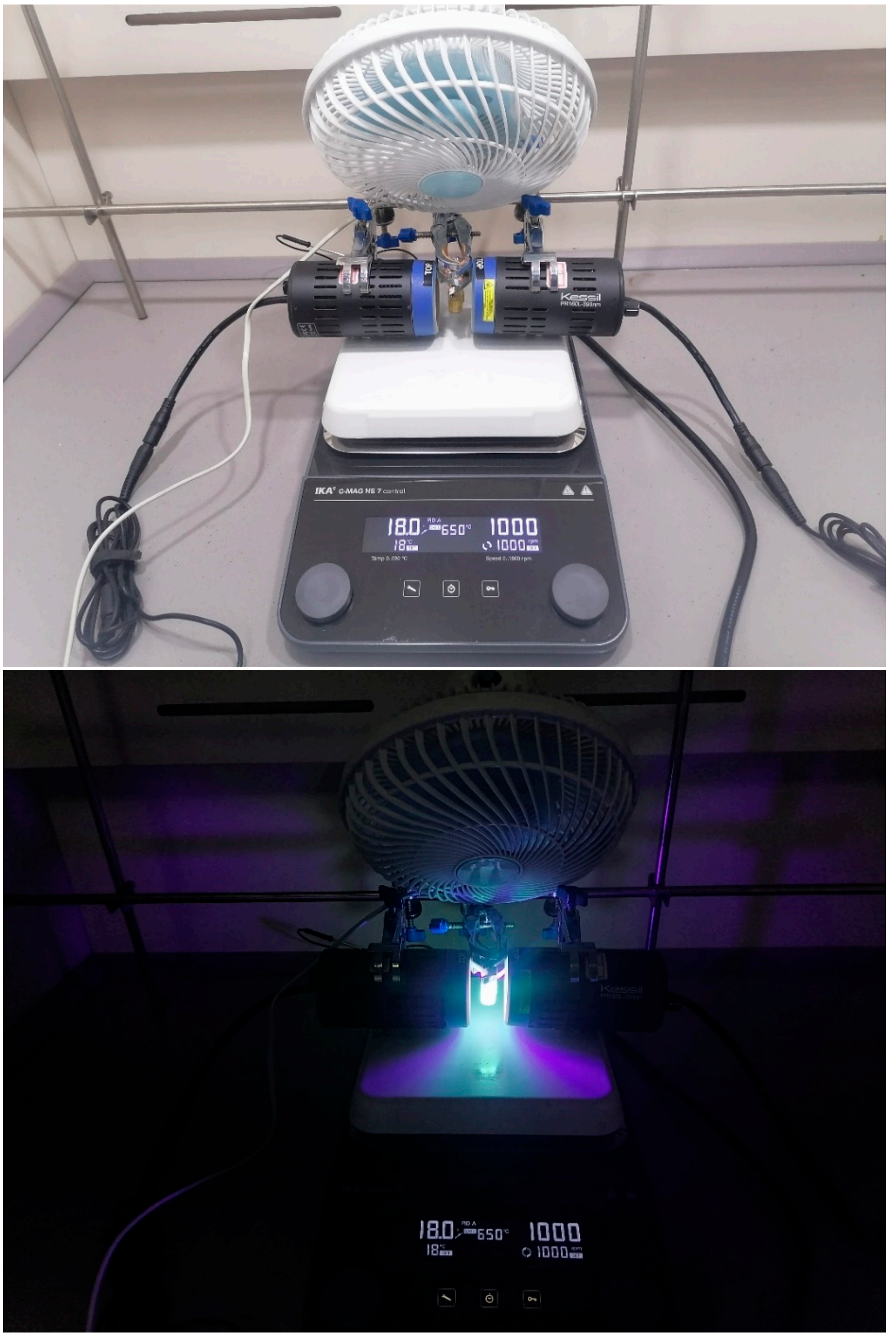

Scheme S1 Photo reaction setup

2.) Optimization Studies

General procedure (A):

An oven-dried 2 mL vial was charged with a magnetic stir bar, and to the solvent of the 3-Methoxy-4-(methoxycarbonyl) aniline (36.2 mg, 0.2 mmol, 1.0 eq) in AcN (0.5 mL), suitable NBS, B_2pin_2 and base, isoquinoline (5.2 mg, 0.04 mmol, 20 mol%) were added. The vial was sealed with a plastic cap and then irradiated with a 2*390 nm LEDs light for a period of time. The inorganic base was removed by filtration and the solvent was removed by vacuum evaporation. NMR yield was determined by ^1H NMR using pyrazine or HMDSO as the internal standard. The reaction mixture was purified by flash chromatography on silica gel to afford the desired borylated product.

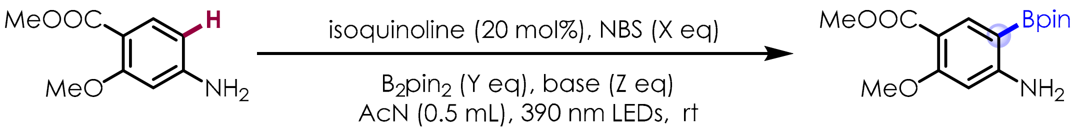

entry	NBS(X eq)	B_2pin_2 (Y eq)	base (Z eq)	time (h)	yield (%)[a]
1	NBS (1.1)	2.0	NH_4HCO_3(3.0)	12h	51
2	NBS (1.1)	2.0	NH_4HCO_3(5.0)	12h	53
3	NBS (1.1)	4.0	NH_4HCO_3(5.0)	12h	58
**4**	**NBS (1.1)**	**4.0**	**NH_4HCO_3(5.0)**	**36h**	**72(68%)[b]**
5	NBS (2.0)	4.0	NH_4HCO_3(5.0)	36h	54
6	NBS (0)	4.0	NH_4HCO_3(5.0)	36h	0
7	NBS (1.1)	4.0	NH_4HCO_3(5.0)	36h	0[c]
8	NBS (1.1)	4.0	NH_4HCO_3(5.0)	36h	0[d]
9	NBS (1.0)	4.0	NH_4HCO_3(5.0)	36h	65
10	NBS (1.1)	4.0	NEt_3(5.0)	36h	31
11	NBS (1.1)	4.0	DIPEA(5.0)	36h	26
12	NBS (1.1)	4.0	KOH(5.0)	36h	trace
13	NBS (1.1)	4.0	Cs_2CO_3(5.0)	36h	54
14	NBS (1.1)	4.0	$NaHCO_3$(5.0)	36h	19

[a] NMR yields were using pyrazine or hexamethyl disiloxane as the internal standard. [b] Isolated yields, single isomer. [c] No light. [d] Without isoquinoline.

Table S1 Optimization of reaction conditions

3.) Substrate Scope of Arenes and Characterization Data

General procedure (B):

An oven-dried 2 mL vial was charged with a magnetic stir bar, and to the solvent of the according arenes (0.2 mmol, 1.0 eq) in AcN (0.5 mL), NBS (39.2 mg, 0.22 mmol, 1.1 eq), B_2pin_2 (203.1 mg, 0.8 mmol, 4.0 eq), NH_4HCO_3 (79.1 mg, 1.0 mmol, 5.0 eq), isoquinoline (5.2 mg, 0.04 mmol, 20 mol%) were added. The vial was sealed with a plastic cap and then irradiated with a 2*390 nm LEDs light for 36 h. After filtrating the inorganic base, the solvent was removed by vacuum evaporation. NMR yield was determined by ^{1}H NMR using pyrazine or HMDSO as the internal standard. The reaction mixture was purified by flash chromatography on silica gel to afford the desired borylated product.

General procedure (C):

An oven-dried 2 mL vial was charged with a magnetic stir bar, and to the solvent of the according arenes (0.2 mmol, 1.0 eq) in AcN (0.5 mL), NBS (39.2 mg, 0.22 mmol, 1.1

eq), B_2pin_2 (203.1 mg, 0.8 mmol, 4.0 eq), NH_4HCO_3 (79.1 mg, 1.0 mmol, 5.0 eq), isoquinoline (25.8 mg, 0.2 mmol, 100 mol%) were added. The vial was sealed with a plastic cap and then irradiated with a 2*390 nm LEDs light for 72 h. After filtrating the inorganic base, the solvent was removed by vacuum evaporation. NMR yield was determined by ^1H NMR using pyrazine or HMDSO as the internal standard. The reaction mixture was purified by flash chromatography on silica gel to afford the desired borylated product.

2-(4-methoxyphenyl)-4,4,5,5-tetramethyl-1,3,2-dioxaborolane (1)

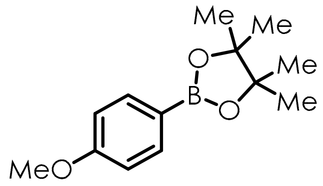

Following general procedure **(B)**, **1** was obtained as colorless liquid in 76% isolated yield from anisole using 40:1 petroleum ether/EtOAc as eluent.

^{1}H NMR (400 MHz, $CDCl_3$) δ 7.76 (d, J = 8.4 Hz, 2H), 6.90 (d, J = 8.6 Hz, 2H), 3.83 (s, 3H), 1.33 (s, 12H). **^{13}C NMR** (151 MHz, $CDCl_3$) δ 162.28, 136.64, 113.44, 83.67, 55.21, 24.99 (a signal for the carbon that is attached to the boron atom was not observed). **^{11}B NMR** (128 MHz, $CDCl_3$) δ 31.08. The NMR data were inconsistent with the reported data.[1]

4-(4,4,5,5-tetramethyl-1,3,2-dioxaborolan-2-yl) phenol (2)

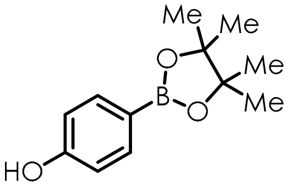

Following general procedure **(B)**, **2** was obtained as white solid in 33% isolated yield from phenol using 10:1 petroleum ether/EtOAc as eluent.

^{1}H NMR (400 MHz, $CDCl_3$) δ 7.70 (d, J = 8.4 Hz, 2H), 6.83 (d, J = 8.5 Hz, 12H), 5.72 (s, 1H), 1.34 (s, 12H). **^{13}C NMR** (151 MHz, $CDCl_3$) δ 158.74, 136.89, 115.01, 83.81, 24.95 (a signal for the carbon that is attached to the boron atom was not observed). **^{11}B NMR** (128 MHz, $CDCl_3$) δ 30.80. The NMR data were inconsistent with the reported data.[2]

2-(4-(triisopropylsilyloxy) phenyl)-4,4,5,5-tetramethyl-1,3,2-dioxaborolane (3)

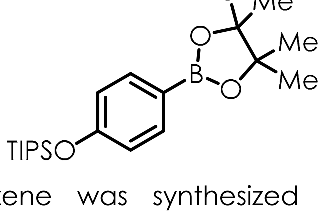

(*tri*-isopropylsilyloxy) benzene was synthesized according to the literature precedent:[3] Phenol (2 g, 21.25 mmol) and imidazole (3.62 g, 53.13 mmol) were dissolved in 130 mL of DCM. *tri*-isopropylsilyl chloride (5.45 mL, 25.5 mmol) was added to the mixture and the mixture was stirred at room temperature until no starting material remained using TLC. The reaction mixture was extracted using distilled H_2O (1 x 70 mL)

and the organic layer was washed with 1 N NaOH (1 x 70 mL) and brine (1 x 70 mL), dried over using Na_2SO_4 and concentrated to afford the pure product as a faint brown oil. Analytical data matched those reported in the literature.[3] **^{1}H NMR** (600 MHz, $CDCl_3$) δ7.22 (dt, J = 7.0, 4.4 Hz, 2H), 6.93 (dt, J = 7.6, 4.6 Hz, 1H), 6.89 (dd, J = 8.3, 2.7 Hz, 2H), 1.30 – 1.23 (m, 3H), 1.11 (dd, J = 7.9, 3.0 Hz, 18H).

Following general procedure **(B)**, **3** was obtained as a colorless liquid in 50% isolated yield from (*tri*-isopropylsilyloxy) benzene using 90:1 petroleum ether/EtOAc as eluent.

^{1}H NMR (400 MHz, $CDCl_3$) δ 7.68 (d, *J* = 8.4 Hz, 2H), 6.87 (d, *J* = 8.4 Hz, 2H), 1.33 (s, 12H), 1.28 – 1.25 (m, 3H), 1.09 (d, *J* = 7.4 Hz, 18H).**^{13}C NMR** (151 MHz, $CDCl_3$) δ 159.10, 136.57, 119.52, 83.65, 25.02, 18.05, 12.85 (a signal for the carbon that is attached to the boron atom was not observed). **^{11}B NMR** (128 MHz, $CDCl_3$) δ 32.12. The NMR data were inconsistent with the reported data.[4]

2-[4-[[(1,1-dimethylethyl) diphenylsilyl] oxy] phenyl]-4,4,5,5-tetramethyl-1,3,2-dioxaborolane (4)

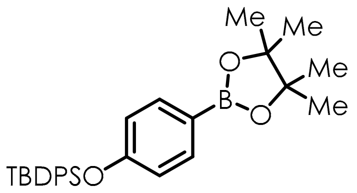

tert-Butyl(phenoxy)diphenylsilane was synthesized according to the literature precedent: [3] phenol (2 g, 21 mmol) and imidazole (2.6 g, 38.5 mmol) were dissolved in 20 mL of MeCN. *tert*-Butyldiphenylsilyl chloride (4.6 mL, 17.5 mmol) was added to the mixture and the reaction was allowed to stir overnight. The reaction was diluted with 40 mL Et_2O and poured into 50 mL of water. The organic layer was extracted with Et_2O (2 x 40 mL), washed with brine (1 x 50 mL) and dried using Na_2SO_4. The crude product was purified by column chromatography to afford the pure product as a colorless oil. Analytical data matched those reported in the literature. [5] **^{1}H NMR** (400 MHz, $CDCl_3$) δ 7.77 – 7.70 (m, 4H), 7.47 – 7.35 (m, 6H), 7.15 – 7.07 (m, 2H), 6.91 – 6.85 (m, 1H), 6.82 – 6.75 (m, 2H), 1.12 (s, 9H).

Following general procedure **(B)**, **4** was obtained as white solid in 32% isolated yield from *tert*-Butyl(phenoxy)diphenylsilane using 60:1 petroleum ether/EtOAc as eluent.

^{1}H NMR (400 MHz, $CDCl_3$) δ 7.73 (dd, J = 8.0, 1.5 Hz, 4H), 7.58 (d, J = 8.5 Hz, 2H), 7.46 – 7.34 (m, 6H), 6.81 – 6.74 (m, 2H), 1.31 (s, 12H), 1.11 (s, 9H). **^{13}C NMR** (101 MHz, $CDCl_3$) δ 136.37, 135.59, 132.87, 130.03, 127.91, 119.37, 83.61, 26.62, 25.17, 24.98, 19.60 (a signal for the carbon that is attached to the boron atom was not observed). **^{11}B NMR** (128 MHz, $CDCl_3$) δ 32.76. The NMR data were inconsistent with the reported data.[6]

4,4,5,5-tetramethyl-2-(4-phenethoxyphenyl)-1,3,2-dioxaborolane (5)

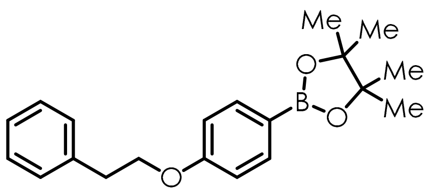

Following general procedure **(C)**, **5** was obtained as colorless liquid in 50% isolated yield from phenethoxybenzene using 60:1 petroleum ether/EtOAc as eluent.

^{1}H NMR (400 MHz, $CDCl_3$) δ 7.74 (d, J = 8.5 Hz, 2H), 7.33 – 7.25 (m, 5H), 6.89 (d, J = 8.5 Hz, 2H), 4.20 (t, J = 7.2 Hz, 2H), 3.10 (t, J = 7.2 Hz, 2H), 1.33 (s, 12H). **^{13}C NMR** (151 MHz, $CDCl_3$) δ 161.54, 138.30, 136.66, 129.15, 128.65, 126.66, 114.08, 83.70, 68.61, 35.89, 25.01 (a signal for the carbon that is attached to the boron atom was not observed). **^{11}B NMR** (128 MHz, $CDCl_3$) δ 31.80. **HRMS** calculated for $C_{20}H_{25}BO_3$ (M + H^+): 325.1970, found: 325.1977.

2-(4-(tert-butoxy) phenyl)-4,4,5,5-tetramethyl-1,3,2-dioxaborolane (6)

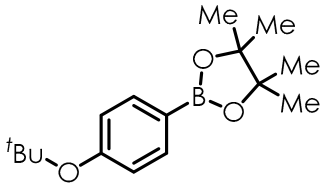

Following general procedure **(B)**, **6** was obtained as colorless liquid in 33% isolated yield from *tert*-butoxybenzene using 40:1 petroleum ether/EtOAc as eluent.

^{1}H NMR (400 MHz, $CDCl_3$) δ 7.72 (d, J = 8.4 Hz, 2H), 6.98 (d, J = 8.5 Hz, 2H), 1.36 (s, 9H), 1.33 (s, 12H). **^{13}C NMR** (101 MHz, $CDCl_3$) δ 135.91, 123.18, 83.79, 29.07, 25.02(signal for the carbon that is attached to the boron atom was not observed). **^{11}B NMR** (128 MHz, $CDCl_3$) δ 31.01. The NMR data were in consistent with the reported data.[7]

4,4,5,5-tetramethyl-2-(4-phenoxyphenyl)-1,3,2-dioxaborolane (7)

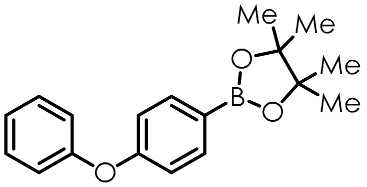

Following general procedure **(B)**, **7** was obtained as white solid in 50% isolated yield from diphenyl ether using 40:1 petroleum ether/EtOAc as eluent.

^{1}H NMR (400 MHz, $CDCl_3$) δ 7.79 (d, J = 8.5 Hz, 2H), 7.35 (t, J = 7.9 Hz, 2H), 7.13 (t, J = 7.4 Hz, 1H), 7.04 (d, J = 8.1 Hz, 2H), 6.99 (d, J = 8.5 Hz, 2H), 1.35 (s,12H). **^{13}C NMR** (101 MHz, $CDCl_3$) δ 160.33, 156.68, 136.77, 129.94, 123.80, 119.61, 117.81, 83.87, 25.00 (a signal for the carbon that is attached to the boron atom was not observed). **^{11}B NMR** (128 MHz, $CDCl_3$) δ 32.20. The NMR data were inconsistent with the reported data.[8]

4-(4,4,5,5-tetramethyl-1,3,2-dioxaborolan-2-yl) aniline (8)

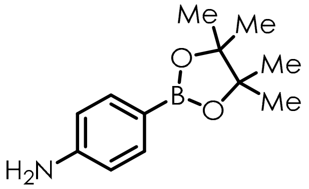

Following general procedure **(B)**, **8** was obtained as colorless solid in 45% isolated yield from aniline using 10:1 petroleum ether/EtOAc as eluent.

^{1}H NMR (400 MHz, $CDCl_3$) δ 7.62 (d, J = 7.9 Hz, 2H), 6.66 (d, J = 7.9 Hz,2H), 3.73 (brs, 2H), 1.32 (s, 12H). **^{13}C NMR** (101 MHz, $CDCl_3$) δ 149.41, 136.53, 114.21, 83.42, 24.97 (a

signal for the carbon that is attached to the boron atom was not observed). **^{11}B NMR** (128 MHz, $CDCl_3$) δ 30.86. The NMR data were inconsistent with the reported data.[1]

***N*-Methyl-4-(4,4,5,5-tetramethyl-1,3,2-dioxaborolan-2-yl) aniline (9)**

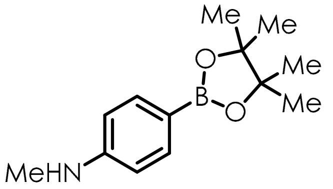

Following general procedure **(B)**, **9** was obtained as white solid in 54% isolated yield from *N*-Methylbenzenamine using 20:1 petroleum ether/EtOAc as eluent.

^{1}H NMR (400 MHz, $CDCl_3$) δ 7.65 (d, J = 8.5 Hz, 2H), 6.66 – 6.59 (m, 2H), 2.86 (s, 3H), 1.32 (s, 12H). **^{13}C NMR** (101 MHz, $CDCl_3$) δ 151.41, 136.48, 112.01, 83.37, 30.74, 24.98 (a signal for the carbon that is attached to the boron atom was not observed). **^{11}B NMR** (128 MHz, $CDCl_3$) δ 30.80. The NMR data were inconsistent with the reported data. [9]

***N*-ethyl-4-(4,4,5,5-tetramethyl-1,3,2-dioxaborolan-2-yl) aniline (10)**

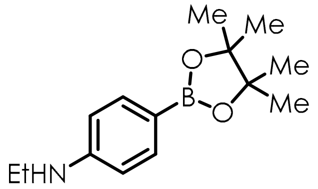

Following general procedure **(B)**, **10** was obtained as colorless solid in 51% isolated yield from *N*-ethylaniline using 60:1 petroleum ether/EtOAc as eluent.

^{1}H NMR (400 MHz, $CDCl_3$) δ 7.63 (d, J = 8.4 Hz, 2H), 6.57 (d, J = 8.5 Hz, 2H), 3.19 (q, J = 7.2 Hz, 2H), 1.32 (s, 12H), 1.25 (t, J = 7.2 Hz, 3H). **^{13}C NMR** (101 MHz, $CDCl_3$) δ 150.96, 136.49, 111.95, 83.30, 38.19, 24.97, 14.88 (a signal for the carbon that is attached to the boron atom was not observed). **^{11}B NMR** (128 MHz, $CDCl_3$) δ 31.09. **HRMS** calculated for $C_{14}H_{22}BNO_2$ (M + H^+): 248.1816, found: 248.1831.

***N, N*-dimethyl-4-(4,4,5,5-tetramethyl-1,3,2-dioxaborolan-2-yl) aniline (11)**

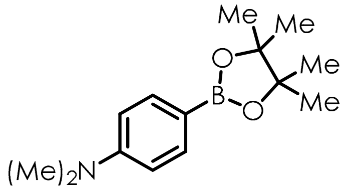

Following general procedure **(B)**, **11** was obtained as white solid in 34% isolated yield from *N, N*-dimethylaniline using 60:1 petroleum ether/EtOAc as eluent.

^{1}H NMR (400 MHz, $CDCl_3$) δ 7.69 (d, J = 8.2 Hz, 2H), 6.70 (d, J = 8.2 Hz, 2H), 2.99 (s, 6H), 1.32 (s, 12H). **^{13}C NMR** (151 MHz, $CDCl_3$) δ 152.66, 136.27, 111.38, 83.28, 40.24, 24.97 (a signal for the carbon that is attached to the boron atom was not observed). **^{11}B NMR** (128 MHz, $CDCl_3$) δ 31.39. The NMR data were inconsistent with the reported data.[2]

***N, N*-diethyl-4-(4,4,5,5-tetramethyl-1,3,2-dioxaborolan-2-yl) aniline (12)**

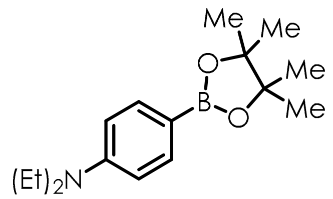

Following general procedure **(B)**, **12** was obtained as white solid in 40% isolated yield from *N, N*-diethylaniline using 40:1 petroleum ether/EtOAc as eluent.

¹H NMR (400 MHz, $CDCl_3$) δ 7.66 (d, J = 8.8 Hz, 2H), 6.64 (d, J = 8.8 Hz, 2H), 3.38 (q, J = 7.0 Hz, 4H), 1.32 (s, 12H), 1.16 (t, J = 7.0 Hz, 6H). **¹³C NMR** (151 MHz, $CDCl_3$) δ 150.09, 136.54, 110.76, 83.21, 44.36, 24.98, 12.72 (a signal for the carbon that is attached to the boron atom was not observed). **¹¹B NMR** (193 MHz, $CDCl_3$) δ 30.66. **HRMS** calculated for $C_{16}H_{26}BNO_2$ (M + H^+): 276.2129, found: 276.2151.

tert-butyl (4-(4,4,5,5-tetramethyl-1,3,2-dioxaborolan-2-yl) phenyl) carbamate (13)

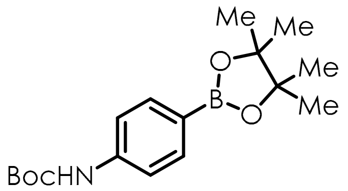

Following general procedure **(C)**, isoquinoline (25.8 mg, 0.2 mmol, 1.0 eq) was used and after irradiation for 72 h, **13** was obtained as a white solid in 55% isolated yield from *tert*-butyl phenylcarbamate using 40:1 petroleum ether/EtOAc as eluent.

¹H NMR (400 MHz, $CDCl_3$) δ 7.73 (d, J = 8.4 Hz, 2H), 7.36 (d, J = 8.3 Hz, 2H), 6.55 (s, 1H), 1.51 (s, 9H), 1.33 (s, 12H). **¹³C NMR** (151 MHz, $CDCl_3$) δ152.54, 141.23, 135.99, 117.35, 83.78, 80.85, 28.47, 25.00 (a signal for the carbon that is attached to the boron atom was not observed). **¹¹B NMR** (193 MHz, $CDCl_3$) δ 31.06. The NMR data were inconsistent with the reported data.[1]

_N_-(4-(4,4,5,5-tetramethyl-1,3,2-dioxaborolan-2-yl) phenyl) acetamide (14)

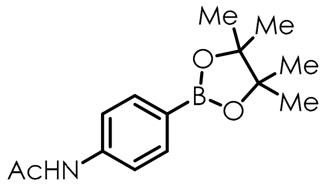

Following general procedure **(C)**, isoquinoline (25.8 mg, 0.2 mmol, 1.0 eq) was used and after irradiation for 72 h, **14** was obtained as colorless crystal in 48% isolated yield from *N*-phenylacetamide using 4:1 petroleum ether/EtOAc as eluent.

¹H NMR (400 MHz, $CDCl_3$) δ 7.76 (d, J = 8.2 Hz, 2H), 7.51 (d, J = 8.0 Hz, 2H), 2.17 (s, 3H), 1.33 (s, 12H). **¹³C NMR** (151 MHz, $CDCl_3$) δ 168.38, 140.70, 135.96, 118.67, 83.89, 25.01, 24.91(a signal for the carbon that is attached to the boron atom was not observed). **¹¹B NMR** (193 MHz, $CDCl_3$) δ 31.02. The NMR data were inconsistent with the reported data.[10]

4-[4-(4,4,5,5-Tetramethyl-1,3,2-dioxaborolan-2-yl) phenyl] morpholine (15)

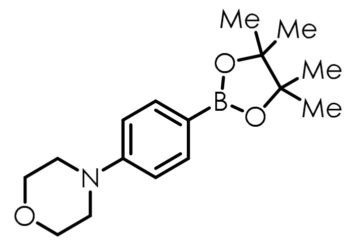

Following general procedure **(B)**, **15** was obtained as colorless crystal in 61% isolated yield from *N*-Phenylmorpholine using 40:1 petroleum ether/EtOAc as eluent.

^{1}H NMR (400 MHz, $CDCl_3$) δ 7.72 (d, *J* = 8.3 Hz, 2H), 6.88 (d, *J* = 8.3 Hz, 2H), 3.87 – 3.83 (m, 4H), 3.24 – 3.20 (m, 4H), 1.33 (s, 12H). **^{13}C NMR** (151 MHz, $CDCl_3$) δ 153.48, 136.29, 114.23, 83.54, 66.90, 48.52, 24.97 (a signal for the carbon that is attached to the boron atom was not observed). **^{11}B NMR** (193 MHz, $CDCl_3$) δ 31.05. The NMR data were inconsistent with the reported data.[11]

1-[4-(4,4,5,5-Tetramethyl-1,3,2-dioxaborolan-2-yl) phenyl] pyrrolidine (16)

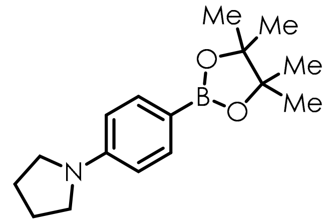

Following general procedure **(B)**, **16** was obtained as colorless crystal in 43% isolated yield from *N*-Phenylpyrolidine using 60:1 petroleum ether/EtOAc as eluent.

^{1}H NMR (400 MHz, $CDCl_3$) δ 7.67 (d, J = 8.6 Hz, 2H), 6.55 (d, J = 8.1 Hz, 2H), 3.35 – 3.28 (m, 4H), 2.03 – 1.97 (m, 4H), 1.32 (s, 12H). **^{13}C NMR** (151 MHz, $CDCl_3$) δ 150.04, 136.37, 111.22, 83.23, 47.69, 25.56, 24.98 (a signal for the carbon that is attached to the boron atom was not observed). **^{11}B NMR** (193 MHz, $CDCl_3$) δ 31.38. The NMR data were inconsistent with the reported data. [12]

2-(3,4-dimethoxyphenyl)-4,4,5,5-tetramethyl-1,3,2-dioxaborolane (17)

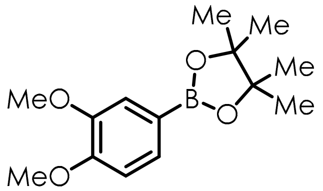

Following general procedure **(B)**, **17** was obtained as colorless solid in 64% isolated yield from 3,4-dimethoxybenzene using 30:1 petroleum ether/EtOAc as eluent.

^{1}H NMR (400 MHz, $CDCl_3$) δ 7.42 (d, J = 8.0 Hz, 1H), 7.28 (s, 1H), 6.87 (d, J = 8.0 Hz, 1H), 3.91 (s, 3H), 3.89 (s, 3H), 1.33 (s, 12H). **^{13}C NMR** (151 MHz, $CDCl_3$) δ 151.76, 148.46, 128.68, 116.70, 110.61, 83.77, 55.96, 55.85, 25.04 (d, J = 25.5 Hz) (a signal for the carbon that is attached to the boron atom was not observed). **^{11}B NMR** (128 MHz, $CDCl_3$) δ 30.69. The NMR data were inconsistent with the reported data.[1]

2-(benzo[d] [1,3] dioxol-5-yl)-4,4,5,5-tetramethyl-1,3,2-dioxaborolane (18)

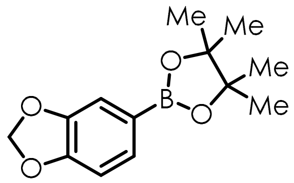

Following general procedure **(B)**, **18** was obtained as colorless liquid in 36% isolated yield from benzo[d] [1,3] dioxole using 60:1 petroleum ether/EtOAc as eluent.

^{1}H NMR (400 MHz, $CDCl_3$) δ 7.36 (d, J = 7.7 Hz, 1H), 7.24 (s, 1H), 6.83 (d, J = 7.6 Hz, 1H), 5.95 (s, 2H), 1.33 (s, 12H). **^{13}C NMR** (151 MHz, $CDCl_3$) δ 150.29, 147.32, 129.84, 114.06, 108.40, 100.84, 83.82, 24.95 (a signal for the carbon that is attached to the boron atom was not observed). **^{11}B NMR** (193 MHz, $CDCl_3$) δ 30.66. The NMR data were inconsistent with the reported data.[1]

2-(4-methoxy-3-methylphenyl)-4,4,5,5-tetramethyl-1,3,2-dioxaborolane (19)

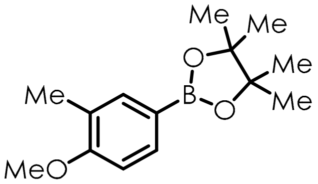

Following general procedure **(B)**, **19** was obtained as colorless solid in 26% isolated yield from 1-methoxy-2-methylbenzene using 40:1 petroleum ether/EtOAc as eluent.

^{1}H NMR (400 MHz, $CDCl_3$) δ 7.64 (dd, J = 8.1, 1.7 Hz, 1H), 7.59 (s, 1H), 6.82 (d, J = 8.1 Hz, 1H), 3.85 (s, 3H), 2.21 (s, 3H), 1.33 (s, 12H). **^{13}C NMR** (151 MHz, $CDCl_3$) δ 160.60, 137.30, 134.41, 109.43, 83.64, 55.34, 25.01, 16.09(signal for the carbon that is attached to the boron atom was not observed). **^{11}B NMR** (193 MHz, $CDCl_3$) δ 30.66. The NMR data were inconsistent with the reported data.[13]

methyl 2-methoxy-5-(4,4,5,5-tetramethyl-1,3,2-dioxaborolan-2-yl) benzoate (20)

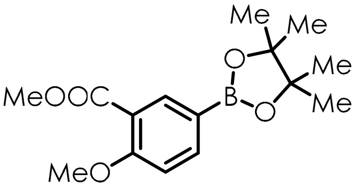

Following general procedure **(B)**, **20** was obtained as colorless solid in 55% isolated yield from methyl 2-methoxybenzoate using 10:1 petroleum ether/EtOAc as eluent.

^{1}H NMR (400 MHz, $CDCl_3$) δ 8.22 (s, 1H), 7.90 (d, J = 8.3 Hz, 1H), 6.96 (d, J = 8.4 Hz, 1H), 3.92 (s, 3H), 3.88 (s, 3H), 1.34 (s, 12H). **^{13}C NMR** (101 MHz, $CDCl_3$) δ 166.75, 161.59, 140.36, 138.50, 119.84, 111.35, 84.03, 56.08, 52.05, 25.01(a signal for the carbon that is attached to the boron atom was not observed). **^{11}B NMR** (128 MHz, $CDCl_3$) δ 31.59 The NMR data were inconsistent with the reported data.[14]

2-fluoro-4-(4,4,5,5-tetramethyl-1,3,2-dioxaborolan-2-yl)-phenol (21)

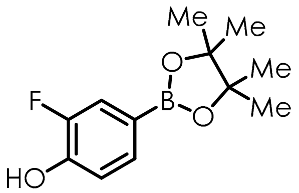

Following general procedure **(B)**, **21** was obtained as white solid in 30 % isolated yield from 2-fluorophenol using 6:1 petroleum ether/EtOAc as eluent.

^{1}H NMR (400 MHz, $CDCl_3$) δ 7.49 (dd, J = 9.5, 5.4 Hz, 2H), 6.98 (t, J = 8.3 Hz, 1H), 5.37 (s, 1H), 1.33 (s, 12H). **^{13}C NMR** (151 MHz, $CDCl_3$)δ150.93 (d, J = 238.6 Hz), 146.57 (d, J = 14.2 Hz), 131.94 (d, J = 3.4 Hz), 121.55 (d, J = 16.1 Hz), 117.04, 84.06, 24.92. **^{11}B NMR** (128 MHz, $CDCl_3$) δ 30.74.**^{19}F NMR** (377 MHz, $CDCl_3$) δ -141.94 (dd, J = 11.0, 8.4 Hz). The NMR data were inconsistent with the reported data.[15]

2-Methoxy-4-(4,4,5,5-tetramethyl-1,3,2-dioxaborolan-2-yl) aniline (22)

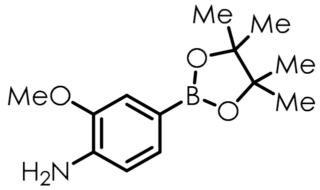

Following general procedure **(B)**, **22** was obtained as white solid in 51% isolated yield from 2-Aminomethoxybenzene using 10:1 petroleum ether/EtOAc as eluent.

^{1}H NMR (600 MHz, $CDCl_3$) δ 7.29 (d, J = 7.7 Hz, 1H), 7.20 (s, 1H), 6.70 (d, J = 7.7 Hz, 1H), 3.89 (s, 3H), 1.33 (s, 12H). **^{13}C NMR** (101 MHz, $CDCl_3$) δ 146.59, 139.56, 128.97, 115.96, 114.17, 83.48, 55.66, 24.98 (a signal for the carbon that is attached to the boron atom was not observed). **^{11}B NMR** (193 MHz, $CDCl_3$) δ 31.16. The NMR data were inconsistent with the reported data.[16]

2-Fluoro-4-(4,4,5,5-tetramethyl-1,3,2-dioxaborolan-2-yl) aniline (23)

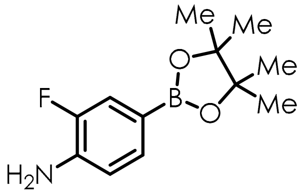

Following general procedure **(B)**, **23** was obtained as pale-yellow solid in 51% isolated yield from 2-Fluoroaniline using 40:1 petroleum ether/EtOAc as eluent.

^{1}H NMR (400 MHz, $CDCl_3$) δ7.44 – 7.34 (m, 2H), 6.76 (td, J = 8.2, 1.8 Hz, 1H), 1.32 (d, J = 1.8 Hz, 12H). **^{13}C NMR** (151 MHz, $CDCl_3$) δ 151.25 (d, J = 239.1 Hz), 137.66 (d, J = 12.6 Hz), 131.64 (d, J = 3.2 Hz), 121.13 (d, J = 16.5 Hz), 116.09 (d, J = 3.1 Hz), 83.73, 24.97 (a signal for the carbon that is attached to the boron atom was not observed). **^{11}B NMR** (128 MHz, $CDCl_3$) δ 35.04. **^{19}F NMR** (377 MHz, $CDCl_3$) δ -137.19 (t, J = 10.2 Hz). The NMR data were inconsistent with the reported data.[17]

4-(4,4,5,5-tetramethyl-1,3,2-dioxaborolan-2-yl)-2-(trifluoromethyl) aniline (24)

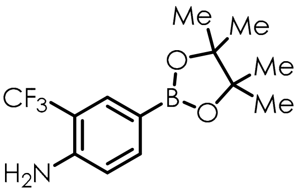

Following general procedure **(B)**, **24** was obtained as white solid in 42% isolated yield from 2-(trifluoromethyl) aniline using 40:1 petroleum ether/EtOAc as eluent.

^{1}H NMR (400 MHz, $CDCl_3$) δ 7.89 (s, 1H), 7.71 (d, J = 8.1 Hz, 1H), 6.70 (d, J = 8.1 Hz, 1H), 1.32 (s, 12H). **^{13}C NMR** (151 MHz, $CDCl_3$) δ 147.06, 139.46, 133.85 – 133.73 (m), 125.23 (d, J = 272.1 Hz), 116.26, 113.23 (d, J = 30.2 Hz), 83.85, 24.98 (a signal for the carbon that is attached to the boron atom was not observed). **^{11}B NMR** (128 MHz, $CDCl_3$) δ 31.79. **^{19}F NMR** (377 MHz, $CDCl_3$) δ -62.53. The NMR data were inconsistent with the reported data.[18]

4-(4,4,5,5-tetramethyl-1,3,2-dioxaborolan-2-yl)-2-(trifluoromethoxy) aniline (25)

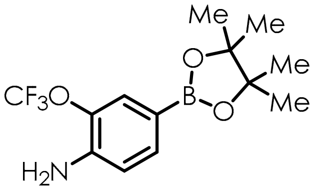

Following general procedure **(B)**, **25** was obtained as colorless solid in 75% isolated yield from 2-(trifluoromethoxy) aniline using 20:1 petroleum ether/EtOAc as eluent.

^{1}H NMR (400 MHz, $CDCl_3$) δ 7.56 (s, 1H), 7.52 (d, J = 7.9 Hz, 1H), 6.77 (d, J = 8.0 Hz, 1H), 1.32 (s, 12H). **^{13}C NMR** (151 MHz, $CDCl_3$) δ 141.91, 136.08, 134.59, 128.02, 120.23, 116.15, 83.82, 24.98 (a signal for the carbon that is attached to the boron atom was not observed). **^{11}B NMR** (193 MHz, $CDCl_3$) δ 30.27. **^{19}F NMR** (377 MHz, $CDCl_3$) δ -57.91. The NMR data were inconsistent with the reported data.[18]

2-(difluoromethoxy)-4-(4,4,5,5-tetramethyl-1,3,2-dioxaborolan-2-yl)- aniline (26)

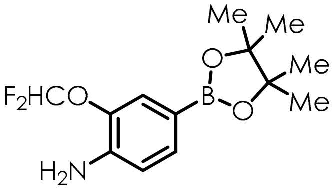

Following general procedure **(B)**, **26** was obtained as colorless crystal in 65% isolated yield from 2-(Difluoromethoxy) aniline using 20:1 petroleum ether/EtOAc as eluent.

^{1}H NMR (400 MHz, $CDCl_3$) δ 7.47 (dd, J = 7.9, 1.3 Hz, 1H), 7.42 (d, J = 1.3 Hz, 1H), 6.76 (d, J = 7.9 Hz, 1H), 6.50 (t, J = 74.5 Hz, 1H), 1.32 (s, 12H). **^{13}C NMR** (151 MHz, $CDCl_3$) δ 141.69, 138.34, 133.54, 125.84, 117.14 (t, J = 258.9 Hz), 115.70, 83.78, 24.97 (a signal for the carbon that is attached to the boron atom was not observed). **^{11}B NMR** (128 MHz, $CDCl_3$) δ 35.04. **^{19}F NMR** (377 MHz, $CDCl_3$) δ -79.18 (d, J = 74.7 Hz). The NMR data were inconsistent with the reported data.[18]

2-amino-5-(4,4,5,5-tetramethyl-1,3,2-dioxaborolan-2-yl) benzonitrile (27)

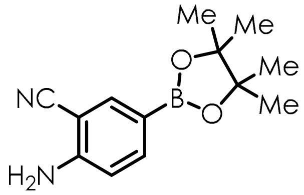

Following general procedure **(B)**, **27** was obtained as white solid in 93% isolated yield from 2-aminobenzonitrile using 20:1 petroleum ether/EtOAc as eluent.

^{1}H NMR (400 MHz, $CDCl_3$) δ 7.87 (d, J = 1.5 Hz, 1H), 7.72 (dd, J = 8.3, 1.5 Hz, 1H), 6.70 (d, J = 8.3 Hz, 1H), 4.57 (s, 2H), 1.32 (s, 12H). **^{13}C NMR** (151 MHz, $CDCl_3$) δ 151.65, 140.12 (d, J = 36.7 Hz), 117.54, 114.27, 95.93, 84.04, 24.98 (a signal for the carbon that is attached to the boron atom was not observed). **^{11}B NMR** (193 MHz, $CDCl_3$) δ 30.52. The NMR data were inconsistent with the reported data.[18]

2,4-bis(4,4,5,5-tetramethyl-1,3,2-dioxaborolan-2-yl) aniline (28)

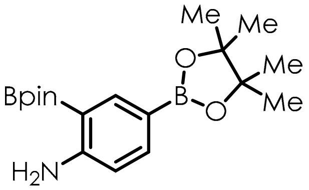

Following general procedure **(B)**, **28** was obtained as colorless solid in 77% isolated yield from 2-(4,4,5,5-tetramethyl-1,3,2-dioxaborolan-2-yl) aniline using 10:1 petroleum ether/EtOAc as eluent.

^{1}H NMR (400 MHz, $CDCl_3$) δ 8.11 (d, J = 1.6 Hz, 1H), 7.67 (dd, J = 8.1, 1.6 Hz, 1H), 6.64 (d, J = 8.1 Hz, 1H), 1.33 (s, 12H), 1.31 (s, 12H). **^{13}C NMR** (151 MHz, $CDCl_3$) δ 155.26, 144.57, 139.64, 114.59, 83.75, 83.38, 25.06, 24.99 (a signal for the carbons that are attached to the boron atoms were not observed). **^{11}B NMR** (193 MHz, $CDCl_3$) δ 31.13. The NMR data were inconsistent with the reported data.[16]

2-Fluoro-*N*-methyl-4-(tetramethyl-1,3,2-dioxaborolan-2-yl) aniline (29)

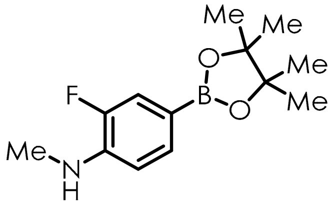

Following general procedure **(B)**, **29** was obtained as colorless solid in 66% isolated yield from 2-Fluoro-*N*-methylaniline using 40:1 petroleum ether/EtOAc as eluent.

^{1}H NMR (400 MHz, $CDCl_3$) δ 7.48 (dd, J = 7.8, 1.3 Hz, 1H), 7.37 (dd, J = 12.2, 1.3 Hz, 1H), 6.66 (t, J = 8.2 Hz, 1H), 2.90 (s, 3H), 1.32 (s, 12H). **^{13}C NMR** (151 MHz, $CDCl_3$) δ 151.20 (d, J = 239.1 Hz), 140.40, 132.13 (d, J = 3.0 Hz), 119.78 (d, J = 16.2 Hz), 110.79, 83.63, 30.11, 24.97 (a signal for the carbon that is attached to the boron atom was not observed). **^{11}B NMR** (193 MHz, $CDCl_3$) δ 30.84. **^{19}F NMR** (377 MHz, $CDCl_3$) δ -139.06. The NMR data were inconsistent with the reported data.[19]

4-Methyl-2-(4,4,5,5-tetramethyl-1,3,2-dioxaborolan-2-yl) aniline (30)

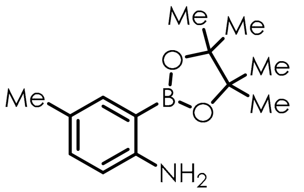

Following general procedure **(B)**, **30** was obtained as colorless solid in 40% isolated yield from 4-Methylbenzenamine using 40:1 petroleum ether/EtOAc as eluent.

^{1}H NMR (400 MHz, $CDCl_3$) δ 7.43 (d, J = 2.3 Hz, 1H), 7.05 (dd, J = 8.1, 2.2 Hz, 1H), 6.59 (d, J = 8.2 Hz, 1H), 2.22 (s, 3H), 1.34 (s, 12H). **^{13}C NMR** (151 MHz, $CDCl_3$) δ 150.66, 136.85, 133.74, 126.62, 115.58, 83.72, 25.05, 20.38 (a signal for the carbon that is attached to the boron atom was not observed). **^{11}B NMR** (193 MHz, $CDCl_3$) δ 31.52. The NMR data were inconsistent with the reported data.[20]

Methyl 4-amino-3-(4,4,5,5-tetramethyl-1,3,2-dioxaborolan-2-yl) benzoate (31)

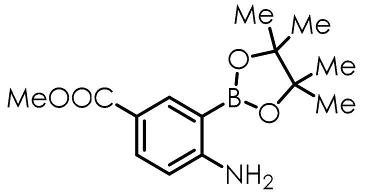

Following general procedure **(B)**, **31** was obtained as white solid in 62% isolated yield from 4-(Methoxycarbonyl) aniline using 10:1 petroleum ether/EtOAc as eluent.

^{1}H NMR (400 MHz, $CDCl_3$) δ 8.31 (d, J = 2.2 Hz, 1H), 7.87 (dd, J = 8.6, 2.2 Hz, 1H), 6.55 (d, J = 8.6 Hz, 1H), 3.84 (s, 3H), 1.34 (s, 12H). **^{13}C NMR** (101 MHz, $CDCl_3$) δ 167.40, 157.56, 139.64, 134.48, 118.12, 114.03, 83.92, 51.56, 24.97 (a signal for the carbon that is attached to the boron atom was not observed). **^{11}B NMR** (128 MHz, $CDCl_3$) δ 30.96. The NMR data were inconsistent with the reported data.[21]

2-(4-methoxy-2-methylphenyl)-4,4,5,5-tetramethyl-1,3,2-dioxaborolane (32)

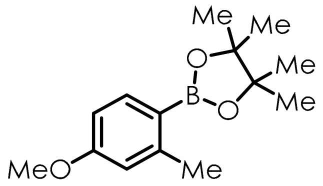

Following general procedure **(B)**, **32** was obtained as colorless oil in 30% isolated yield from 1-methoxy-3-methylbenzene using 40:1 petroleum ether/EtOAc as eluent.

^{1}H NMR (400 MHz, $CDCl_3$) δ 7.72 (d, J = 9.0 Hz, 1H), 6.73 – 6.67 (m, 2H), 3.80 (s, 3H), 2.52 (s, 3H), 1.33 (s, 12H). **^{13}C NMR** (101 MHz, $CDCl_3$) δ 161.83, 147.38, 137.97, 115.65, 110.26, 83.27, 55.14, 25.02, 22.55 (a signal for the carbon that is attached to the boron atom was not observed). **^{11}B NMR** (128 MHz, $CDCl_3$) δ 32.12. The NMR data were inconsistent with the reported data.[22]

methyl 4-amino-2-methoxy-5-(4,4,5,5-tetramethyl-1,3,2-dioxaborolan-2-yl) benzoate (33)

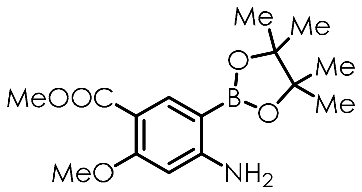

Following general procedure **(B)**, **33** was obtained as white solid in 76% isolated yield from 4-amino-2-methoxyphenyl acetate using 8:1 petroleum ether/EtOAc as eluent.

^{1}H NMR (400 MHz, $CDCl_3$) δ 8.19 (s, 1H), 6.06 (s, 1H), 3.85 (s, 3H), 3.81 (s, 3H), 1.32 (s, 12H). **^{13}C NMR** (101 MHz, $CDCl_3$) δ 166.16, 164.09, 158.87, 142.81, 134.35, 96.70, 83.74, 55.77, 51.44, 25.02 (a signal for the carbon that is attached to the boron atom was not observed). **^{11}B NMR** (128 MHz, $CDCl_3$) δ 31.02. **HRMS** calculated for $C_{15}H_{22}BNO_5$ (M + H$^+$): 308.1664, found: 308.1679.

2-(4,5-dimethoxy-2-methylphenyl)-4,4,5,5-tetramethyl-1,3,2-dioxaborolane (34)

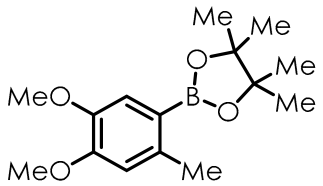

Following general procedure **(B)**, **34** was obtained as white solid in 55% isolated yield from 1,2-dimethoxy-4-methylbenzene using 20:1 petroleum ether/EtOAc as eluent.

^{1}H NMR (400 MHz, $CDCl_3$) δ 7.19 (s, 1H), 6.62 (s, 1H), 3.83 (s, 3H), 3.81 (s, 3H), 2.44 (s, 3H), 1.27 (s, 12H). **^{13}C NMR** (151 MHz, $CDCl_3$) δ 151.04, 146.24, 139.15, 118.38, 113.31, 83.33, 56.13, 55.75, 25.00, 21.79 (a signal for the carbon that is attached to the boron atom was not observed). **^{11}B NMR** (193 MHz, $CDCl_3$) δ 30.70. **HRMS** calculated for $C_{15}H_{23}BO_4$ (M + H$^+$): 279.1762, found: 279.1777.

5-methoxy-2-(4,4,5,5-tetramethyl-1,3,2-dioxaborolan-2-yl)-4-(trifluoromethyl) aniline (35)

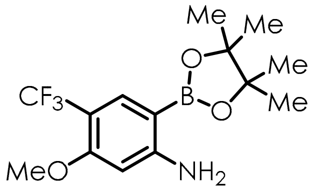

Following general procedure **(B)**, **35** was obtained as colorless crystal in 65% isolated yield from 3-methoxy-4-(trifluoromethyl) aniline using 10:1 petroleum ether/EtOAc as eluent.

^{1}H NMR (400 MHz, $CDCl_3$) δ 7.78 (s, 1H), 6.11 (s, 1H), 3.84 (s, 3H), 1.33 (s, 12H). **^{13}C NMR** (101 MHz, $CDCl_3$) δ 161.43, 158.37, 136.59 (d, J = 5.1 Hz), 124.55 (d, J = 270.6 Hz), 108.20 (d, J = 31.2 Hz), 97.14, 83.81, 55.65, 25.02 (a signal for the carbon that is attached to the boron atom was not observed). **^{11}B NMR** (128 MHz, $CDCl_3$) δ 30.65. **^{19}F NMR** (377 MHz, $CDCl_3$) δ -60.54. **HRMS** calculated for $C_{14}H_{19}BF_3NO_3$ (M + H$^+$): 318.1483, found: 318.1498.

2-methyl-6-(4,4,5,5-tetramethyl-1,3,2-dioxaborolan-2-yl)-4-(trifluoromethyl) aniline (36)

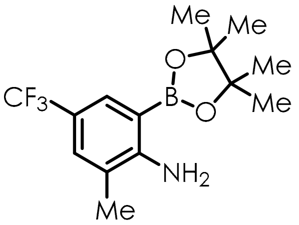

Following general procedure **(B)**, **36** was obtained as colorless crystal in 68% isolated yield from 2-methyl-4-(trifluoromethyl) aniline using 40:1 petroleum ether/EtOAc as eluent.

^{1}H NMR (600 MHz, $CDCl_3$) δ 7.76 (s, 1H), 7.33 (s, 1H), 2.16 (s, 3H), 1.35 (s, 12H). **^{13}C NMR** (151 MHz, $CDCl_3$) δ 154.49, 132.15 (d, J = 3.9 Hz), 130.20 (d, J = 3.6 Hz), 125.18 (d, J = 270.5 Hz), 121.25, 118.53 (d, J = 32.3 Hz), 84.12, 25.04, 17.61 (a signal for the carbon that is attached to the boron atom was not observed). **^{11}B NMR** (128 MHz, $CDCl_3$) δ 31.47. **^{19}F NMR** (377 MHz, $CDCl_3$) δ -60.99. **HRMS** calculated for $C_{14}H_{19}BF_3NO_2$ (M + H$^+$): 302.1534, found: 302.1551.

ethyl 4-amino-3-methoxy-5-(4,4,5,5-tetramethyl-1,3,2-dioxaborolan-2-yl) benzoate (37)

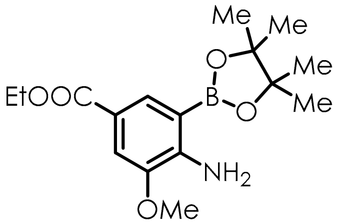

Following general procedure **(B)**, **37** was obtained as white solid in 67% isolated yield from ethyl 4-amino-3-methoxybenzoate using 8:1 petroleum ether/EtOAc as eluent.

^{1}H NMR (400 MHz, $CDCl_3$) δ 7.98 (d, J = 1.8 Hz, 1H), 7.46 (d, J = 1.9 Hz, 1H), 4.32 (q, J = 7.1 Hz, 2H), 3.89 (s, 3H), 1.37 (t, J = 7.1 Hz, 3H), 1.35 (s, 12H). **^{13}C NMR** (151 MHz, $CDCl_3$) δ 167.22, 148.34, 145.69, 131.47, 117.97, 112.97, 83.93, 60.44, 55.77, 25.05, 14.70 (a signal for the carbon that is attached to the boron atom was not observed). **^{11}B NMR** (193 MHz, $CDCl_3$) δ 31.05. **HRMS** calculated for $C_{16}H_{24}BNO_5$ (M + H$^+$): 322.1820, found: 322.1837.

2-(4-methoxy-2,3-dimethylphenyl)-4,4,5,5-tetramethyl-1,3,2-dioxaborolane (38)

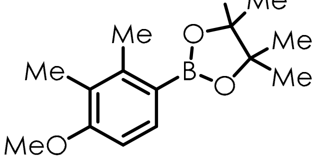

Following general procedure **(B)**, **38** was obtained as colorless crystal in 70% isolated yield from ethyl 1-methoxy-2,3-dimethylbenzene using 60:1 petroleum ether/EtOAc as eluent.

¹H NMR (400 MHz, $CDCl_3$) δ 7.63 (d, J = 8.3 Hz, 1H), 6.71 (d, J = 8.3 Hz, 1H), 3.82 (s, 3H), 2.49 (s, 3H), 2.14 (s, 3H), 1.33 (s, 12H). **¹³C NMR** (151 MHz, $CDCl_3$) δ 159.80, 144.95, 134.88, 125.12, 107.15, 83.28, 55.52, 25.00, 18.84, 11.67(a signal for the carbon that is attached to the boron atom was not observed). **¹¹B NMR** (193 MHz, $CDCl_3$) δ 31.55. **HRMS** calculated for $C_{15}H_{23}BO_3$ (M + H^+): 263.1813, found: 263.1831.

6-methoxy-2-methyl-3-(4,4,5,5-tetramethyl-1,3,2-dioxaborolan-2-yl) phenol (39)

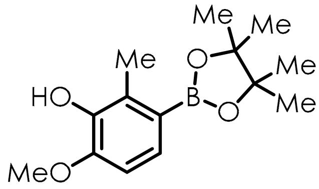

Following general procedure **(B)**, **39** was obtained as colorless liquid in 31% isolated yield from 2-methoxy-6-methylphenol using 20:1 petroleum ether/EtOAc as eluent.

¹H NMR (400 MHz, $CDCl_3$) δ 7.32 (d, J = 8.2 Hz, 1H), 6.71 (d, J = 8.2 Hz, 1H), 5.65 (s, 1H), 3.89 (s, 3H), 2.46 (s, 3H), 1.33 (s, 12H). **¹³C NMR** (151 MHz, $CDCl_3$) δ 148.45, 143.53, 130.51, 128.07, 107.39, 83.35, 55.99, 25.00, 14.29 (a signal for the carbon that is attached to the boron atom was not observed). **¹¹B NMR** (193 MHz, $CDCl_3$) δ 30.87. **HRMS** calculated for $C_{14}H_{21}BO_4$ (M + H^+): 265.1606, found: 265.1604.

2,6-dimethyl-4-(4,4,5,5-tetramethyl-1,3,2-dioxaborolan-2-yl) phenol (40)

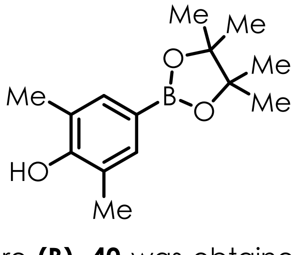

Following general procedure **(B)**, **40** was obtained as white solid in 26% isolated yield from 2,6-dimethylphenol using 20:1 petroleum ether/EtOAc as eluent.

¹H NMR (400 MHz, $CDCl_3$) δ 7.45 (s, 2H), 4.84 (s, 1H), 2.25 (s, 6H), 1.33 (s, 12H). **¹³C NMR** (151 MHz, $CDCl_3$) δ 155.27, 135.72, 122.43, 83.65, 24.99, 15.67 (a signal for the carbon that is attached to the boron atom was not observed). **¹¹B NMR** (193 MHz, $CDCl_3$) δ 30.69. The NMR data were inconsistent with the reported data.[23]

2,6-dimethyl-4-(4,4,5,5-tetramethyl-1,3,2-dioxaborolan-2-yl) aniline (41)

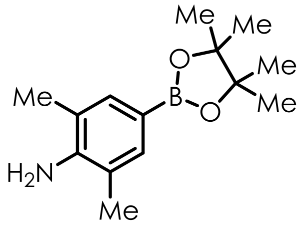

Following general procedure **(B)**, **41** was obtained as colorless crystal in 30% isolated yield from 2,6-dimethylaniline using 10:1 petroleum ether/EtOAc as eluent.

^{1}H NMR (400 MHz, $CDCl_3$) δ 7.42 (s, 2H), 3.79 (s, 2H), 2.18 (s, 6H), 1.33 (s, 12H). **^{13}C NMR** (151 MHz, $CDCl_3$) δ 146.06, 135.27, 120.78, 83.37, 24.97, 17.40 (a signal for the carbon that is attached to the boron atom was not observed). **^{11}B NMR** (193 MHz, $CDCl_3$) δ 31.16. The NMR data were inconsistent with the reported data.[24]

2,6-dimethoxy-4-(4,4,5,5-tetramethyl-1,3,2-dioxaborolan-2-yl) aniline (42)

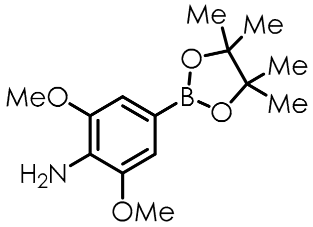

Following general procedure **(B)**, **42** was obtained as colorless crystal in 40%isolated yield from 2,6-dimethoxyaniline using 10:1 petroleum ether/EtOAc as eluent.

^{1}H NMR (400 MHz, $CDCl_3$) δ 6.98 (s, 2H), 4.06 (s, 2H), 3.89 (s, 6H), 1.33 (s, 12H). **^{13}C NMR** (151 MHz, $CDCl_3$) δ 146.81, 129.24, 110.43, 83.57, 56.11, 25.00 (a signal for the carbon that is attached to the boron atom was not observed). **^{11}B NMR** (193 MHz, $CDCl_3$) δ 31.39. **HRMS** calculated for $C_{14}H_{22}BNO_4$ (M + H$^+$): 280.1715, found: 280.1733.

2,6-difluoro-4-(4,4,5,5-tetramethyl-1,3,2-dioxaborolan-2-yl) aniline (43)

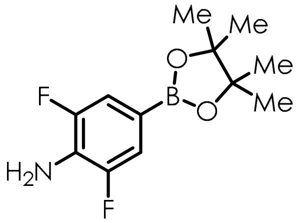

Following general procedure **(B)**, **43** was obtained as white solid in 61% isolated yield from 2,6-difluoroaniline using 20:1 petroleum ether/EtOAc as eluent.

^{1}H NMR (400 MHz, $CDCl_3$) δ 7.24 (dd, J = 6.8, 2.2 Hz, 2H), 3.93 (s, 2H), 1.32 (s, 12H). **^{13}C NMR** (151 MHz, $CDCl_3$) δ 152.57 – 150.63 (m), 127.08 (t, J = 16.1 Hz), 116.85 (dd, J = 14.3, 4.8 Hz), 84.05, 24.97 (a signal for the carbon that is attached to the boron atom was not observed). **^{11}B NMR** (193 MHz, $CDCl_3$) δ 30.44. **^{19}F NMR** (377 MHz, $CDCl_3$) δ -133.90 (dd, J = 6.6, 2.2 Hz). The NMR data were inconsistent with the reported data.[25]

2-(4-methoxy-2,3,6-trimethylphenyl)-4,4,5,5-tetramethyl-1,3,2-dioxaborolane (44)

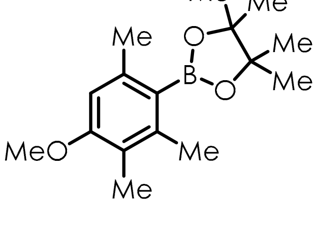

Following general procedure **(B)**, **44** was obtained as colorless crystal in 33% isolated yield from 1-methoxy-2,3,5-trimethylbenzene using 80:1 petroleum ether/EtOAc as eluent.

^{1}H NMR (400 MHz, $CDCl_3$) δ 6.51 (s, 1H), 3.78 (s, 3H), 2.38 (s, 3H), 2.31 (s, 3H), 2.08 (s, 3H), 1.38 (s, 12H). **^{13}C NMR** (151 MHz, $CDCl_3$) δ 158.36, 141.64, 140.21, 121.78, 109.57, 83.57, 55.50, 25.13, 22.55, 19.80, 11.32 (a signal for the carbon that is attached to the boron atom was not observed). **^{11}B NMR** (193 MHz, $CDCl_3$) δ 32.35. **HRMS** calculated for $C_{16}H_{25}BO_3$ (M + H$^+$): 277.1970, found: 277.1979.

2-(benzofuran-5-yl)-4,4,5,5-tetramethyl-1,3,2-dioxaborolane (45)

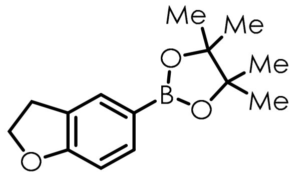

Following general procedure **(B)**, **45** was obtained as colorless liquid in 35% isolated yield from 2,3-dihydrobenzofuran using 60:1 petroleum ether/EtOAc as eluent.

^{1}H NMR (600 MHz, $CDCl_3$) δ 7.66 (s, 1H), 7.60 (d, J = 8.0 Hz, 1H), 6.79 (d, J = 8.0 Hz, 1H), 4.57 (t, J = 8.7 Hz, 2H), 3.19 (t, J = 8.7 Hz, 2H), 1.33 (s, 12H). **^{13}C NMR** (101 MHz, $CDCl_3$) δ 163.01, 135.71, 131.66, 126.65, 109.14, 83.64, 71.49, 29.35, 25.00 (a signal for the carbon that is attached to the boron atom was not observed). **^{11}B NMR** (128 MHz, $CDCl_3$) δ 31.80. The NMR data were i consistent with the reported data.[26]

2-(chroman-6-yl)-4,4,5,5-tetramethyl-1,3,2-dioxaborolane (46)

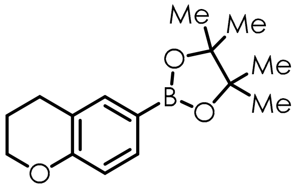

Following general procedure **(B)**, **46** was obtained as colorless solid in 55% isolated yield from chromane using 40:1 petroleum ether/EtOAc as eluent.

^{1}H NMR (600 MHz, $CDCl_3$) δ 7.53 (d, J = 8.6 Hz, 1H), 7.52 (s, 1H), 6.78 (d, J = 8.0 Hz, 1H), 4.22 – 4.17 (m, 2H), 2.79 (t, J = 6.5 Hz, 2H), 2.02 – 1.97 (m, 2H), 1.33 (s, 12H). **^{13}C NMR** (101 MHz, $CDCl_3$) δ 157.86, 137.02, 134.19, 121.77, 116.39, 83.63, 66.81, 24.98, 24.83, 22.46 (a signal for the carbon that is attached to the boron atom was not observed). **^{11}B NMR** (193 MHz, $CDCl_3$) δ 30.92. **HRMS** calculated for $C_{15}H_{21}BO_3$ (M + H$^+$): 261.1657, found: 261.1664.

1-methyl-6-(4,4,5,5-tetramethyl-1,3,2-dioxaborolan-2-yl)-1,2,3,4-tetrahydroquinoline (47)

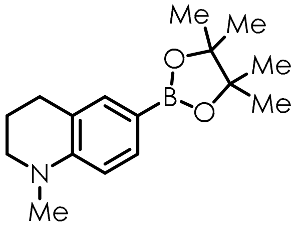

Following general procedure **(B)**, **47** was obtained as white solid in 45% isolated yield from 1-methyl-1,2,3,4-tetrahydroquinoline using 60:1 petroleum ether/EtOAc as eluent.

^{1}H NMR (400 MHz, $CDCl_3$) δ 7.53 (dd, J = 8.2, 1.6 Hz, 1H), 7.40 (d, J = 1.6 Hz, 1H), 6.55 (d, J = 8.2 Hz, 1H), 3.30 – 3.23 (m, 2H), 2.91 (s, 3H), 2.76 (t, J = 6.4 Hz, 2H), 2.00 – 1.91 (m, 2H), 1.32 (s, 12H). **^{13}C NMR** (151 MHz, $CDCl_3$) δ 149.18, 135.43, 134.58, 121.75, 109.98, 83.23, 51.35, 38.90, 27.77, 24.97, 22.40 (a signal for the carbon that is attached to the boron atom was not observed). **^{11}B NMR** (193 MHz, $CDCl_3$) δ 31.12. The NMR data were inconsistent with the reported data.[27]

7-(4,4,5,5-tetramethyl-1,3,2-dioxaborolan-2-yl)-3,4-dihydro-2H-benzo[b] [1,4] oxazine (48)

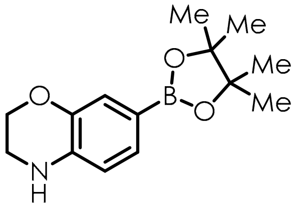

Following general procedure **(B)**, **48** was obtained as colorless solid in 28% isolated yield from 3,4-dihydro-2H-benzo[b] [1,4] oxazine using 40:1 petroleum ether/EtOAc as eluent.

^{1}H NMR (400 MHz, $CDCl_3$) δ 7.25 – 7.19 (m, 2H), 6.56 (d, J = 8.1 Hz, 1H), 4.24 – 4.19 (m, 2H), 3.94 (s, 1H), 3.44 (dd, J = 5.3, 3.5 Hz, 2H), 1.31 (s, 12H). **^{13}C NMR** (151 MHz, $CDCl_3$) δ 143.42, 136.86, 128.69, 123.08, 114.62, 83.45, 64.94, 41.11, 24.97 (a signal for the carbon that is attached to the boron atom was not observed). **^{11}B NMR** (193 MHz, $CDCl_3$) δ 30.57. The NMR data were inconsistent with the reported data.[28]

Anaesthesine derivative

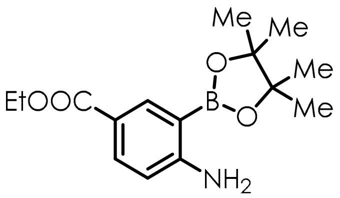

Following general procedure **(B)**, Anaesthesine derivative was obtained as colorless oil in 75% isolated yield from 4-(Ethoxycarbonyl) aniline using 10:1 petroleum ether/EtOAc as eluent.

^{1}H NMR (400 MHz, $CDCl_3$) δ 8.31 (d, J = 2.2 Hz, 1H), 7.88 (dd, J = 8.6, 2.2 Hz, 1H), 6.55 (d, J = 8.6 Hz, 1H), 4.31 (q, J = 7.1 Hz, 2H), 1.37 (d, J = 7.1 Hz, 3H), 1.34 (s, 12H). **^{13}C NMR** (101 MHz, $CDCl_3$) δ 166.93, 157.46, 139.62, 134.53, 118.77, 114.04, 83.98, 60.32, 25.06, 14.65 (a signal for the carbon that is attached to the boron atom was not observed). **^{11}B NMR** (128 MHz, $CDCl_3$) δ 32.31. **HRMS** calculated for $C_{15}H_{22}BNO_4$ (M + H$^+$): 292.1715, found: 292.1735.

Thymol derivative

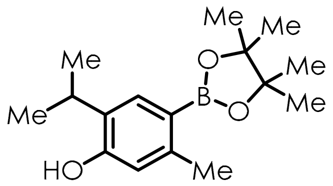

Following general procedure **(B)**, Thymol derivative was obtained as colorless crystal in 53% isolated yield from 2-isopropyl-5-methylphenol using 40:1 petroleum ether/EtOAc as eluent.

^{1}H NMR (400 MHz, $CDCl_3$) δ 7.60 (s, 1H), 6.55 (s, 1H), 4.88 (s, 1H), 3.14 (hept, J = 6.9 Hz, 1H), 2.45 (s, 3H), 1.32 (s, 12H), 1.26 (d, J = 6.9 Hz, 6H). **^{13}C NMR** (151 MHz, $CDCl_3$) δ 155.20, 144.70, 134.97, 130.63, 117.08, 83.23, 27.31, 25.04, 22.69, 21.84 (a signal for the carbon that is attached to the boron atom was not observed). **^{11}B NMR** (193 MHz, $CDCl_3$) δ 31.28. **HRMS** calculated for $C_{16}H_{25}BO_3$ (M + H$^+$): 277.1970, found: 277.1975.

Atomoxetine derivative

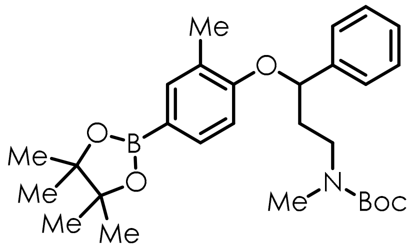

Following general procedure **(B)**, Atomoxetine derivative was obtained as colorless oil in 37% isolated yield from *tert*-butyl methyl(3-phenyl-3-(o-tolyloxy) propyl) carbamate using 15:1 petroleum ether/EtOAc as eluent.

^{1}H NMR (600 MHz, $CDCl_3$) δ 7.58 (s, 1H), 7.43 (d, J = 6.6 Hz, 1H), 7.30 (d, J = 5.0 Hz, 4H), 7.22 (dt, J = 9.1, 4.6 Hz, 1H), 6.57 (d, J = 8.2 Hz, 1H), 5.21 (d, J = 8.5 Hz, 1H), 3.41 (s, 2H), 2.83 (s, 3H), 2.33 (s, 3H), 2.24 – 2.05 (m, 2H), 1.43 – 1.34 (m, 9H), 1.29 (d, J = 3.6 Hz, 12H). **^{13}C NMR** (151 MHz, $CDCl_3$) δ 158.55, 155.89, 141.60, 137.32, 134.08, 128.80, 127.69, 126.36, 125.74, 112.11, 83.56, 79.51, 46.10, 37.39, 34.61, 28.52, 24.95 (d, J = 9.8 Hz), 16.41(a signal for the carbon that is attached to the boron atom was not observed).
HRMS calculated for $C_{28}H_{40}BNO_5$ (M + Na$^+$): 504.28917, found: 504.28864.

Levodropropizine derivative

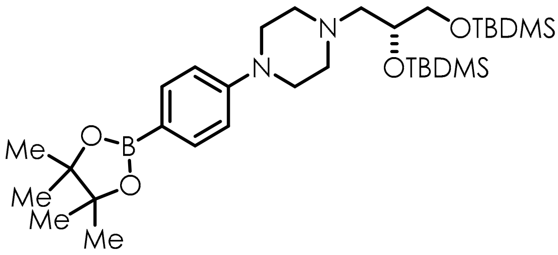

Following general procedure **(B)**, Levodropropizine derivative was obtained as colorless oil in 43% isolated yield from (R)-1-(2,3-bis((*tert*-butyldimethylsilyl) oxy) propyl)-4-phenylpiperazine using 25:1 petroleum ether/EtOAc as eluent.

^{1}H NMR (600 MHz, $CDCl_3$) δ7.70 (d, J = 8.2 Hz, 2H), 6.88 (d, J = 8.2 Hz, 2H), 3.84 – 3.76 (m, 1H), 3.60 (dd, J = 10.1, 5.8 Hz, 1H), 3.53 (dd, J = 10.2, 5.5 Hz, 1H), 3.24 (t, J = 5.0 Hz, 3H), 2.66 (dt, J = 10.6, 5.0 Hz, 2H), 2.62 – 2.54 (m, 2H), 2.49 (dd, J = 13.1, 4.7 Hz, 1H), 2.37 (dd, J = 13.1, 6.0 Hz, 1H), 2.04 (t, J = 12.2 Hz, 1H), 1.32 (s, 12H), 0.89 (d, J = 5.2 Hz, 18H), 0.09 – 0.07 (m, 6H), 0.06 (s, 6H). **^{13}C NMR** (151 MHz, $CDCl_3$) δ 153.66, 136.25, 114.34, 83.48, 72.00, 66.28, 62.12, 54.24, 48.29, 26.10 (d, J = 12.0 Hz), 25.00, -4.74 (dd, J = 127.4, 9.3 Hz) (a signal for the carbon that is attached to the boron atom was not observed). **HRMS** calculated for $C_{31}H_{59}BN_2O_4Si_2$ (M + Na^+): 613.39986, found: 613.39868.

Gemfibrozil derivative A

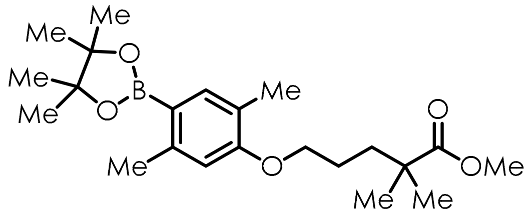

Following general procedure **(B)**, Gemfibrozil derivative A was obtained as colorless liquid in 73% isolated yield from methyl 5-(2,5-dimethylphenoxy)-2,2-dimethylpentanoate using 70:1 petroleum ether/EtOAc as eluent.

^{1}H NMR (600 MHz, $CDCl_3$) δ7.54 (s, 1H), 6.59 (s, 1H), 3.94 (t, J = 5.4 Hz, 2H), 3.67 (s, 3H), 2.51 (s, 3H), 2.17 (s, 3H), 1.72 (dd, J = 4.6, 3.0 Hz, 4H), 1.33 (s, 12H), 1.22 (s, 6H). **^{13}C NMR** (151 MHz, $CDCl_3$) δ 178.41, 159.30, 144.79, 138.43, 122.95, 112.63, 83.15, 67.80, 51.85, 42.21, 37.15, 25.30, 25.24, 24.99, 22.34, 15.55 (a signal for the carbon that is attached to the boron atom was not observed). **^{11}B NMR** (128 MHz, $CDCl_3$) δ 32.03. **HRMS** calculated for $C_{22}H_{35}BO_5$ (M + Na^+): 413.24698, found: 413.24646.

Gemfibrozil derivative B

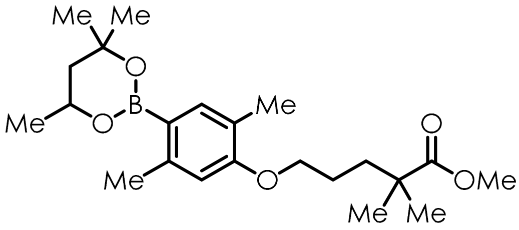

Following general procedure **(D)**, 4,4,4',4',6,6'-hexamethyl-2,2'-bi(1,3,2-dioxaborinane) (203.2 mg, 0.8 mmol, 4.0 eq) was added. Gemfibrozil derivative B was obtained as colorless oil in 51% isolated yield from methyl 5-(2,5-dimethylphenoxy)-2,2-dimethylpentanoate using 70:1 petroleum ether/EtOAc as eluent.

^{1}H NMR (400 MHz, $CDCl_3$) δ 7.50 (s, 1H), 6.55 (s, 1H), 4.32 (dqd, J = 12.2, 6.1, 2.9 Hz, 1H), 3.92 (q, J = 5.2, 4.2 Hz, 2H), 3.66 (s, 3H), 2.48 (s, 3H), 2.17 (s, 3H), 1.84 (dd, J = 13.8, 3.0 Hz, 1H), 1.71 (d, J = 3.0 Hz, 4H), 1.60 – 1.52 (m, 1H), 1.36 (s, 6H), 1.33 (d, J = 6.1 Hz, 3H), 1.21 (s, 6H). **^{13}C NMR** (151 MHz, $CDCl_3$) δ 178.45, 158.53, 143.66, 137.51, 122.71, 113.08, 70.95, 67.90, 65.00, 51.84, 46.19, 42.25, 37.21, 31.59, 28.40, 25.32, 23.49, 22.57, 15.69 (a signal for the carbon that is attached to the boron atom was not observed). **^{11}B NMR** (193 MHz, $CDCl_3$) δ 32.17. **HRMS** calculated for $C_{22}H_{35}BO_5$ (M + H^+): 391.2650, found: 391.2660.

Gemfibrozil derivative C

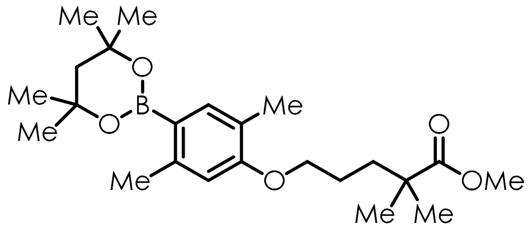

Following general procedure **(B)**, 4,4,4',4',6,6,6',6'-octamethyl-2,2'-bi(1,3,2-dioxaborinane) (225.6 mg, 0.8 mmol, 4.0 eq) was added. Gemfibrozil derivative C was obtained as colorless oil in 40% isolated yield from methyl 5-(2,5-dimethylphenoxy)-2,2-dimethylpentanoate using 70:1 petroleum ether/EtOAc as eluent.

^{1}H NMR (400 MHz, $CDCl_3$) δ 7.50 (s, 1H), 6.56 (s, 1H), 3.93 (t, J = 3.5 Hz, 2H), 3.67 (s, 3H), 2.49 (s, 3H), 2.17 (s, 3H), 1.89 (s, 2H), 1.75 – 1.68 (m, 4H), 1.42 (s, 12H), 1.22 (s, 6H). **^{13}C NMR** (151 MHz, $CDCl_3$) δ 178.47, 158.51, 143.54, 137.51, 122.74, 113.10, 70.81, 67.94, 51.85, 49.08, 42.25, 37.21, 32.08, 25.32, 22.59, 15.70 (a signal for the carbon that is attached to the boron atom was not observed). **^{11}B NMR** (193 MHz, $CDCl_3$) δ 27.03. **HRMS** calculated for $C_{23}H_{37}BO_5$ (M + H^+): 405.2807, found: 405.2819.

Gemfibrozil derivative D

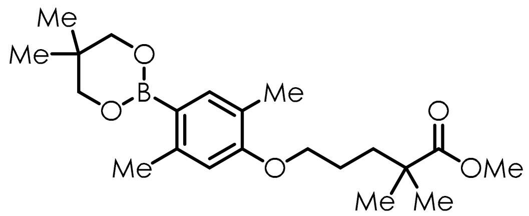

Following general procedure **(B)**, 5,5,5',5'-tetramethyl-2,2'-bi(1,3,2-dioxaborinane) (180.7 mg, 0.8 mmol, 4.0 eq) was added. Gemfibrozil derivative D was obtained as colorless crystal in 52% isolated yield from methyl 5-(2,5-dimethylphenoxy)-2,2-dimethylpentanoate using 70:1 petroleum ether/EtOAc as eluent.

^{1}H NMR (400 MHz, $CDCl_3$) δ 7.51 (s, 1H), 6.56 (s, 1H), 3.96 – 3.91 (m, 2H), 3.75 (s, 4H), 3.66 (s, 3H), 2.48 (s, 3H), 2.17 (s, 3H), 1.71 (d, J = 3.0 Hz, 4H), 1.21 (s, 6H), 1.02 (s, 6H). **^{13}C**

NMR (151 MHz, $CDCl_3$) δ 178.45, 158.75, 143.88, 137.64, 122.80, 113.00, 72.36, 67.86, 51.85, 42.25, 37.22, 31.78, 25.33, 25.31, 22.52, 22.04, 15.6 (a signal for the carbon that is attached to the boron atom was not observed). **^{11}B NMR** (193 MHz, $CDCl_3$) δ 27.47. **HRMS** calculated for $C_{21}H_{33}BO_5$ (M + H^+): 377.2494, found: 377.2494.

4.) Substrates with Low Yield under Optimized Conditions

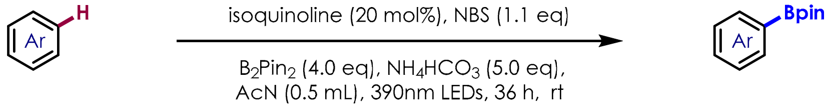

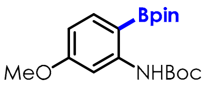

S1, 12%

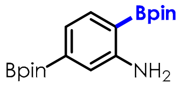

S2, 13%

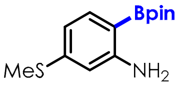

S3, 10%

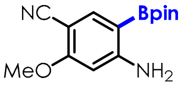

S4, 15%

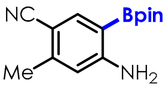

S5, 10%

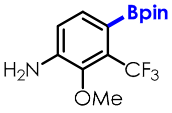

S6, 10%

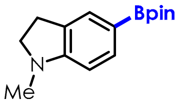

S7, 18%

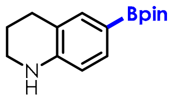

S8, 10%

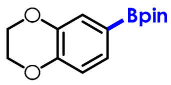

S9, 5%

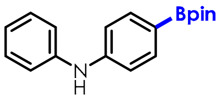

S10, 25%

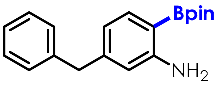

S11, 30%

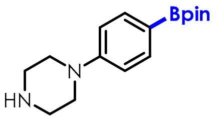

S12, 20%

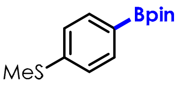

S14, 15%

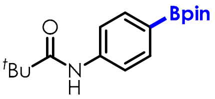

S15, 15%

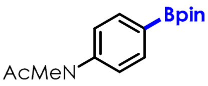

S16, 7%

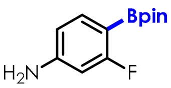

S17, 25%

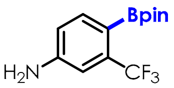

S18, 8%

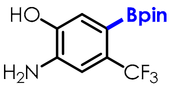

S19, 8%

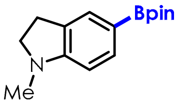

S20, 5%

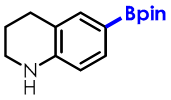

S21, 25%

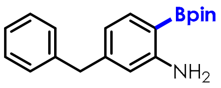

S21, 25%

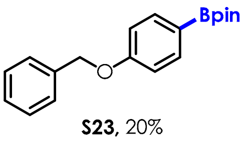

S23, 20%

[a] Reactions were conducted following general procedure **(B)**; yields were determined by ^{1}H NMR using pyrazine or hexamethyl disiloxane as internal standard.

Scheme S2 Substrates with low yield under optimized conditions

5.) Novel Cascade Transformation of Arenes

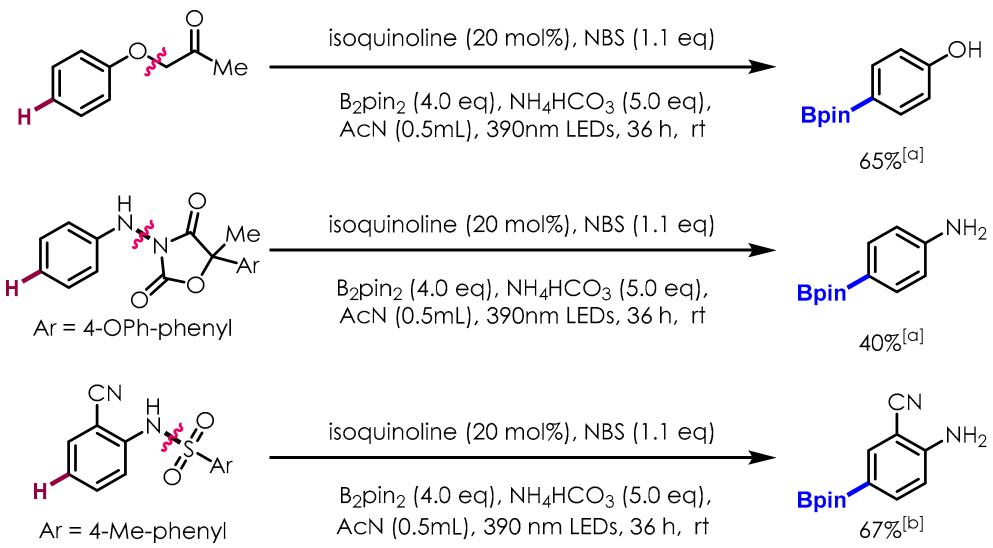

[a] Reactions were conducted following general procedure **(B)**, isolated yield.

Scheme S3 Novel cascade transformation

6.) Mechanistic Study

6.1 Preparation of complex A

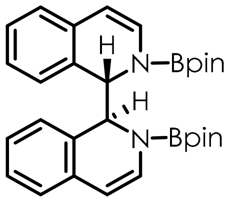

An oven-dried 25 mL round-bottom flask was charged with a magnetic stir bar, and B_2pin_2 (1269.7 mg, 5.0 mmol, 1.0 eq), isoquinoline (1011.9 mg, 10.0 mmol, 2.0 eq) and AcN (5 mL)were added. The tube was sealed with a plastic cap and the reaction mixture was allowed to react at room temperature for 1 h. After filtering the solution and the solid was washed with AcN, the complex A was obtained as white solid.

^{1}H NMR (400 MHz, $CDCl_3$) δ 7.10 (t, J = 7.6 Hz, 2H), 7.00 (d, J = 7.6 Hz, 2H), 6.67 (t, J = 7.5 Hz, 2H), 6.58 (d, J = 7.3 Hz, 2H), 5.87 (t, J = 6.9 Hz, 4H), 4.57 (s, 2H), 1.27 (s, 24H). **^{13}C NMR** (101 MHz, $CDCl_3$) δ 132.41, 131.16, 129.00, 128.78, 127.25, 124.17, 122.92, 106.02, 83.35, 54.24, 25.52, 24.70. The NMR data were inconsistent with the reported data.[29]

6.2 Oxidant screen and control experiments

General procedure (E):

An oven-dried 2 mL vial was charged with a magnetic stir bar, and to the solvent of *N*-methyl-*N*-phenylacetamide (29.8 mg, 0.2 mmol, 1.0 eq) in AcN (0.5 mL), different oxidants (1.1 eq), B_2pin_2 (203.1 mg, 0.8 mmol, 4.0 eq), NH_4HCO_3 (79.1 mg, 1.0 mmol, 5.0 eq), isoquinoline (5.2 mg, 0.04 mmol, 20 mol%) were added. The vial was sealed with a plastic cap and then irradiated with 2*390 nm LEDs light for 72 h. After filtrating the inorganic base, the solvent was removed by vacuum evaporation. NMR yield was determined by ^{1}H NMR using pyrazine or HMDSO as the internal standard. The reaction

mixture was purified by fresh silica-gel chromatography, to afford the desired borylated product.

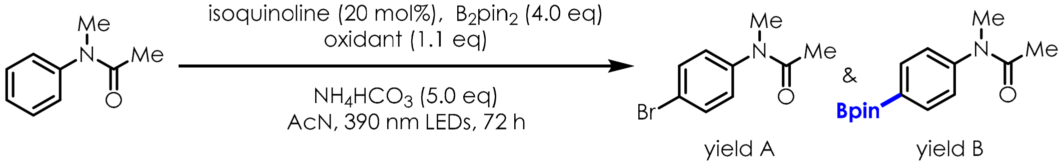

entry	oxidant (1.1 eq)	yield A (%)[a]	yield B (%)[a]
1	Oxone	0	<1
2	Selectfluro	0	<1
3	$(NH_4)_2S_2O_8$	0	<1
4	CBr_4	0	<1
5	Ph_2IOTf	0	<1
6	NCS	0	<2
7	NIS	0	<10
**8**	**NBS**	**<1**	**22**
9	NBS	<1[b]	0[b]

[a] Yields were determined by ^1H NMR using pyrazine or hexamethyl disiloxane as internal standard. [b] Without light.

Table S2 Oxidative additive screen and control experiments

General procedure (F):

An oven-dried 2 mL vial was charged with a magnetic stir bar, and to the solvent of *N*-methyl-*N*-phenylacetamide (29.8 mg, 0.2 mmol, 1.0 eq) in AcN (0.5 mL), NBS (39.2 mg, 0.22 mmol, 1.1 eq), complex **A** (102.5 mg, 0.2 mmol, 1.0 eq), and suitable B_2pin_2, NH_4HCO_3 (79.1 mg, 1.0 mmol, 5.0 eq) were added. The vial was sealed with a plastic cap and then irradiated with a 2*390 nm LEDs light for 72 h. After filtrating the inorganic base, the solvent was removed by vacuum evaporation. NMR yield was determined by ^1H NMR using pyrazine or HMDSO as the internal standard. The reaction mixture was purified by fresh silica-gel chromatography, to afford the desired borylated product.

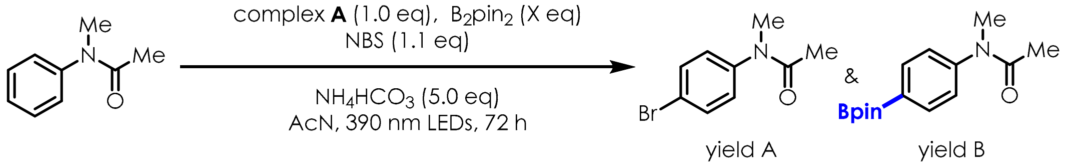

entry	B_2pin_2(X eq)	yield A (%)^a	yield B (%)^a
1	0.0	<1	0
2	2.0	<1	10
3	3.0	<1	23

[a] Yields were determined by ^{1}H NMR using pyrazine or hexamethyl disiloxane as internal standard.

Table S3 Reaction with complex **A**

N-methyl-N-(4-(4,4,5,5-tetramethyl-1,3,2-dioxaborolan-2-yl) phenyl) acetamide (49)

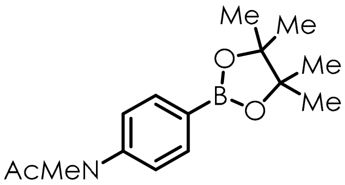

Following general procedure **(C)**, **49** was obtained as white solid using 4:1 petroleum ether/EtOAc as eluent.

^{1}H NMR (400 MHz, $CDCl_3$) δ 7.85 (d, J = 8.0 Hz, 2H), 7.19 (d, J = 8.0 Hz, 2H), 3.27 (s, 3H), 1.88 (s, 3H), 1.35 (s, 12H). The NMR data were inconsistent with the reported data.[30]

6.3 Computational Experiment

All DFT calculations were performed using Gaussian 16 software. Geometry optimization and frequency calculation of ground state were carried out by the M062X[31]/def2SVP[32] level with Grimme's D3 dispersion correction[33] in PCM[34]-CH_3CN solvent. Using the optimized geometry of ground states, the excited states were calculated by the dependent density functional theory (TD-DFT) calculations[35] using the same theoretical level.

The first excited states of transient EDA complex and complex A + substrate, as well as the corresponding oscillator strength *f* and excitation energies, are reported. The corresponding molecular orbitals are depicted below. Compared with complex A + substrate, one more charge-transfer transition from complex B (complex B = complex A + Base) to substrate (MO 193 to MO 196) is observed in the transient EDA complex, indicating the base (HCO_3^-) can help make the substrate more active when the complex is excited by light.

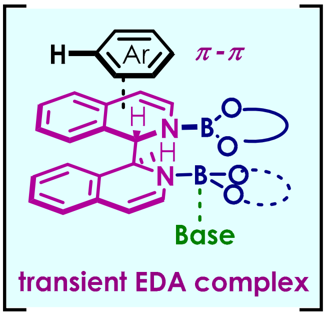

Excitation energies and oscillator strengths:

Excited State 1: 4.0548 eV 305.77 nm f=0.5574

192 ->196 0.19610

193 ->196 0.12890

MO 192

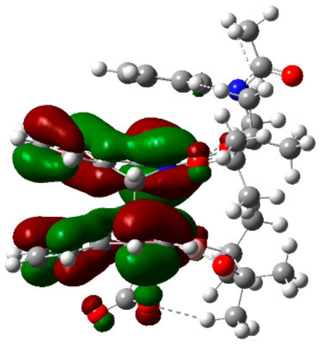

MO193

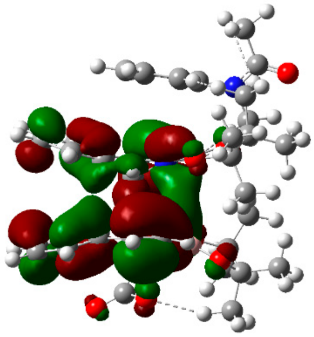

MO196

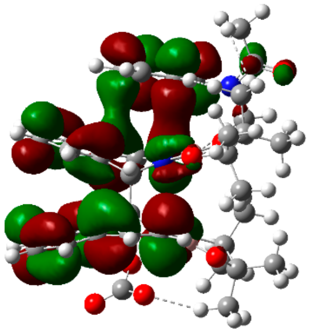

Figure S1 The computational results with Base.

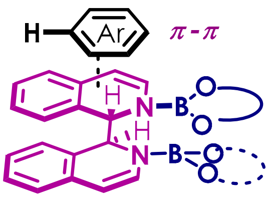

Excitation energies and oscillator strengths:

Excited State 1: 4.1747 eV 296.99 nm f=0.4040

176 ->180 0.17743

MO 176 MO180

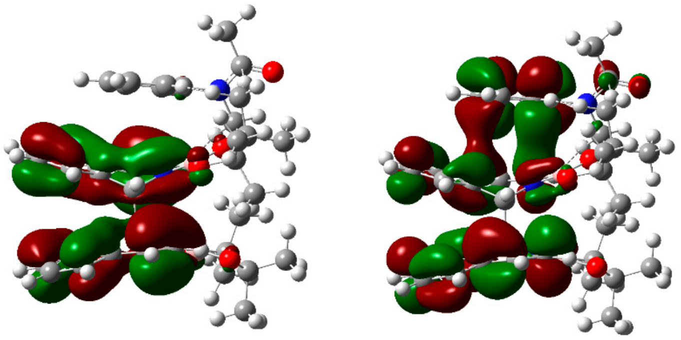

Figure S2 The computational results without Base.

7.) X-ray Crystallographic Data

Single-Crystal X-ray Diffraction Data for 22($C_{13}H_{20}BNO_3$)

Experimental

Single crystals of **22**($C_{13}H_{20}BNO_3$) were obtained by slow evaporation of a mixture of hexane/dichloromethane at room temperature. A suitable crystal was selected and detected on a SuperNova, Dual, Cu at home/near, AtlasS2 diffractometer. The crystal was kept at 297.99(10) K during data collection. Using Olex2 [36], the structure was solved with the ShelXT [37] structure solution program using Intrinsic Phasing and refined with the ShelXL [38] refinement package using least-squares minimization.

Crystal-structure determination of 22($C_{13}H_{20}BNO_3$)

Crystal data for **22**($C_{13}H_{20}BNO_3$) (*M* =249.11 g/mol):
orthorhombic, space group $P2_12_12_1$ (no. 19), *a* = 7.8703(5) Å, *b* = 9.8070(8) Å, *c* = 17.9336(13) Å, *V* = 1384.19(17) Å^3, *Z* = 4, *T* = 297.99(10) K, μ(CuKα) = 0.667 mm^{-1}, *Dcalc* = 1.195 g/cm^3, 5819 reflections measured (9.864° ≤ 2Θ ≤ 146.69°), 2681 unique (R_{int} = 0.0313, R_{sigma} = 0.0375), which were used in all calculations. The final R_1 was 0.0420 (I > 2σ(I)) and wR_2 was 0.1182 (all data).

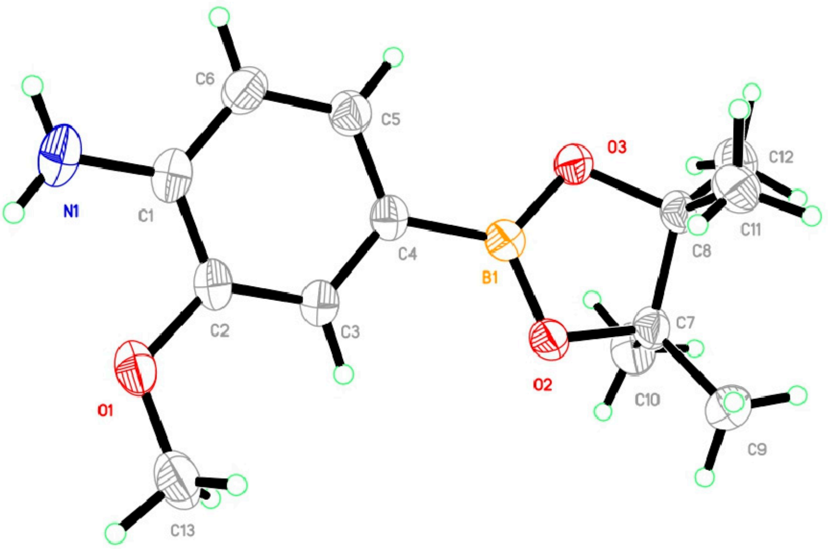

Crystal Data for **22**($C_{13}H_{20}BNO_3$): **CCDC 2247881**

Identification code	22
Empirical formula	$C_{13}H_{20}BNO_3$
Formula weight	249.11
Temperature/K	297.99(10)
Crystal system	orthorhombic
Space group	$P2_12_12_1$
a/Å	7.8703(5)
b/Å	9.8070(8)
c/Å	17.9336(13)
α/°	90
β/°	90
γ/°	90
Volume/Å^3	1384.19(17)
Z	4
ρ_{calc}g/cm^3	1.195
μ/mm^{-1}	0.667
F(000)	536
Crystal size/mm^3	0.3 × 0.2 × 0.12
Radiation	CuKα (λ = 1.54184)
2Θ range for data collection/°	9.864 to 146.69
Index ranges	-9 ≤ h ≤ 5, -11 ≤ k ≤ 11, -22 ≤ l ≤ 18
Reflections collected	5819
Independent reflections	2681 [R_{int} = 0.0313, R_{sigma} = 0.0375]
Data/restraints/parameters	2681/0/169
Goodness-of-fit on F^2	1.074

Final R indexes [I>=2σ (I)]	R_1 = 0.0420, wR_2 = 0.1116
Final R indexes [all data]	R_1 = 0.0486, wR_2 = 0.1182
Largest diff. peak/hole / e Å^{-3}	0.15/-0.15
Flack parameter	-0.34(19)

Table S4 Crystal data and structure refinement for **22**

Single-Crystal X-ray Diffraction Data for 34($C_{15}H_{23}BO_4$)

Single crystals of **34**($C_{15}H_{23}BO_4$) were obtained by slow evaporation of a mixture of hexane/dichloromethane at room temperature.

Crystal-structure determination of 34($C_{15}H_{23}BO_4$)

Crystal data for **34**($C_{15}H_{23}BO_4$) (*M* =278.14 g/mol):
monoclinic, space group P2$_1$/n (no. 14), *a* = 10.3129(5) Å, *b* = 7.3918(3) Å, *c* = 20.7542(8) Å, β = 91.830(4)°, *V* = 1581.30(12) Å^3, *Z* = 4, *T* = 297.99(10) K, μ(CuKα) = 0.663 mm^{-1}, *Dcalc* = 1.168 g/cm^3, 10331 reflections measured (8.526° ≤ 2Θ ≤ 146.172°), 3126 unique (R_{int} = 0.0482, R_{sigma} = 0.0376), which were used in all calculations. The final R_1 was 0.0580 (I > 2σ(I)) and wR_2 was 0.1753 (all data).

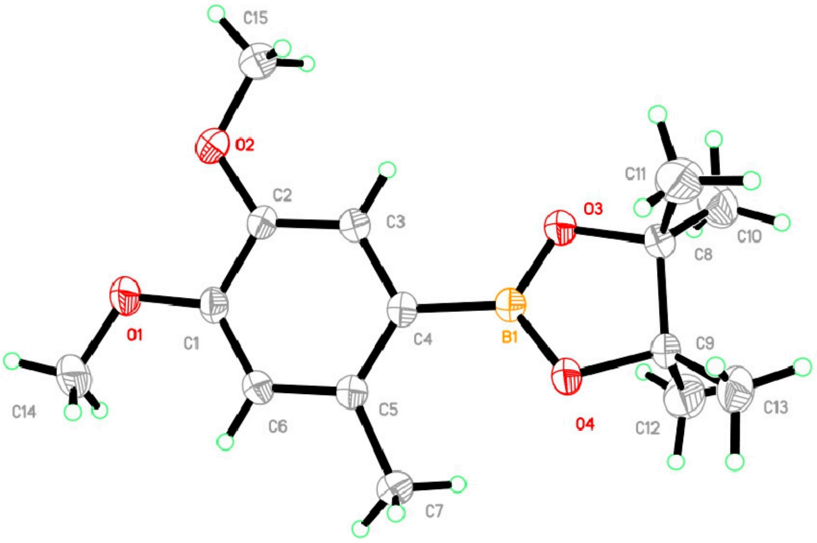

Crystal Data for **34**($C_{15}H_{23}BO_4$): **CCDC 2247883**

Identification code	34
Empirical formula	$C_{15}H_{23}BO_4$
Formula weight	278.14
Temperature/K	297.99(10)
Crystal system	monoclinic
Space group	P2$_1$/n
a/Å	10.3129(5)
b/Å	7.3918(3)
c/Å	20.7542(8)
α/°	90
β/°	91.830(4)
γ/°	90

Volume/Å^3	1581.30(12)
Z	4
ρ_{calc}g/cm^3	1.168
μ/mm^{-1}	0.663
F(000)	600
Crystal size/mm^3	0.300 × 0.200 × 0.120
Radiation	CuKα (λ = 1.54184)
2Θ range for data collection/°	8.526 to 146.172
Index ranges	-11 ≤ h ≤ 12, -9 ≤ k ≤ 9, -25 ≤ l ≤ 22
Reflections collected	10331
Independent reflections	3126 [R_{int} = 0.0482, R_{sigma} = 0.0376]
Data/restraints/parameters	3126/0/189
Goodness-of-fit on F^2	1.048
Final R indexes [I>=2σ (I)]	R_1 = 0.0580, wR_2 = 0.1640
Final R indexes [all data]	R_1 = 0.0650, wR_2 = 0.1753
Largest diff. peak/hole / e Å^{-3}	0.27/-0.22

Table S5 Crystal data and structure refinement for **34**

Single-Crystal X-ray Diffraction Data for 35($C_{14}H_{19}BF_3NO_3$)

Single crystals of **35**($C_{14}H_{19}BF_3NO_3$) were obtained by slow evaporation of a mixture of hexane/dichloromethane at room temperature.

Crystal-structure determination of 35($C_{14}H_{19}BF_3NO_3$)

Crystal data for **35**($C_{14}H_{19}BF_3NO_3$) (*M* =317.11 g/mol):
monoclinic, space group P2$_1$ (no. 4), *a* = 9.1846(3) Å, *b* = 7.21653(17) Å, *c* = 12.3685(3) Å, *β* = 109.976(3)°, *V* = 770.47(4) Å^3, *Z* = 2, *T* = 149.99(10) K, μ(CuKα) = 1.010 mm^{-1}, *Dcalc* = 1.367 g/cm^3, 5000 reflections measured (7.606° ≤ 2Θ ≤ 145.392°), 2942 unique (R_{int} = 0.0242, R_{sigma} = 0.0281). which were used in all calculations. The final R_1 was 0.0325 (I > 2σ(I)) and wR_2 was 0.0906 (all data).

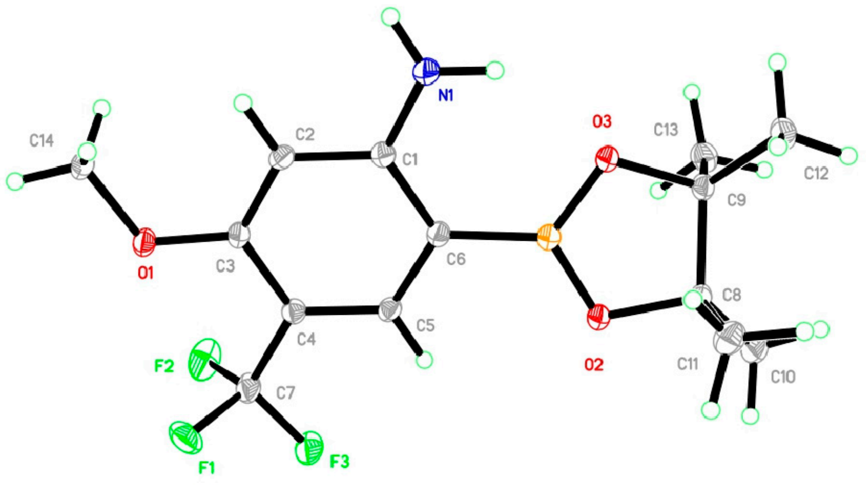

Crystal Data for **35**($C_{14}H_{19}BF_3NO_3$): **CCDC 2247894**

Identification code	35
Empirical formula	$C_{14}H_{19}BF_3NO_3$
Formula weight	317.11
Temperature/K	149.99(10)
Crystal system	monoclinic
Space group	$P2_1$
a/Å	9.1846(3)
b/Å	7.21653(17)
c/Å	12.3685(3)
α/°	90
β/°	109.976(3)
γ/°	90
Volume/Å^3	770.47(4)
Z	2
ρ_{calc}g/cm^3	1.367
μ/mm^{-1}	1.01
F(000)	332
Crystal size/mm^3	0.23 × 0.12 × 0.08
Radiation	CuKα (λ = 1.54184)
2Θ range for data collection/°	7.606 to 145.392
Index ranges	-11 ≤ h ≤ 10, -8 ≤ k ≤ 8, -15 ≤ l ≤ 15
Reflections collected	5000
Independent reflections	2942 [R_{int} = 0.0242, R_{sigma} = 0.0281]
Data/restraints/parameters	2942/1/212
Goodness-of-fit on F^2	1.045
Final R indexes [I>=2σ (I)]	R_1 = 0.0325, wR_2 = 0.0896
Final R indexes [all data]	R_1 = 0.0332, wR_2 = 0.0906
Largest diff. peak/hole / e Å^{-3}	0.20/-0.28
Flack parameter	0.44(6)

Table S6 Crystal data and structure refinement for **35**

Single-Crystal X-ray Diffraction Data for Gemfibrozil derivative D ($C_{21}H_{33}BO_5$)

Single crystals of **Gemfibrozil derivative D** ($C_{21}H_{33}BO_5$) were obtained by slow evaporation of a mixture of hexane/dichloromethane at room temperature.

Crystal-structure determination of Gemfibrozil derivative D ($C_{21}H_{33}BO_5$)

Crystal data for **Gemfibrozil derivative D** ($C_{21}H_{33}BO_5$) (*M* =376.28 g/mol):
monoclinic, space group $P2_1/c$ (no. 14), *a* = 12.87350(19) Å, *b* = 9.89691(13) Å, *c* = 16.8202(2) Å, *β* = 94.9717(13)°, *V* = 2134.96(5) Å^3, *Z* = 4, *T* = 149.99(10) K, μ(CuKα) = 0.650 mm^{-1}, *Dcalc* = 1.171 g/cm^3, 7874 reflections measured (6.892° ≤ 2Θ ≤ 145.826°), 4144 unique (R_{int} = 0.0209, R_{sigma} = 0.0244,) which were used in all calculations. The final R_1 was 0.0400 (I > 2σ(I)) and wR_2 was 0.1167 (all data).

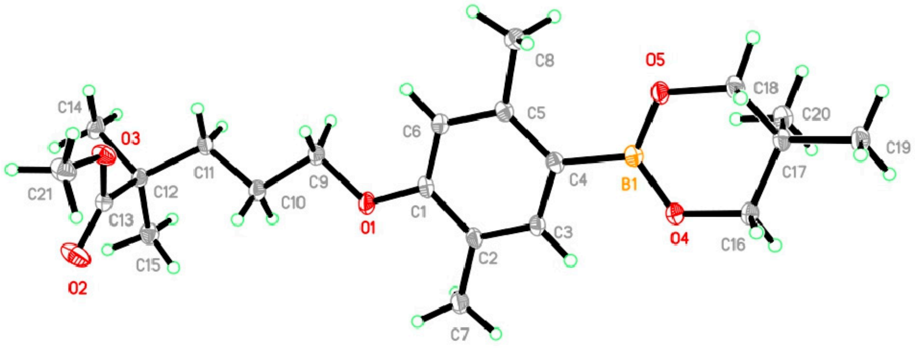

Crystal Data for **Gemfibrozil derivative D** ($C_{21}H_{33}BO_5$): **CCDC 2247895**

Identification code	Gemfibrozil derivative D
Empirical formula	$C_{21}H_{33}BO_5$
Formula weight	376.28
Temperature/K	149.99(10)
Crystal system	monoclinic
Space group	$P2_1/c$
a/Å	12.87350(19)
b/Å	9.89691(13)
c/Å	16.8202(2)
α/°	90
β/°	94.9717(13)
γ/°	90
Volume/Å^3	2134.96(5)
Z	4
ρ_{calc}g/cm^3	1.171
μ/mm^{-1}	0.65
F(000)	816
Crystal size/mm^3	0.28 × 0.18 × 0.12
Radiation	CuKα (λ = 1.54184)
2Θ range for data collection/°	6.892 to 145.826
Index ranges	-14 ≤ h ≤ 15, -12 ≤ k ≤ 8, -20 ≤ l ≤ 20
Reflections collected	7874
Independent reflections	4144 [R_{int} = 0.0209, R_{sigma} = 0.0244]
Data/restraints/parameters	4144/0/252
Goodness-of-fit on F^2	1.019
Final R indexes [I>=2σ (I)]	R_1 = 0.0400, wR_2 = 0.1123
Final R indexes [all data]	R_1 = 0.0435, wR_2 = 0.1167
Largest diff. peak/hole / e Å^{-3}	0.34/-0.19

Table S7 Crystal data and structure refinement for **Gemfibrozil derivative D**

8.) References

For experiments:

[1] S.-F. Jin, H. T. Dang, G. C. Haug, R. He, V. D. Nguyen, V.T. Nguyen, H. D. Arman, K. S. Schanze, O. V. Larionov, Visible Light-Induced Borylation of C–O, C–N, and C–X Bonds. *J. Am. Chem. Soc.* **2020,** 142, 1603-1613.

[2] K. Chen, M. S. Cheung, Z.-Y. Lin, P.-F Li, Metal-free borylation of electron-rich aryl (pseudo)halides under continuous-flow photolytic conditions. *Org. Chem. Front.* **2016**, 3, 875-879.

[3] J. B. McManus, D. A. Nicewicz, Direct C–H Cyanation of Arenes via Organic Photoredox Catalysis. *J Am Chem Soc*. **2017**, 139, 2880-2883.

[4] Y. Uetake, T. Niwa, T. Hosoya, Rhodium-Catalyzed ipso-Borylation of Alkylthioarenes via C–S Bond Cleavage. *Org. Lett.* **2016**, 18**,** 2758-2761.

[5] R. Mir, T. Dudding, Phase-Transfer Catalyzed O-Silyl Ether Deprotection Mediated by a Cyclopropenium Cation. *J. Org. Chem.* **2017**, 82, 709-714.

[6] A. Nitelet, D., Thevenet, B. Schiavi, C. Hardouin, J. Fournier, R. Tamion, X. Pannecoucke, P. Jubault, T. Poisson. Copper-Photocatalyzed Borylation of Organic Halides under Batch and Continuous-Flow Conditions. *Chem. -Eur. J.* **2019**, 25, 3262-3266.

[7] Y.-X. Gong, Z.-D. Zhu, Q. Qian, W.-Q. Tong, H.-G. Gong, Miyaura Borylation and One-Pot Two-Step Homocoupling of Aryl Chlorides and Bromides under Solvent-Free Conditions. *Org. Lett.* **2021**, 23, 1005-1010.

[8] P. B. Dzhevakov, M. A. Topchiy, D. A. Zharkova, O. S. Morozov, A. F. Asachenko, M. S Nechaev, Miyaura Borylation and One-Pot Two-Step Homocoupling of Aryl Chlorides and Bromides under Solvent-Free Conditions. *Adv. Synth. Catal*. **2016**, 358, 977-983.

[9] D. Wei, O. Sadek, V. Dorcet, T. Roisnel, C. Darcel, E. Gras, E. Clot, J.-B. Sortais, Selective mono N-methylation of anilines with methanol catalyzed by rhenium complexes: An experimental and theoretical study. *J. Catal.* **2018**, *366*, 300-309.

[10] J. D. Firth, L. A. Hammarback, T. J. Burden, J. B. Eastwood, J. R. Donald, C. S. Horbaczewskyj, T. McRobie, A. Tramaseur, I. P. Clark, M. Towrie, A. Robinson, J. P. Krieger, J. M. Lynam, I. J. S. Fairlamb, Light- and Manganese-Initiated Borylation of Aryl Diazonium Salts: Mechanistic Insight on the Ultrafast Time-Scale Revealed by Time-Resolved Spectroscopic Analysis. *Chem. Eur. J.* **2020**, 27, 3979-3985.

[11] T. Yamamoto, T. Morita, J. Takagi, T. Yamakawa, NiCl2(PMe3)2-Catalyzed Borylation of Aryl Chlorides. *Org. Lett*. **2011**, *12*, 5766-5769.

[12] S. Gisbertz, S. Reischauer, B. Pieber, Overcoming limitations in dual photoredox/nickel-catalysed C–N cross-couplings due to catalyst deactivation. *Nat. Catal.* **2020**, 3, 611-620.

[13] H. D. S. Guerrand, L. D. Marciasini, M. Jousseaume, M. Vaultier, M. Pucheault, Borylation of Unactivated Aryl Chlorides under Mild Conditions by Using Diisopropylaminoborane as a Borylating Reagent. *Chem. Eur. J.* **2014**, 20, 5573-5579.

[14] J. Dong, H. Guo, Q.-S. Hu, Room Temperature Ni0/PCy3-Catalyzed Coupling Reactions of Aryl Arenesulfonates with Bis(pinacolato)diboron. *Eur. J. Org. Chem.* **2017**, 2017, 7087-7090.

[15] M. J. Waring, A. M. Birch, S. Birtles, L. K. Buckett, R. J. Butlin, L. Campbell, P. M. Gutierrez, P. D. Kemmitt, A. G. Leach, P. A. MacFaul, C. O'Donnell, A. V. Turnbull,

Optimisation of biphenyl acetic acid inhibitors of diacylglycerol acetyl transferase 1 – the discovery of AZD2353. *Med. Chem. Commun*. **2013**, 4, 159-164.

[16] S. M. Preshlock, D. L. Plattner, P. E. Maligres, S. W. Krska, R. E. Maleczka J, M. R. Smith **III**, A Traceless Directing Group for C H Borylation. *Angew. Chem. Int. Ed.* **2013,** 52, 12915-12919.

[17] D. Qiu, S. Wang, S.-B. Tang, H. Meng, L. Jin, F.-Y. Mo, Y. Zhang, J.-B. Wang, Synthesis of Trimethylstannyl Arylboronate Compounds by Sandmeyer-Type Transformations and Their Applications in Chemoselective Cross-Coupling Reactions. *J. Org. Chem.* **2014**, 79, 1979-1988.

[18] M. T. Mihai, B. D. Williams, R. J. Phipps, Para-Selective C–H Borylation of Common Arene Building Blocks Enabled by Ion-Pairing with a Bulky Countercation. *J. Am. Chem. Soc.* **2019**, 141, 15477–15482.

[19] N. J. Taylor, E. Emer, S. Preshlock, M. Schedler, M. Tredwell, S. Verhoog, J. Mercier, C. Genicot, V. Gouverneur, Derisking the Cu-Mediated ^{18}F-Fluorination of Heterocyclic Positron Emission Tomography Radioligands. *J. Am. Chem. Soc.* **2017**, 139, 8267–8276.

[20] C. Kong, N. Jana, C. Jones, T. G. Driver, Control of the Chemoselectivity of Metal N-Aryl Nitrene Reactivity: C–H Bond Amination versus Electrocyclization. *J. Am. Chem. Soc.* **2016**, 138, 13271-13280.

[21] S. R. Inglis, E. C. Y. Woon, A. L. Thompson, C. J. Schofield, Observations on the Deprotection of Pinanediol and Pinacol Boronate Esters via Fluorinated Intermediates. *J. Org. Chem.* **2010**, 75, 468-471.

[22] J. W. Clary, T. J. Rettenmaier, R. Snelling, W. Bryks, J. Banwell, W. T. Wipke, B. Singaram, Hydride as a Leaving Group in the Reaction of Pinacolborane with Halides under Ambient Grignard and Barbier Conditions. One-Pot Synthesis of Alkyl, Aryl, Heteroaryl, Vinyl, and Allyl Pinacolboronic Esters. *J. Org, Chem.* **2011**, 76, 9602-9610.

[23] A. Zernickel, W.-Y. Du, S. A. Ghorpade, D. N. Sawant, A. A. Makki, N. Sekar, J. Eppinger, Bedford-Type Palladacycle-Catalyzed Miyaura Borylation of Aryl Halides with Tetrahydroxydiboron in Water. *J. Org. Chem.* **2018**, 83, 1842-1851.

[24] A. R. Davalos, E. Sylvester, S. T. Diver, Macrocyclic N-Heterocyclic Carbenes: Synthesis and Catalytic Applications. *Organometallics.* **2019**, 38, 2338-2346.

[25] T. M. Chapman, S. A. Osborne, C. Wallace, K. Birchall, N. Bouloc, H. M. Jones, K. H. Ansell, D. L. Taylor, B. Clough, J. L. Green, A. A. Holder, Optimization of an Imidazopyridazine Series of Inhibitors of Plasmodium falciparum Calcium-Dependent Protein Kinase 1 (PfCDPK1). *J. Med. Chem.* **2014**, 57, 3570–3587.

[26] H.-R. L, I. Kevlishvili, X. Yu, P. Liu, G.-B. Dong, Boron insertion into alkyl ether bonds via zinc/nickel tandem catalysis. *Science* **2021**, 372, 175-182.

[27] Q. Yin, H. F. T. Klare, M, Oestreich, Catalytic Friedel–Crafts C−H Borylation of Electron-Rich Arenes: Dramatic Rate Acceleration by Added Alkenes. *Angew. Chem. Int. Ed.* **2017**, 56, 3712-3717.

[28] Y.-C. Lv, M.-Y. Li, T. Liu, L.-J. Tong, T. Peng, L.-X. Wei, J. Ding, H. Xie, W.-H, Duan, Discovery of a New Series of Naphthamides as Potent VEGFR-2 Kinase Inhibitors. *ACS Med. Chem. Lett.* **2014**, 5, 592–597.

[29] D.-P. Chen, G.-Q. Xu, Q.-H. Zhou, L.-W. Chung, W.-J Tang, Practical and Asymmetric Reductive Coupling of Isoquinolines Templated by Chiral Diborons. *J Am Chem Soc.*,

2017, **139**, 9767-9770.
[30] Y. Uetake, T. Niwa, T. Hosoya, Rhodium-Catalyzed ipso-Borylation of Alkylthioarenes via C–S Bond Cleavage. *Org. Lett.* **2016**, 18, 2758–2761.
[31] Y. Zhao, D. G. Truhlar, The M06 suite of density functionals for main group thermochemistry, thermochemical kinetics, noncovalent interactions, excited states, and transition elements: two new functionals and systematic testing of four M06-class functionals and 12 other functionals. *Theor. Chem. Acc.* **2008,** *120*, 215-241.
[32] F. Weigend, R. Ahlrichs, Balanced basis sets of split valence, triple zeta valence and quadruple zeta valence quality for H to Rn: Design and assessment of accuracy. *Phys. Chem. Chem. Phys.* **2005,** *7*, 3297-3305.
[33] T. Clark, J. Chandrasekhar, G. W. Spitznagel, P. V. Schleyer, Efficient diffuse function-augmented basis sets for anion calculations. III.† The 3-21+G basis set for first-row elements, Li–F. *J. Comput. Chem.* **1983**, *4*, 294-301.
[34] J. Tomasi, B. Mennucci, R. Cammi, Quantum Mechanical Continuum Solvation Models. *Chem. Rev.* **2005,** *105*, 2999-3094.
[35] C. Adamo, D. Jacquemin, The calculations of excited-state properties with Time-Dependent Density Functional Theory. *Chem. Soc. Rev.* **2013,** *42*, 845-856.
[36] O. V. Dolomanov, L. J. Bourhis, R. J, Gildea, J. A. K. Howard, H. J. Puschmann, OLEX2: a complete structure solution, refinement and analysis program. *J. Appl. Cryst*. **2009**, 42, 339-341.
[37] G.M. Sheldrick. SHELXT – Integrated space-group and crystalstructure determination. *Acta Cryst*. **2015**, A71, 3-8.
[38] G.M. Sheldrick. Crystal structure refinement with SHELXL. *Acta Cryst*. **2015**, C71, 3-8.

9.) NMR Spectroscopic Data

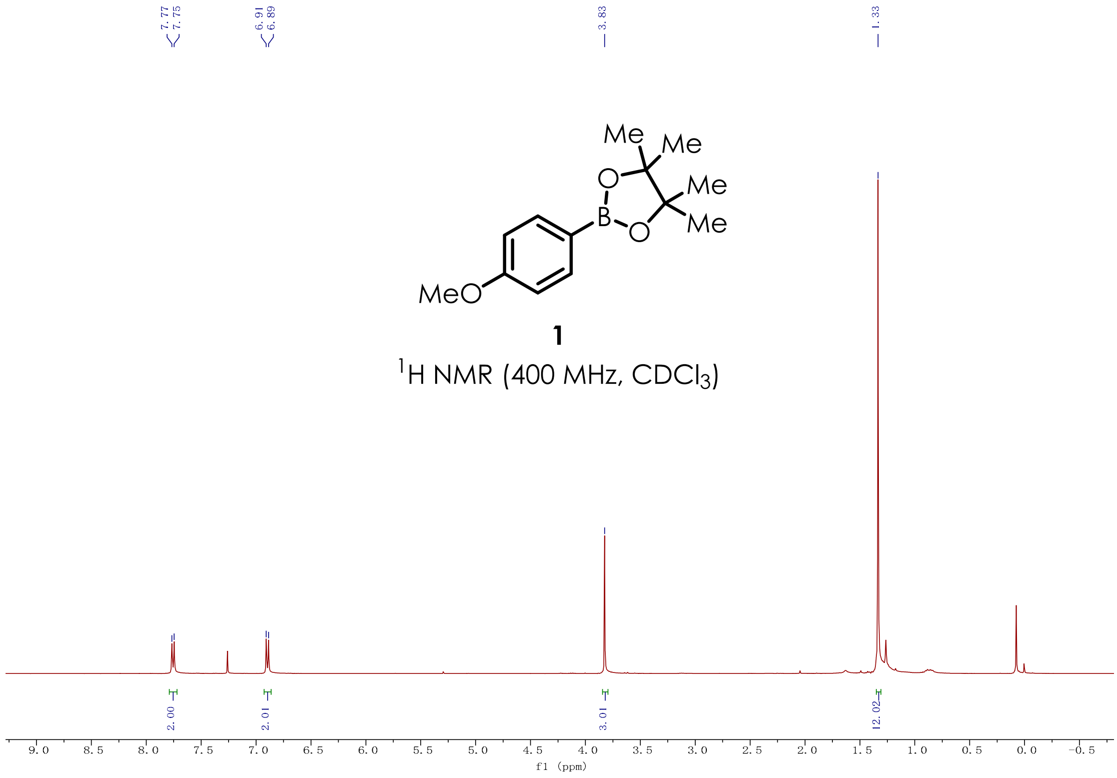

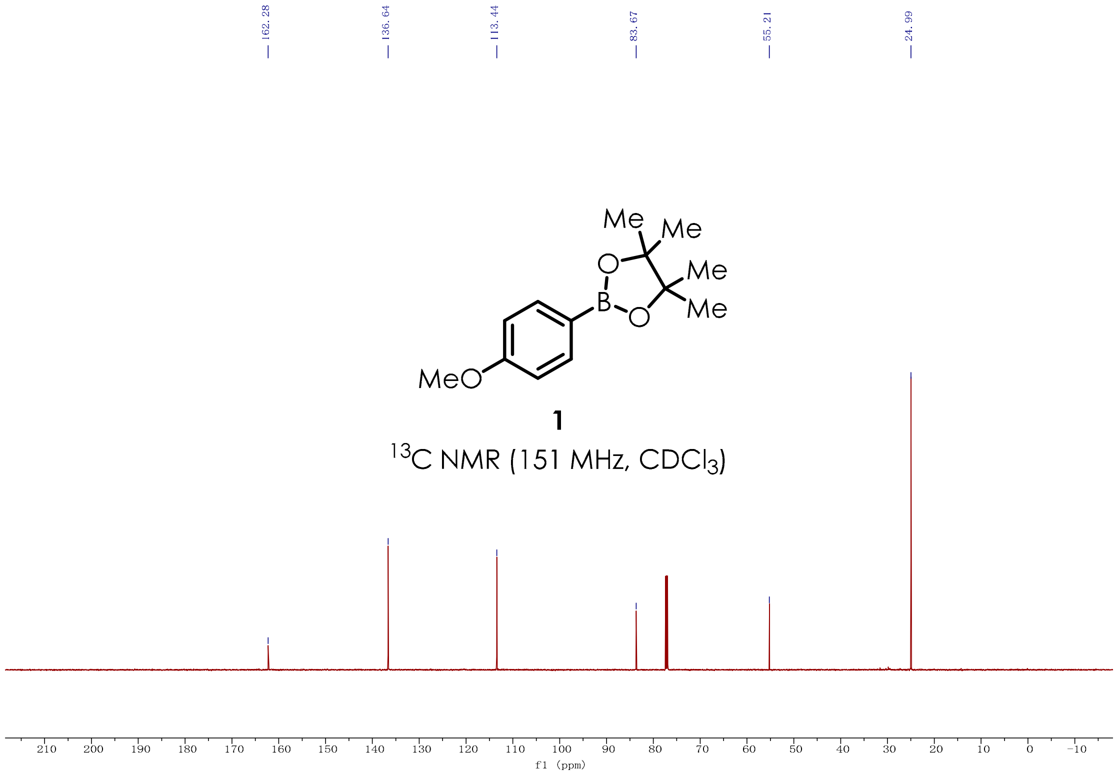

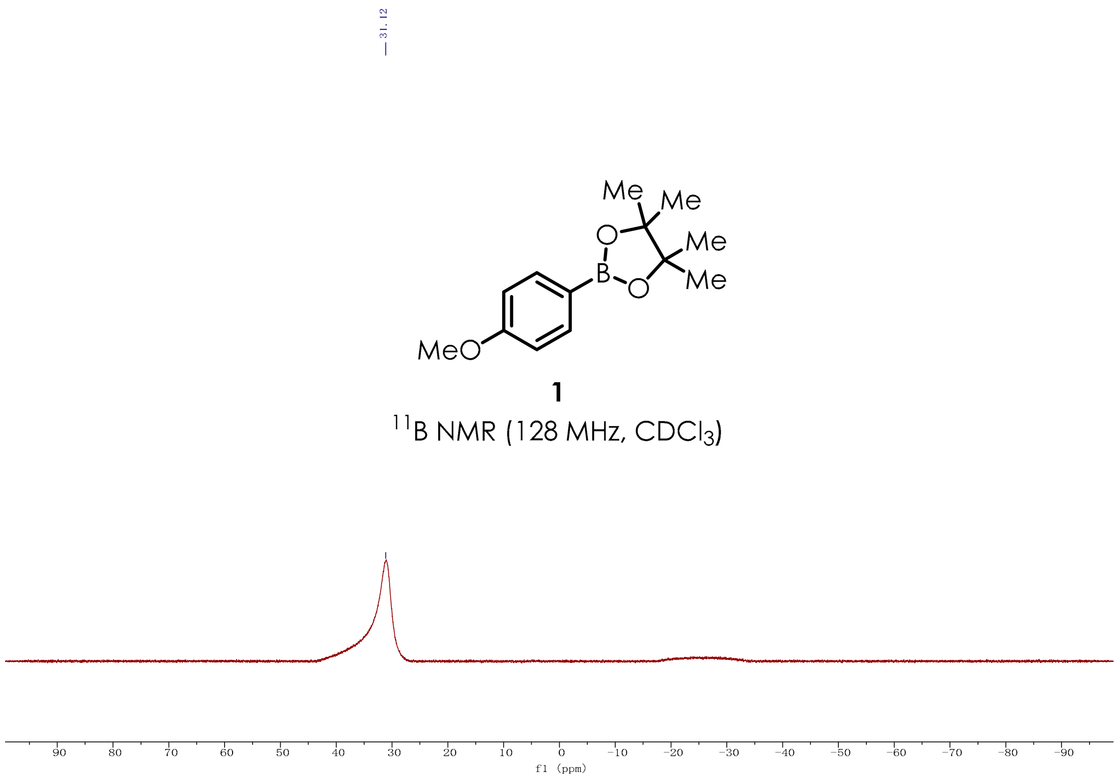

1

^{11}B NMR (128 MHz, $CDCl_3$)

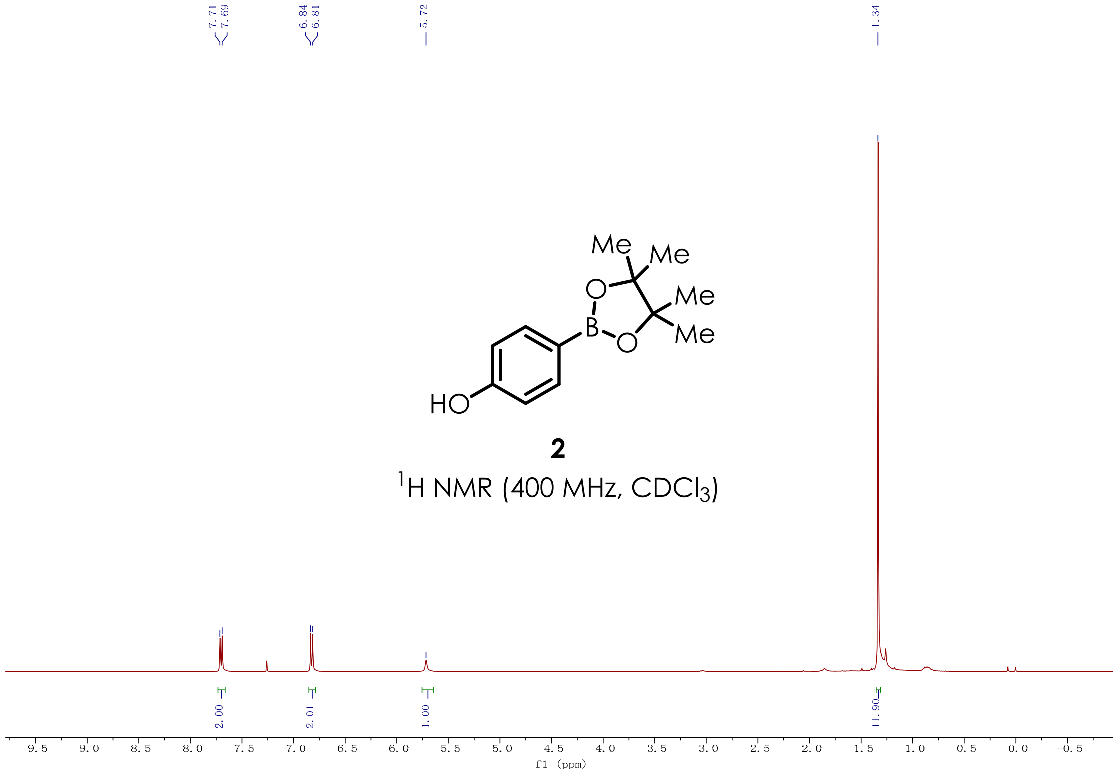

2

^{1}H NMR (400 MHz, $CDCl_3$)

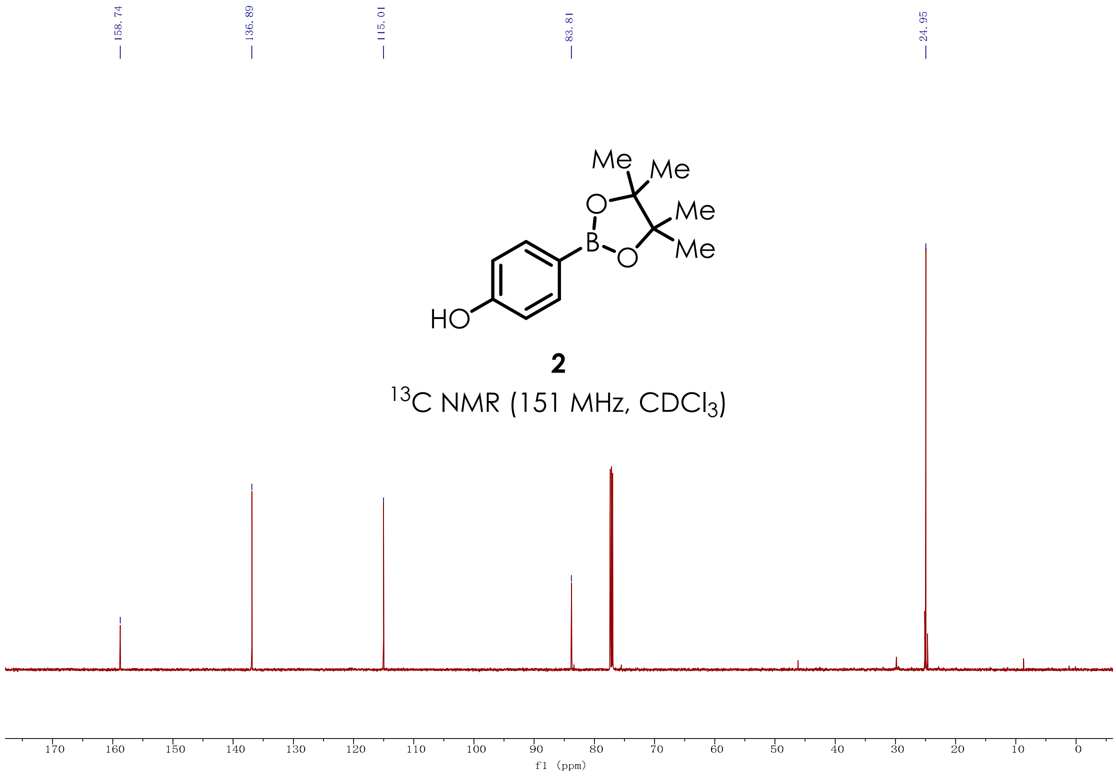

2

^{13}C NMR (151 MHz, $CDCl_3$)

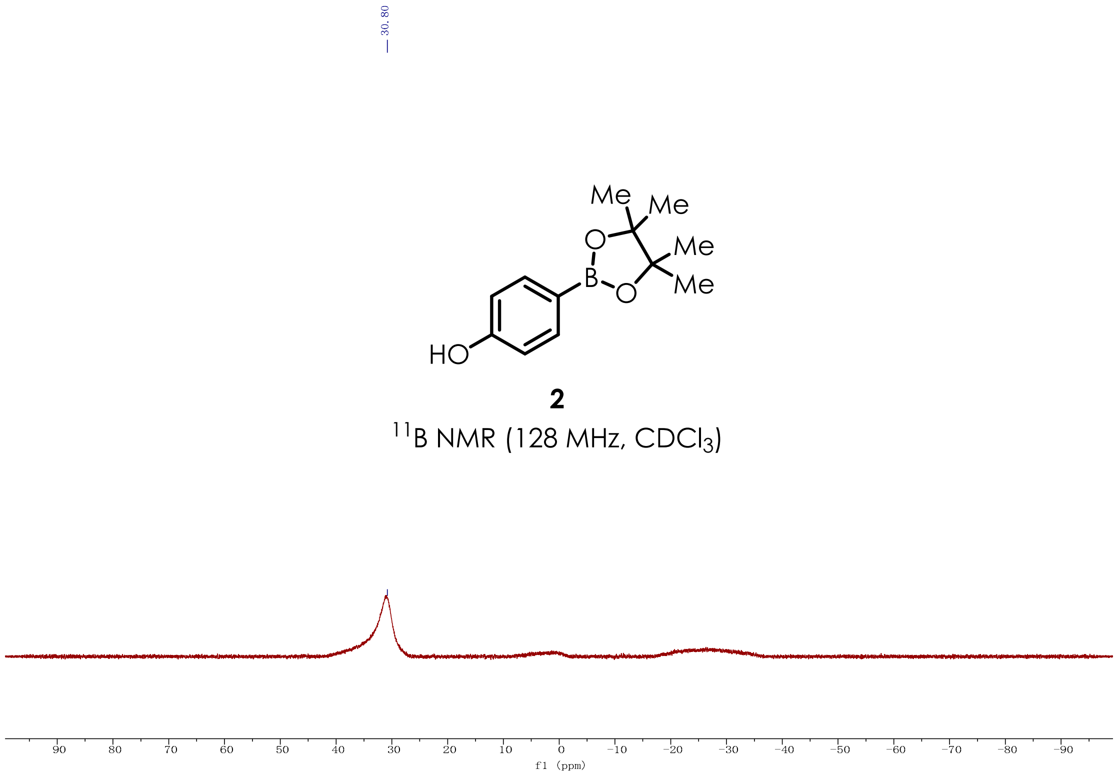

2

^{11}B NMR (128 MHz, $CDCl_3$)

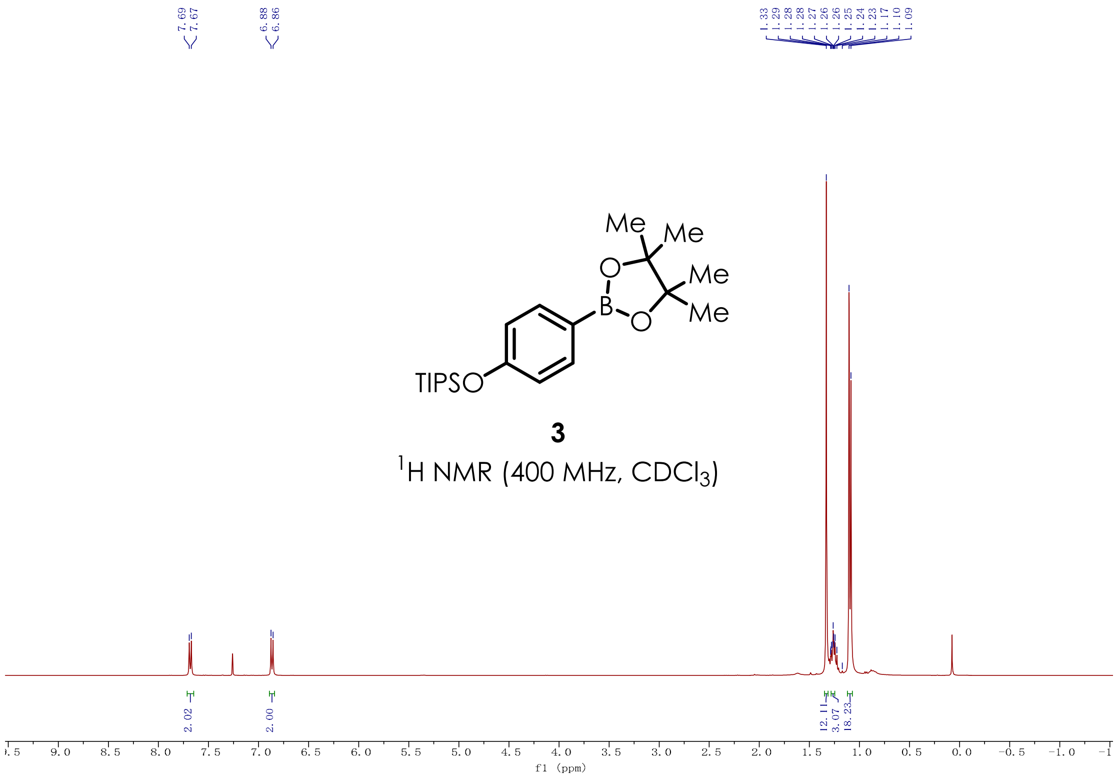

7.69
7.67
6.88
6.86
1.33
1.29
1.28
1.28
1.27
1.26
1.26
1.25
1.24
1.23
1.17
1.10
1.09
Me
Me
Me
Me
O
B
O
TIPSO
3
^{1}H NMR (400 MHz, CDCl$_3$)
2.02
2.00
12.11
3.07
18.23
f1 (ppm)

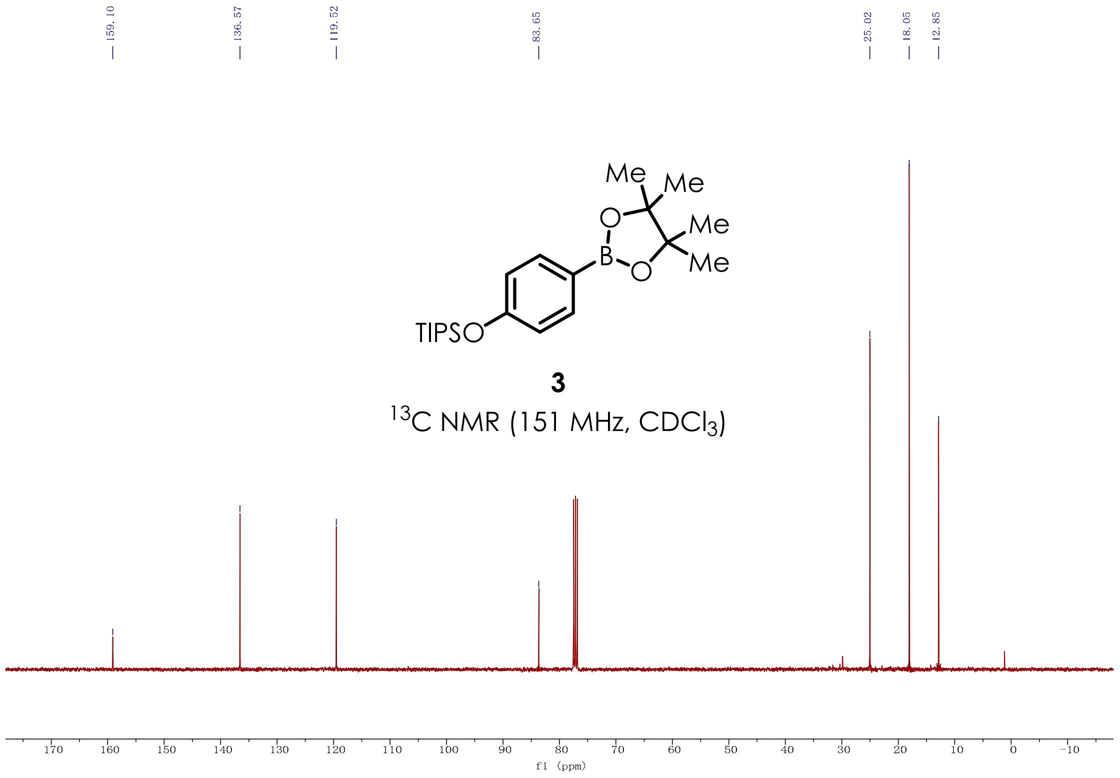

159.10
136.57
119.52
83.65
25.02
18.05
12.85
Me
Me
Me
Me
O
B
O
TIPSO
3
^{13}C NMR (151 MHz, CDCl$_3$)
f1 (ppm)

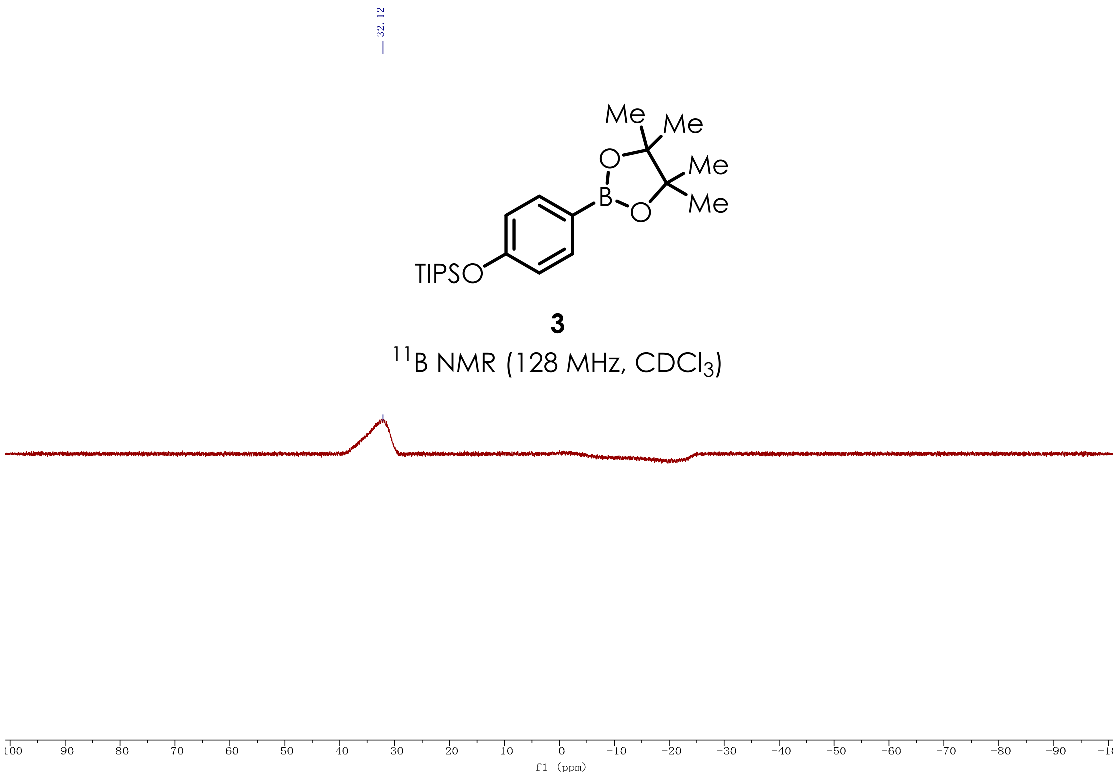
32.12
Me
Me
Me
Me
O
B
O
TIPSO
3
^{11}B NMR (128 MHz, $CDCl_3$)
f1 (ppm)

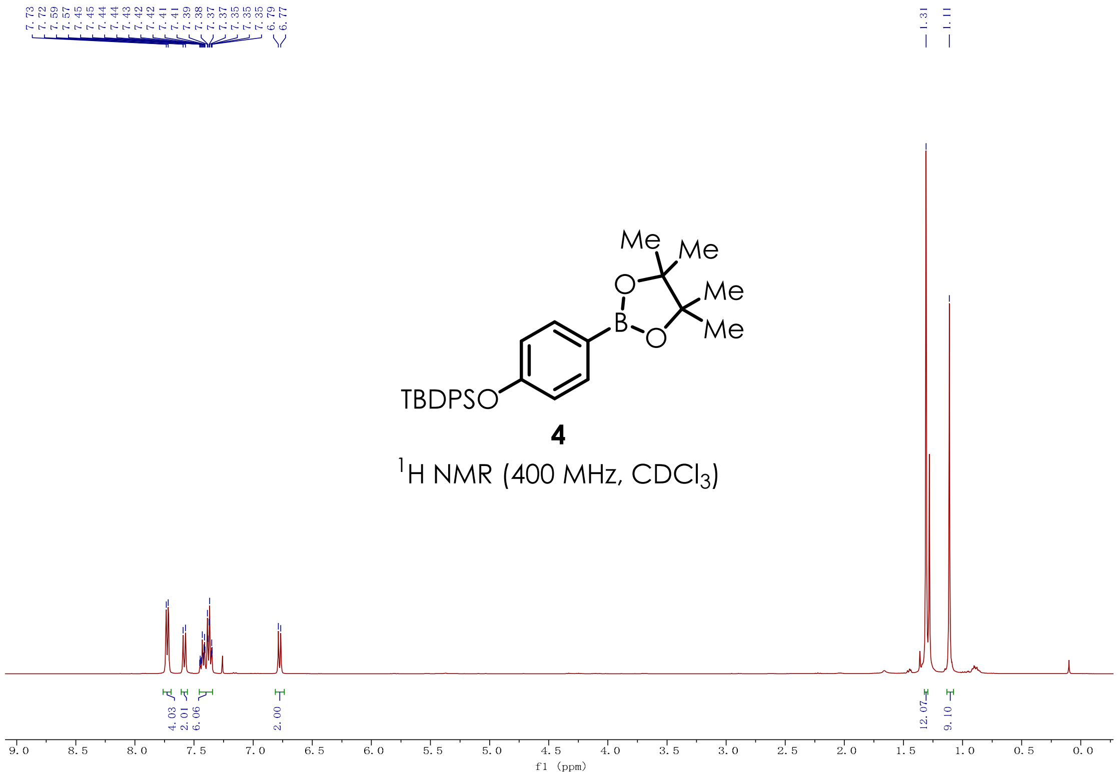
7.73 7.72 7.59 7.57 7.45 7.45 7.44 7.44 7.43 7.42 7.42 7.41 7.41 7.39 7.38 7.37 7.37 7.35 7.35 6.79 6.77
1.31
1.11
Me
Me
Me
Me
O
B
O
TBDPSO
4
^{1}H NMR (400 MHz, $CDCl_3$)
4.03
2.01
6.06
2.00
12.07
9.10
f1 (ppm)

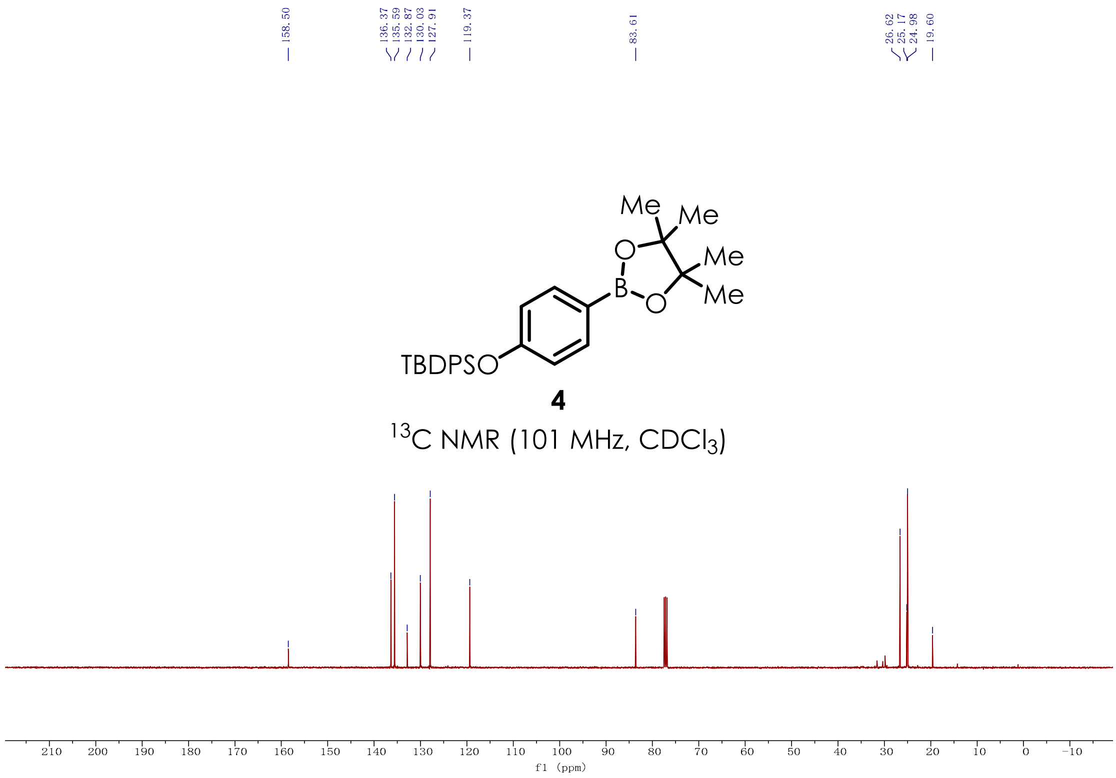
158.50
136.37
135.59
132.87
130.03
127.91
119.37
83.61
26.62
25.17
24.98
19.60
Me
Me
Me
Me
O
B
O
TBDPSO
4
13C NMR (101 MHz, CDCl3)
f1 (ppm)

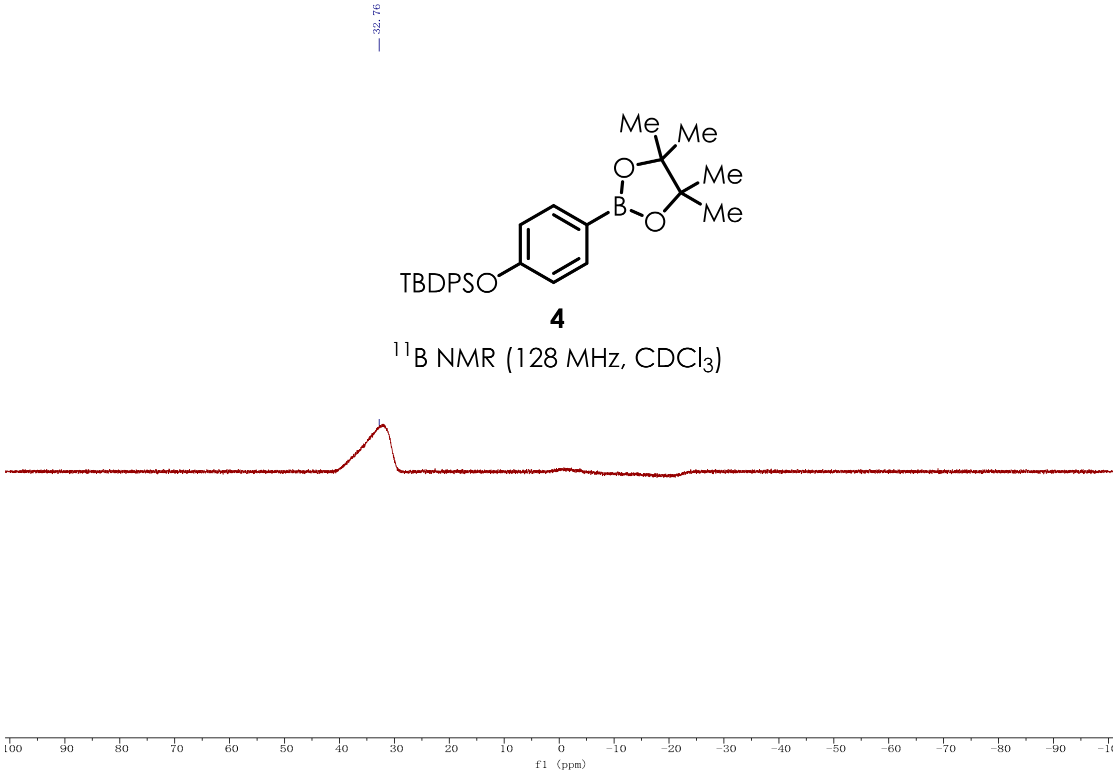
32.76
Me
Me
Me
Me
O
B
O
TBDPSO
4
11B NMR (128 MHz, CDCl3)
f1 (ppm)

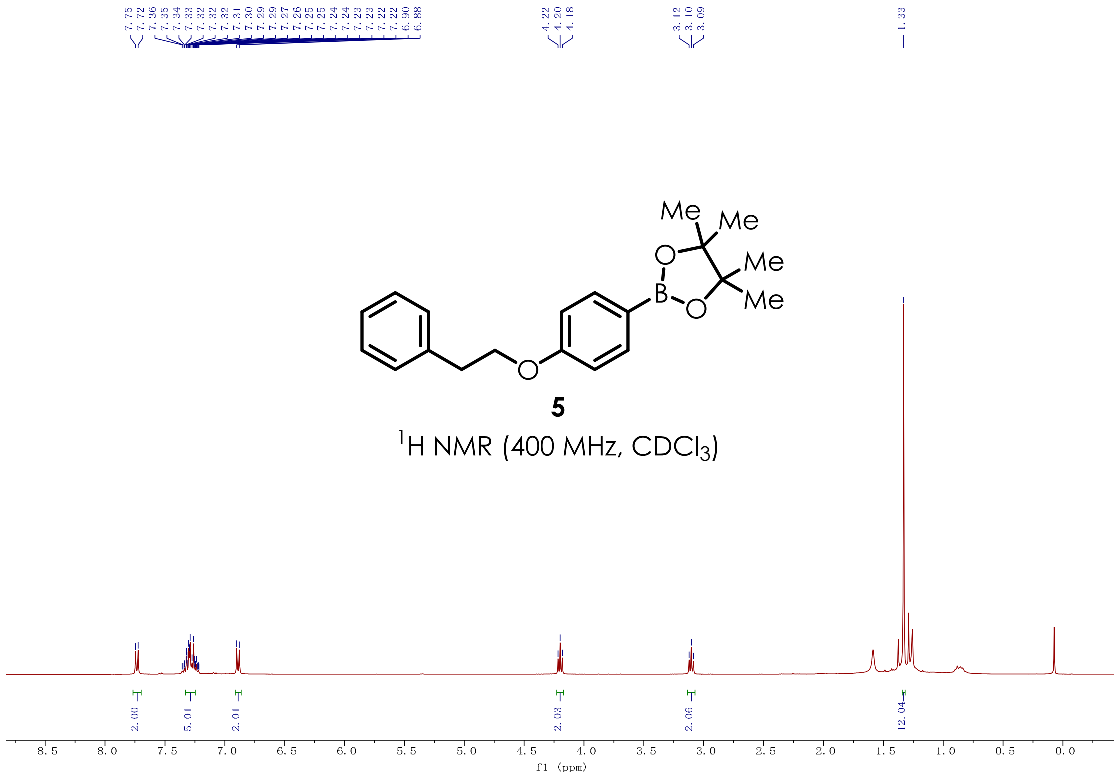

7.75
7.72
7.36
7.35
7.34
7.33
7.32
7.32
7.32
7.31
7.30
7.29
7.29
7.27
7.26
7.25
7.25
7.24
7.24
7.23
7.23
7.22
7.22
6.90
6.88
4.22
4.20
4.18
3.12
3.10
3.09
1.33
Me
Me
Me
Me
O
B
O
O
5
1H NMR (400 MHz, CDCl3)
2.00
5.01
2.01
2.03
2.06
12.04
f1 (ppm)

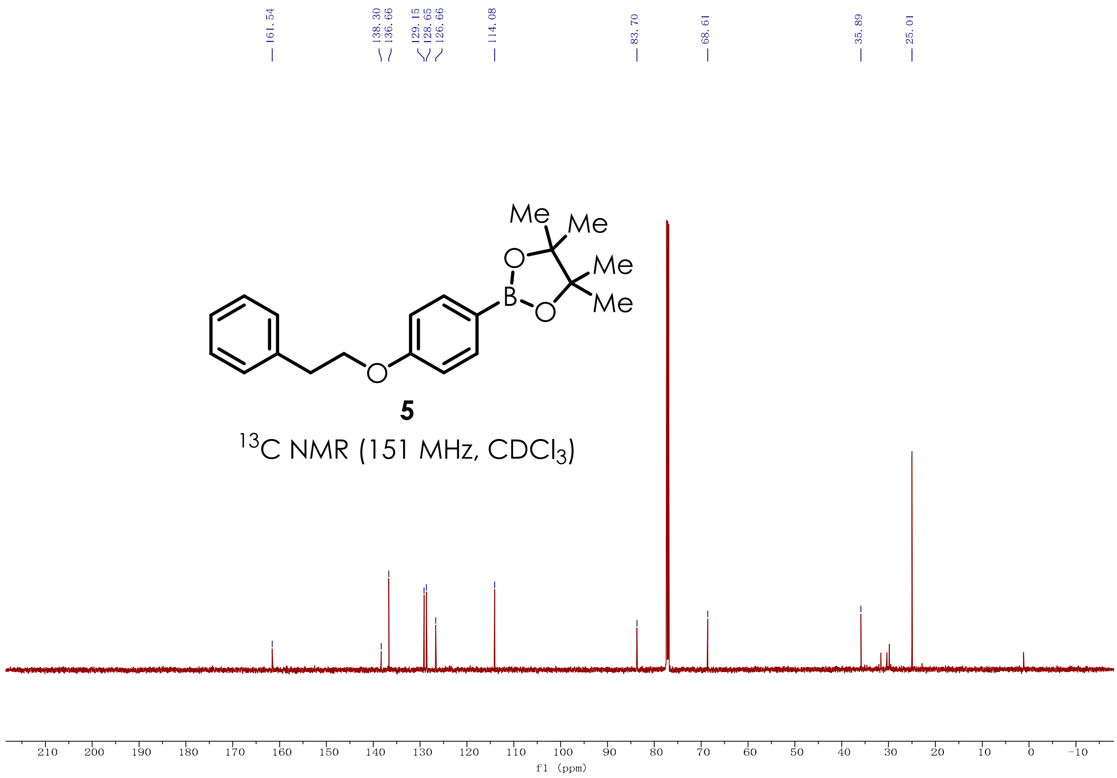

161.54
138.30
136.66
129.15
128.65
126.66
114.08
83.70
68.61
35.89
25.01
Me
Me
Me
Me
O
B
O
O
5
13C NMR (151 MHz, CDCl3)
f1 (ppm)

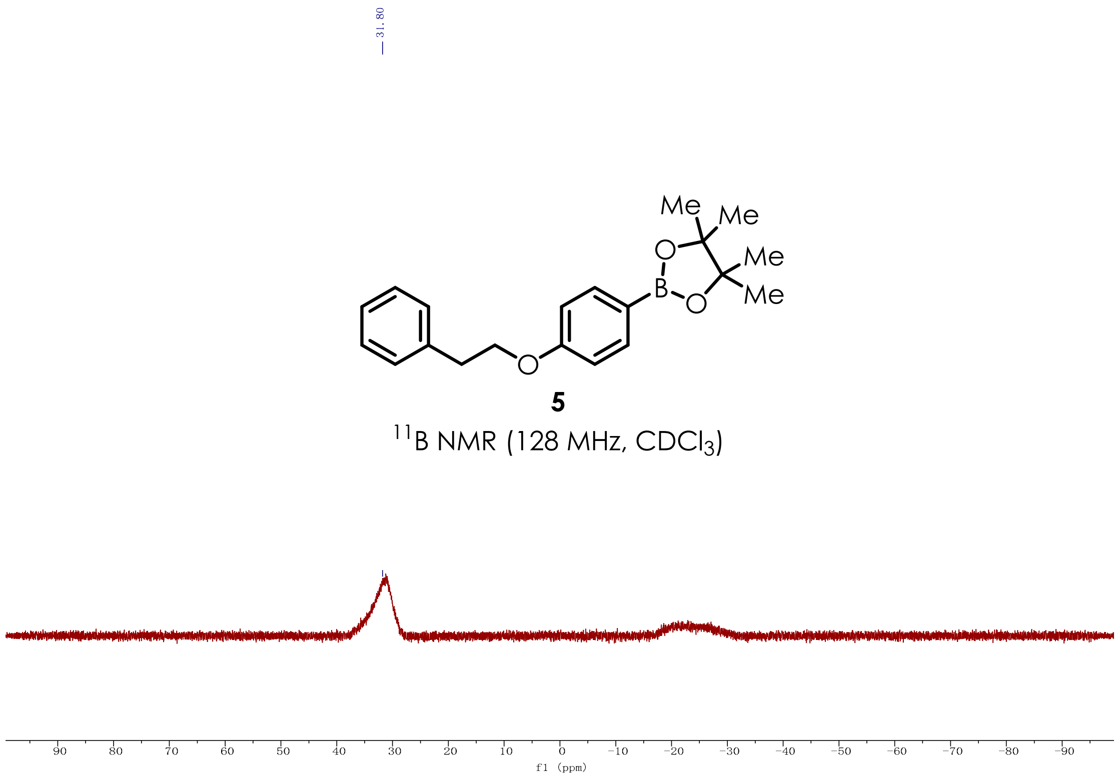

5

^{11}B NMR (128 MHz, $CDCl_3$)

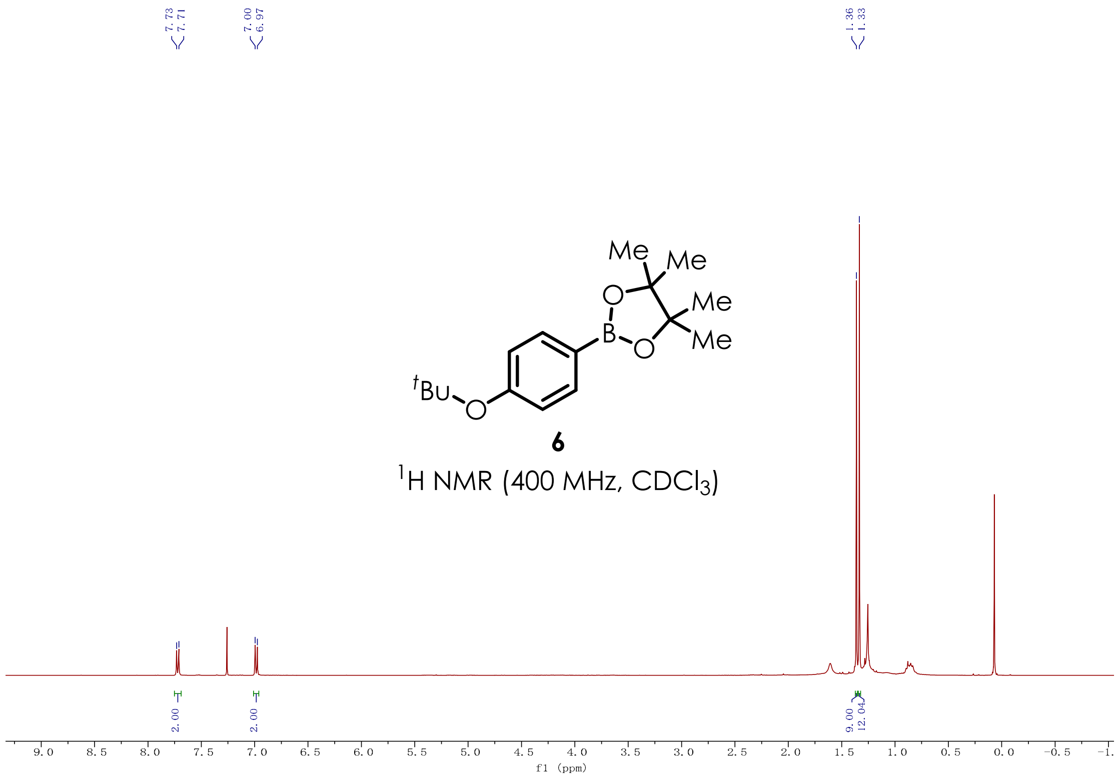

6

^{1}H NMR (400 MHz, $CDCl_3$)

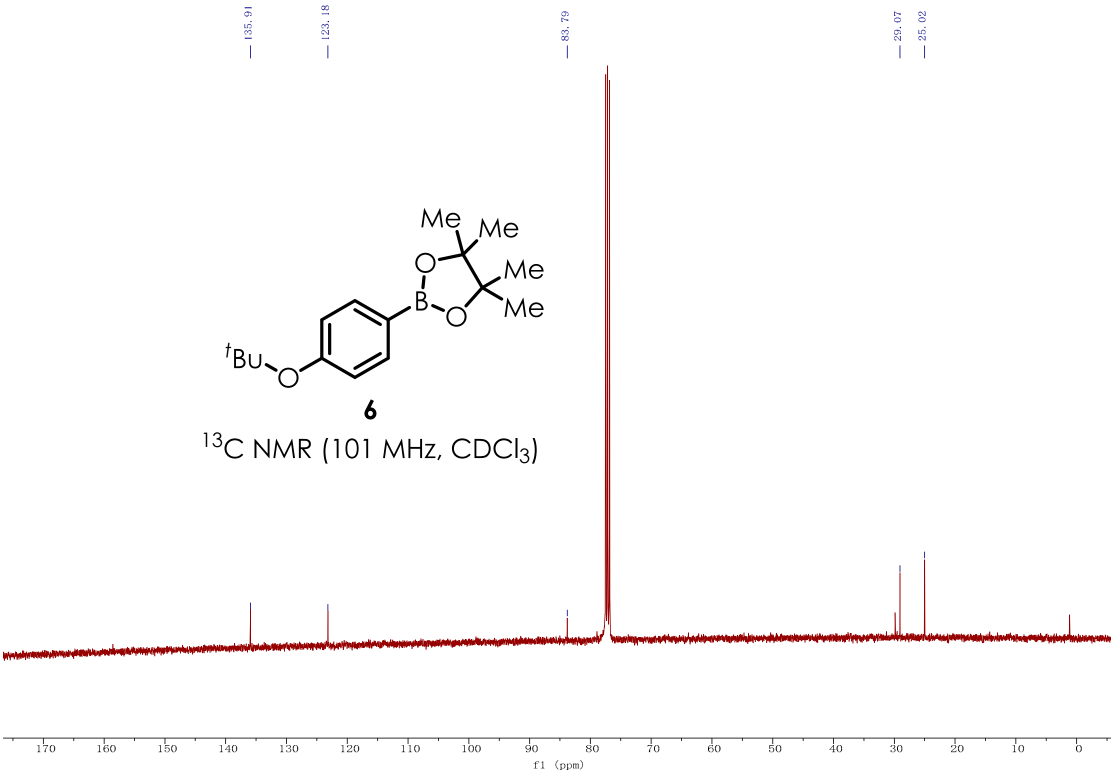

6

^{13}C NMR (101 MHz, $CDCl_3$)

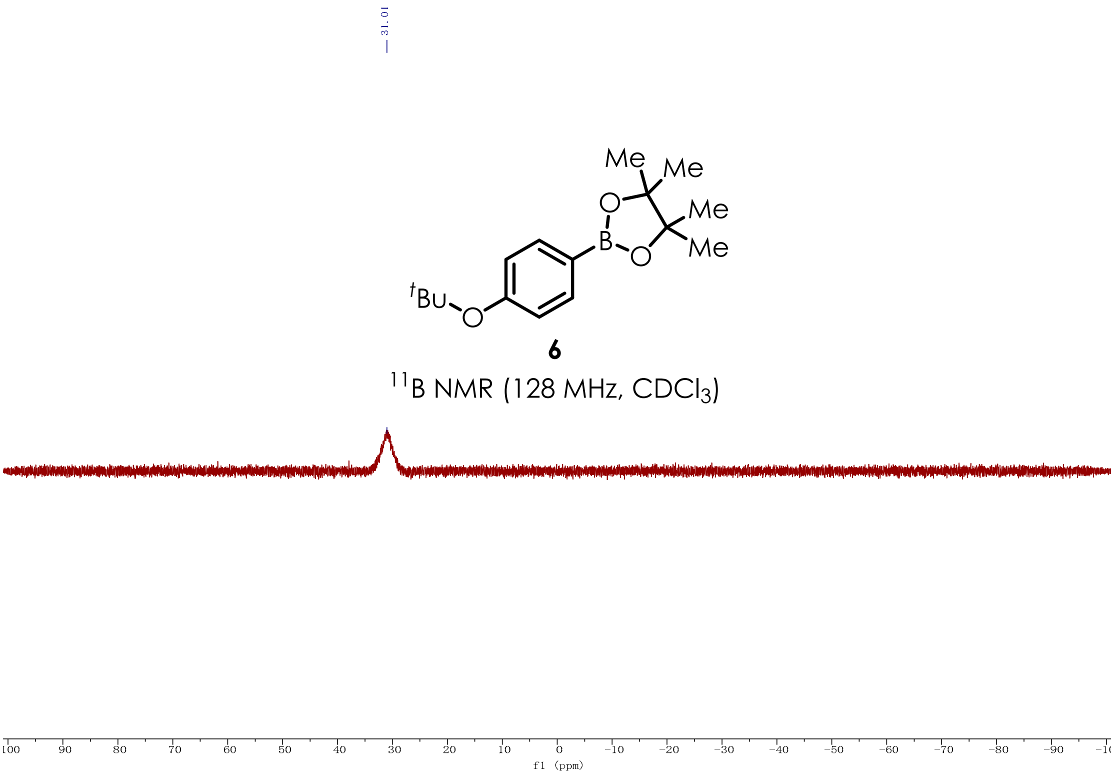

6

^{11}B NMR (128 MHz, $CDCl_3$)

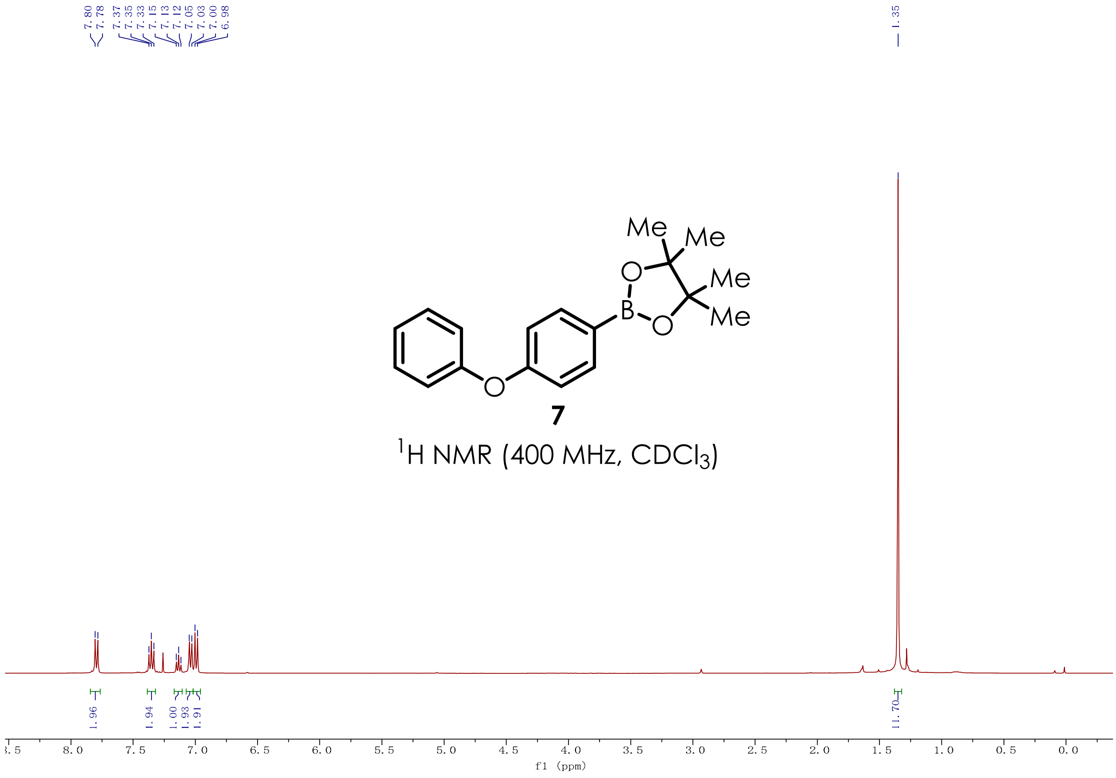

7.80
7.78
7.37
7.35
7.33
7.15
7.13
7.12
7.05
7.03
7.00
6.98
1.35
Me
Me
Me
Me
O
B
O
O
7
1H NMR (400 MHz, CDCl3)
1.96
1.94
1.00
1.93
1.91
11.70
f1 (ppm)

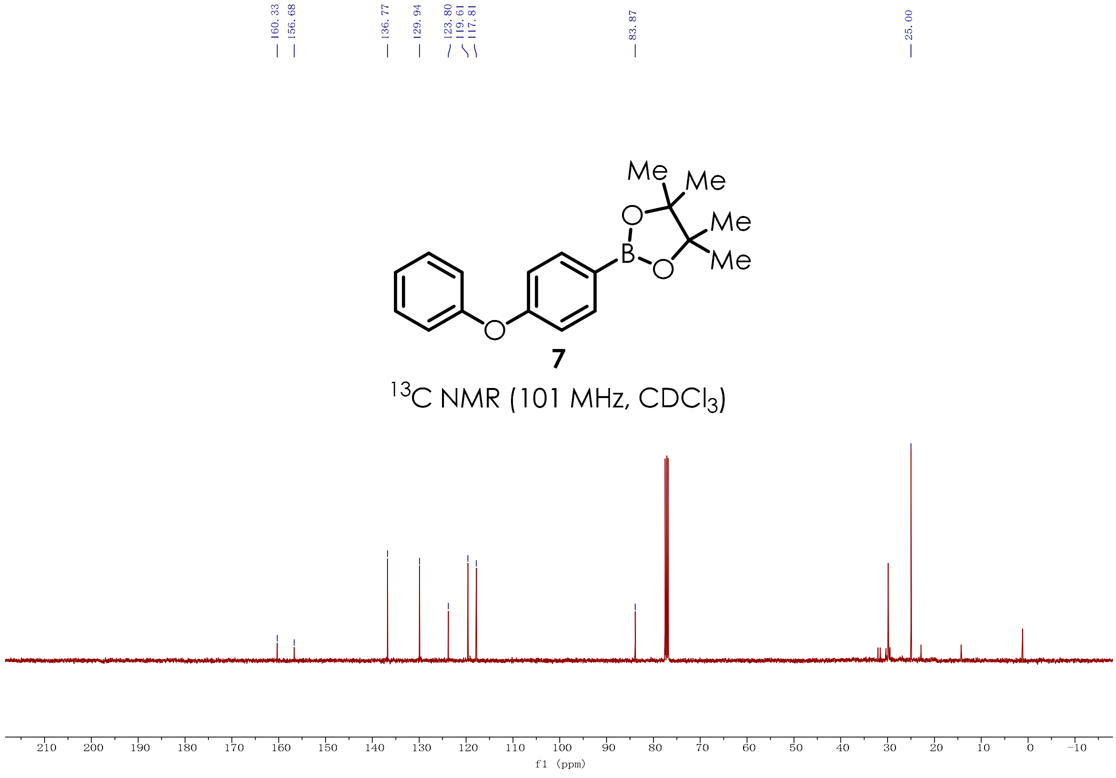

160.33
156.68
136.77
129.94
123.80
119.61
117.81
83.87
25.00
Me
Me
Me
Me
O
B
O
O
7
13C NMR (101 MHz, CDCl3)
f1 (ppm)

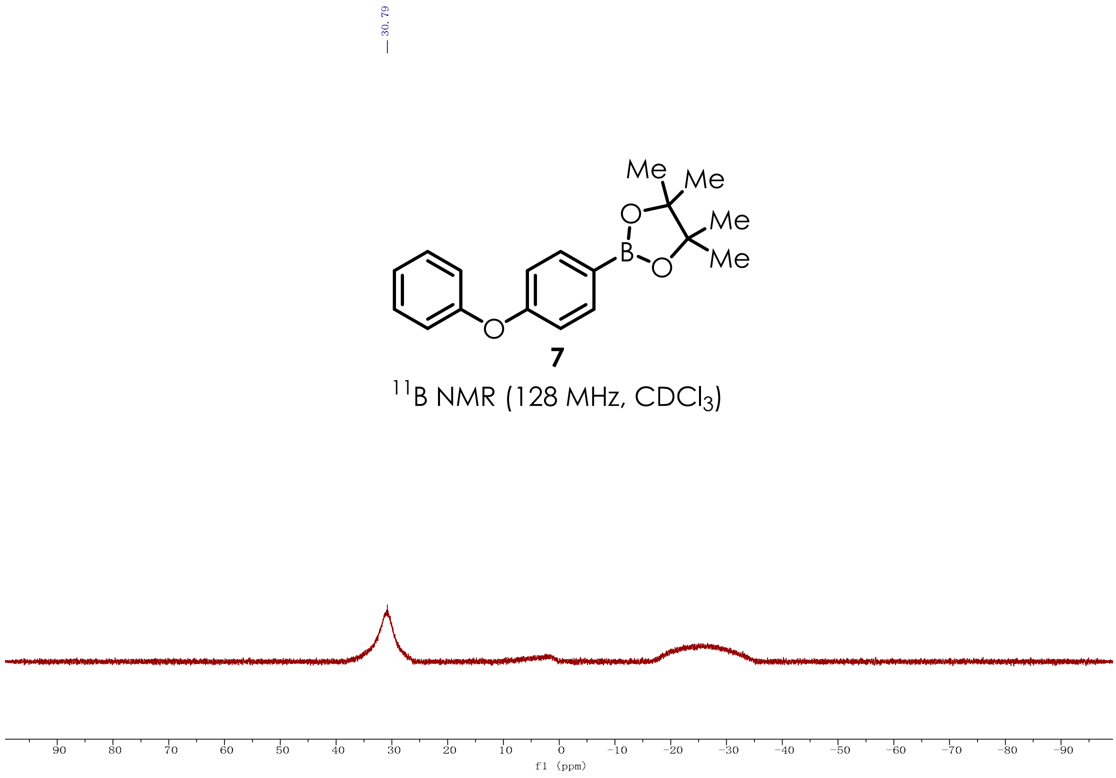
30.79
Me
Me
O
Me
B
O
Me
O
7
11B NMR (128 MHz, CDCl3)
f1 (ppm)

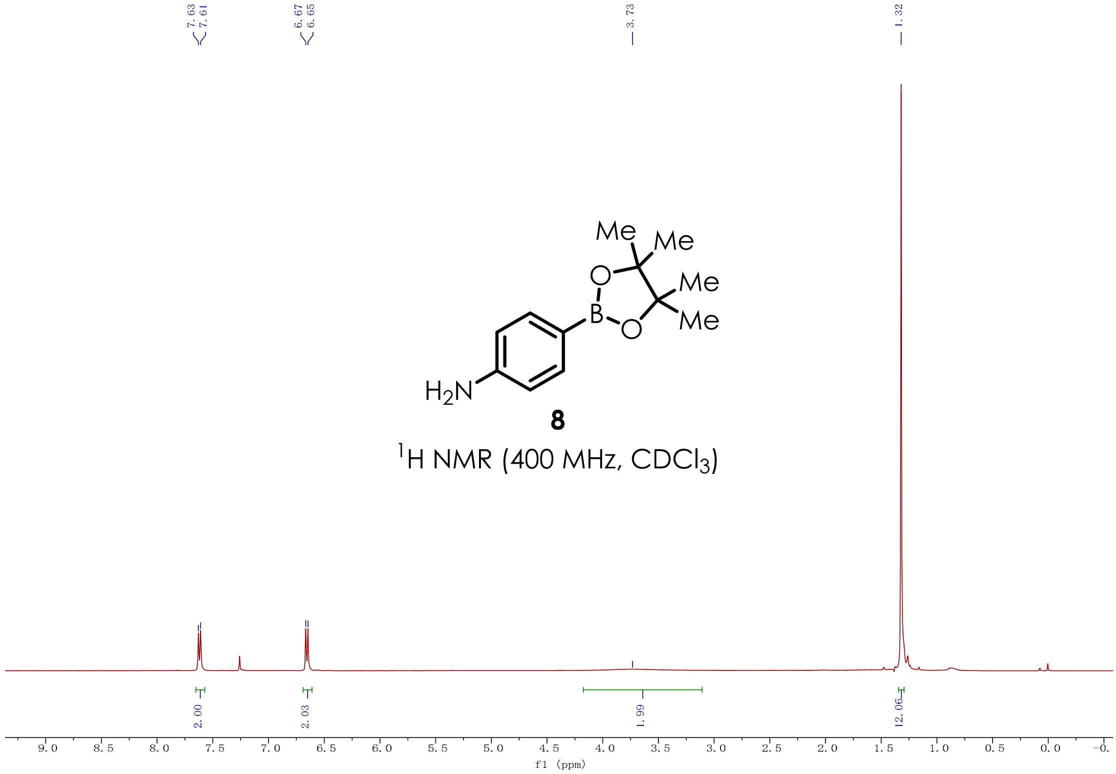
7.63
7.61
6.67
6.65
3.73
1.32
Me
Me
O
Me
B
O
Me
H2N
8
1H NMR (400 MHz, CDCl3)
2.00
2.03
1.99
12.06
f1 (ppm)

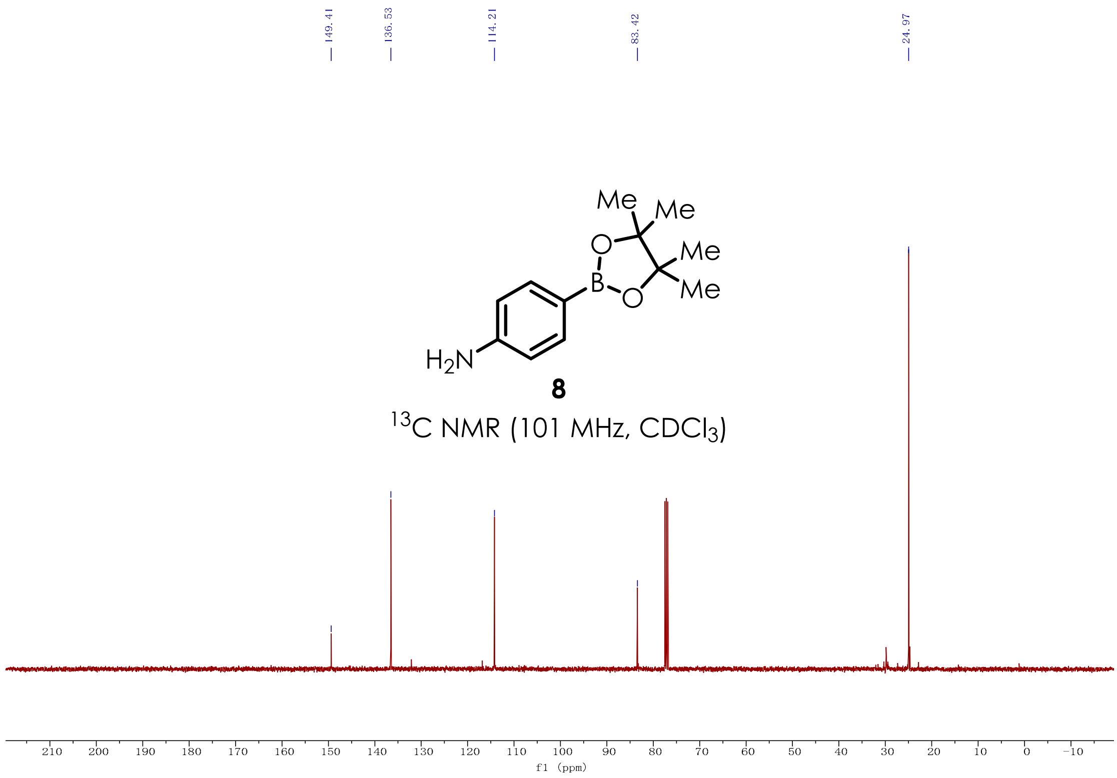
149.41
136.53
114.21
83.42
24.97
Me
Me
Me
Me
O
B
O
H2N
8
13C NMR (101 MHz, CDCl3)
f1 (ppm)

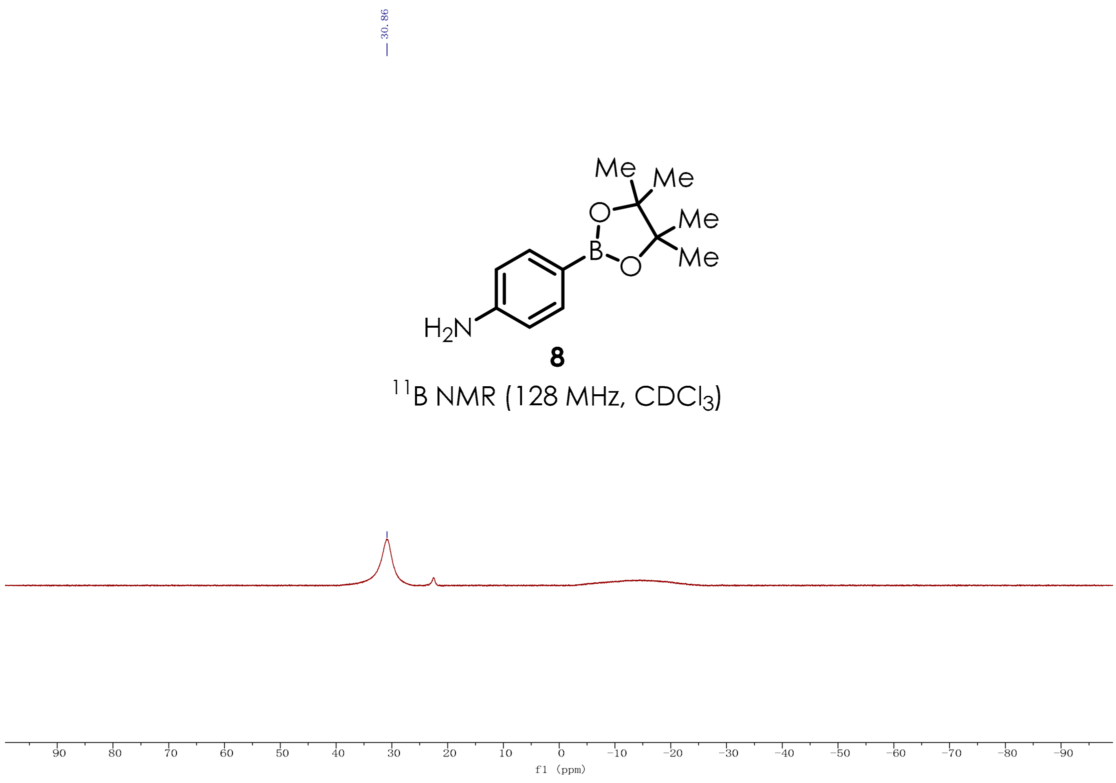
30.86
Me
Me
Me
Me
O
B
O
H2N
8
11B NMR (128 MHz, CDCl3)
f1 (ppm)

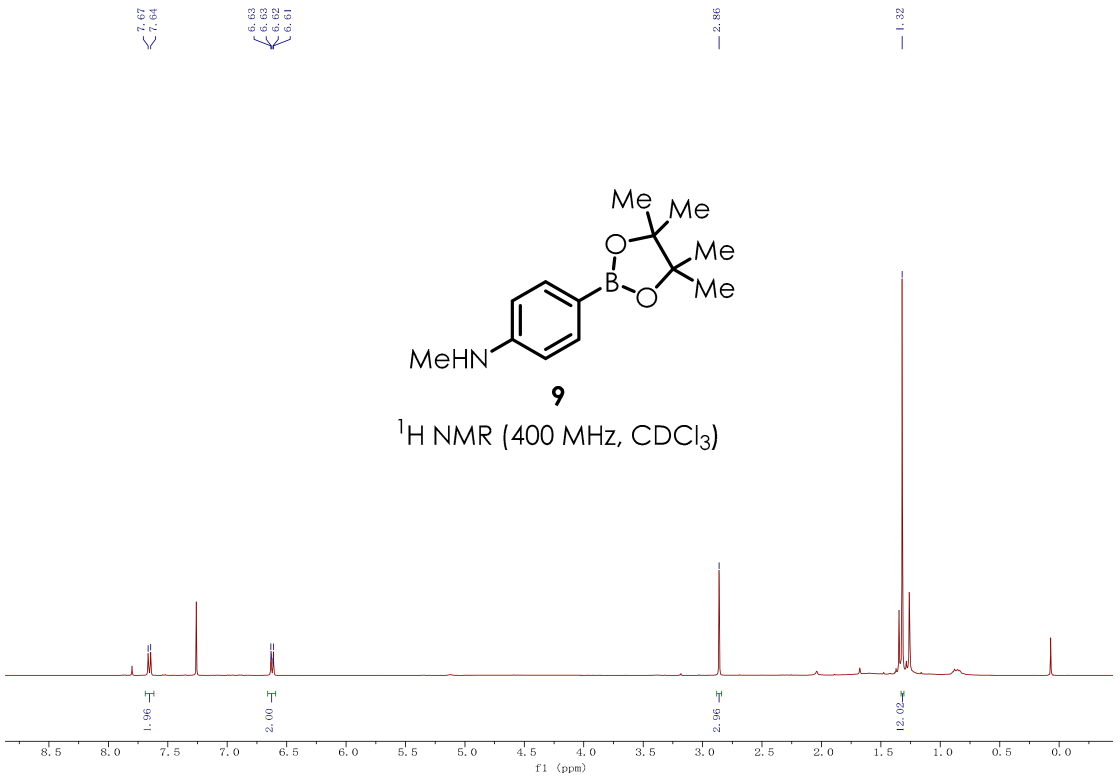

Me
Me
O
B
O
Me
Me
MeHN
9
^{1}H NMR (400 MHz, $CDCl_3$)
f1 (ppm)

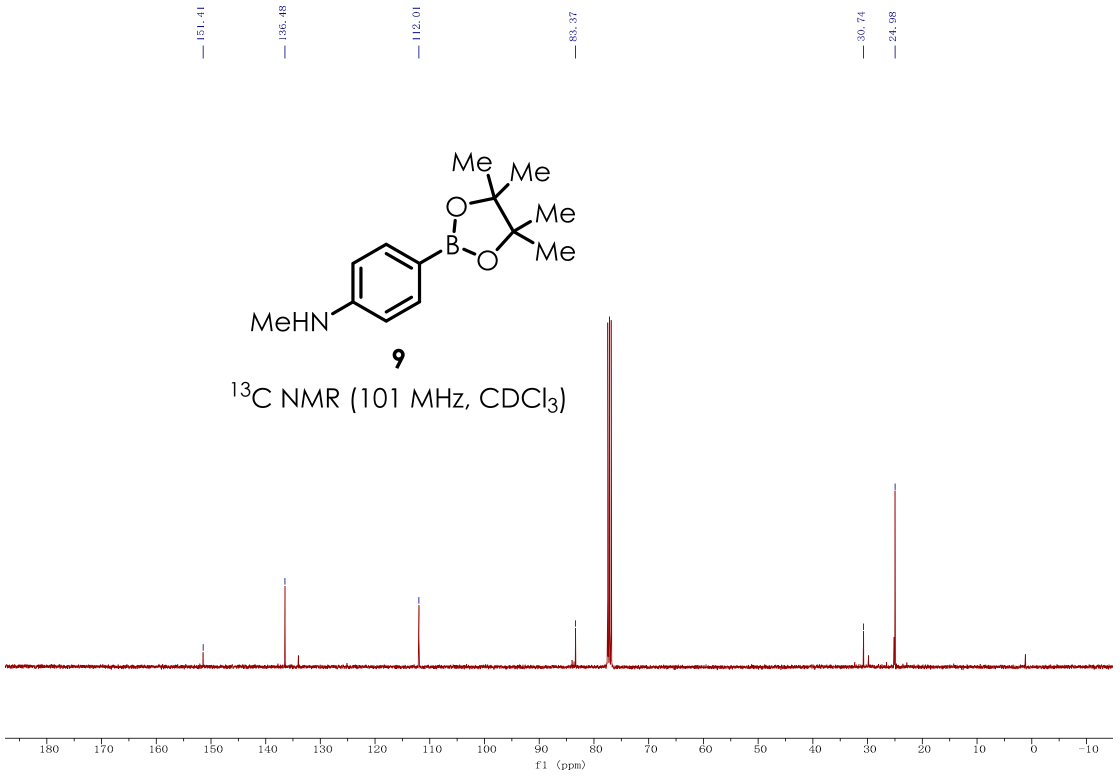

Me
Me
O
B
O
Me
Me
MeHN
9
^{13}C NMR (101 MHz, $CDCl_3$)
f1 (ppm)

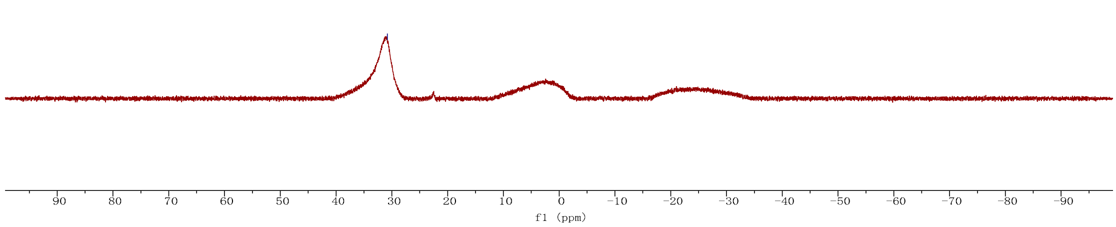

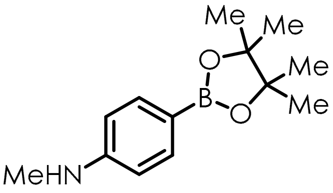

9

^{11}B NMR (128 MHz, $CDCl_3$)

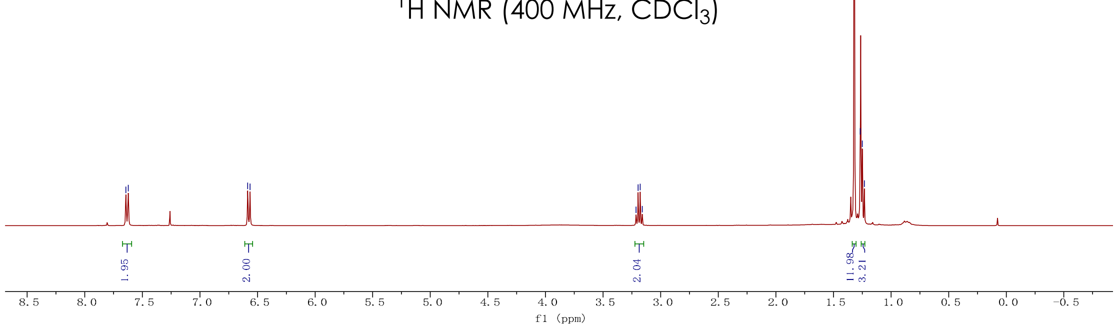

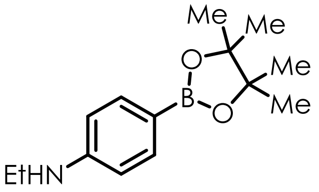

10

^{1}H NMR (400 MHz, $CDCl_3$)

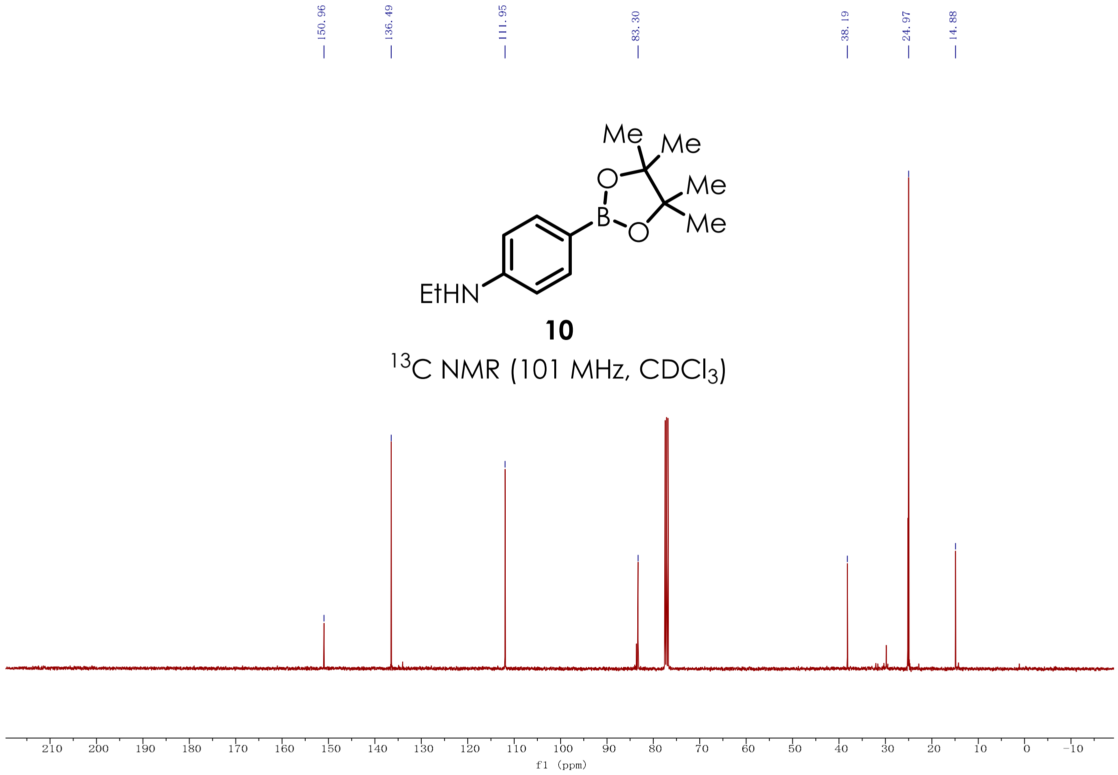

150.96
136.49
111.95
83.30
38.19
24.97
14.88
Me
Me
O
Me
B
O
Me
EtHN
10
^{13}C NMR (101 MHz, $CDCl_3$)
210 200 190 180 170 160 150 140 130 120 110 100 90 80 70 60 50 40 30 20 10 0 -10
f1 (ppm)

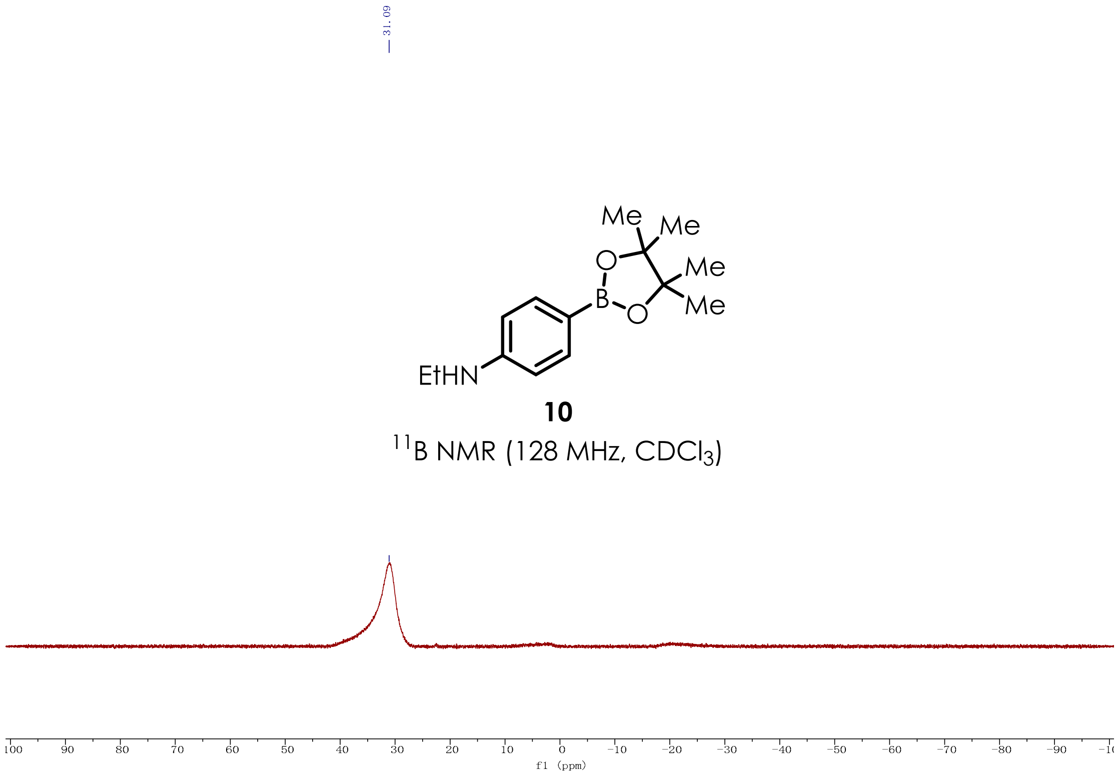

31.09
Me
Me
O
Me
B
O
Me
EtHN
10
^{11}B NMR (128 MHz, $CDCl_3$)
100 90 80 70 60 50 40 30 20 10 0 -10 -20 -30 -40 -50 -60 -70 -80 -90
f1 (ppm)

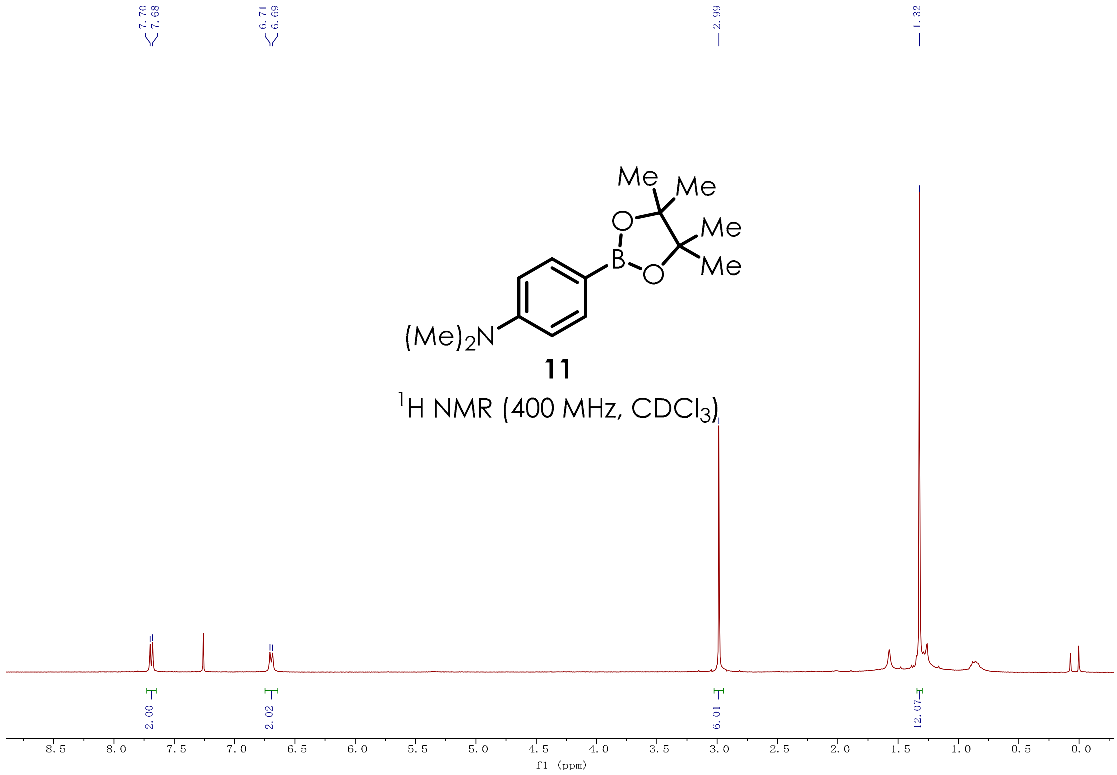
7.70
7.68
6.71
6.69
2.99
1.32
Me
Me
Me
Me
O
B
O
(Me)2N
11
1H NMR (400 MHz, CDCl3)
2.00
2.02
6.01
12.07
f1 (ppm)

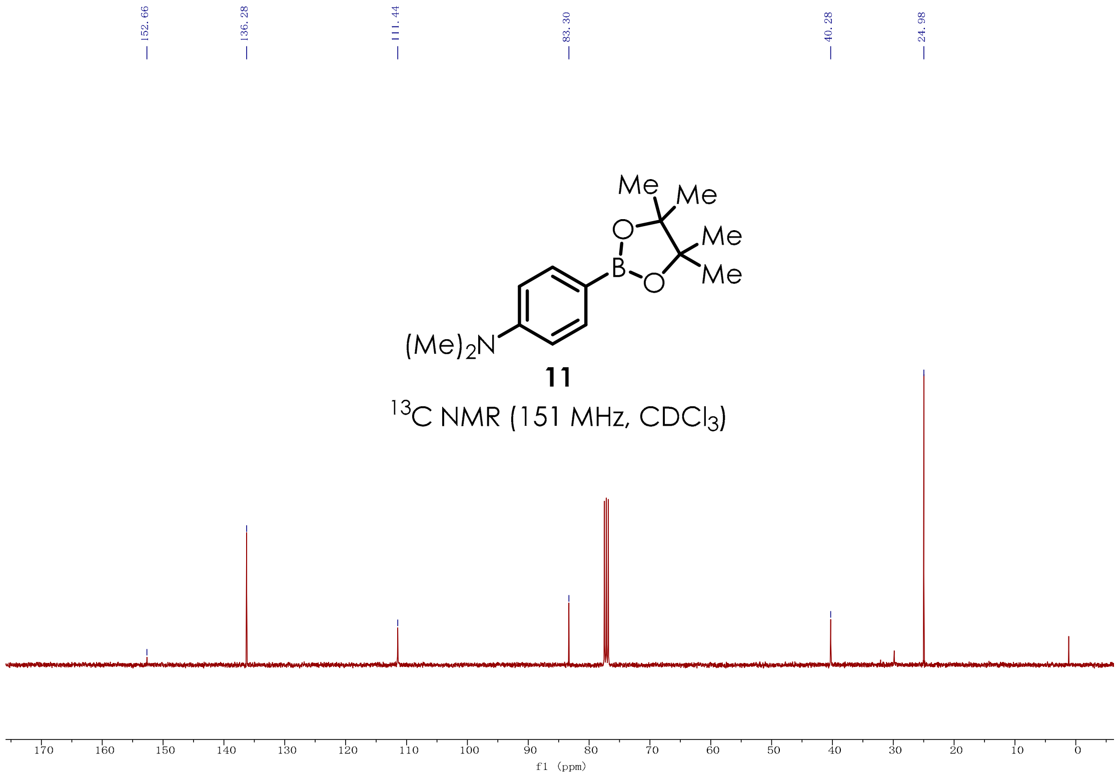
152.66
136.28
111.44
83.30
40.28
24.98
Me
Me
Me
Me
O
B
O
(Me)2N
11
13C NMR (151 MHz, CDCl3)
f1 (ppm)

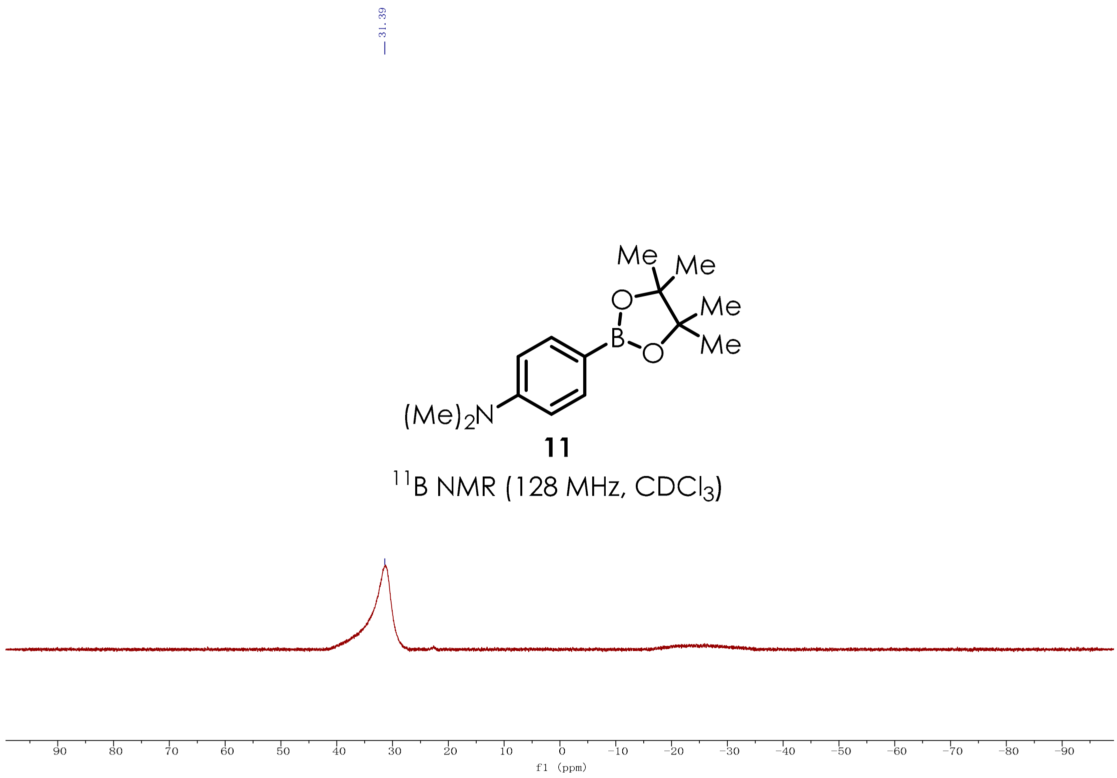

31.39
Me
Me
Me
Me
O
B
O
(Me)2N
11
11B NMR (128 MHz, CDCl3)
f1 (ppm)

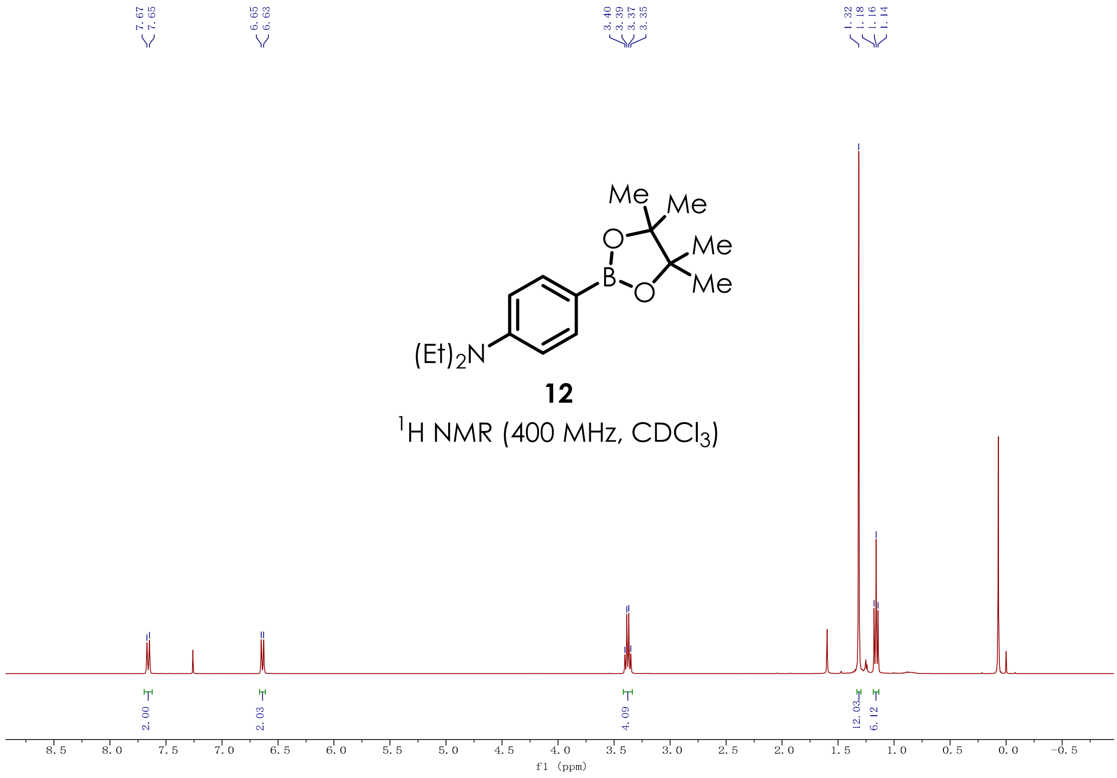

7.67
7.65
6.65
6.63
3.40
3.39
3.37
3.35
1.32
1.18
1.16
1.14
Me
Me
Me
Me
O
B
O
(Et)2N
12
1H NMR (400 MHz, CDCl3)
2.00
2.03
4.09
12.03
6.12
f1 (ppm)

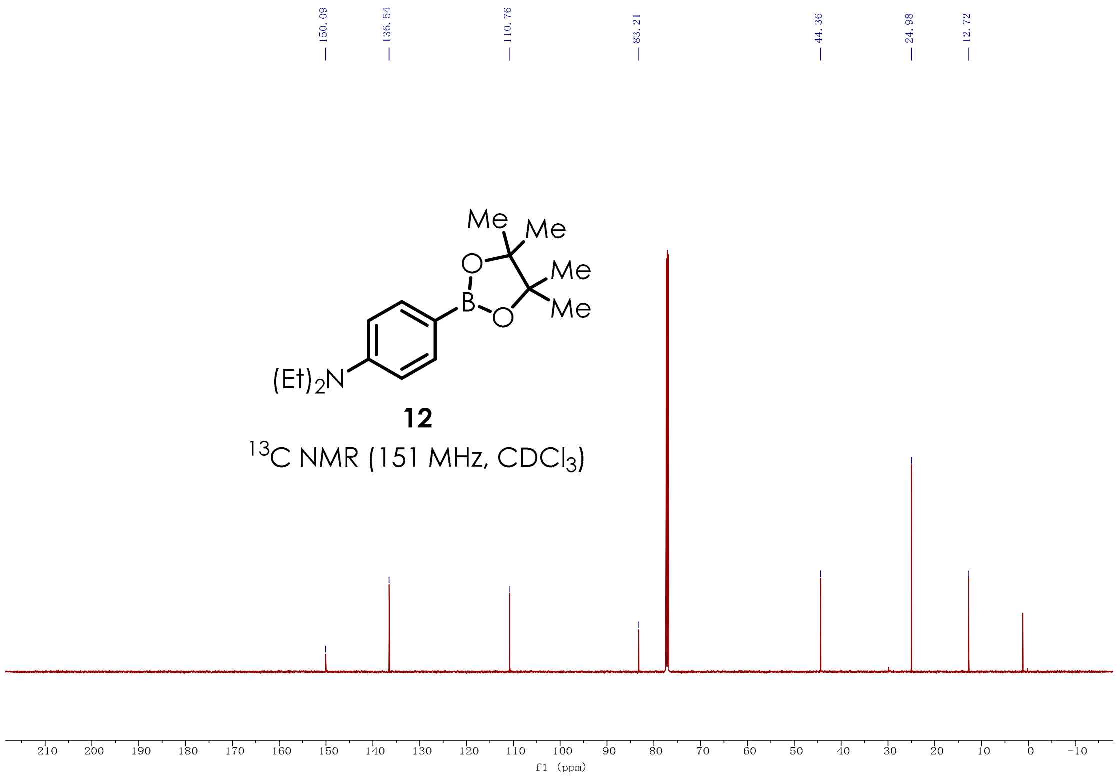
150.09
136.54
110.76
83.21
44.36
24.98
12.72
Me
Me
Me
Me
O
B
O
(Et)2N
12
13C NMR (151 MHz, CDCl3)
210 200 190 180 170 160 150 140 130 120 110 100 90 80 70 60 50 40 30 20 10 0 -10
f1 (ppm)

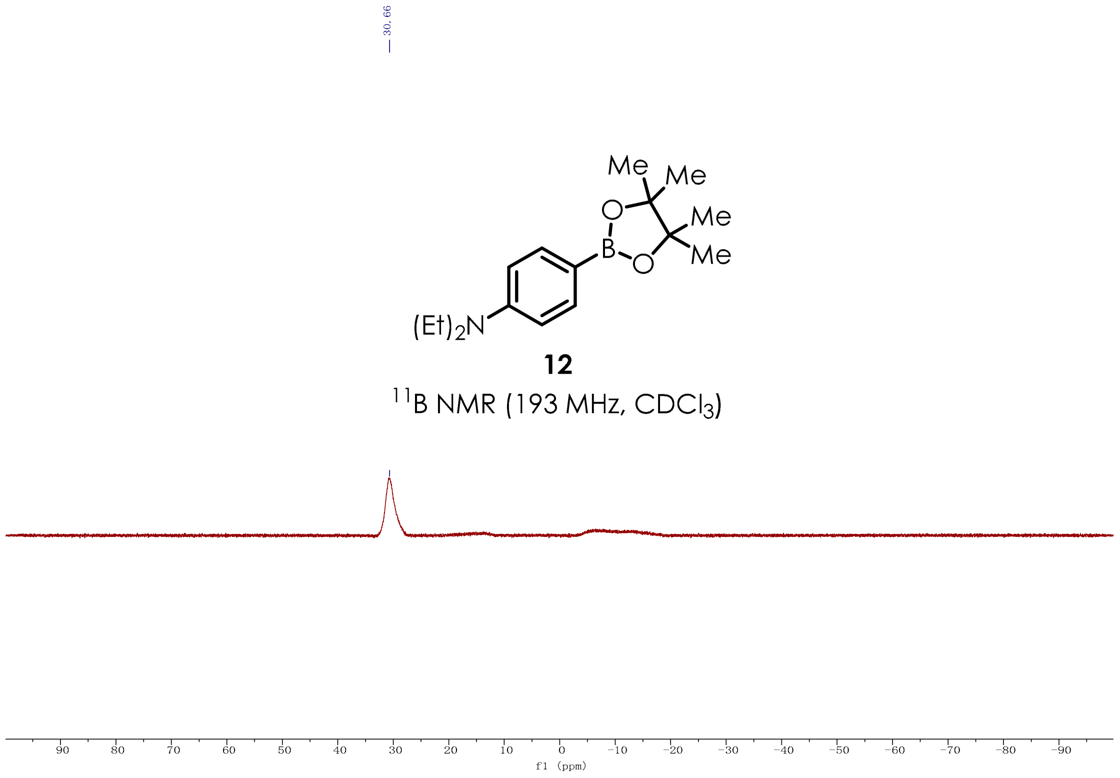
30.66
Me
Me
Me
Me
O
B
O
(Et)2N
12
11B NMR (193 MHz, CDCl3)
90 80 70 60 50 40 30 20 10 0 -10 -20 -30 -40 -50 -60 -70 -80 -90
f1 (ppm)

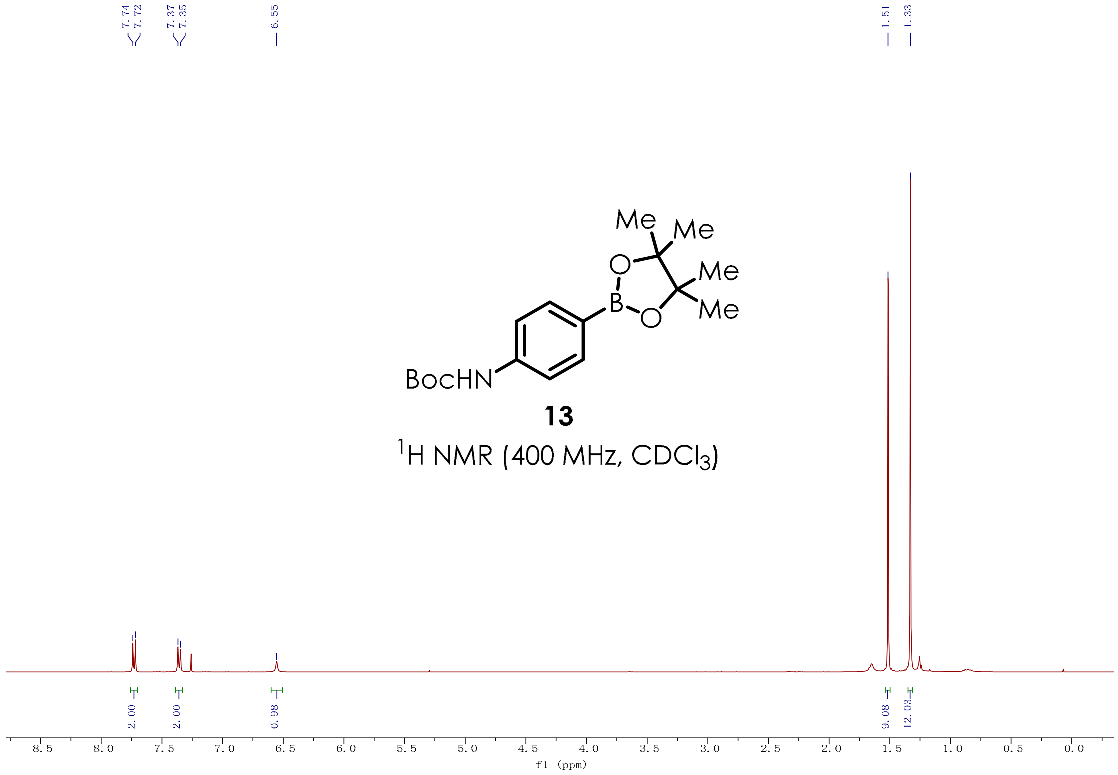
7.74
7.72
7.37
7.35
6.55
1.51
1.33
Me
Me
Me
Me
O
B
O
BocHN
13
1H NMR (400 MHz, CDCl3)
2.00
2.00
0.98
9.08
12.03
f1 (ppm)

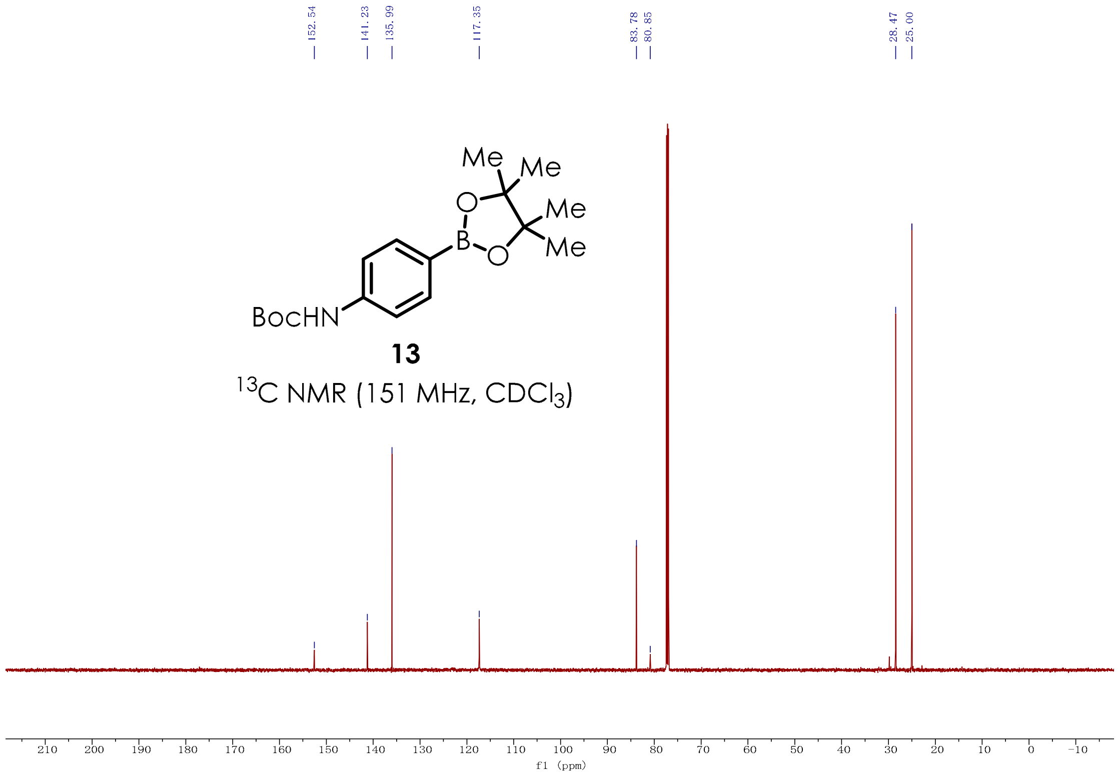
152.54
141.23
135.99
117.35
83.78
80.85
28.47
25.00
Me
Me
Me
Me
O
B
O
BocHN
13
13C NMR (151 MHz, CDCl3)
f1 (ppm)

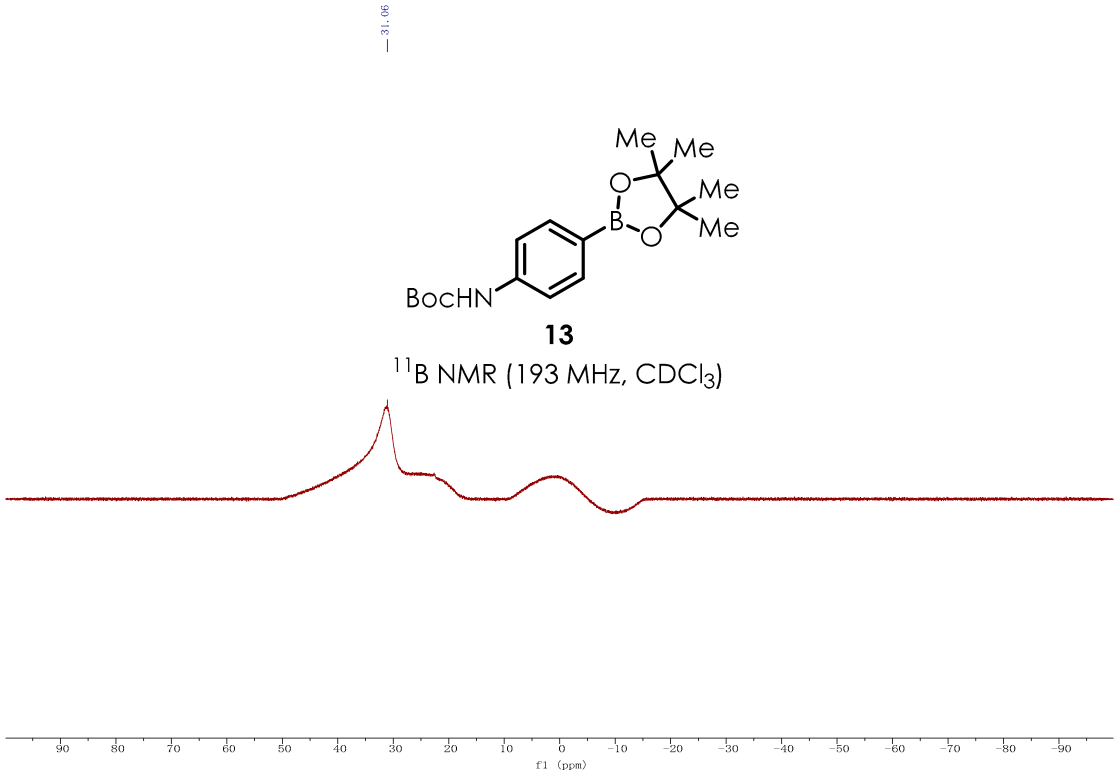

13

^{11}B NMR (193 MHz, $CDCl_3$)

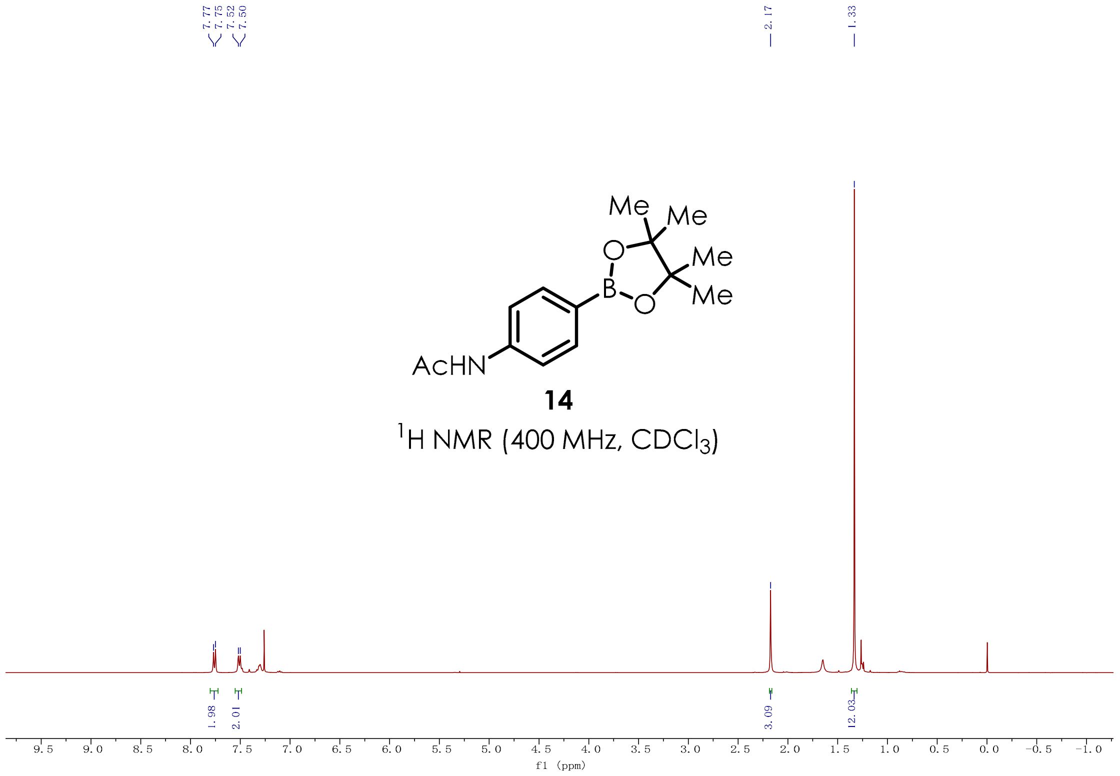

14

^{1}H NMR (400 MHz, $CDCl_3$)

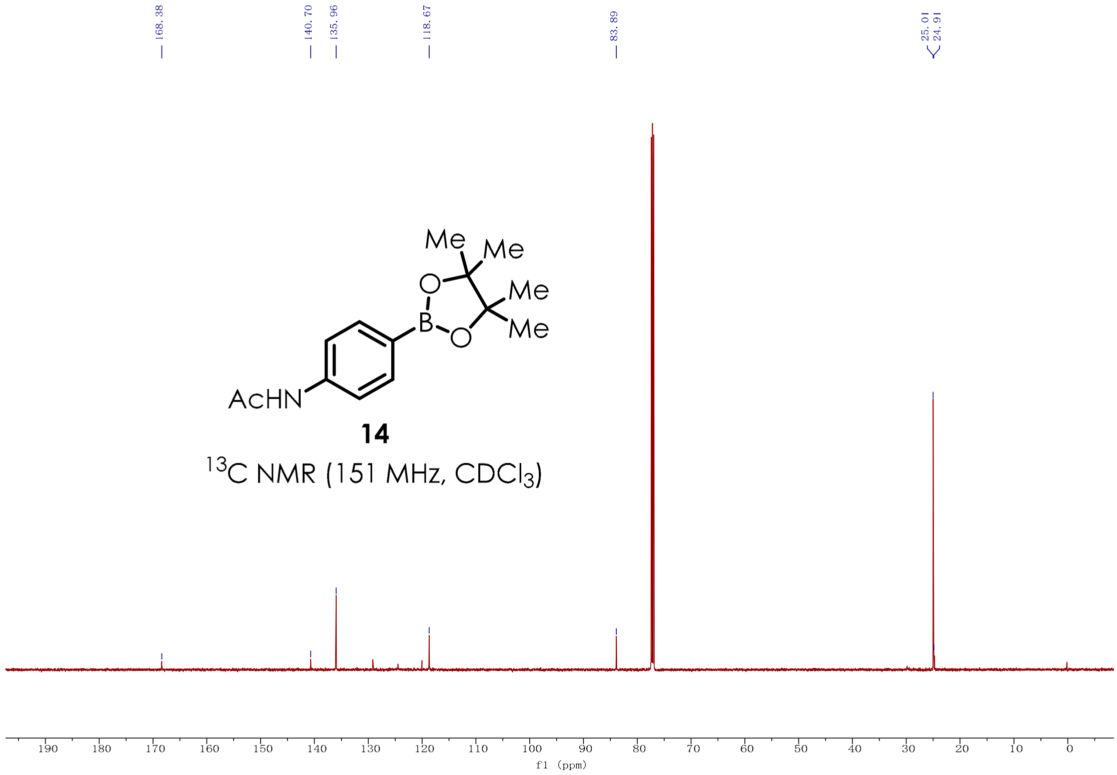
168.38
140.70
135.96
118.67
83.89
25.01
24.91
Me
Me
Me
Me
O
B
O
AcHN
14
^{13}C NMR (151 MHz, CDCl$_3$)
190
180
170
160
150
140
130
120
110
100
90
80
70
60
50
40
30
20
10
0
f1 (ppm)

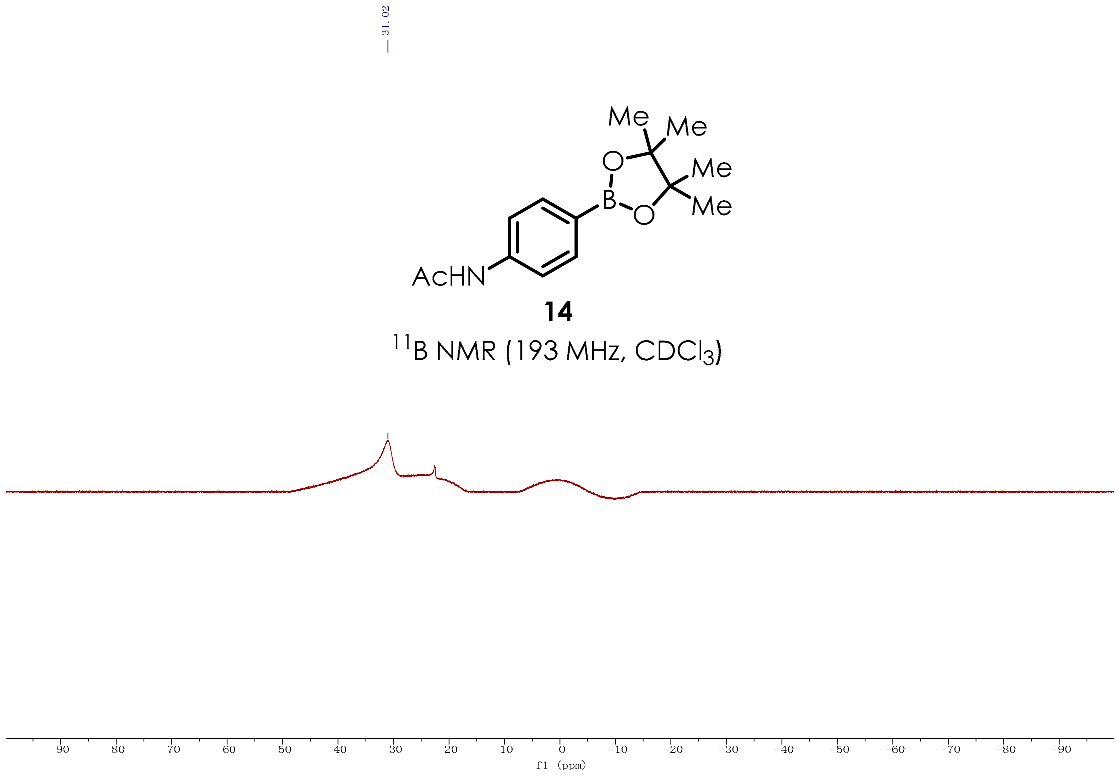
31.02
Me
Me
Me
Me
O
B
O
AcHN
14
^{11}B NMR (193 MHz, CDCl$_3$)
90
80
70
60
50
40
30
20
10
0
-10
-20
-30
-40
-50
-60
-70
-80
-90
f1 (ppm)

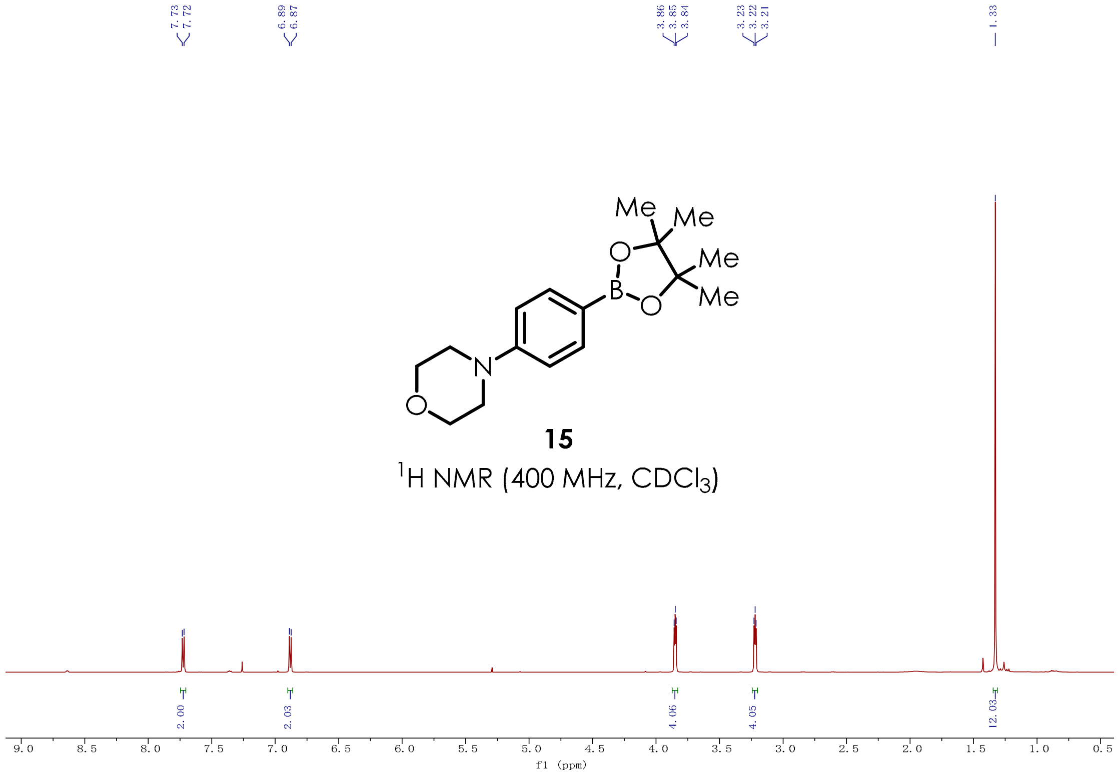
7.73
7.72
6.89
6.87
3.86
3.85
3.84
3.23
3.22
3.21
1.33
15
^{1}H NMR (400 MHz, $CDCl_3$)
2.00
2.03
4.06
4.05
12.03
f1 (ppm)

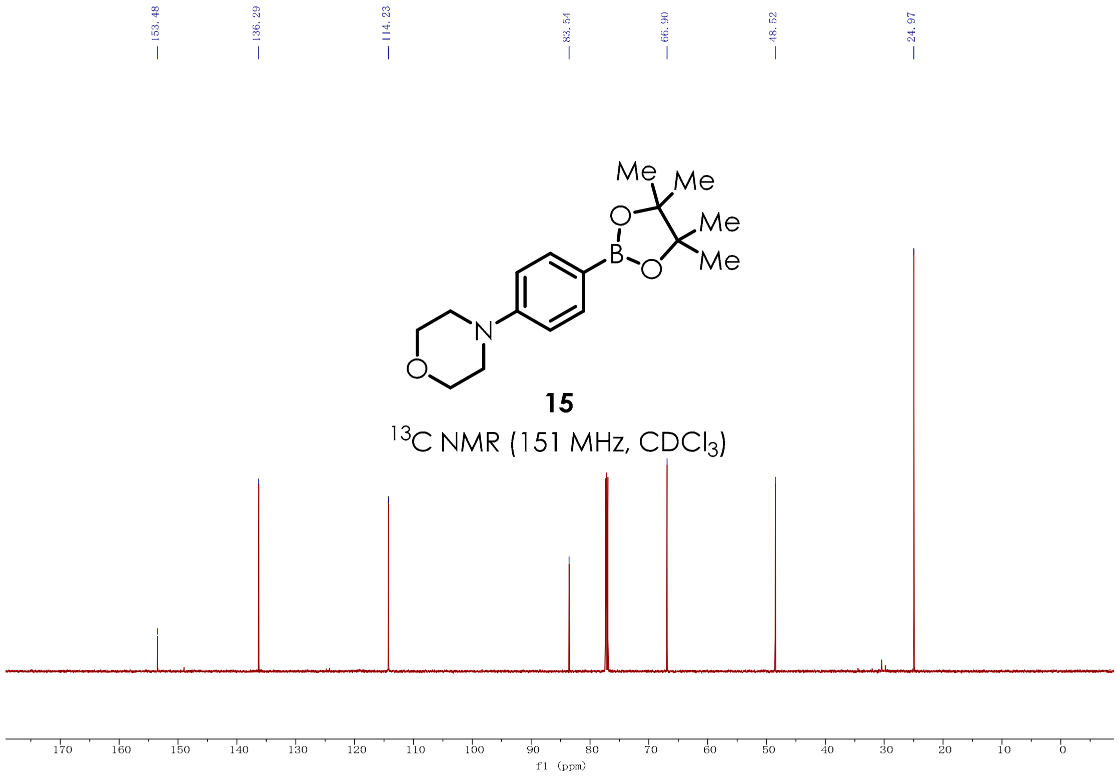
153.48
136.29
114.23
83.54
66.90
48.52
24.97
15
^{13}C NMR (151 MHz, $CDCl_3$)
f1 (ppm)

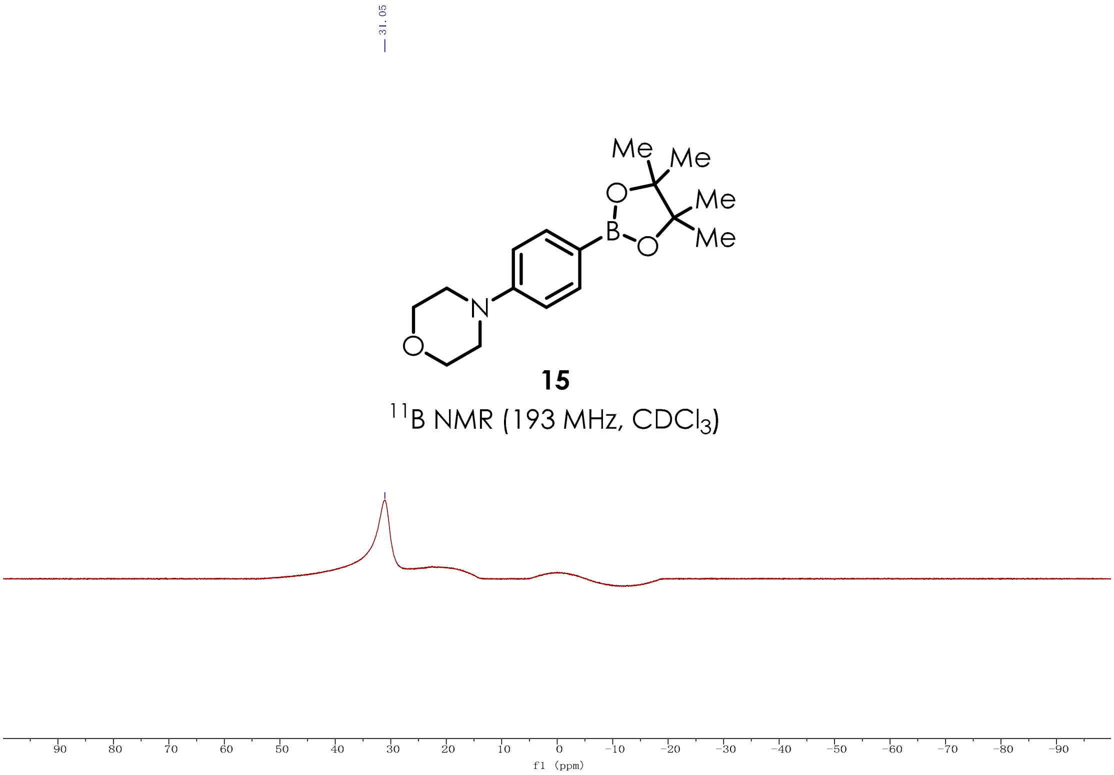

15

^{11}B NMR (193 MHz, $CDCl_3$)

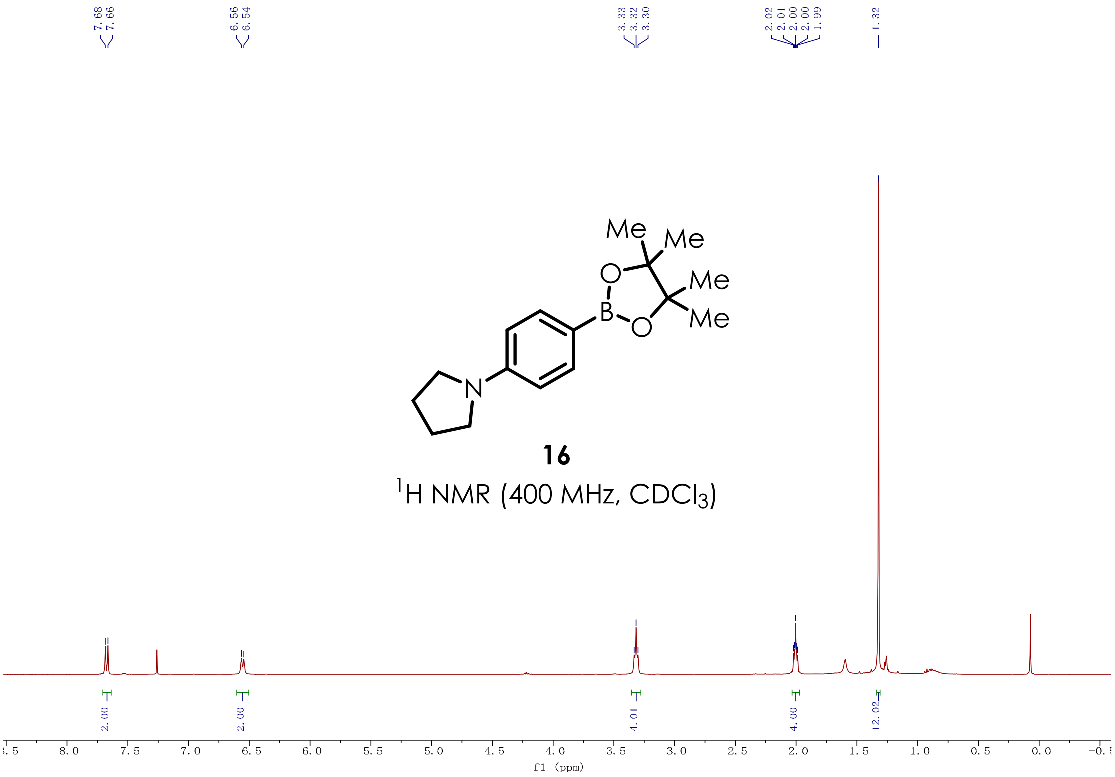

16

^{1}H NMR (400 MHz, $CDCl_3$)

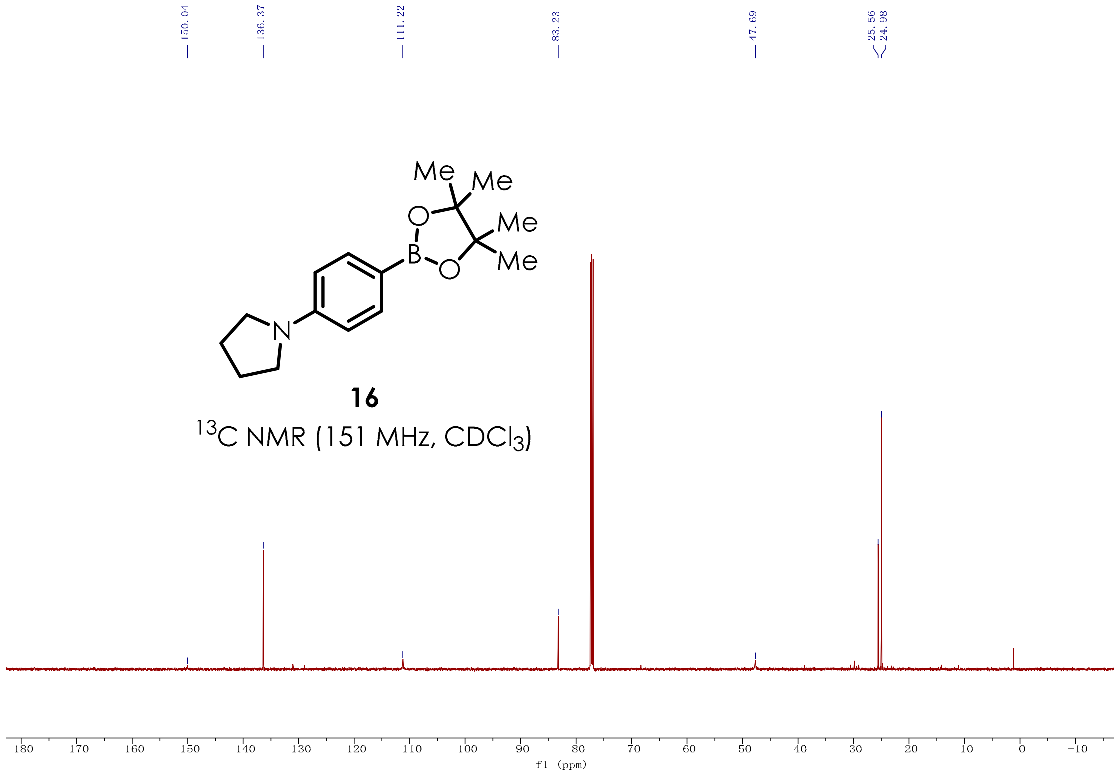
150.04
136.37
111.22
83.23
47.69
25.56
24.98
Me
Me
Me
Me
O
B
O
N
16
^{13}C NMR (151 MHz, $CDCl_3$)
f1 (ppm)

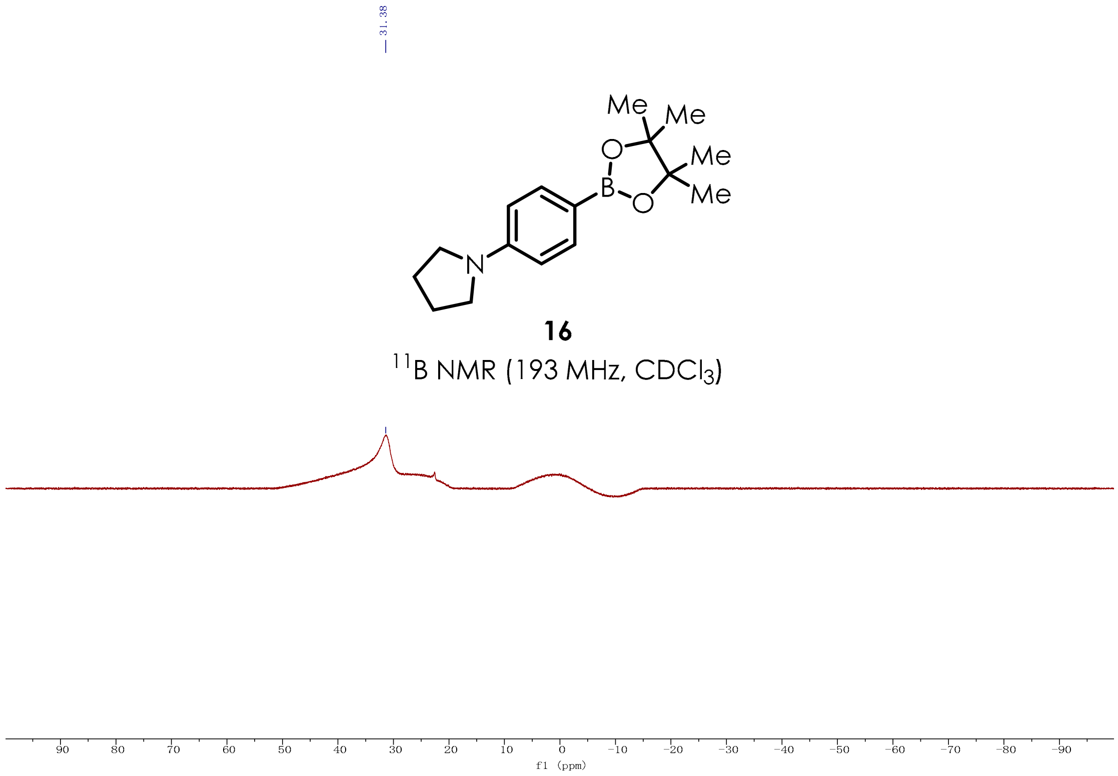
31.38
Me
Me
Me
Me
O
B
O
N
16
^{11}B NMR (193 MHz, $CDCl_3$)
f1 (ppm)

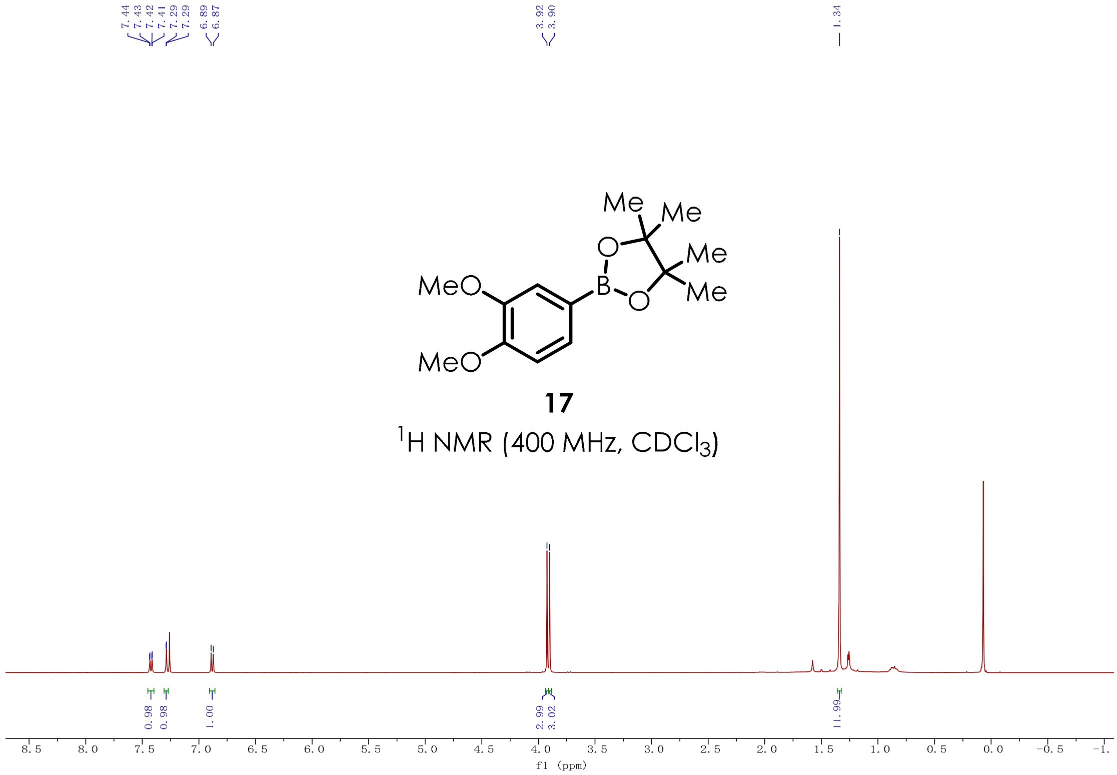
7.44
7.43
7.42
7.41
7.29
7.29
6.89
6.87
3.92
3.90
1.34
MeO
MeO
B
O
O
Me
Me
Me
Me
17
1H NMR (400 MHz, CDCl3)
0.98
0.98
1.00
2.99
3.02
11.99
8.5 8.0 7.5 7.0 6.5 6.0 5.5 5.0 4.5 4.0 3.5 3.0 2.5 2.0 1.5 1.0 0.5 0.0 -0.5 -1.
f1 (ppm)

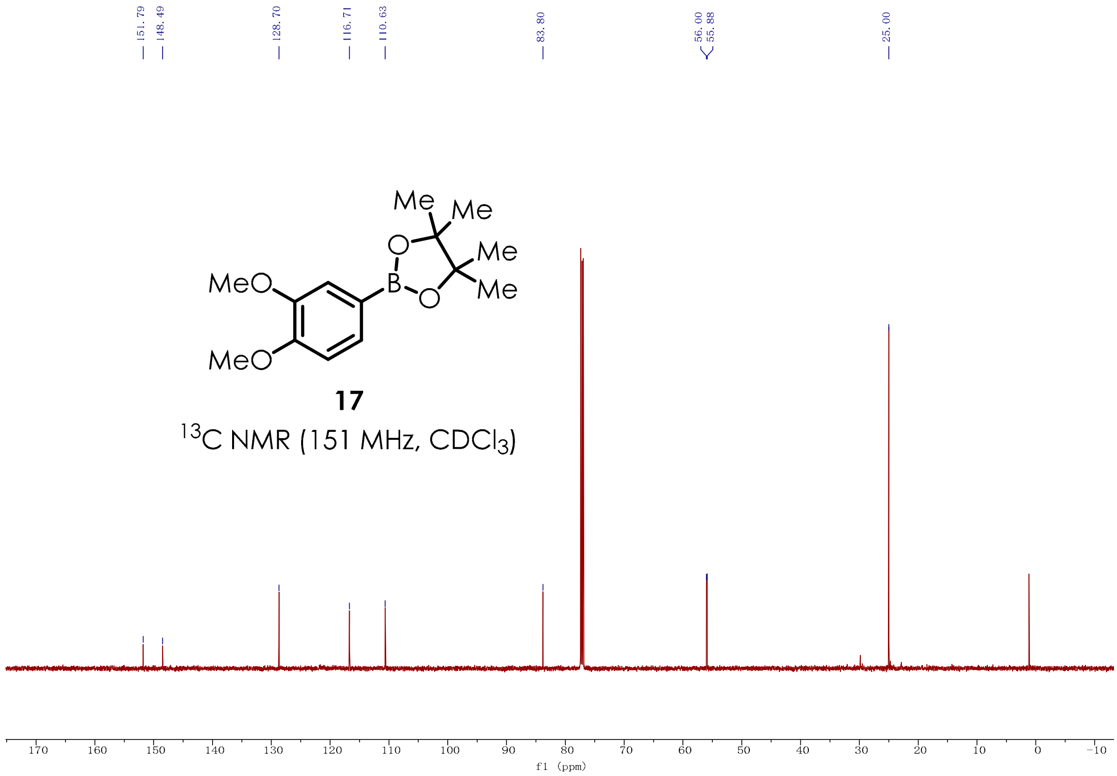
151.79
148.49
128.70
116.71
110.63
83.80
56.00
55.88
25.00
MeO
MeO
B
O
O
Me
Me
Me
Me
17
13C NMR (151 MHz, CDCl3)
170 160 150 140 130 120 110 100 90 80 70 60 50 40 30 20 10 0 -10
f1 (ppm)

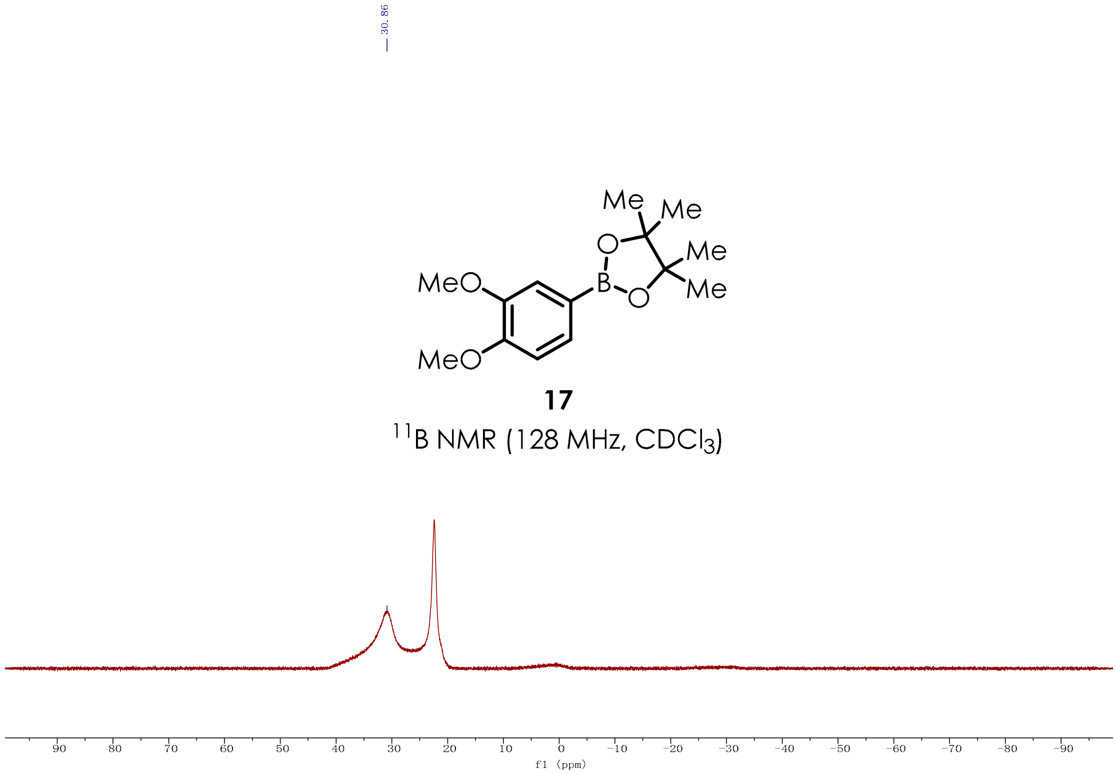

17

^{11}B NMR (128 MHz, $CDCl_3$)

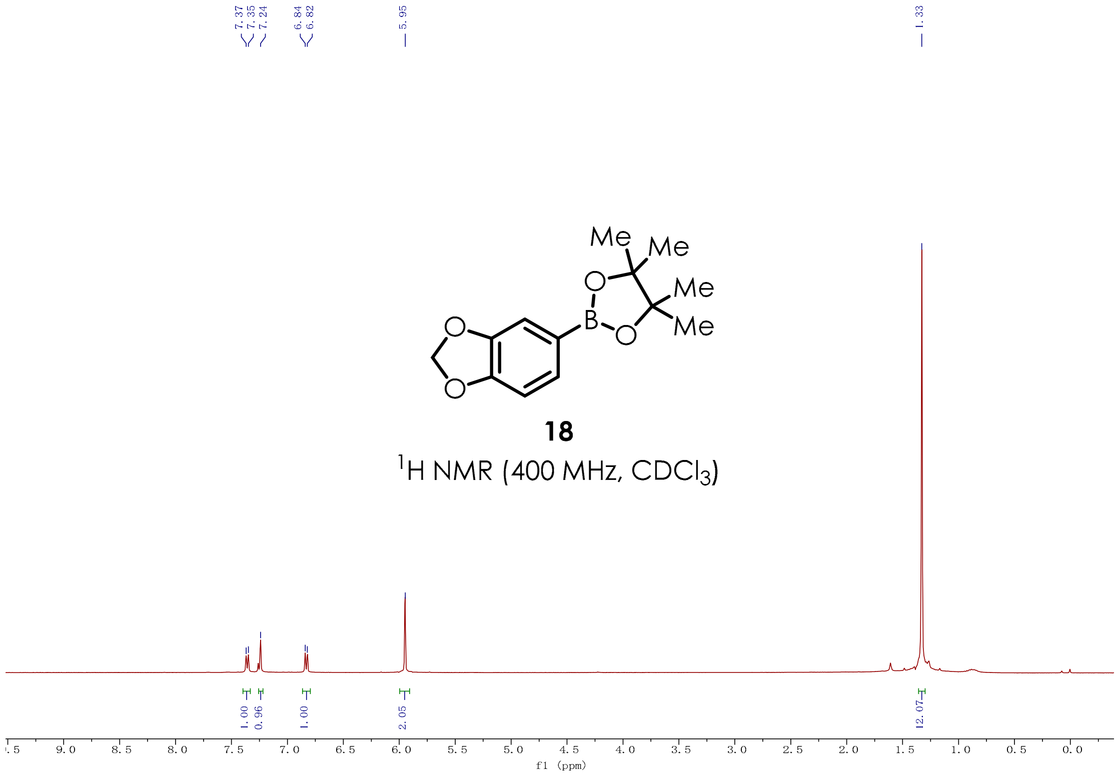

18

^{1}H NMR (400 MHz, $CDCl_3$)

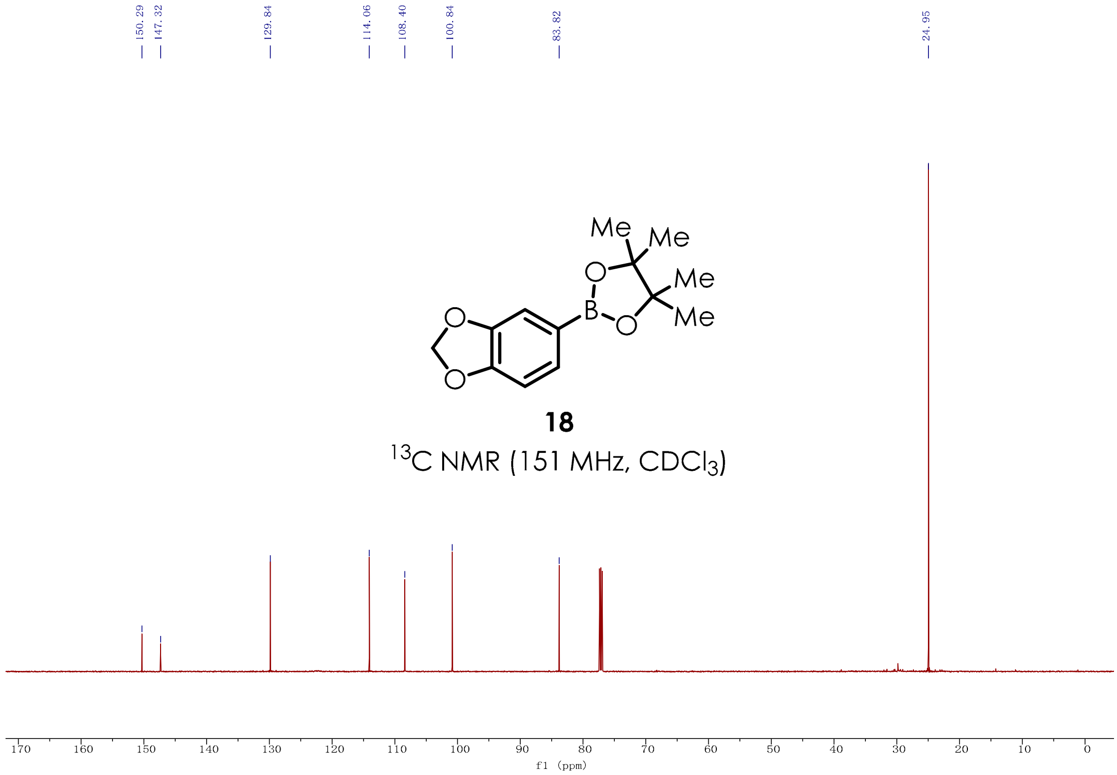

18

^{13}C NMR (151 MHz, $CDCl_3$)

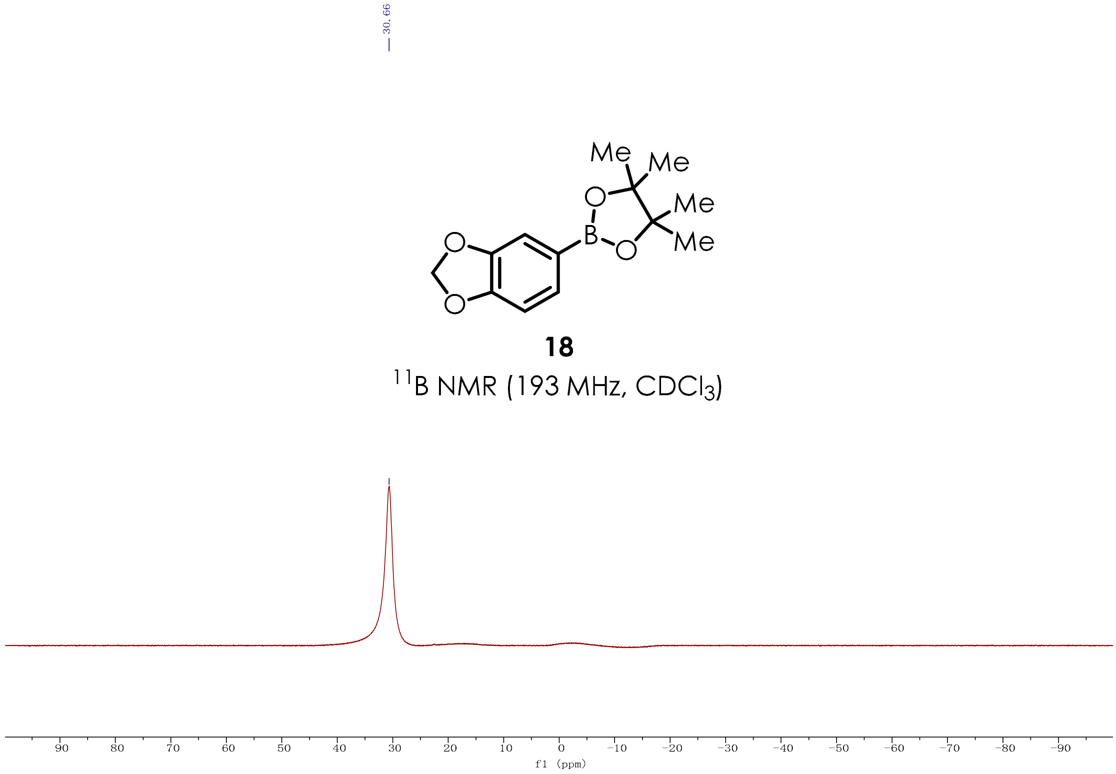

18

^{11}B NMR (193 MHz, $CDCl_3$)

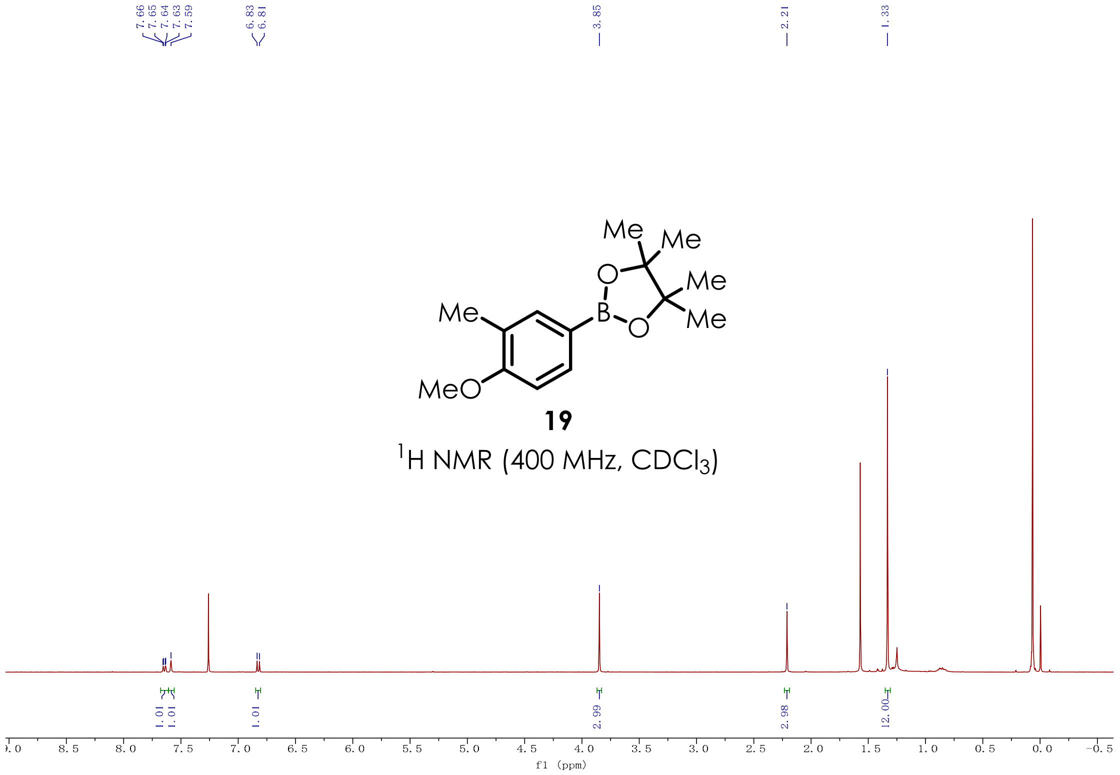
7.66
7.65
7.64
7.63
7.59
6.83
6.81
3.85
2.21
1.33
Me
Me
O
Me
Me
B
O
Me
MeO
19
1H NMR (400 MHz, CDCl3)
1.01
1.01
1.01
2.99
2.98
12.00
f1 (ppm)

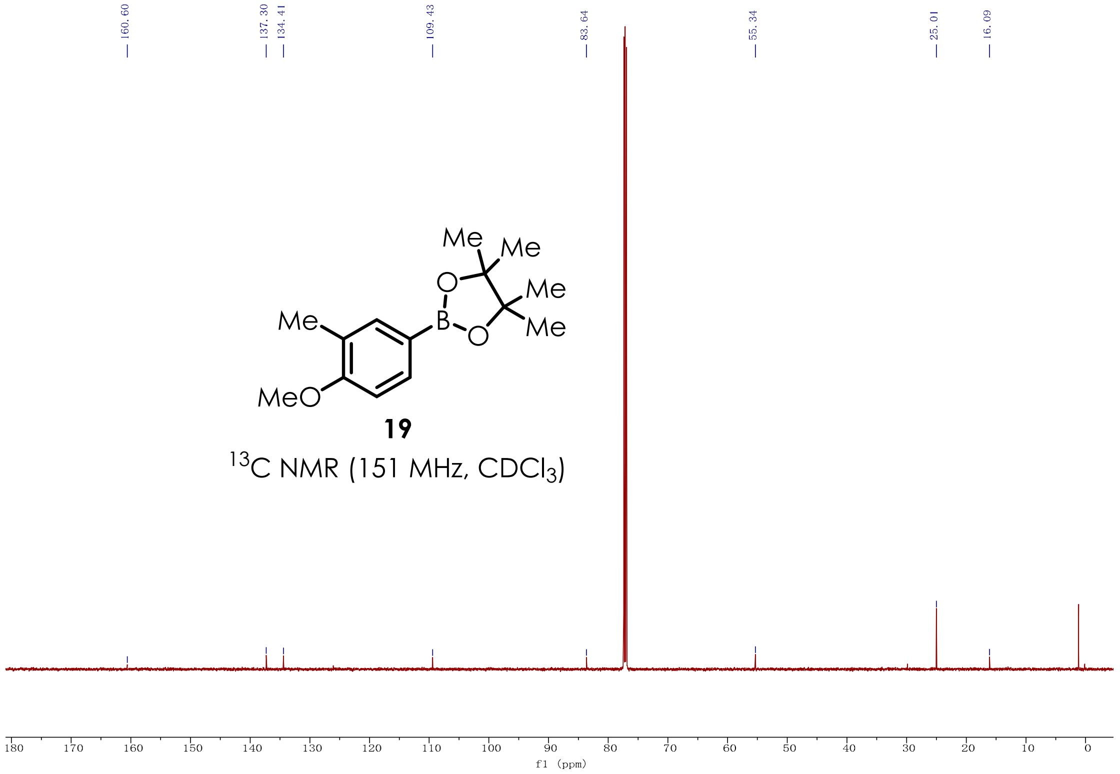
160.60
137.30
134.41
109.43
83.64
55.34
25.01
16.09
Me
Me
O
Me
Me
B
O
Me
MeO
19
13C NMR (151 MHz, CDCl3)
f1 (ppm)

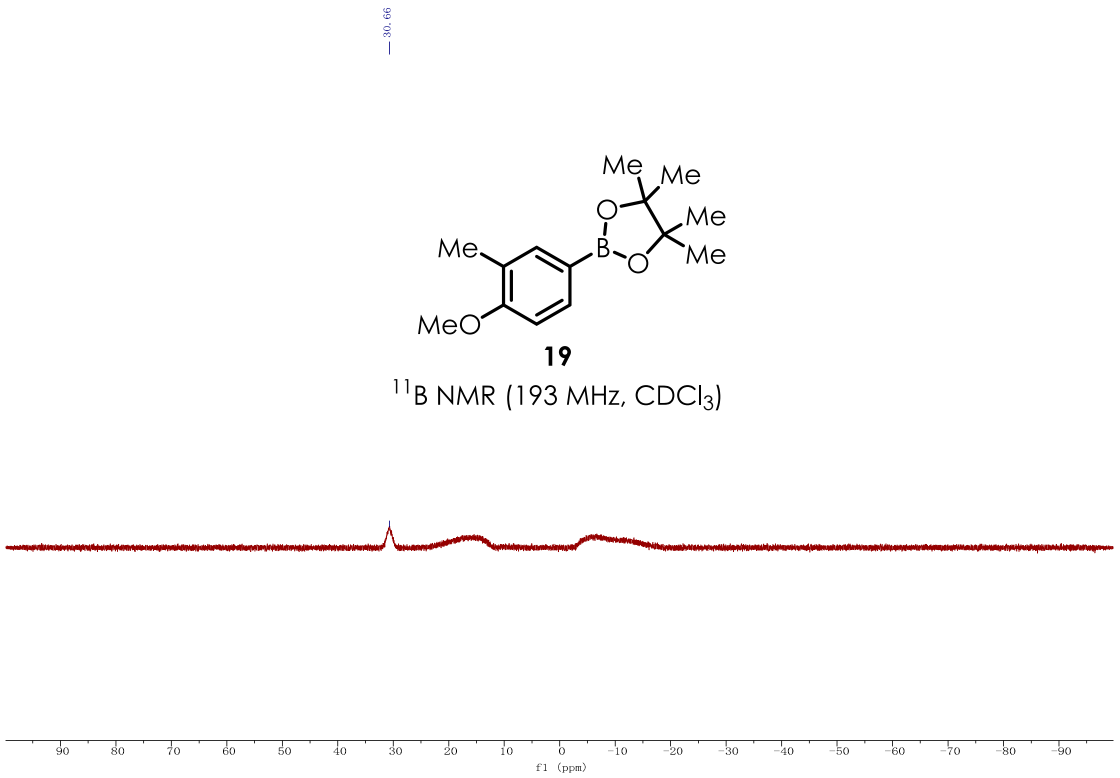

19

^{11}B NMR (193 MHz, $CDCl_3$)

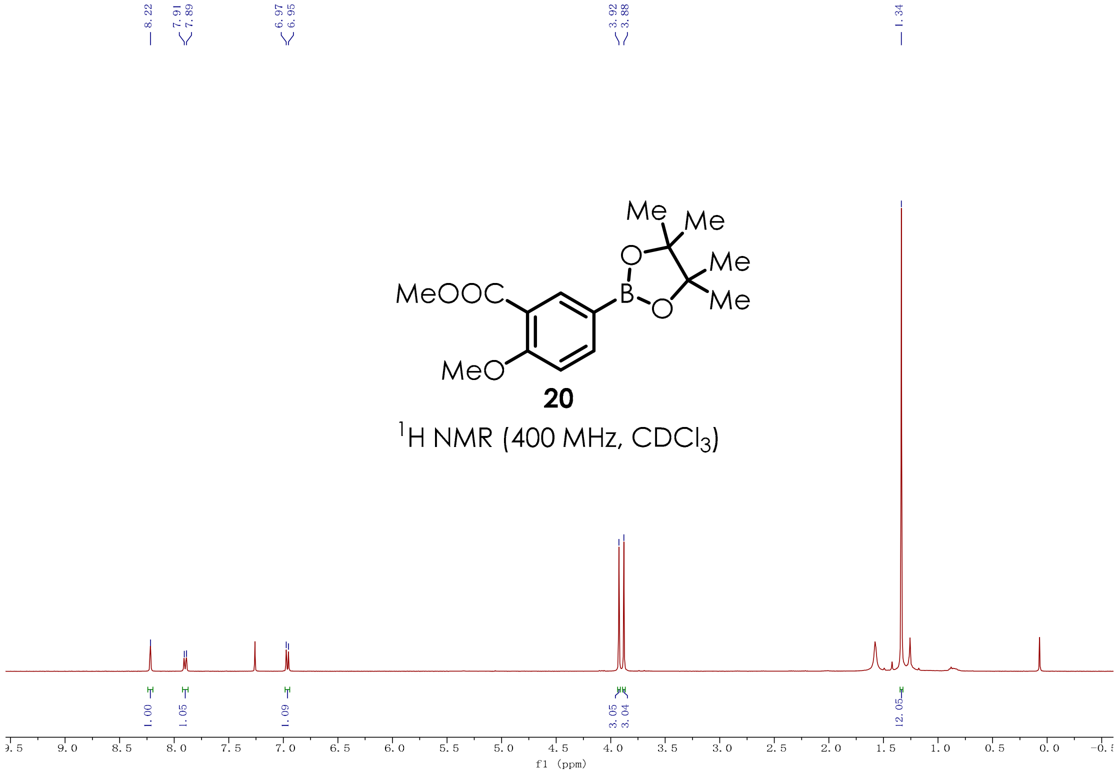

20

^{1}H NMR (400 MHz, $CDCl_3$)

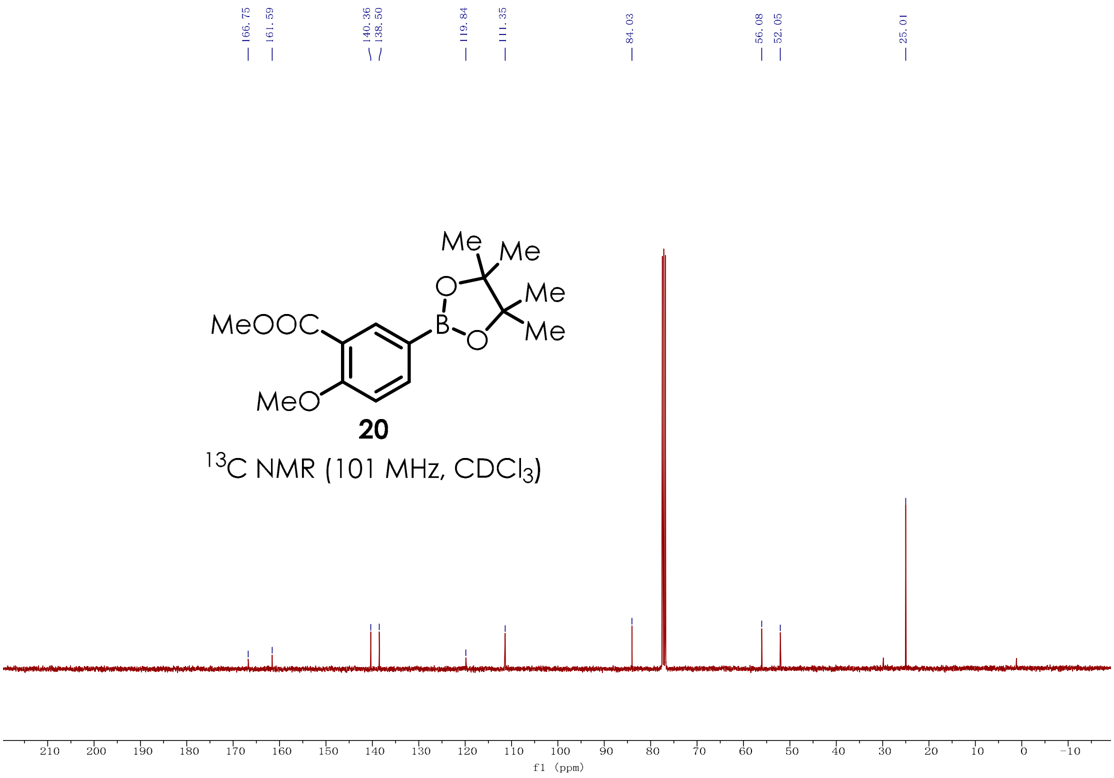
166.75
161.59
140.36
138.50
119.84
111.35
84.03
56.08
52.05
25.01
MeOOC
MeO
Me
O
B
20
¹³C NMR (101 MHz, CDCl₃)
f1 (ppm)

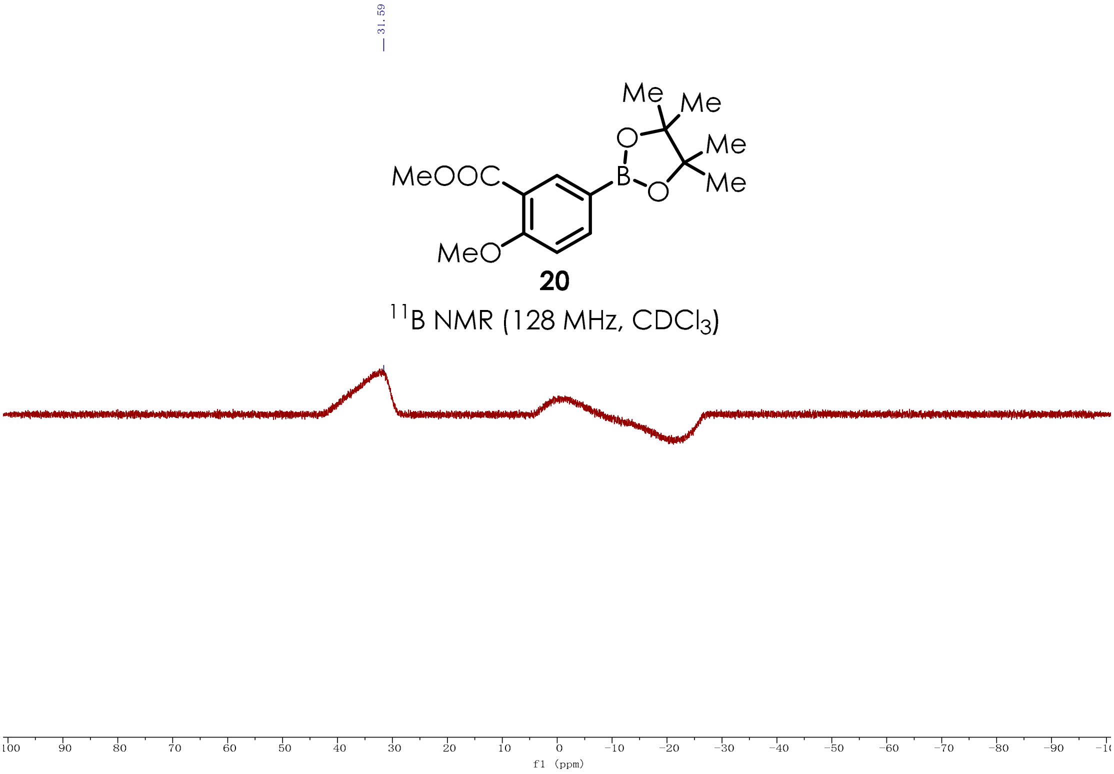
31.59
MeOOC
MeO
Me
O
B
20
¹¹B NMR (128 MHz, CDCl₃)
f1 (ppm)

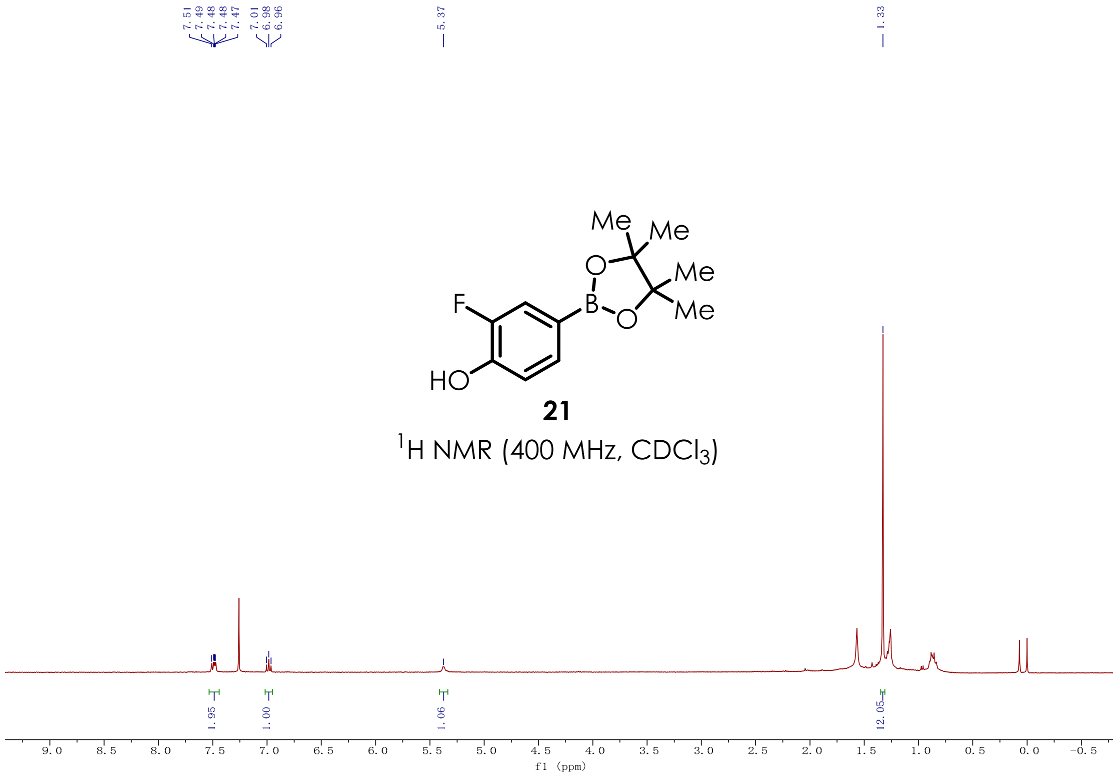
7.51
7.49
7.48
7.48
7.47
7.01
6.98
6.96
5.37
1.33
Me
Me
Me
Me
O
O
B
F
HO
21
1H NMR (400 MHz, CDCl3)
1.95
1.00
1.06
12.05
9.0 8.5 8.0 7.5 7.0 6.5 6.0 5.5 5.0 4.5 4.0 3.5 3.0 2.5 2.0 1.5 1.0 0.5 0.0 -0.5
f1 (ppm)

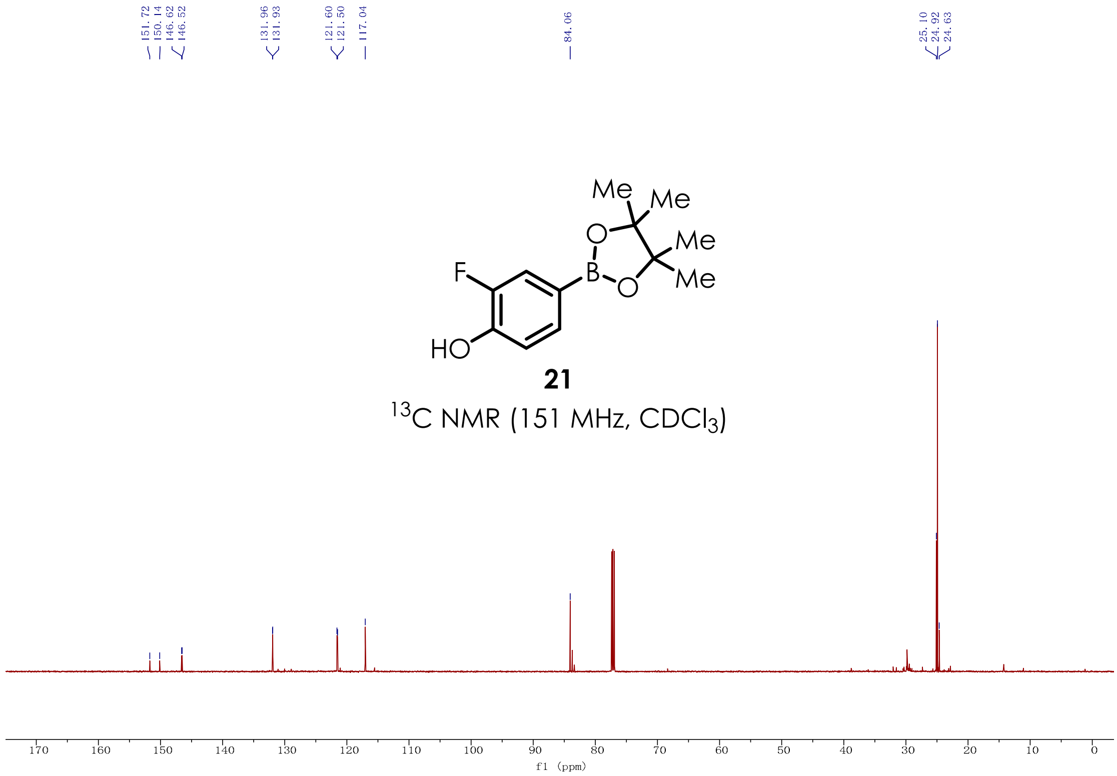
151.72
150.14
146.62
146.52
131.96
131.93
121.60
121.50
117.04
84.06
25.10
24.92
24.63
Me
Me
Me
Me
O
O
B
F
HO
21
13C NMR (151 MHz, CDCl3)
170 160 150 140 130 120 110 100 90 80 70 60 50 40 30 20 10 0
f1 (ppm)

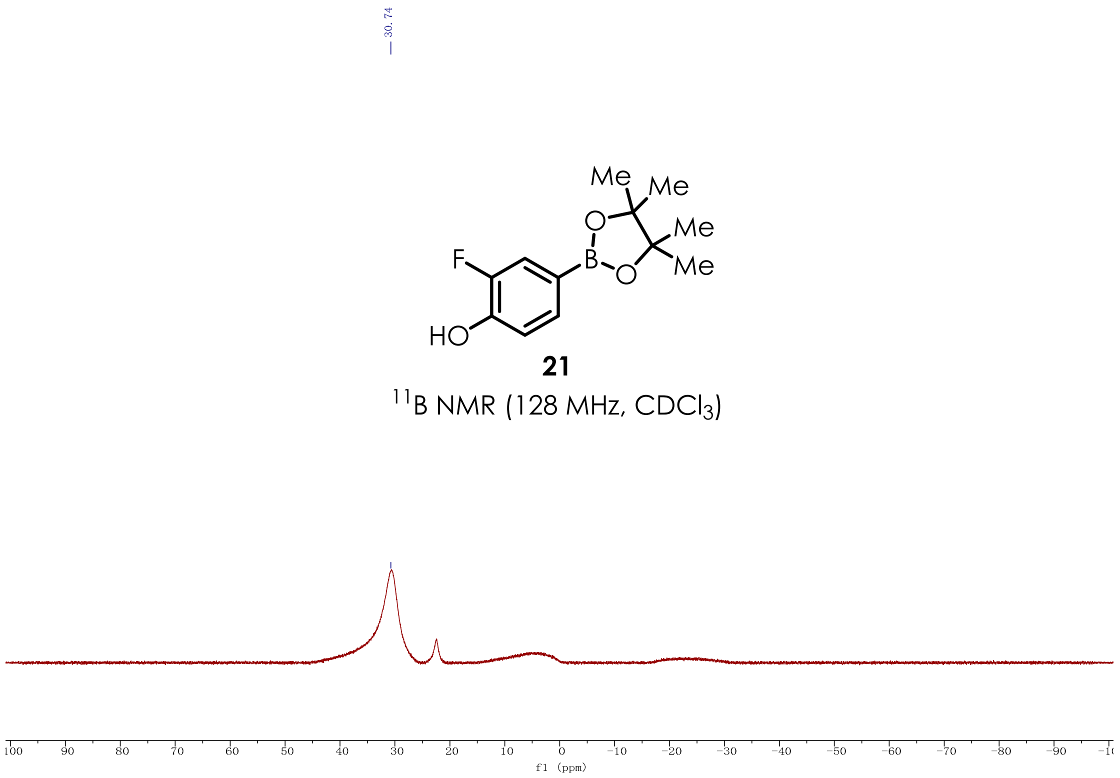

21

^{11}B NMR (128 MHz, $CDCl_3$)

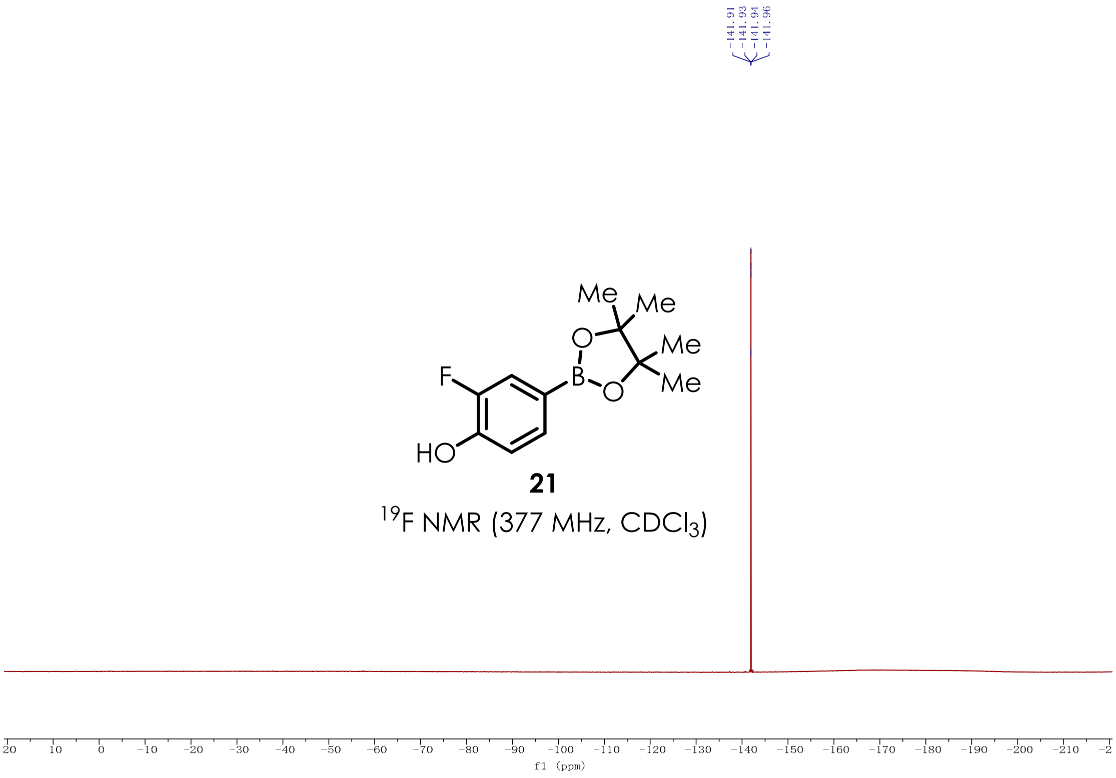

21

^{19}F NMR (377 MHz, $CDCl_3$)

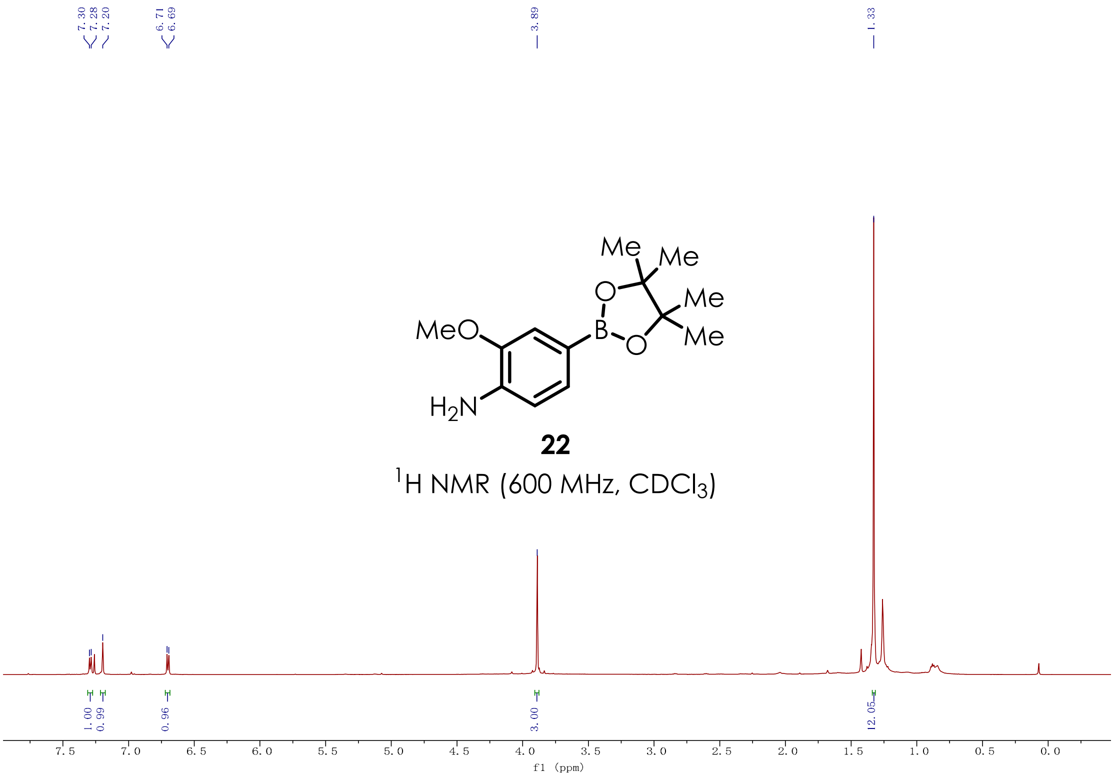

22

^{1}H NMR (600 MHz, CDCl$_3$)

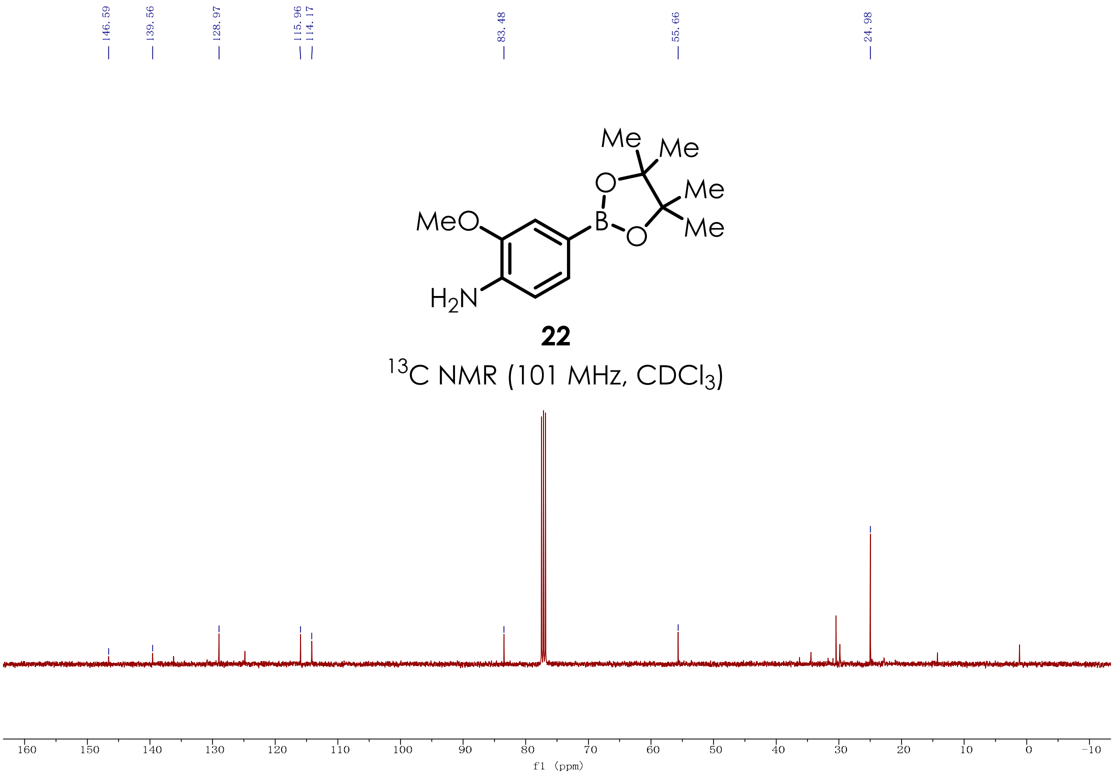

22

^{13}C NMR (101 MHz, CDCl$_3$)

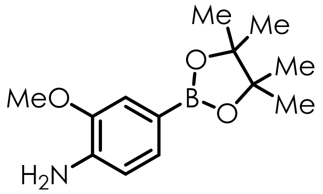

22

^{11}B NMR (193 MHz, $CDCl_3$)

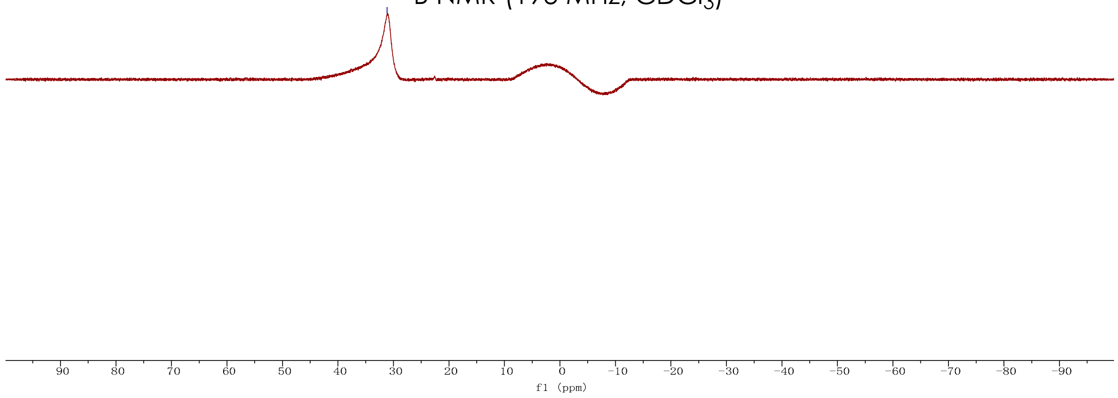

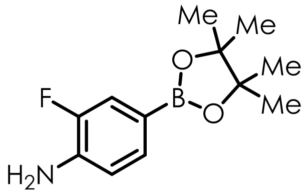

23

^{1}H NMR (400 MHz, $CDCl_3$)

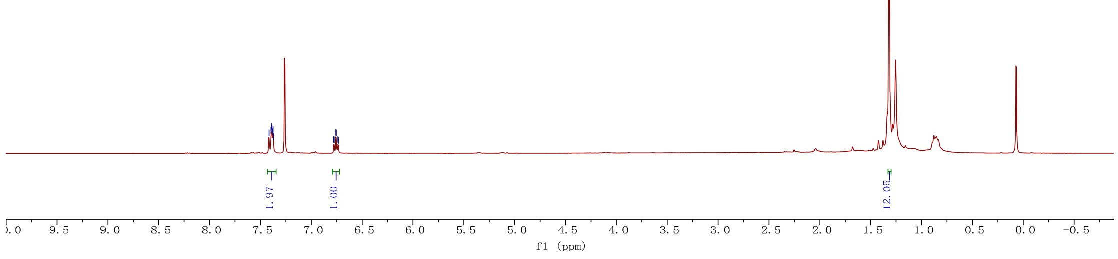

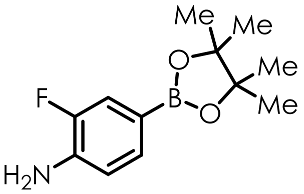

23

^{13}C NMR (151 MHz, $CDCl_3$)

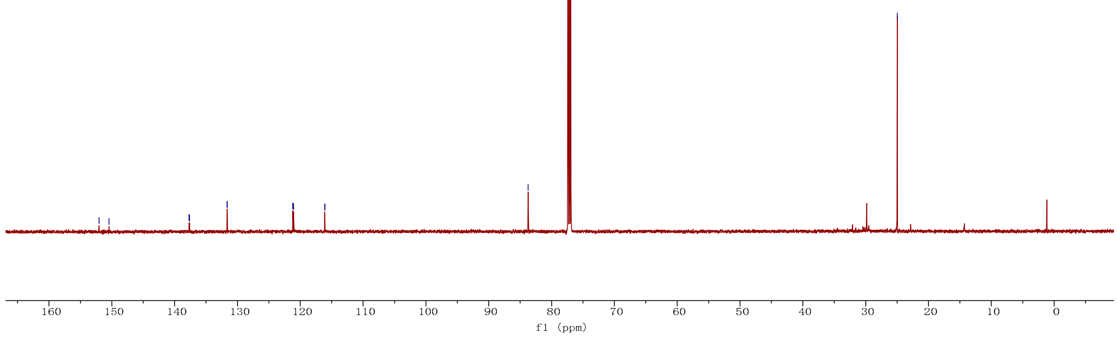

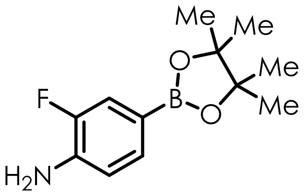

23

^{11}B NMR (128 MHz, $CDCl_3$)

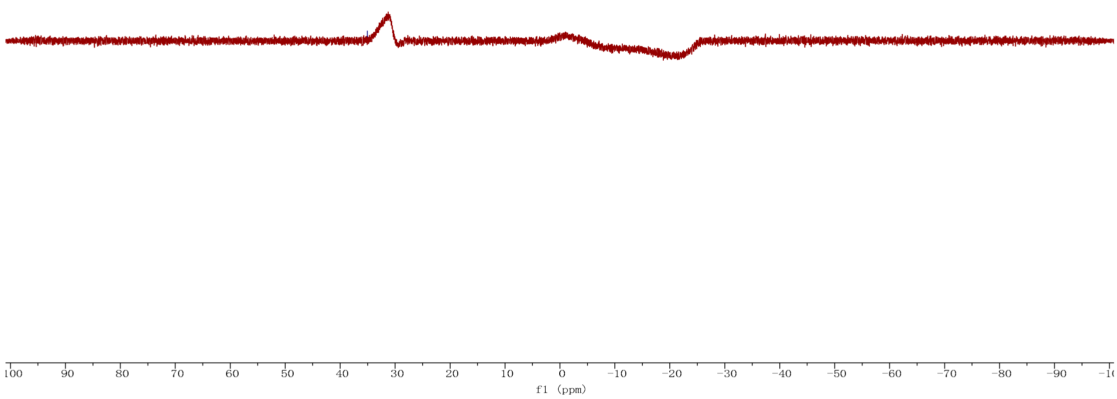

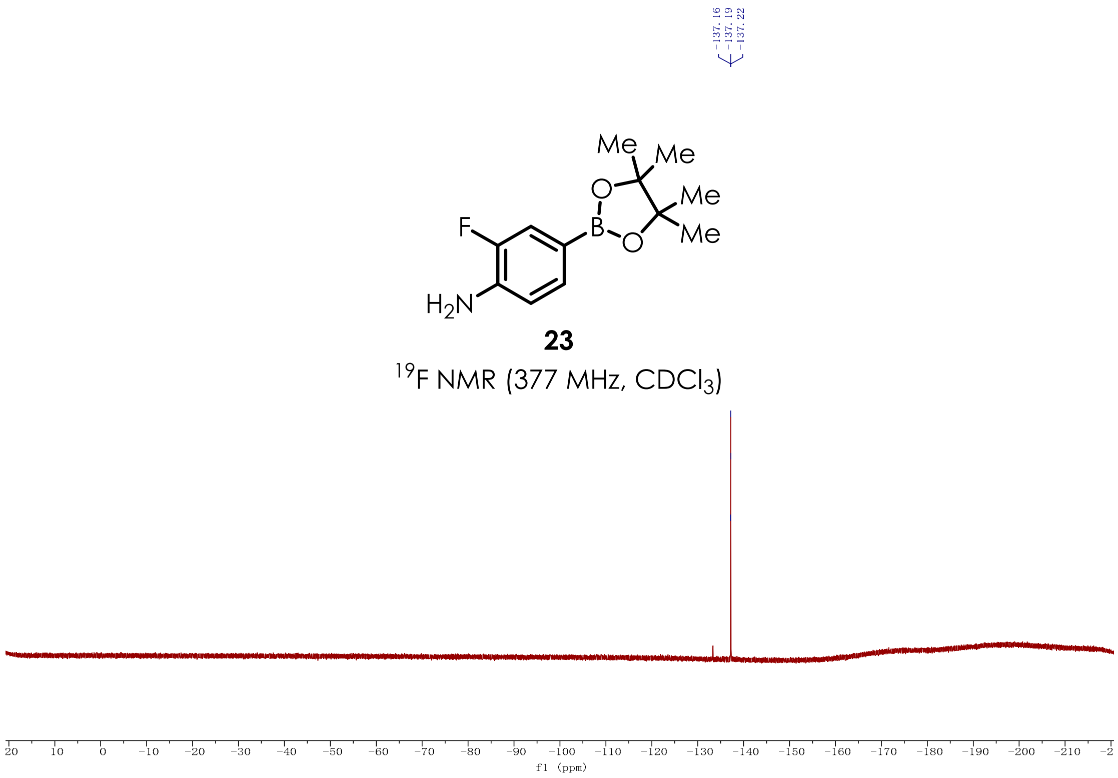
-137.16
-137.19
-137.22
Me
Me
O
Me
F
B
O
Me
H2N
23
19F NMR (377 MHz, CDCl3)
f1 (ppm)

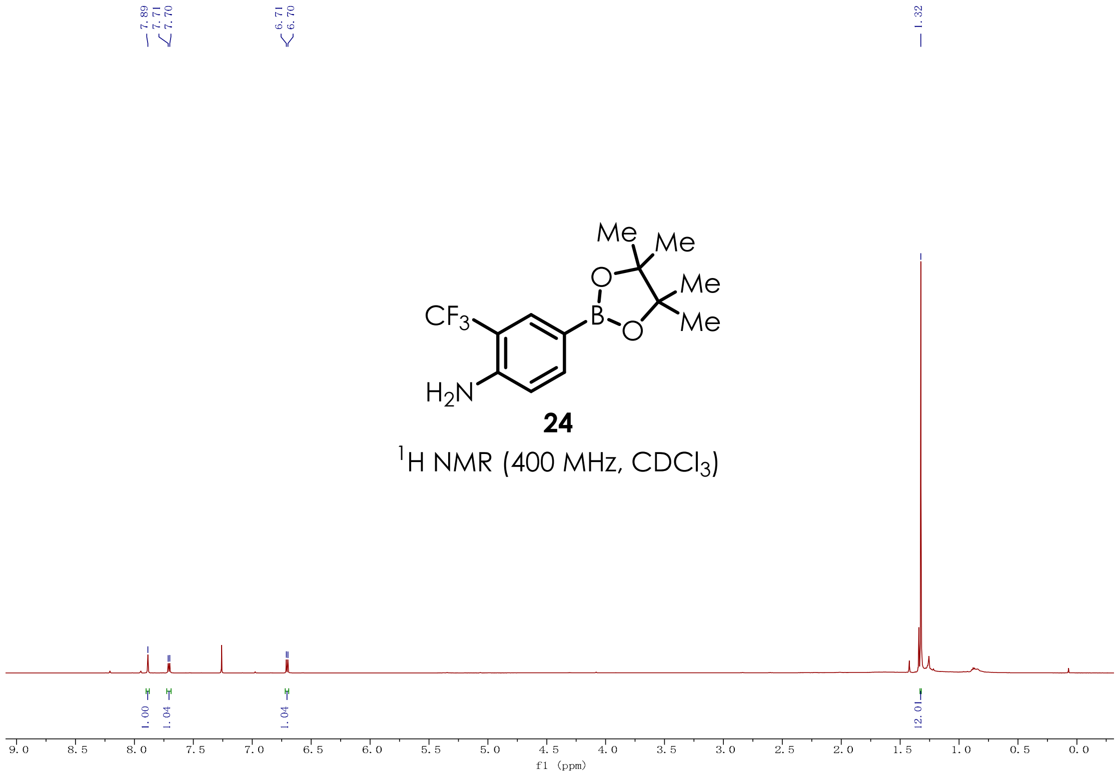
7.89
7.71
7.70
6.71
6.70
1.32
Me
Me
O
Me
CF3
B
O
Me
H2N
24
1H NMR (400 MHz, CDCl3)
1.00
1.04
1.04
12.01
f1 (ppm)

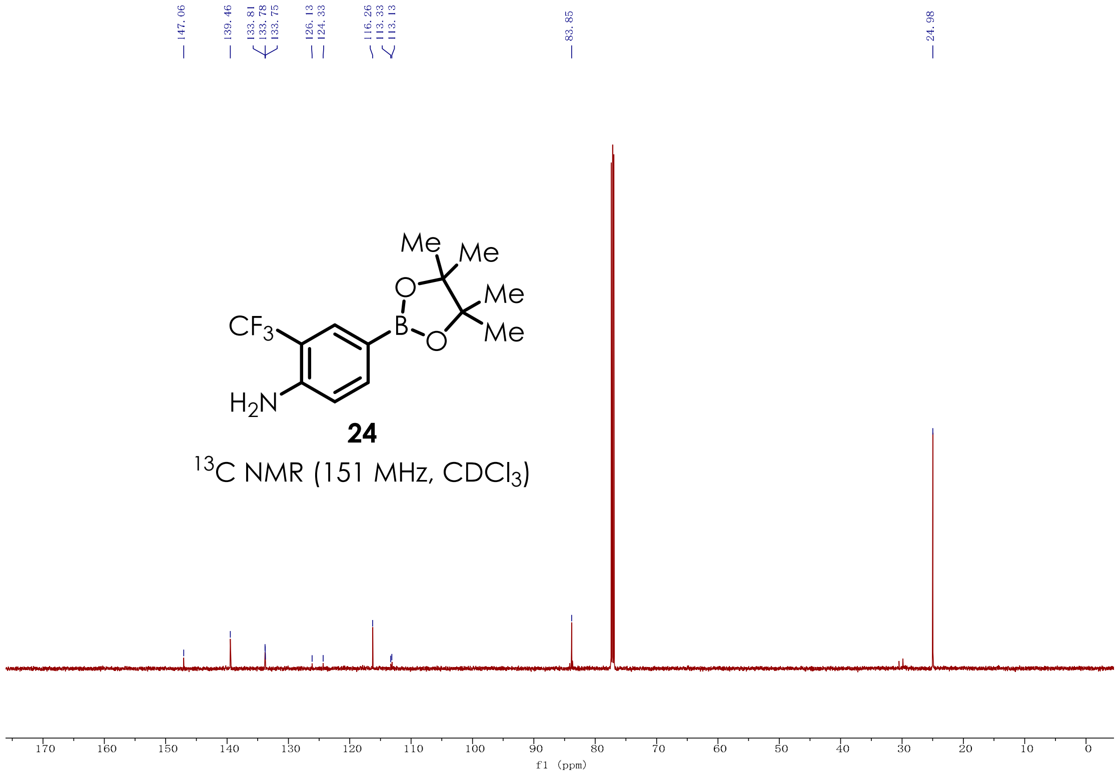
147.06
139.46
133.81
133.78
133.75
126.13
124.33
116.26
113.33
113.13
83.85
24.98
Me
Me
Me
Me
O
O
B
CF3
H2N
24
13C NMR (151 MHz, CDCl3)
f1 (ppm)

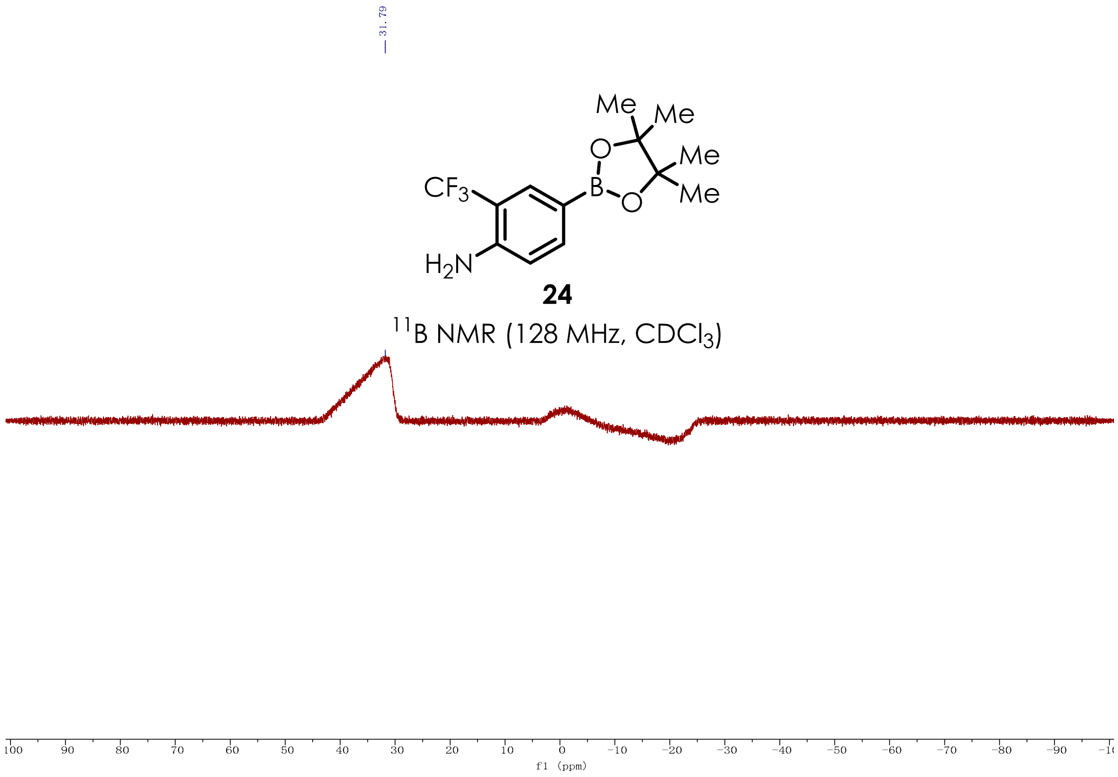
31.79
Me
Me
Me
Me
O
O
B
CF3
H2N
24
11B NMR (128 MHz, CDCl3)
f1 (ppm)

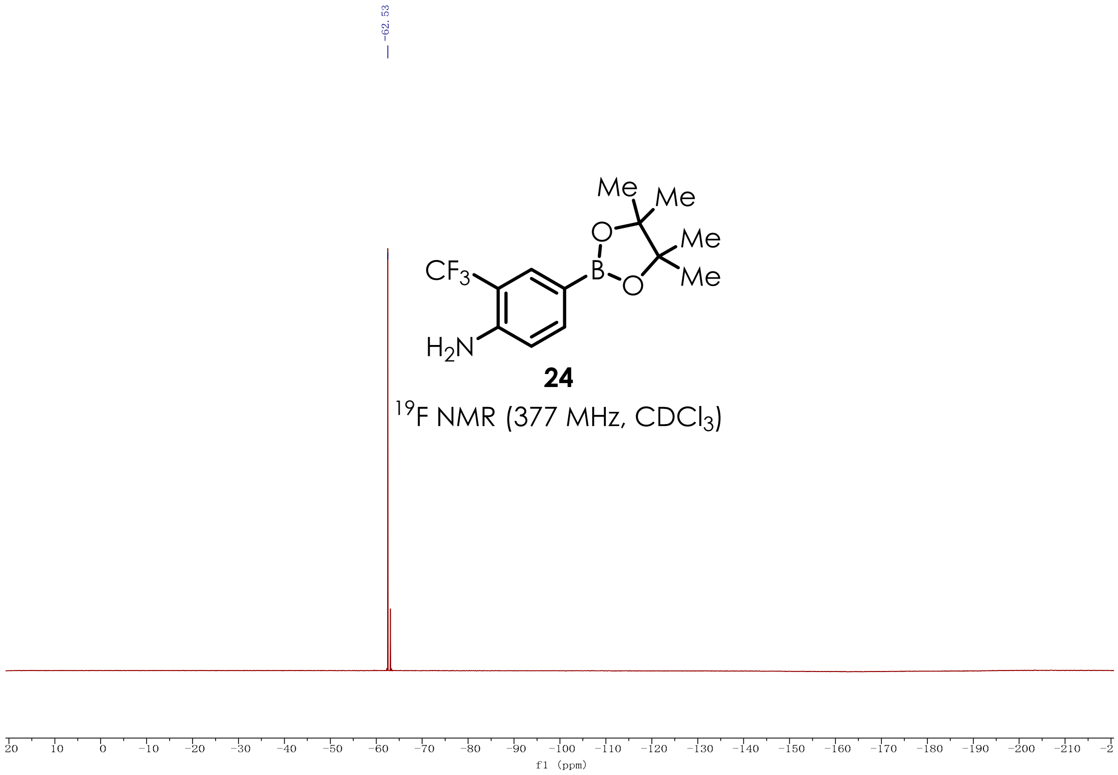
-62.53
CF3
H2N
Me
O
B
24
19F NMR (377 MHz, CDCl3)
f1 (ppm)

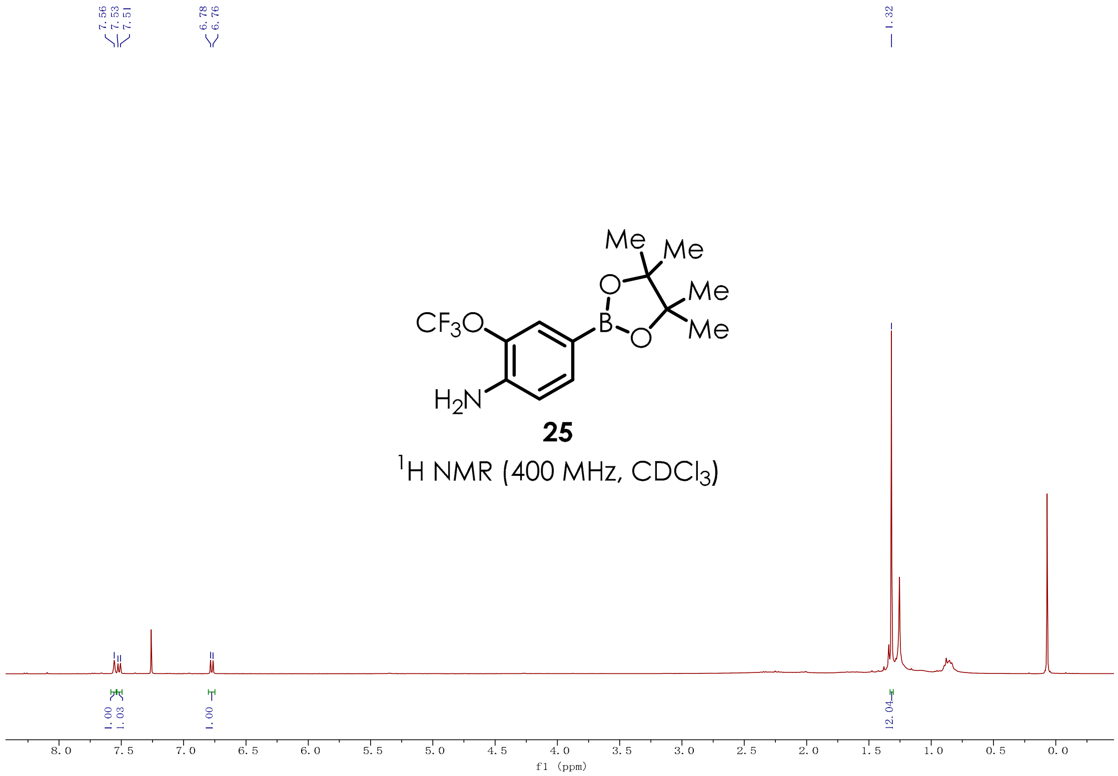
7.56
7.53
7.51
6.78
6.76
1.32
CF3O
H2N
Me
O
B
25
1H NMR (400 MHz, CDCl3)
1.00
1.03
1.00
12.04
f1 (ppm)

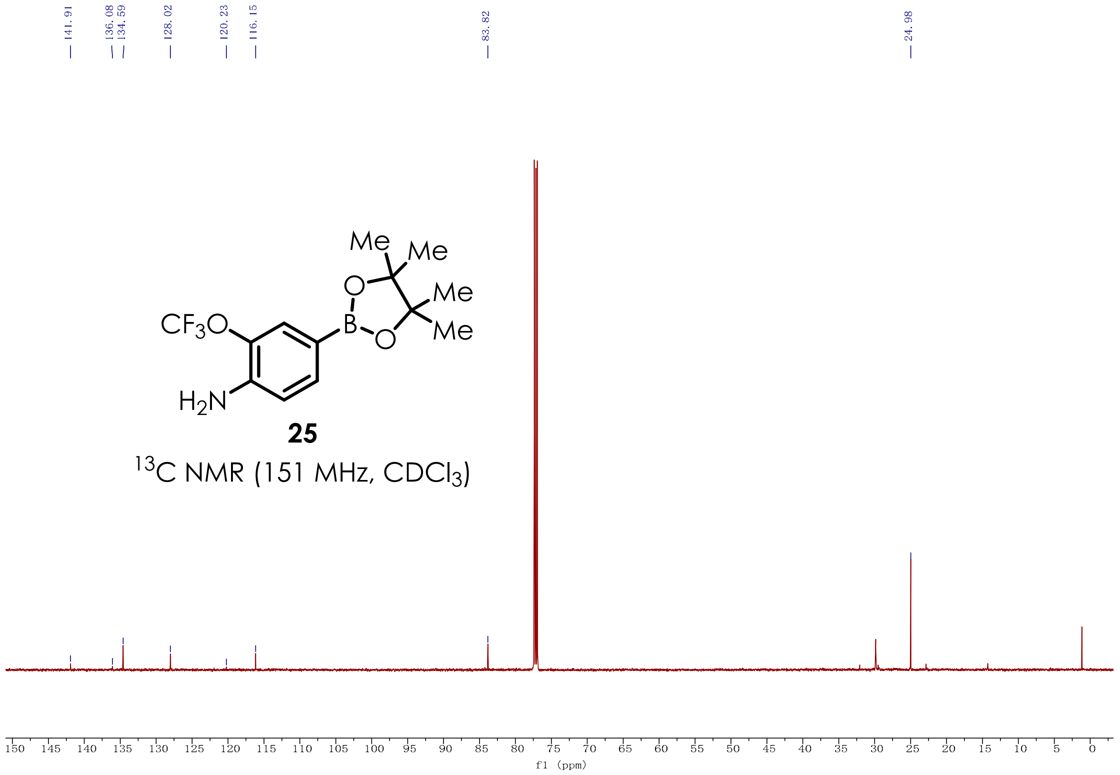
141.91
136.08
134.59
128.02
120.23
116.15
83.82
24.98
CF3O
H2N
Me
Me
Me
Me
O
B
O
25
13C NMR (151 MHz, CDCl3)
150 145 140 135 130 125 120 115 110 105 100 95 90 85 80 75 70 65 60 55 50 45 40 35 30 25 20 15 10 5 0
f1 (ppm)

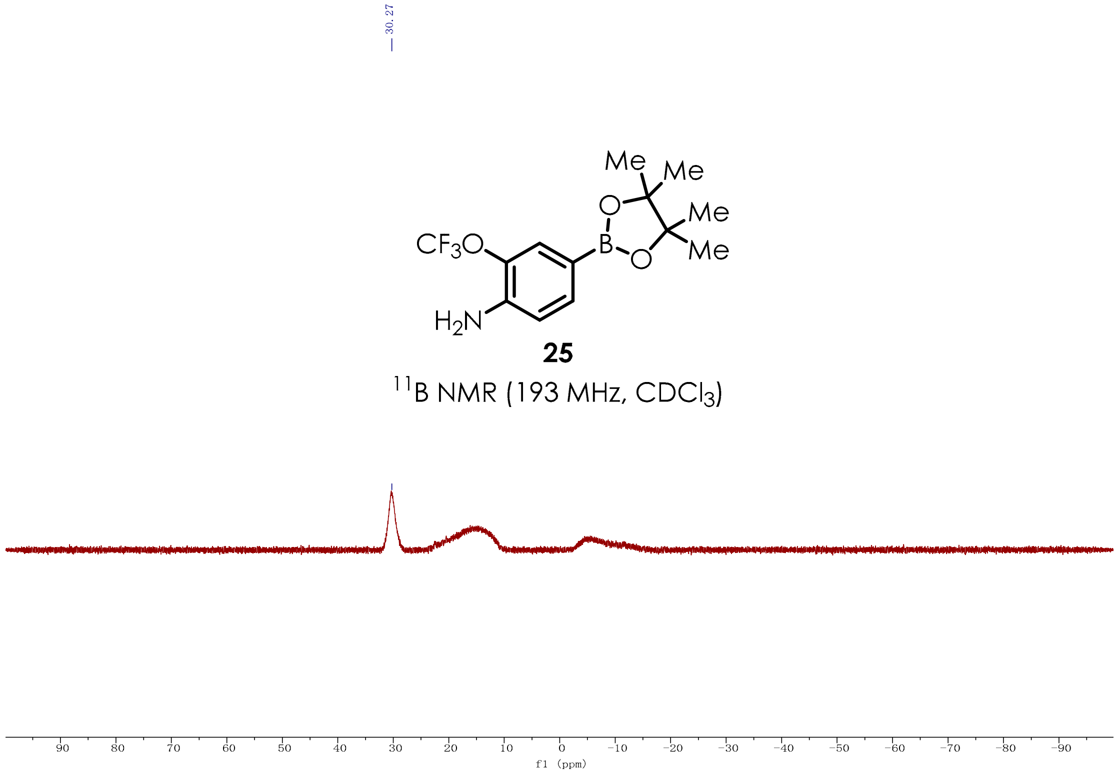
30.27
CF3O
H2N
Me
Me
Me
Me
O
B
O
25
11B NMR (193 MHz, CDCl3)
90 80 70 60 50 40 30 20 10 0 -10 -20 -30 -40 -50 -60 -70 -80 -90
f1 (ppm)

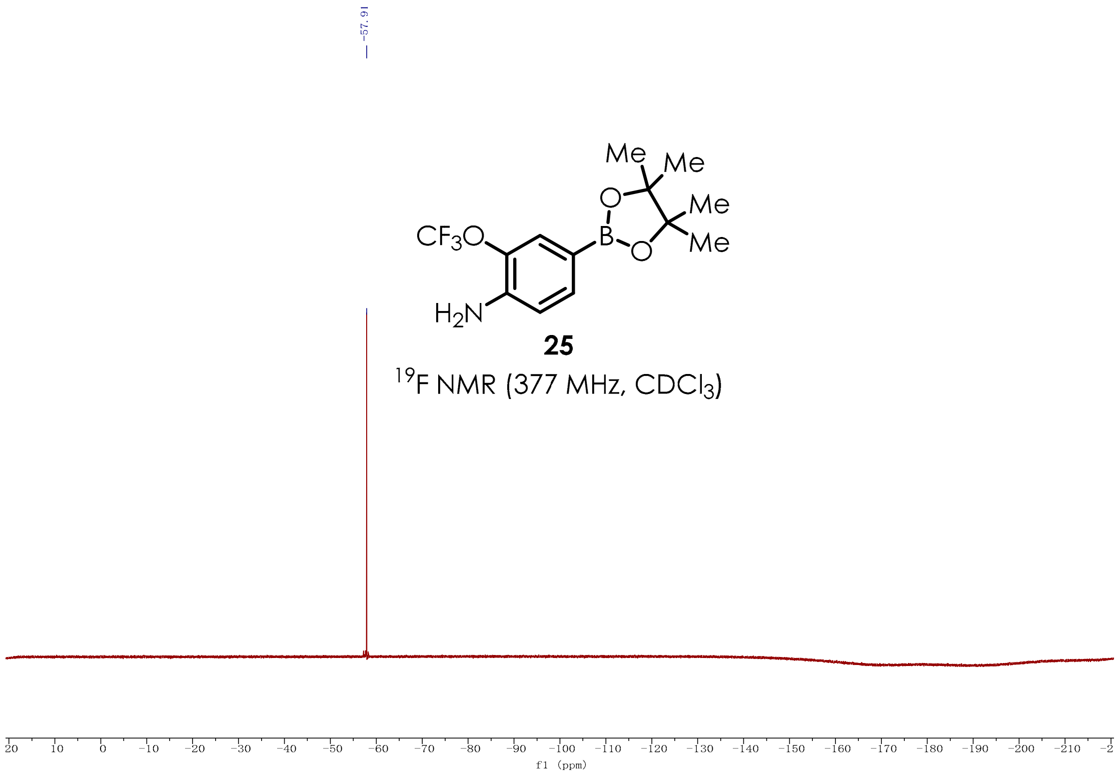
-57.91
Me
Me
O
Me
CF_3O
B
O
Me
H_2N
25
^{19}F NMR (377 MHz, $CDCl_3$)
20 10 0 -10 -20 -30 -40 -50 -60 -70 -80 -90 -100 -110 -120 -130 -140 -150 -160 -170 -180 -190 -200 -210 -2
f1 (ppm)

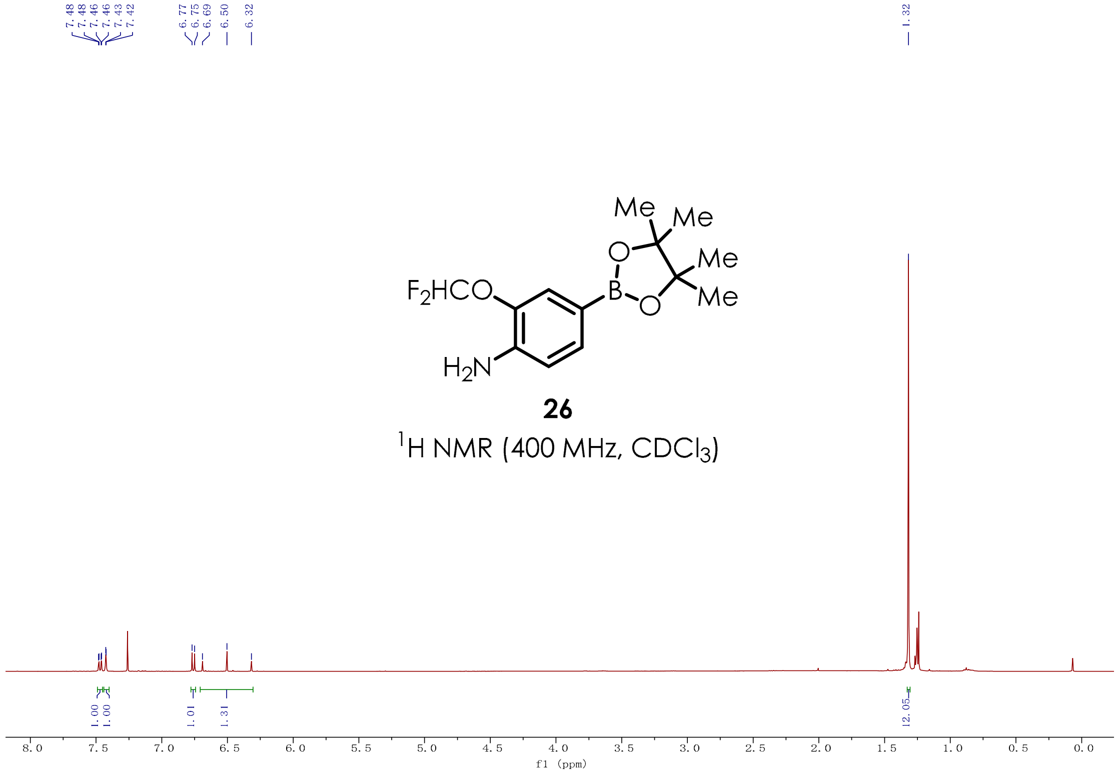
7.48 7.48 7.46 7.43 7.42
6.77 6.75 6.69 6.50 6.32
1.32
Me
Me
O
Me
F_2HCO
B
O
Me
H_2N
26
^1H NMR (400 MHz, $CDCl_3$)
1.00 1.00 1.01 1.31 12.05
8.0 7.5 7.0 6.5 6.0 5.5 5.0 4.5 4.0 3.5 3.0 2.5 2.0 1.5 1.0 0.5 0.0
f1 (ppm)

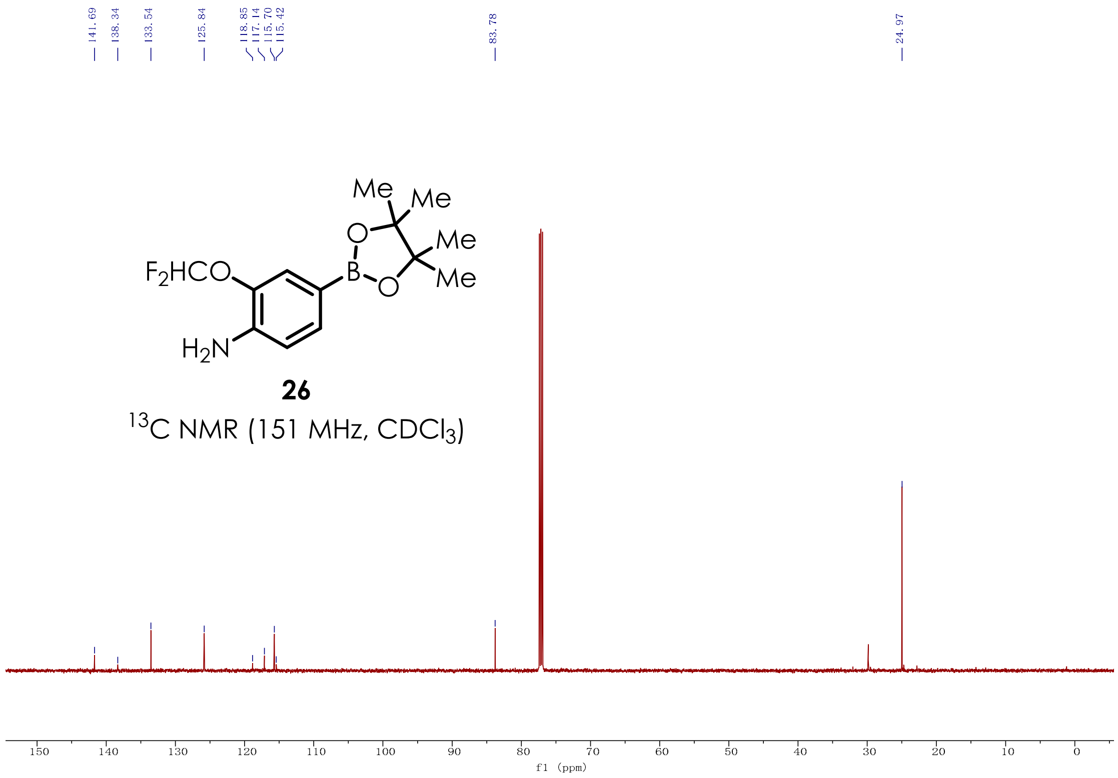
141.69
138.34
133.54
125.84
118.85
117.14
115.70
115.42
83.78
24.97
Me
Me
O
Me
F2HCO
B
O
Me
H2N
26
13C NMR (151 MHz, CDCl3)
150
140
130
120
110
100
90
80
70
60
50
40
30
20
10
0
f1 (ppm)

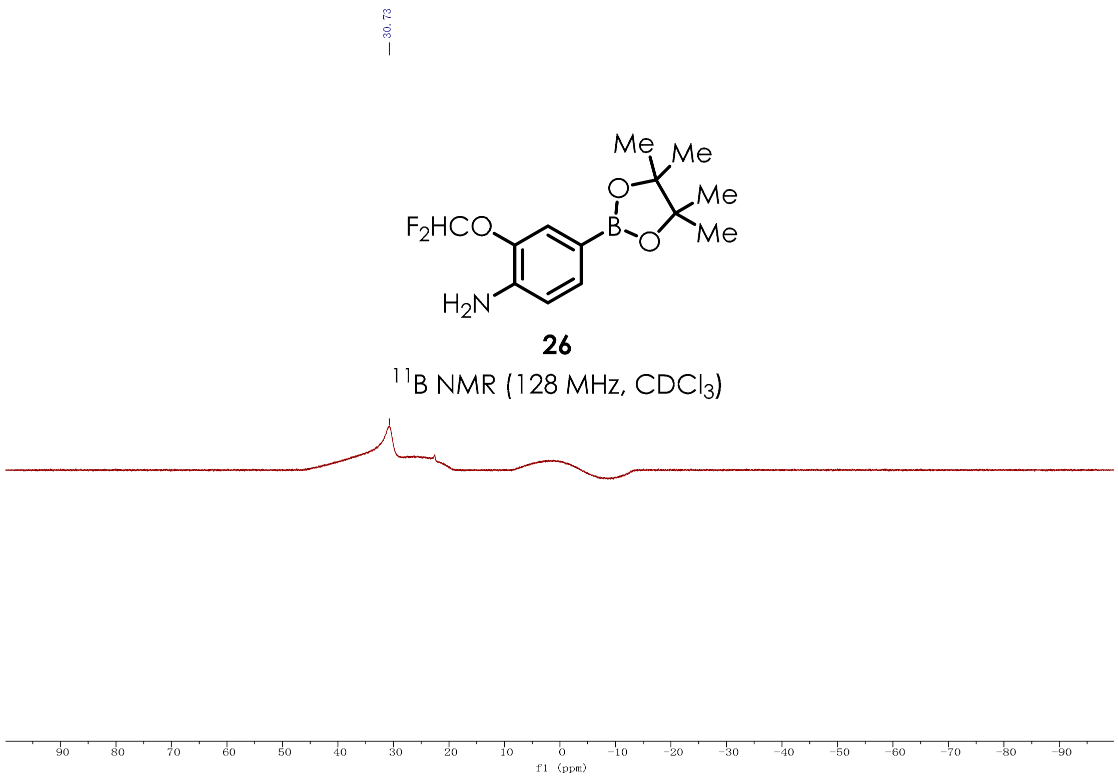
30.73
Me
Me
O
Me
F2HCO
B
O
Me
H2N
26
11B NMR (128 MHz, CDCl3)
90
80
70
60
50
40
30
20
10
0
-10
-20
-30
-40
-50
-60
-70
-80
-90
f1 (ppm)

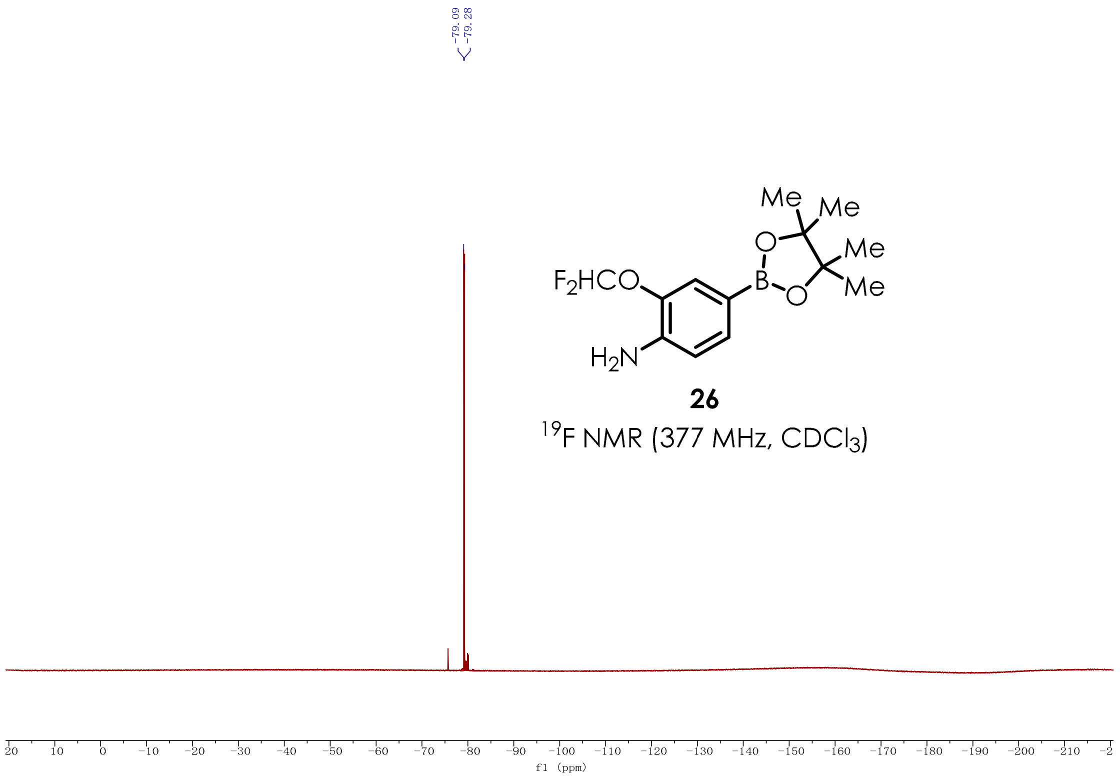
-79.09
-79.28
Me
Me
Me
Me
F2HCO
H2N
O
O
B
26
19F NMR (377 MHz, CDCl3)
f1 (ppm)

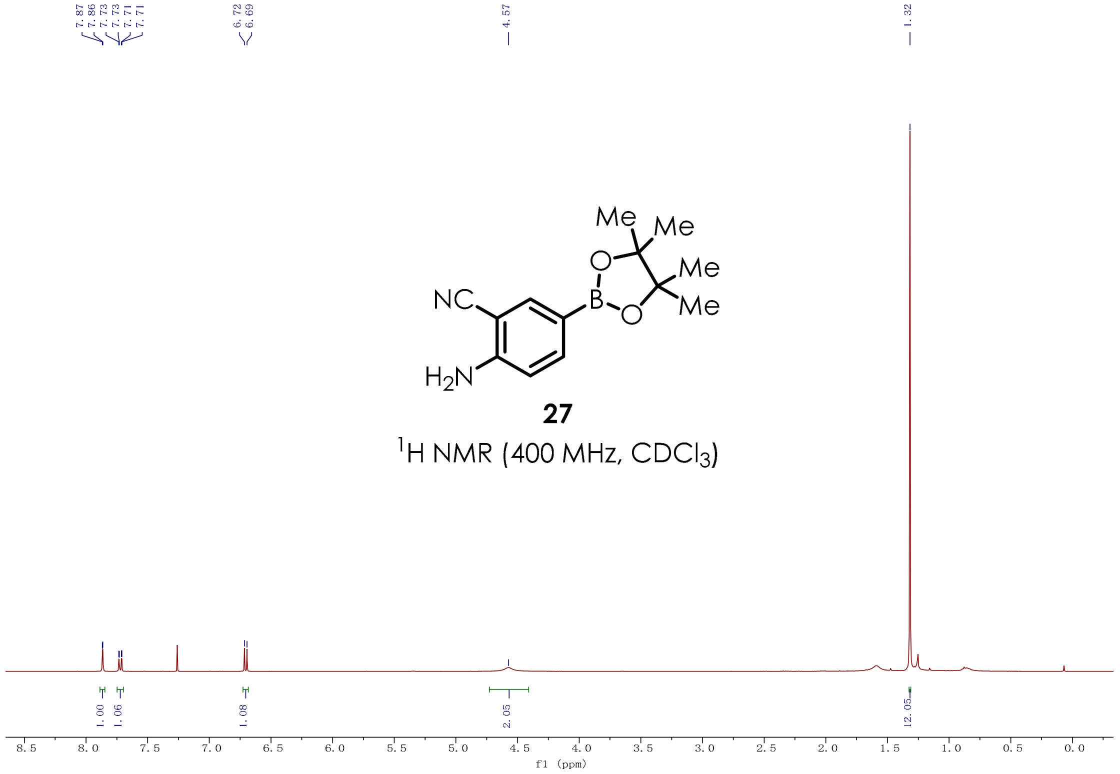
7.87
7.86
7.73
7.73
7.71
7.71
6.72
6.69
4.57
1.32
Me
Me
Me
Me
NC
H2N
O
O
B
27
1H NMR (400 MHz, CDCl3)
1.00
1.06
1.08
2.05
12.05
f1 (ppm)

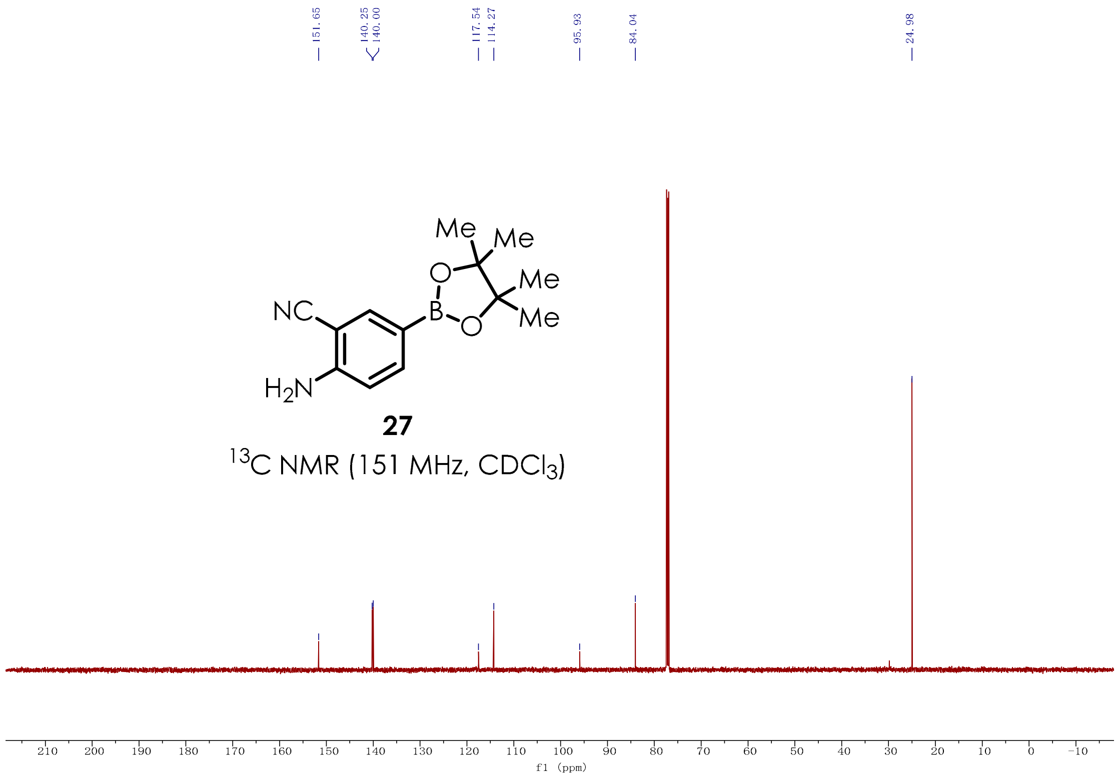
151.65
140.25
140.00
117.54
114.27
95.93
84.04
24.98
NC
H2N
Me
Me
Me
Me
O
B
O
27
13C NMR (151 MHz, CDCl3)
f1 (ppm)

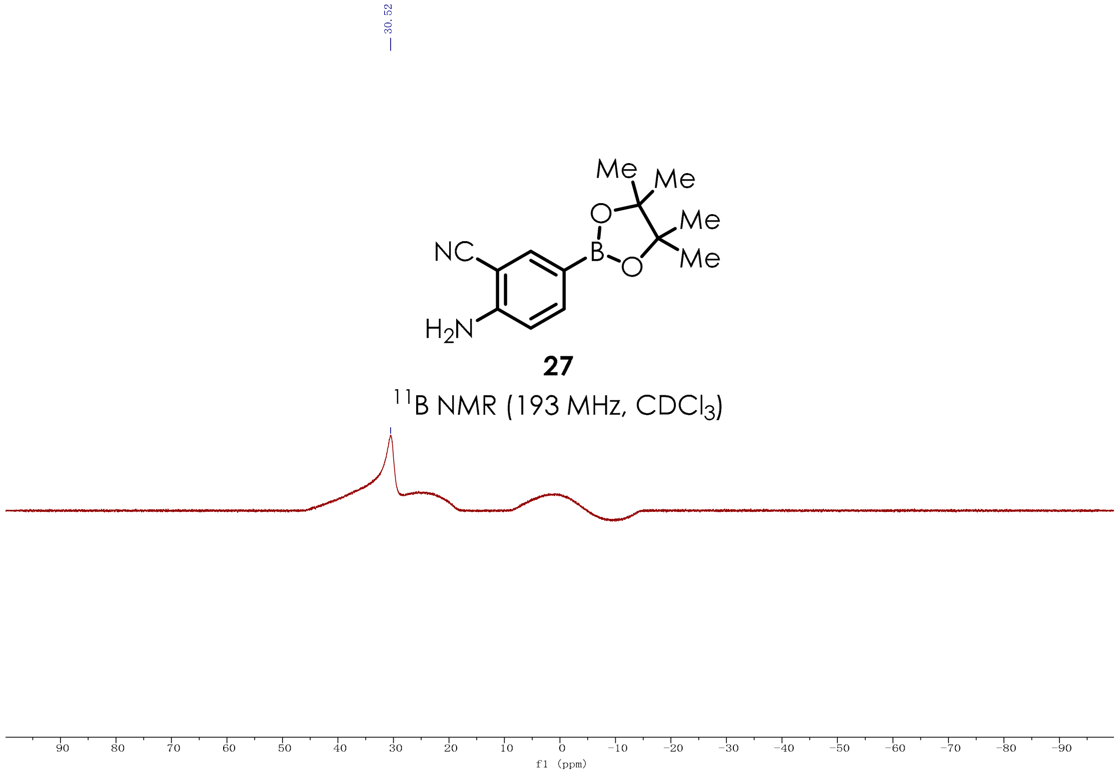
30.52
NC
H2N
Me
Me
Me
Me
O
B
O
27
11B NMR (193 MHz, CDCl3)
f1 (ppm)

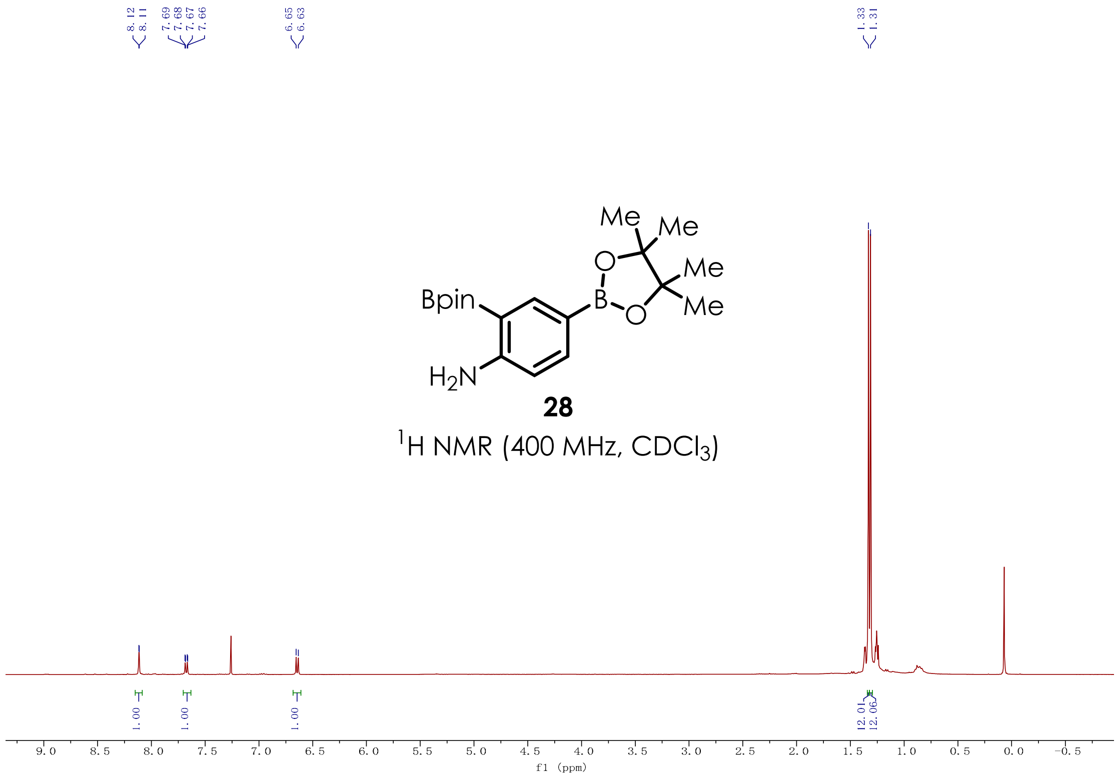
8.12
8.11
7.69
7.68
7.67
7.66
6.65
6.63
1.33
1.31
Me
Me
Me
Me
O
B
O
Bpin
H2N
28
1H NMR (400 MHz, CDCl3)
1.00
1.00
1.00
12.01
12.06
f1 (ppm)

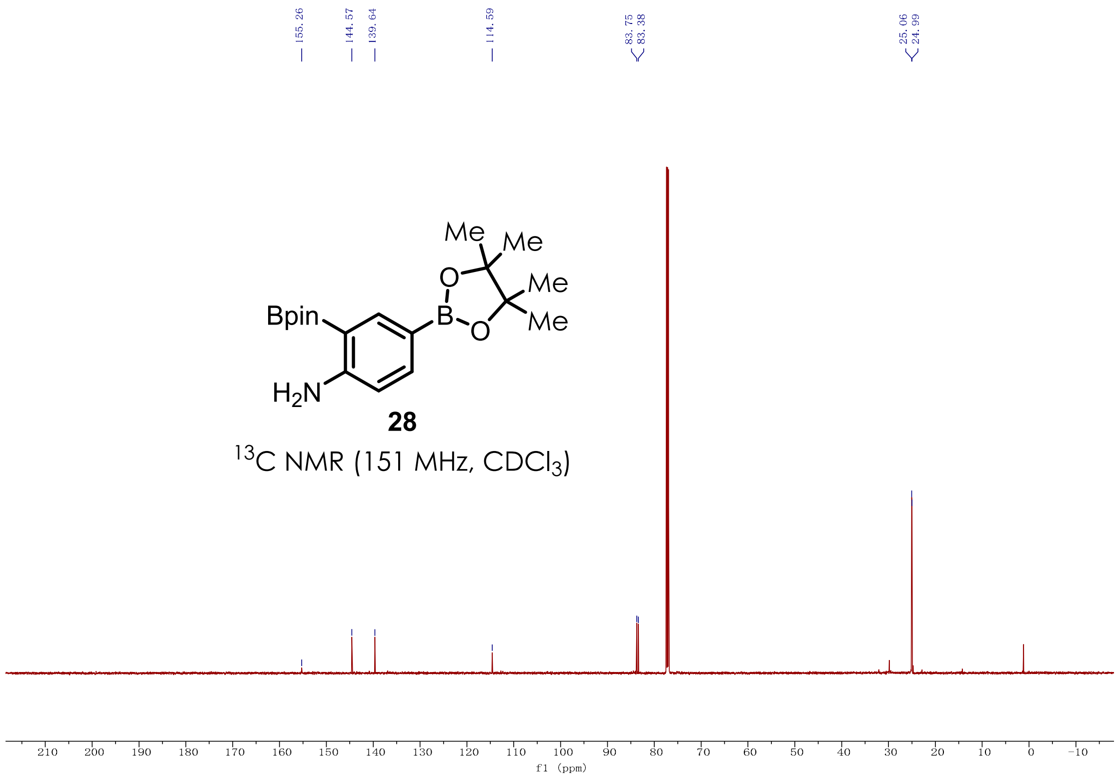
155.26
144.57
139.64
114.59
83.75
83.38
25.06
24.99
Me
Me
Me
Me
O
B
O
Bpin
H2N
28
13C NMR (151 MHz, CDCl3)
f1 (ppm)

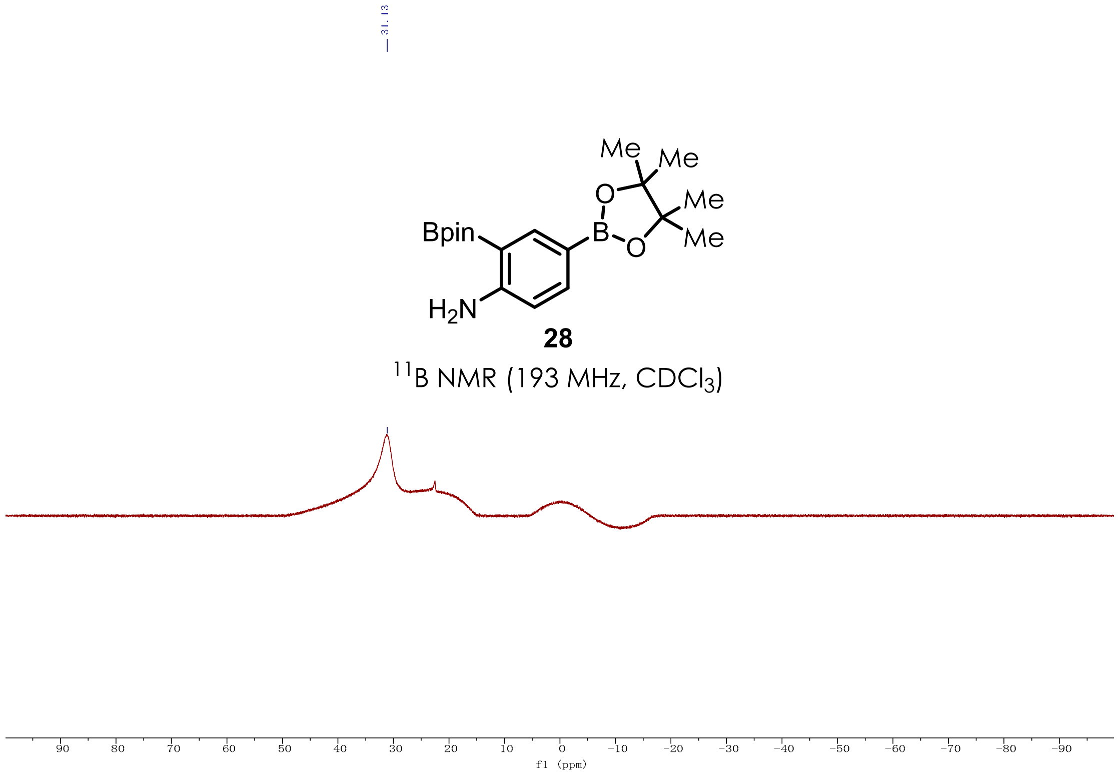
31.13
Bpin
H2N
Me
O
B
28
11B NMR (193 MHz, CDCl3)
f1 (ppm)

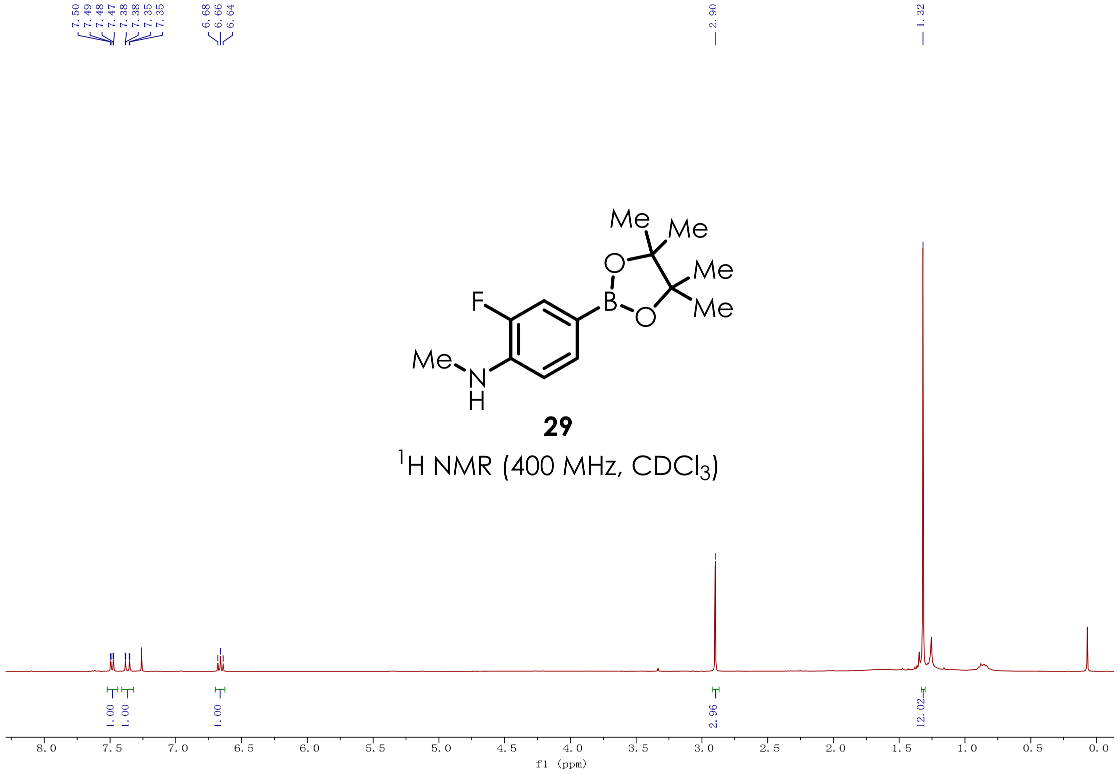
7.50
7.49
7.48
7.47
7.38
7.38
7.35
7.35
6.68
6.66
6.64
2.90
1.32
F
Me
N
H
O
B
29
1H NMR (400 MHz, CDCl3)
1.00
1.00
1.00
2.96
12.02
f1 (ppm)

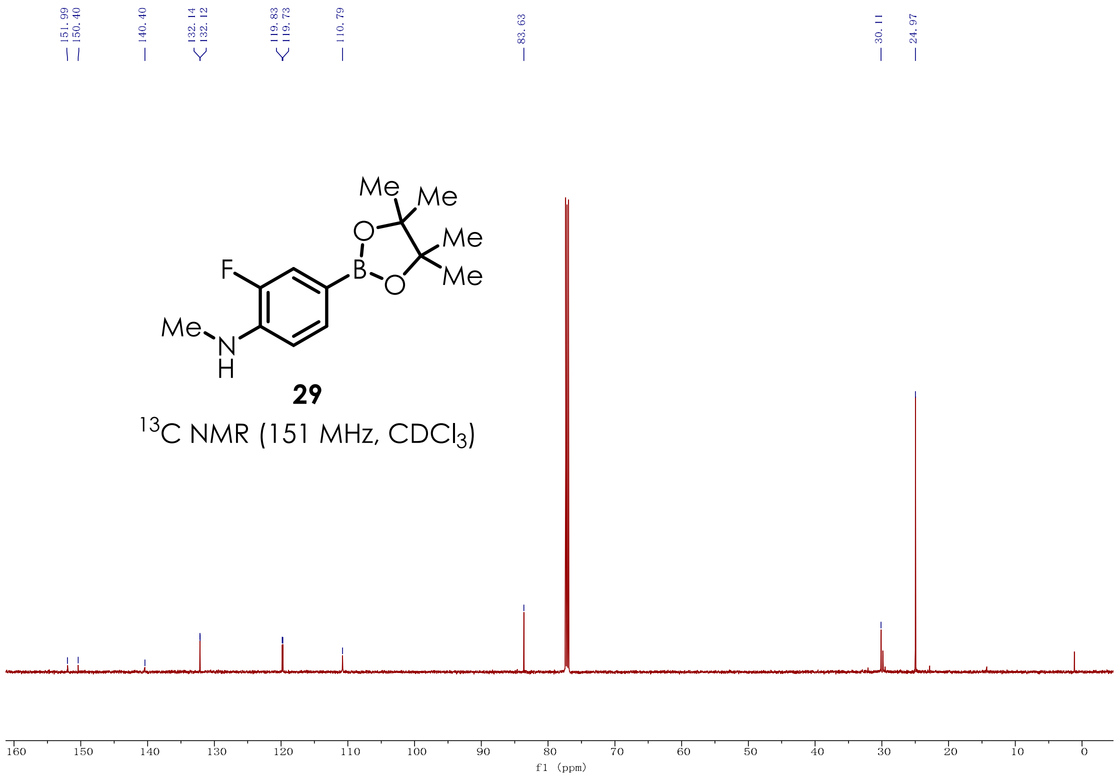
151.99
150.40
140.40
132.14
132.12
119.83
119.73
110.79
83.63
30.11
24.97
F
Me
N
H
B
O
Me
29
^{13}C NMR (151 MHz, $CDCl_3$)
f1 (ppm)

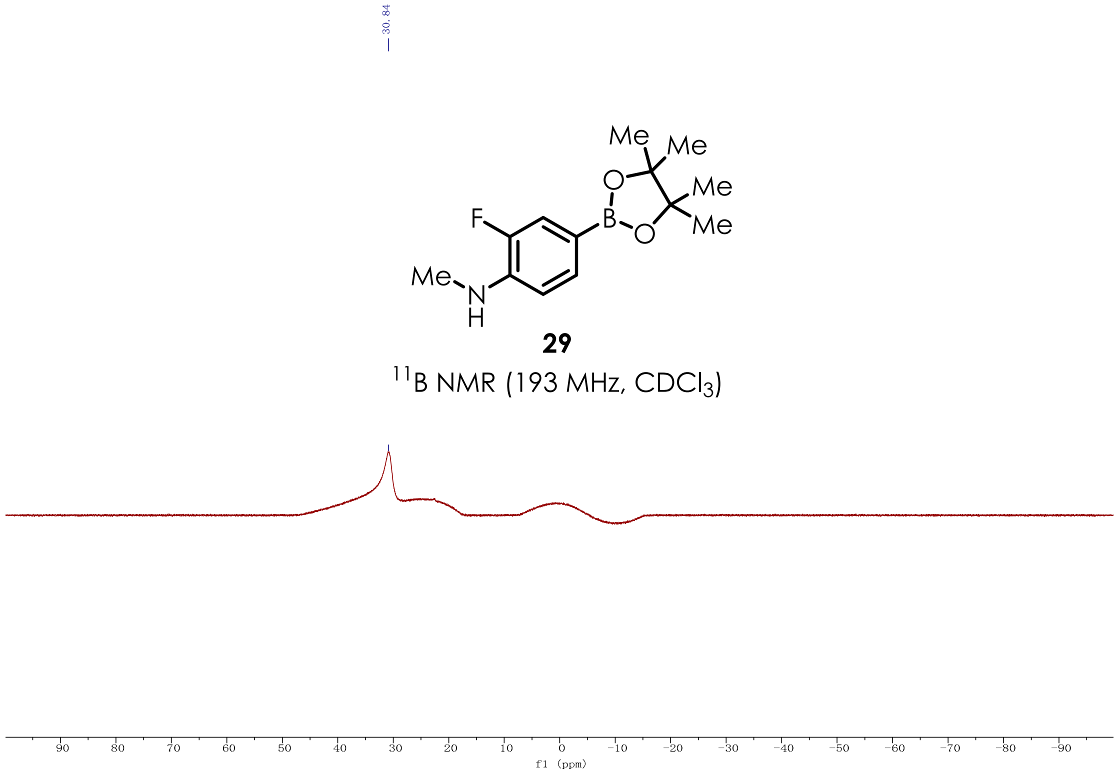
30.84
F
Me
N
H
B
O
Me
29
^{11}B NMR (193 MHz, $CDCl_3$)
f1 (ppm)

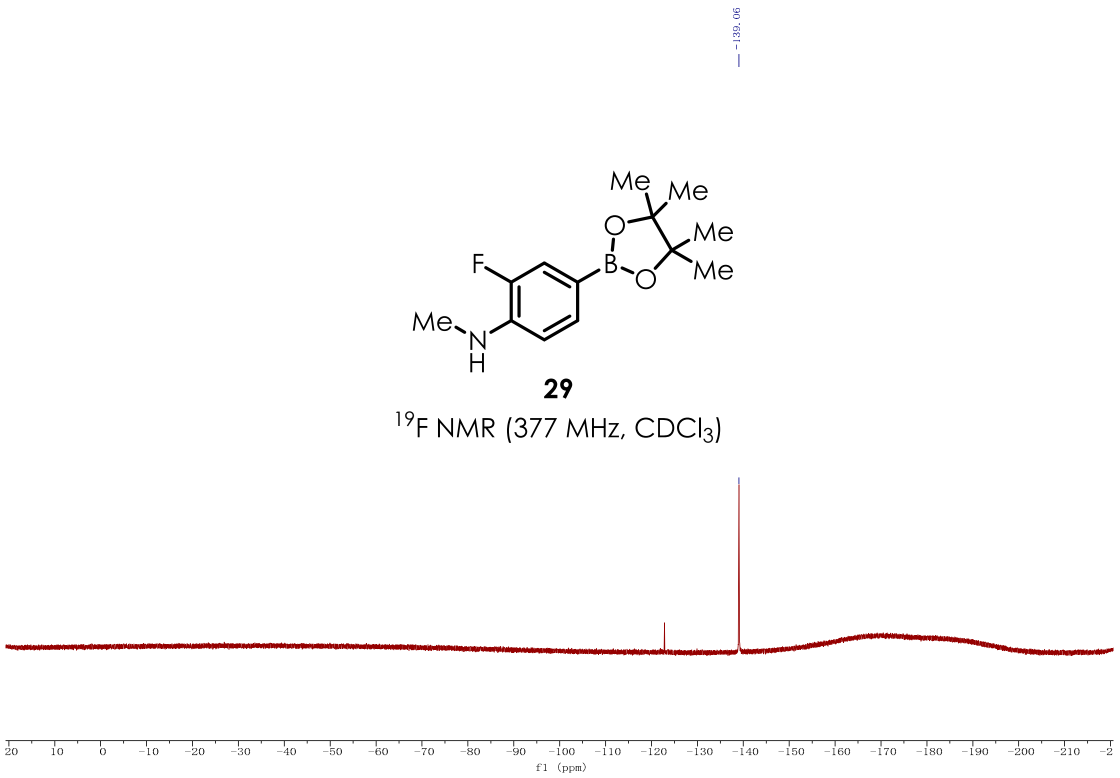
-139.06
Me
Me
Me
Me
F
Me
N
H
O
B
O
29
19F NMR (377 MHz, CDCl3)
20 10 0 -10 -20 -30 -40 -50 -60 -70 -80 -90 -100 -110 -120 -130 -140 -150 -160 -170 -180 -190 -200 -210
f1 (ppm)

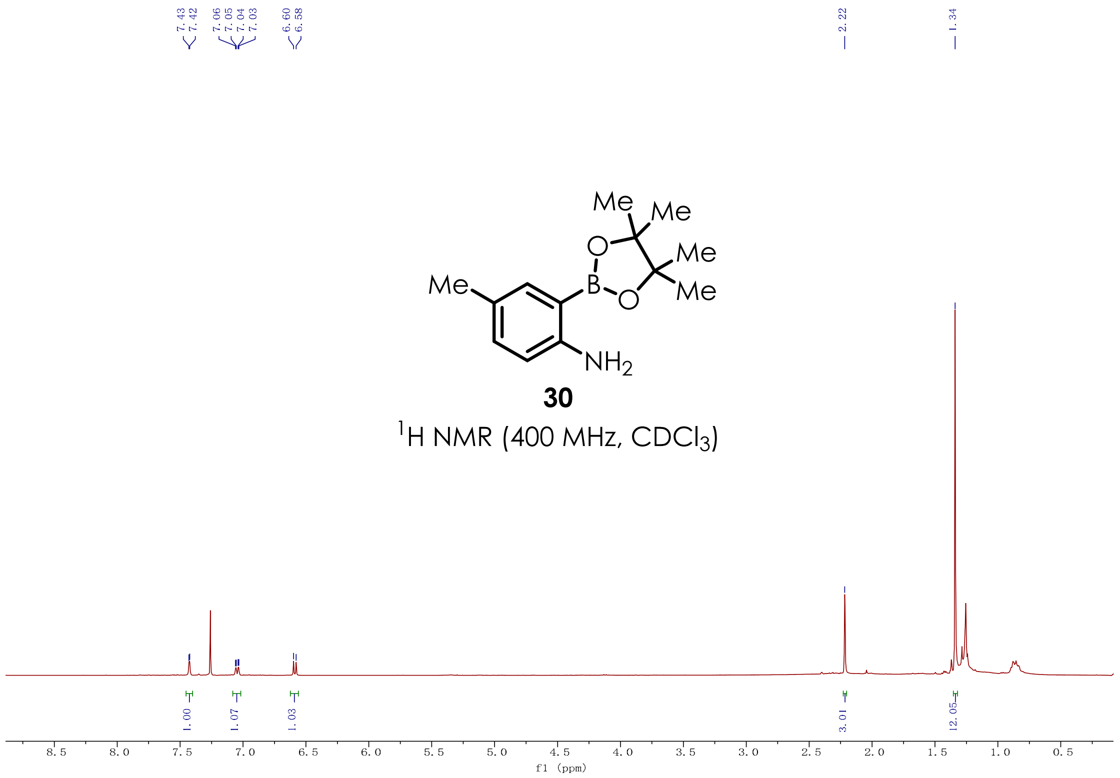
7.43
7.42
7.06
7.05
7.04
7.03
6.60
6.58
2.22
1.34
Me
Me
Me
Me
Me
O
B
O
NH2
30
1H NMR (400 MHz, CDCl3)
1.00
1.07
1.03
3.01
12.05
8.5 8.0 7.5 7.0 6.5 6.0 5.5 5.0 4.5 4.0 3.5 3.0 2.5 2.0 1.5 1.0 0.5
f1 (ppm)

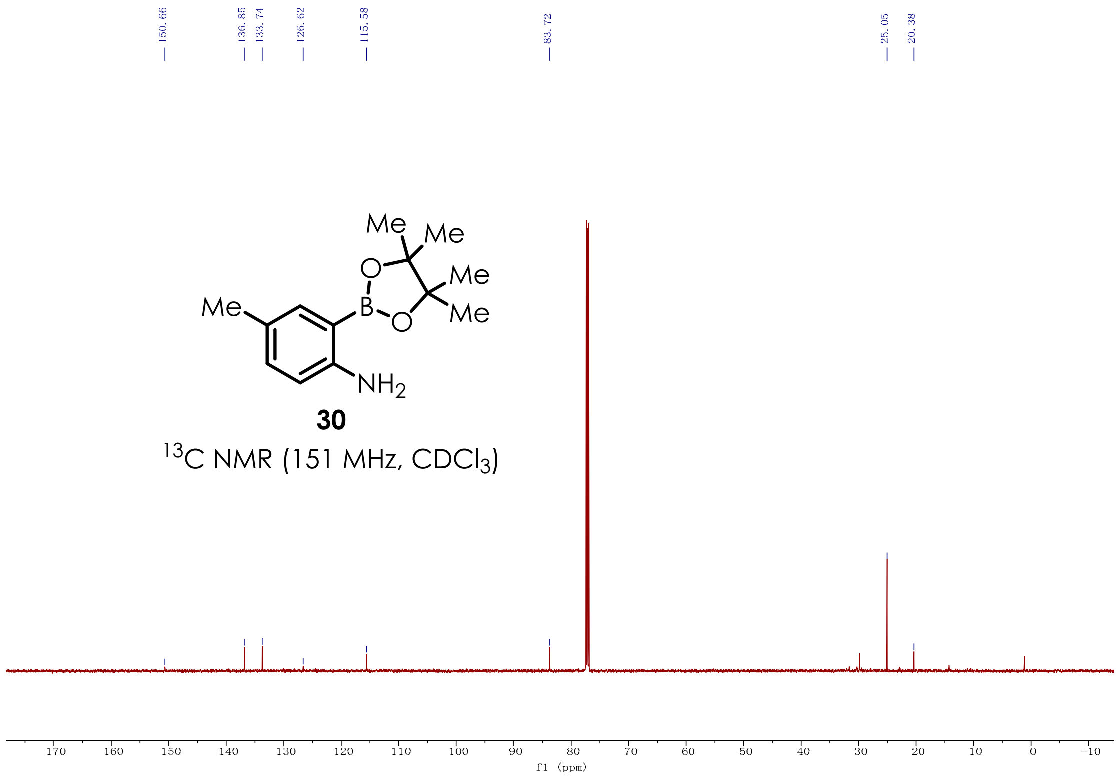

150.66
136.85
133.74
126.62
115.58
83.72
25.05
20.38
Me
Me
Me
Me
Me
O
B
O
NH2
30
13C NMR (151 MHz, CDCl3)
170 160 150 140 130 120 110 100 90 80 70 60 50 40 30 20 10 0 -10
f1 (ppm)

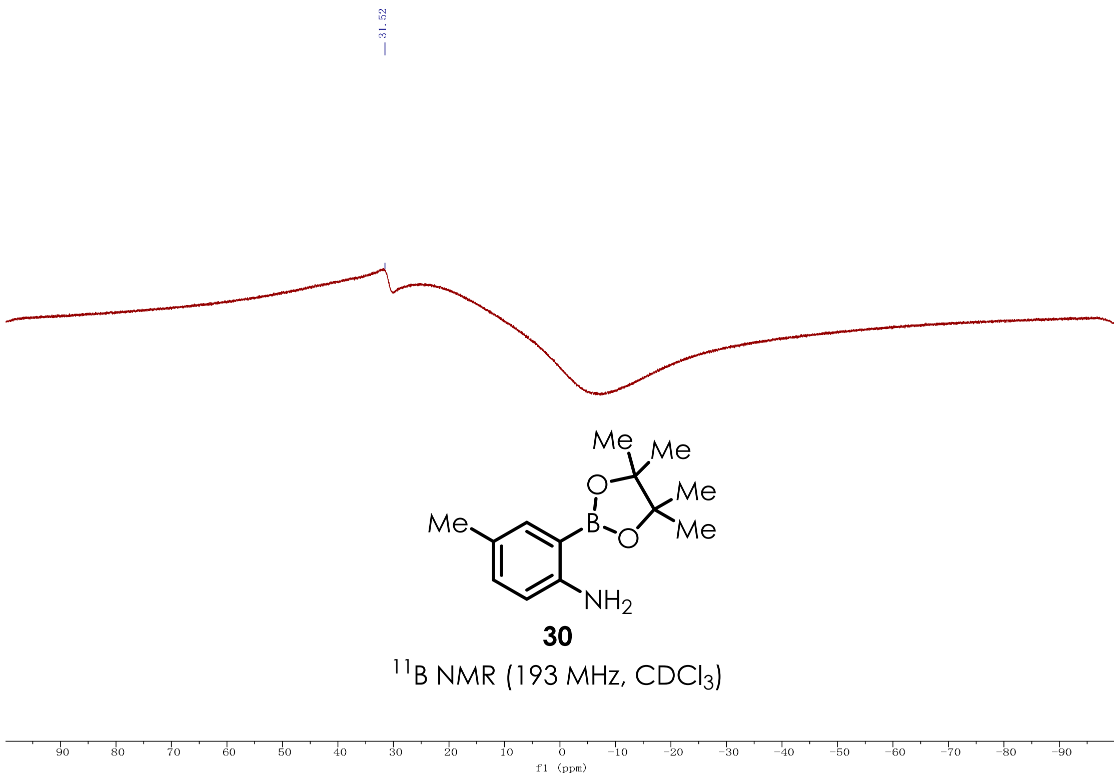

31.52
Me
Me
Me
Me
Me
O
B
O
NH2
30
11B NMR (193 MHz, CDCl3)
90 80 70 60 50 40 30 20 10 0 -10 -20 -30 -40 -50 -60 -70 -80 -90
f1 (ppm)

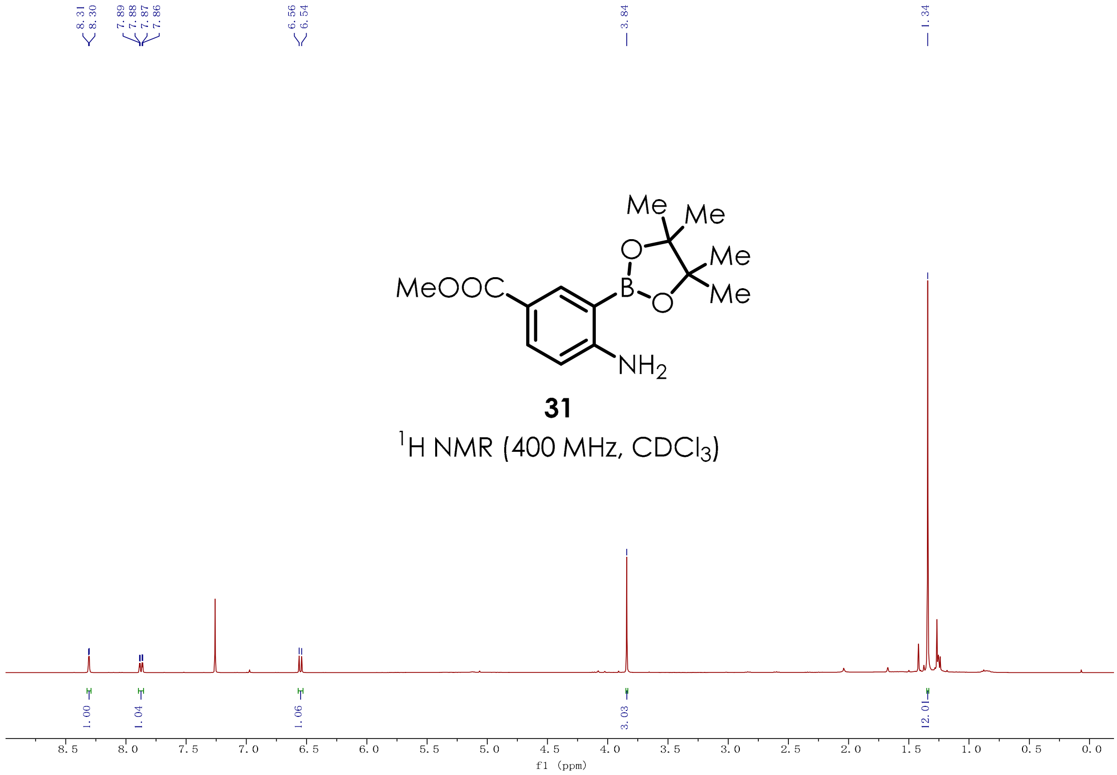
8.31
8.30
7.89
7.88
7.87
7.86
6.56
6.54
3.84
1.34
MeOOC
Me
Me
Me
Me
O
B
O
NH2
31
1H NMR (400 MHz, CDCl3)
1.00
1.04
1.06
3.03
12.01
f1 (ppm)

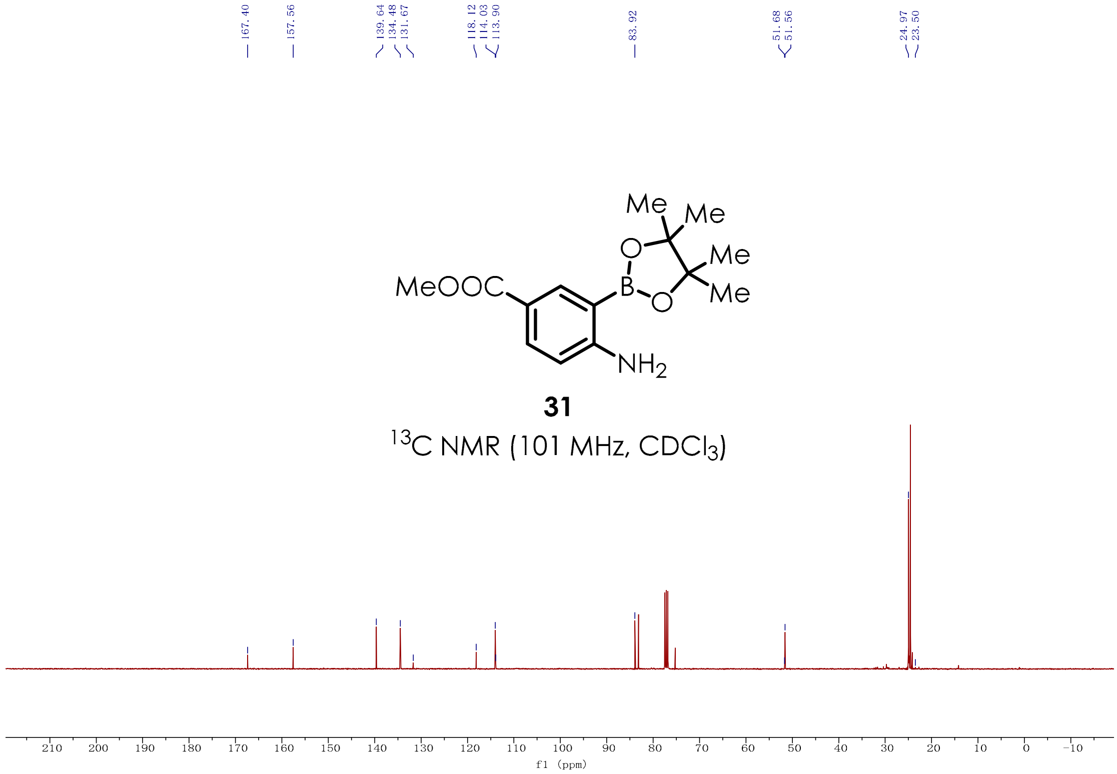
167.40
157.56
139.64
134.48
131.67
118.12
114.03
113.90
83.92
51.68
51.56
24.97
23.50
MeOOC
Me
Me
Me
Me
O
B
O
NH2
31
13C NMR (101 MHz, CDCl3)
f1 (ppm)

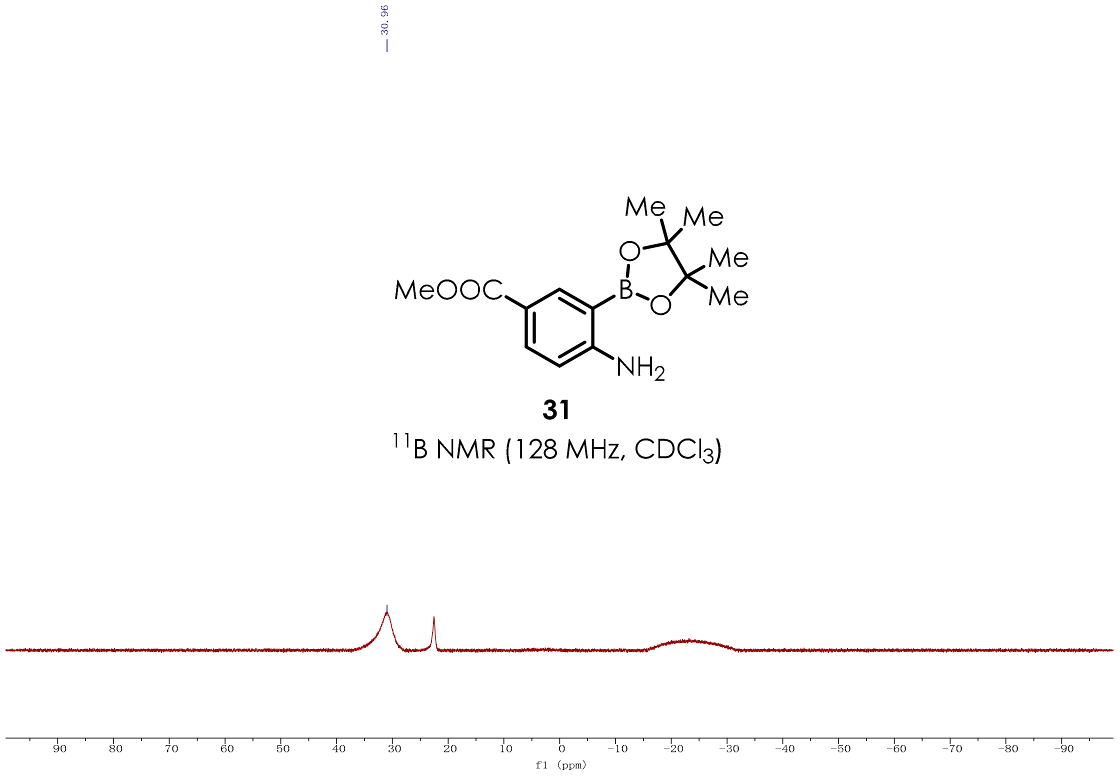
30.96
MeOOC
NH2
Me
O
B
31
^{11}B NMR (128 MHz, $CDCl_3$)
90 80 70 60 50 40 30 20 10 0 -10 -20 -30 -40 -50 -60 -70 -80 -90
f1 (ppm)

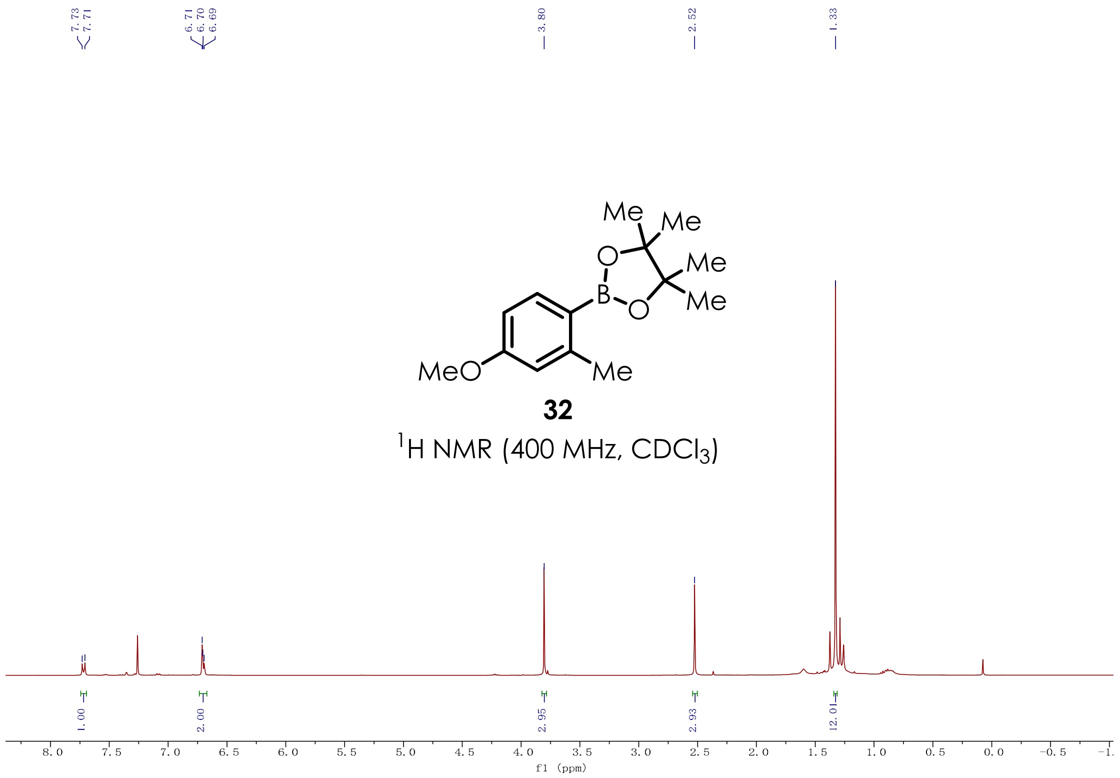
7.73
7.71
6.71
6.70
6.69
3.80
2.52
1.33
MeO
Me
O
B
32
^{1}H NMR (400 MHz, $CDCl_3$)
1.00
2.00
2.95
2.93
12.01
8.0 7.5 7.0 6.5 6.0 5.5 5.0 4.5 4.0 3.5 3.0 2.5 2.0 1.5 1.0 0.5 0.0 -0.5 -1.
f1 (ppm)

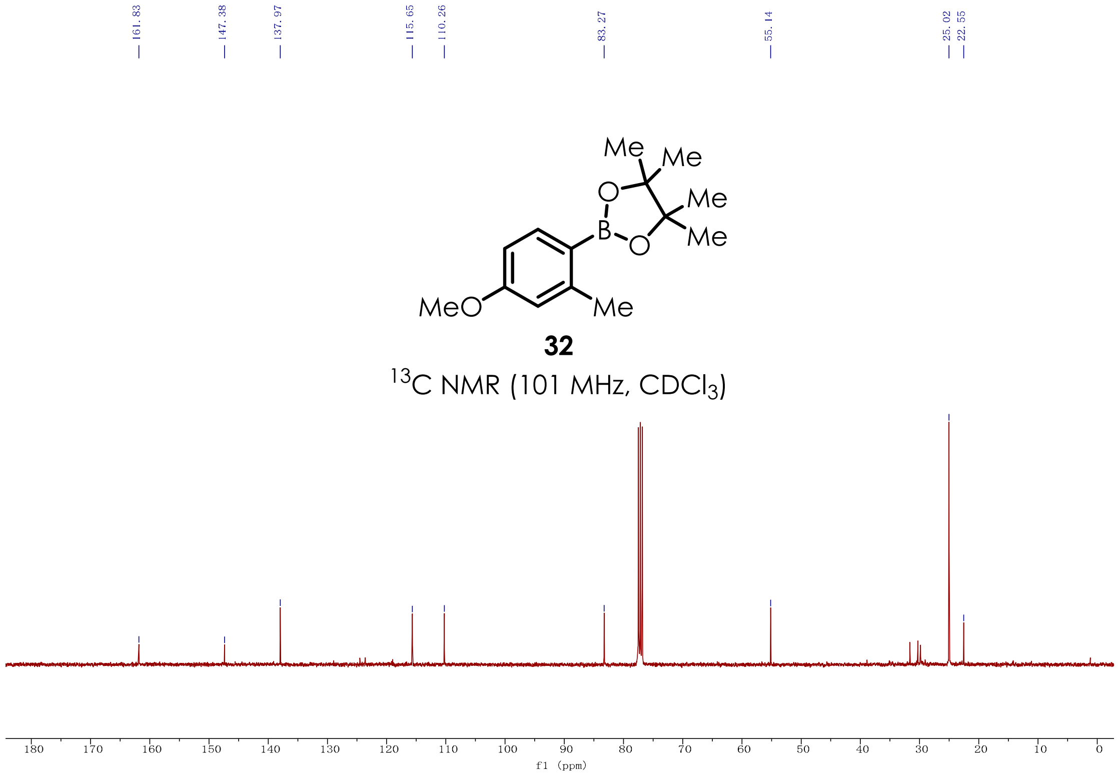
161.83
147.38
137.97
115.65
110.26
83.27
55.14
25.02
22.55
MeO
Me
Me
Me
Me
Me
O
B
O
32
^{13}C NMR (101 MHz, $CDCl_3$)
180 170 160 150 140 130 120 110 100 90 80 70 60 50 40 30 20 10 0
f1 (ppm)

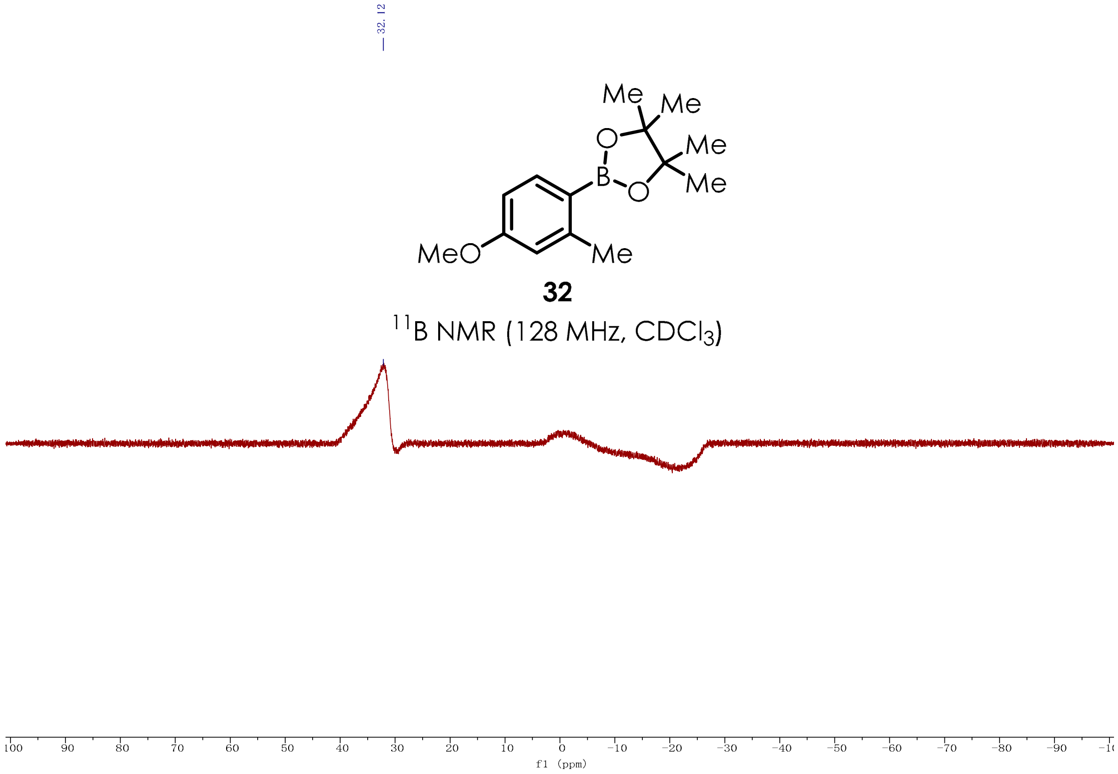
32.12
MeO
Me
Me
Me
Me
Me
O
B
O
32
^{11}B NMR (128 MHz, $CDCl_3$)
100 90 80 70 60 50 40 30 20 10 0 −10 −20 −30 −40 −50 −60 −70 −80 −90
f1 (ppm)

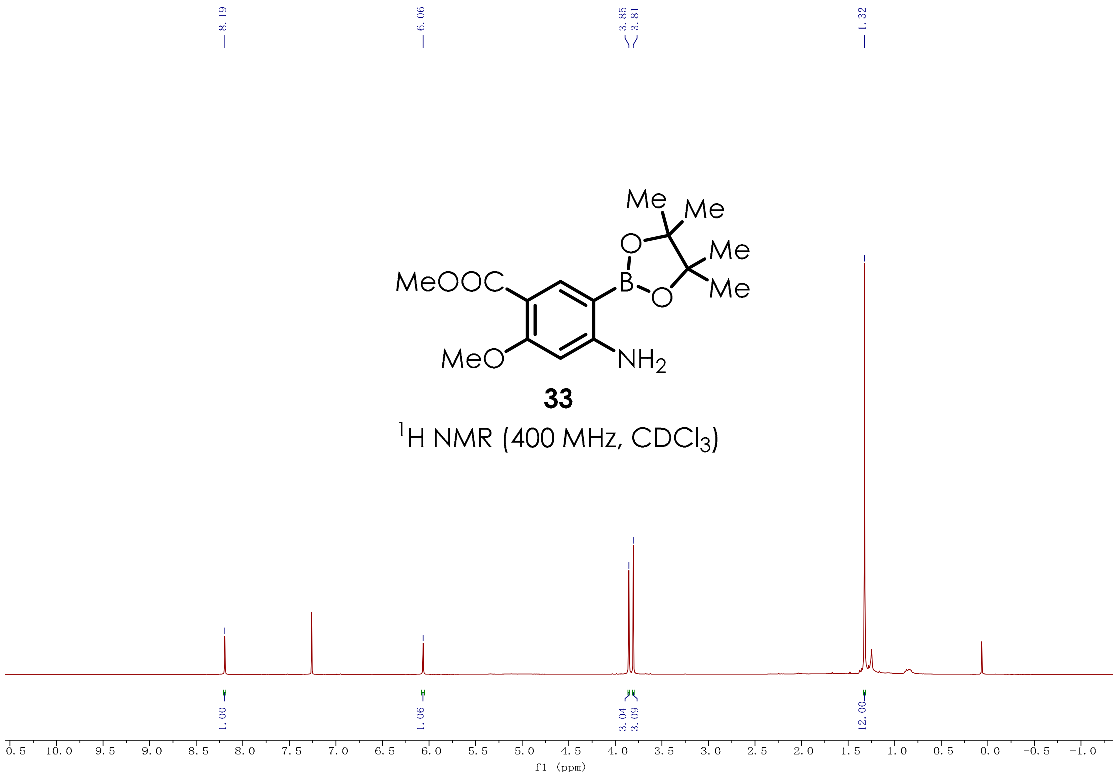
8.19
6.06
3.85
3.81
1.32
MeOOC
MeO
NH2
B
O
O
Me
Me
Me
Me
33
1H NMR (400 MHz, CDCl3)
1.00
1.06
3.04
3.09
12.00
f1 (ppm)

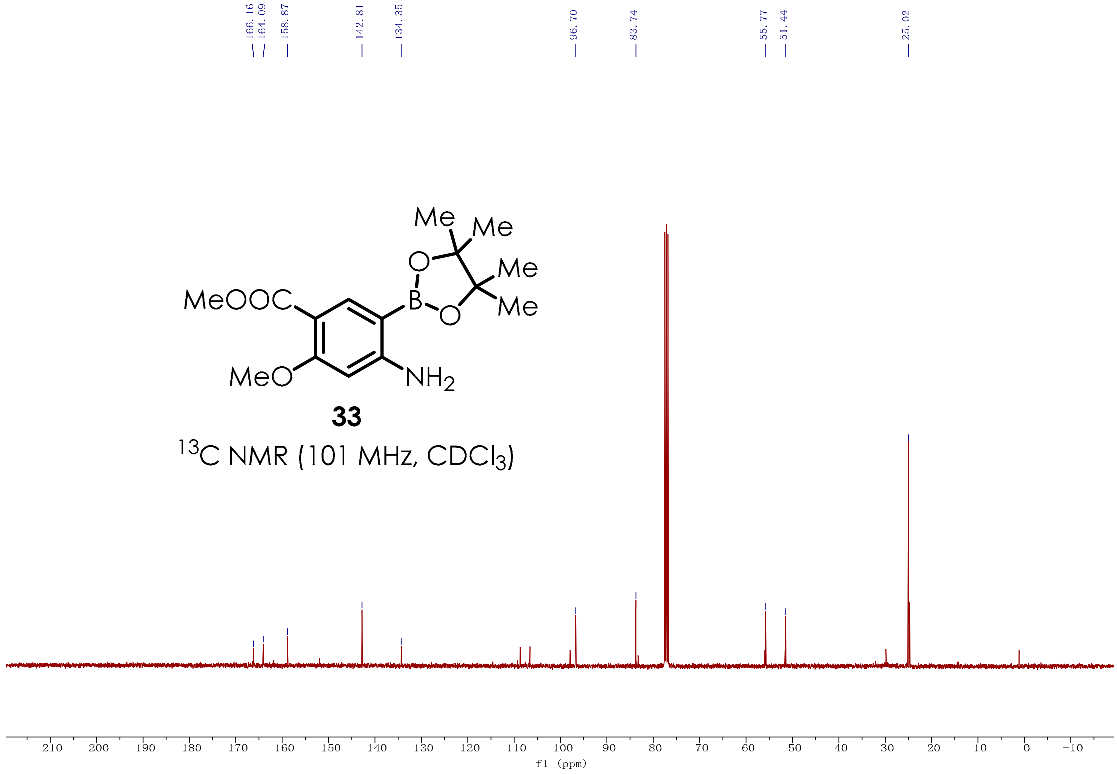
166.16
164.09
158.87
142.81
134.35
96.70
83.74
55.77
51.44
25.02
MeOOC
MeO
NH2
B
O
O
Me
Me
Me
Me
33
13C NMR (101 MHz, CDCl3)
f1 (ppm)

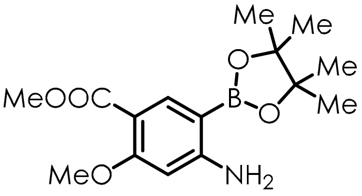

33

^{11}B NMR (128 MHz, $CDCl_3$)

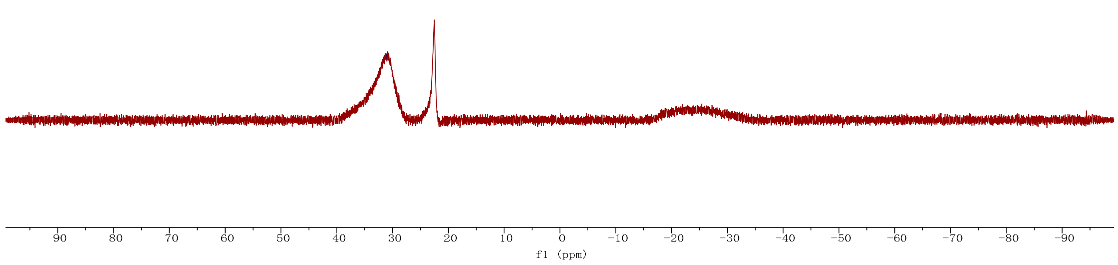

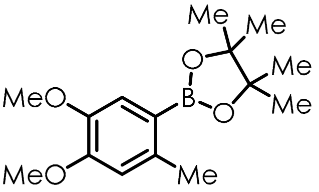

34

^{1}H NMR (400 MHz, $CDCl_3$)

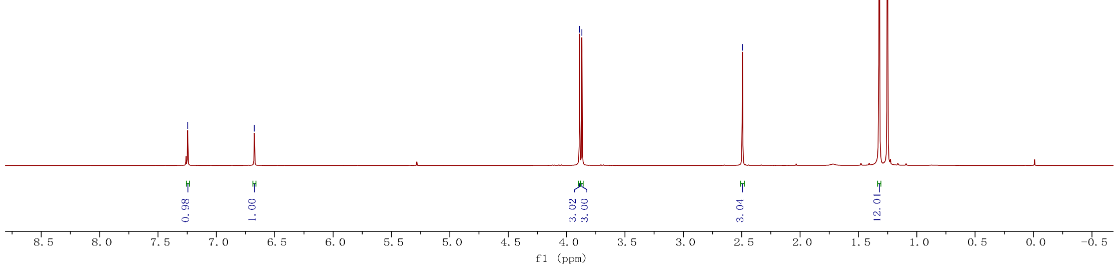

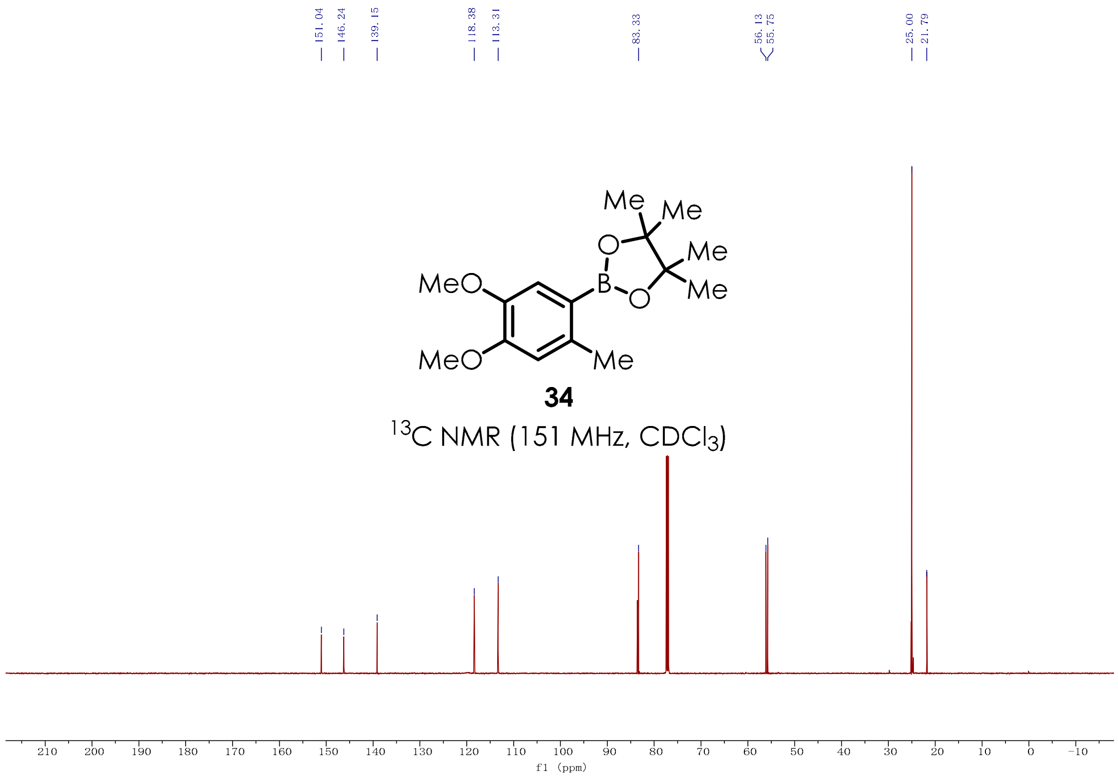
151.04
146.24
139.15
118.38
113.31
83.33
56.13
55.75
25.00
21.79
MeO
MeO
Me
Me
Me
Me
Me
B
O
O
34
^{13}C NMR (151 MHz, $CDCl_3$)
f1 (ppm)

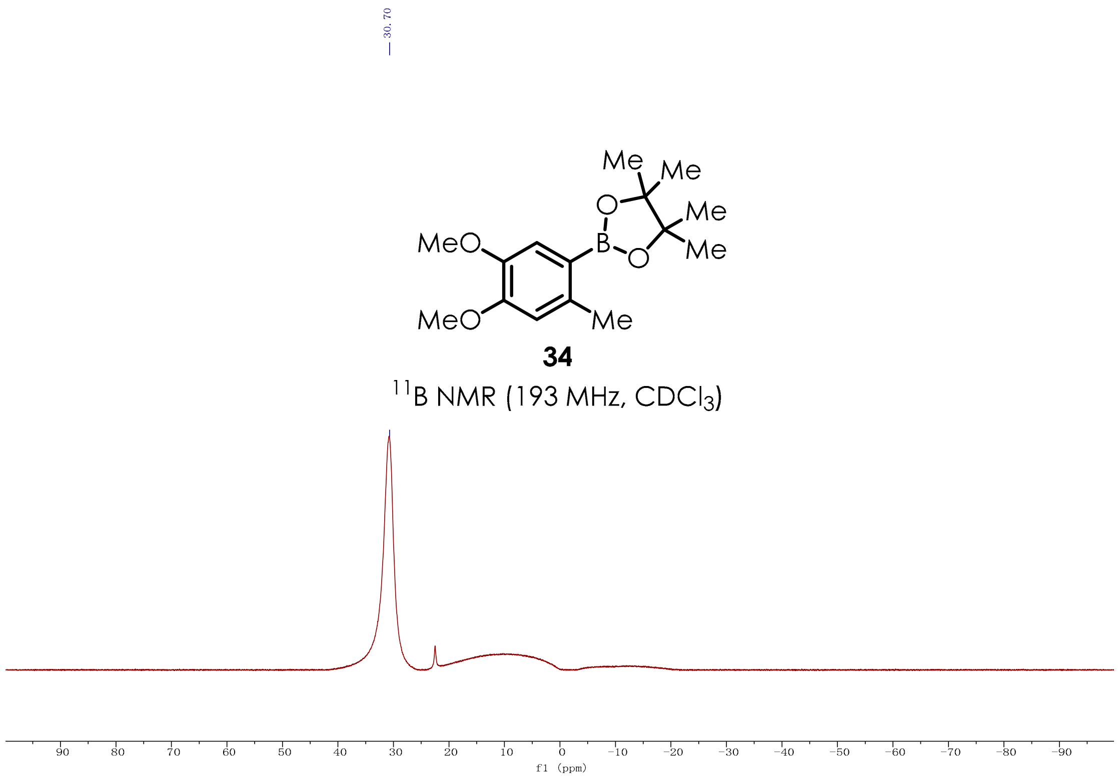
30.70
MeO
MeO
Me
Me
Me
Me
Me
B
O
O
34
^{11}B NMR (193 MHz, $CDCl_3$)
f1 (ppm)

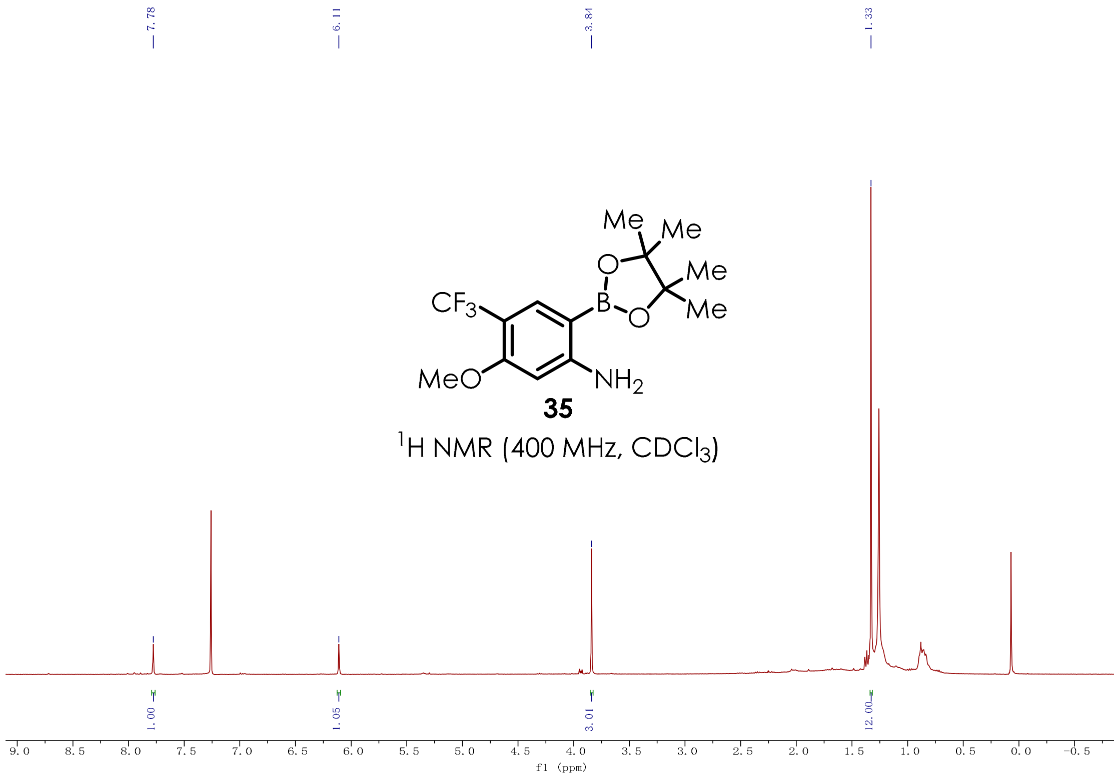
7.78
6.11
3.84
1.33
Me
Me
Me
Me
O
O
B
CF3
MeO
NH2
35
1H NMR (400 MHz, CDCl3)
1.00
1.05
3.01
12.00
9.0 8.5 8.0 7.5 7.0 6.5 6.0 5.5 5.0 4.5 4.0 3.5 3.0 2.5 2.0 1.5 1.0 0.5 0.0 -0.5
f1 (ppm)

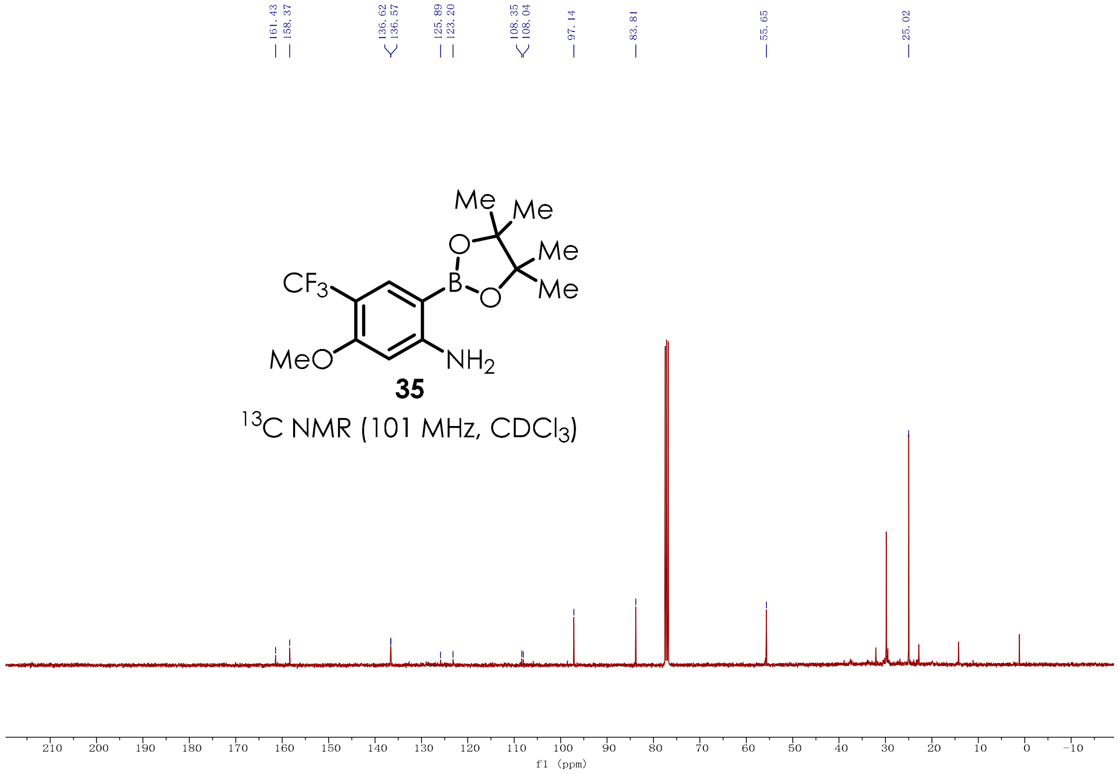
161.43
158.37
136.62
136.57
125.89
123.20
108.35
108.04
97.14
83.81
55.65
25.02
Me
Me
Me
Me
O
O
B
CF3
MeO
NH2
35
13C NMR (101 MHz, CDCl3)
210 200 190 180 170 160 150 140 130 120 110 100 90 80 70 60 50 40 30 20 10 0 -10
f1 (ppm)

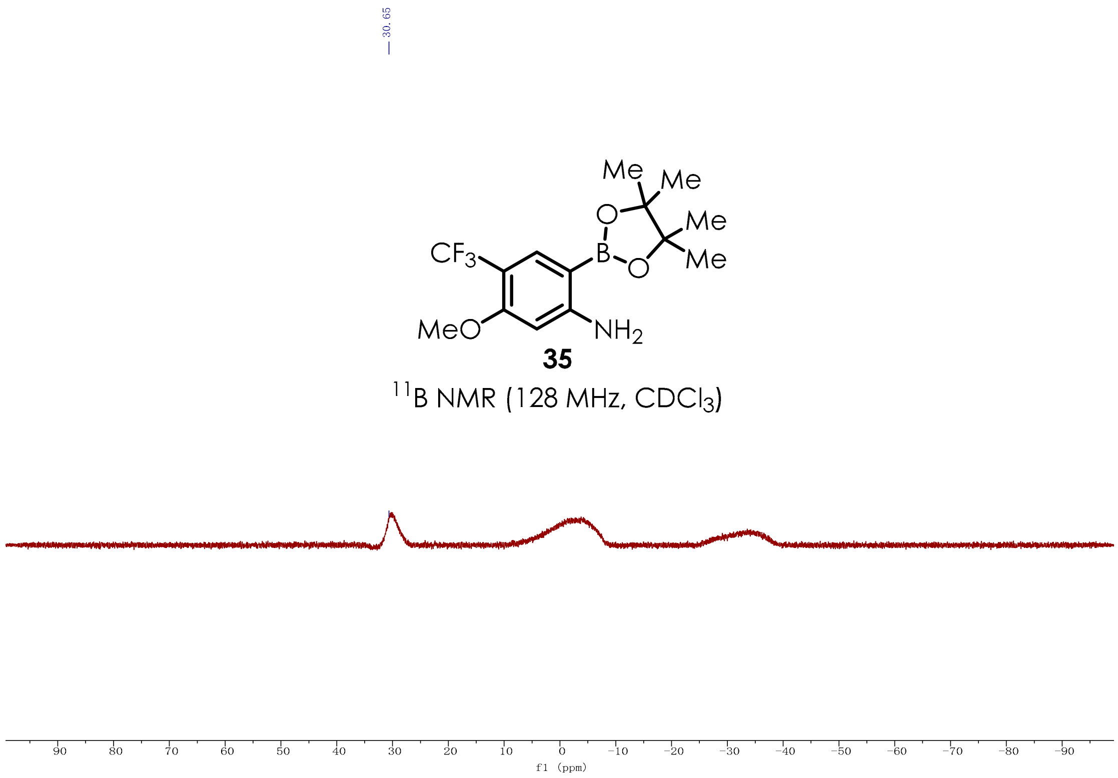
30.65
CF3
MeO
NH2
Me
Me
Me
Me
O
B
O
35
11B NMR (128 MHz, CDCl3)
90 80 70 60 50 40 30 20 10 0 −10 −20 −30 −40 −50 −60 −70 −80 −90
f1 (ppm)

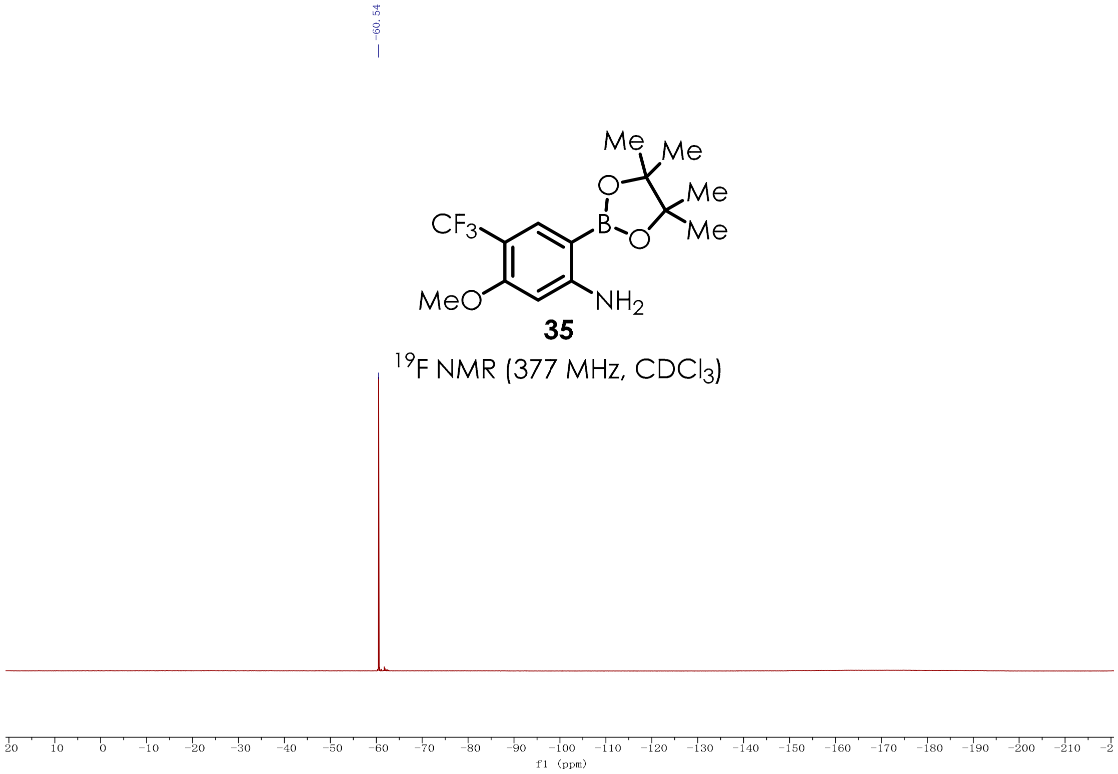
−60.54
CF3
MeO
NH2
Me
Me
Me
Me
O
B
O
35
19F NMR (377 MHz, CDCl3)
20 10 0 −10 −20 −30 −40 −50 −60 −70 −80 −90 −100 −110 −120 −130 −140 −150 −160 −170 −180 −190 −200 −210 −2
f1 (ppm)

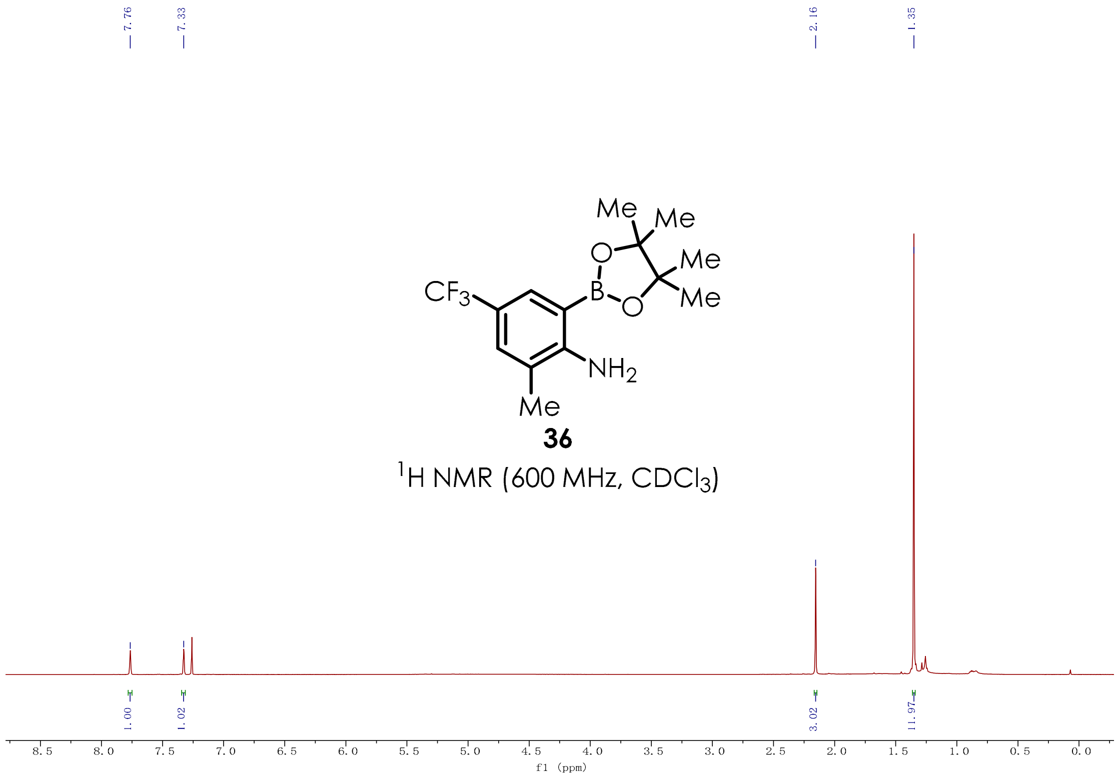
7.76
7.33
2.16
1.35
CF3
Me
Me
Me
Me
O
B
O
NH2
Me
36
1H NMR (600 MHz, CDCl3)
1.00
1.02
3.02
11.97
8.5 8.0 7.5 7.0 6.5 6.0 5.5 5.0 4.5 4.0 3.5 3.0 2.5 2.0 1.5 1.0 0.5 0.0
f1 (ppm)

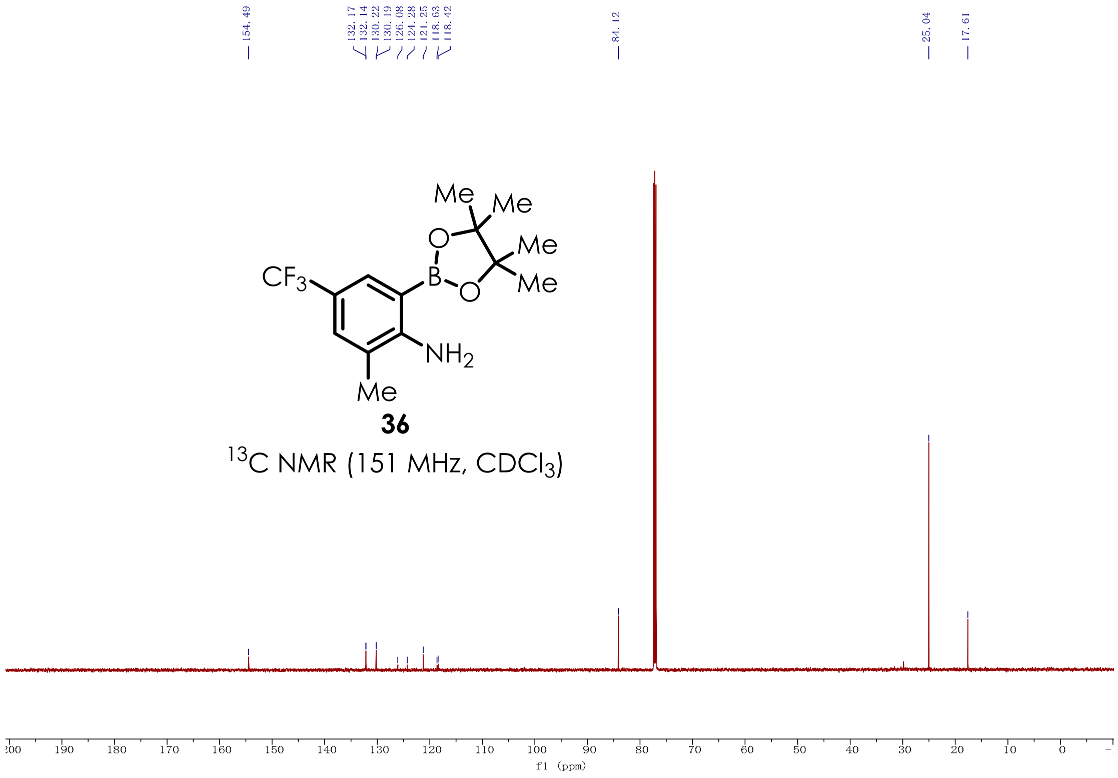
154.49
132.17
132.14
130.22
130.19
126.08
124.28
121.25
118.63
118.42
84.12
25.04
17.61
CF3
Me
Me
Me
Me
O
B
O
NH2
Me
36
13C NMR (151 MHz, CDCl3)
190 180 170 160 150 140 130 120 110 100 90 80 70 60 50 40 30 20 10 0
f1 (ppm)

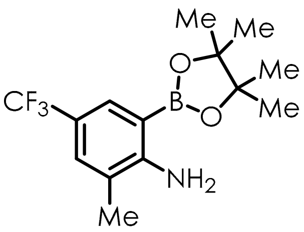

36

^{11}B NMR (128 MHz, $CDCl_3$)

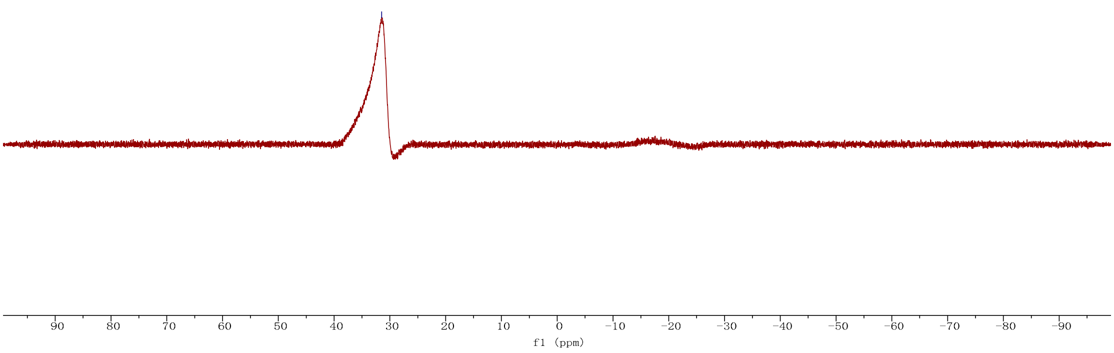

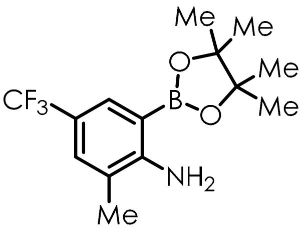

36

^{19}F NMR (377 MHz, $CDCl_3$)

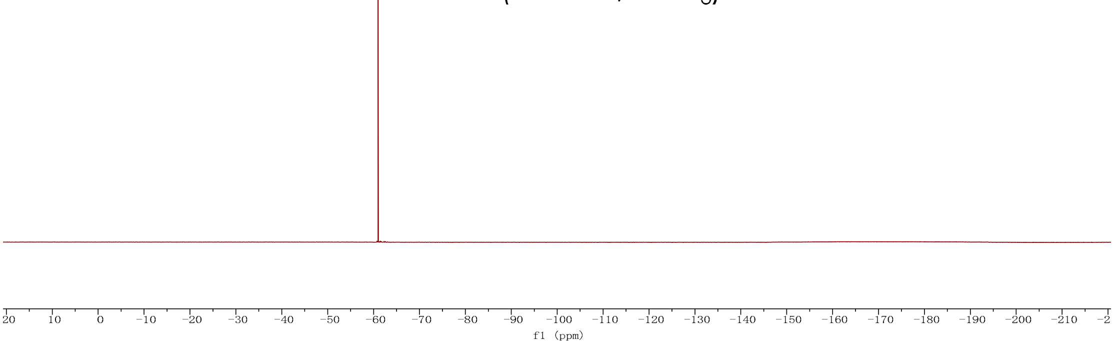

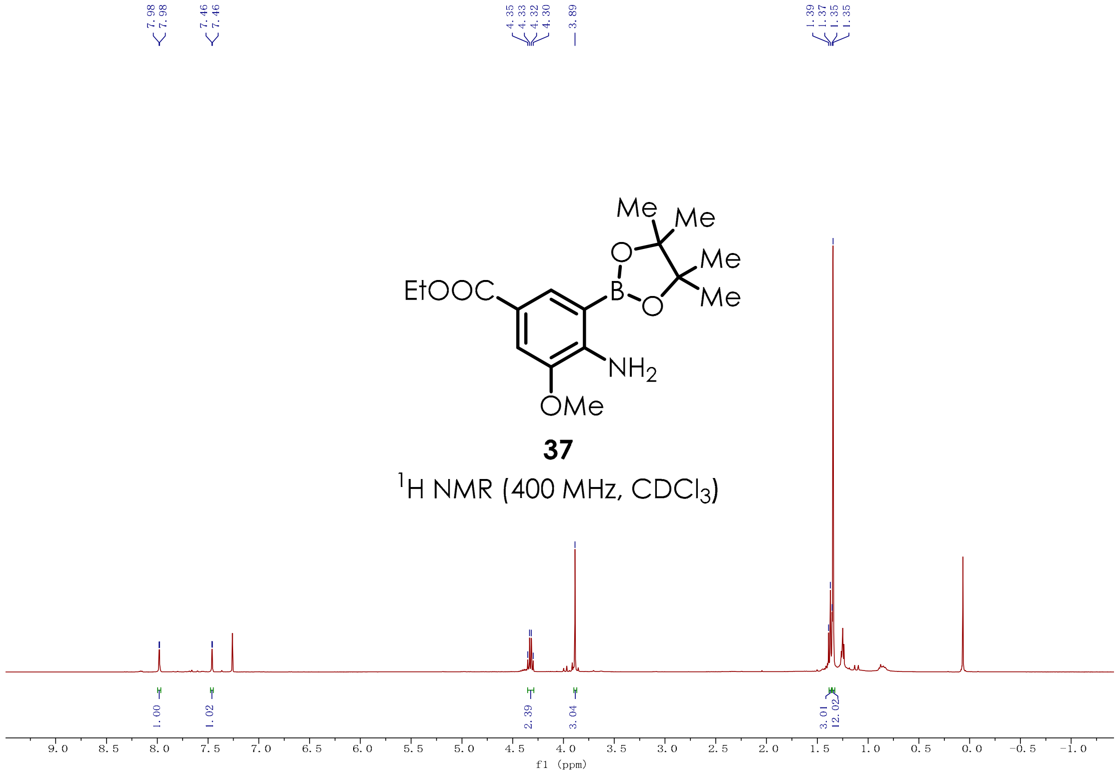
7.98
7.98
7.46
7.46
4.35
4.33
4.32
4.30
3.89
1.39
1.37
1.35
1.35
Me
Me
O
Me
EtOOC
B
O
Me
NH2
OMe
37
1H NMR (400 MHz, CDCl3)
1.00
1.02
2.39
3.04
3.01
12.02
9.0
8.5
8.0
7.5
7.0
6.5
6.0
5.5
5.0
4.5
4.0
3.5
3.0
2.5
2.0
1.5
1.0
0.5
0.0
-0.5
-1.0
f1 (ppm)

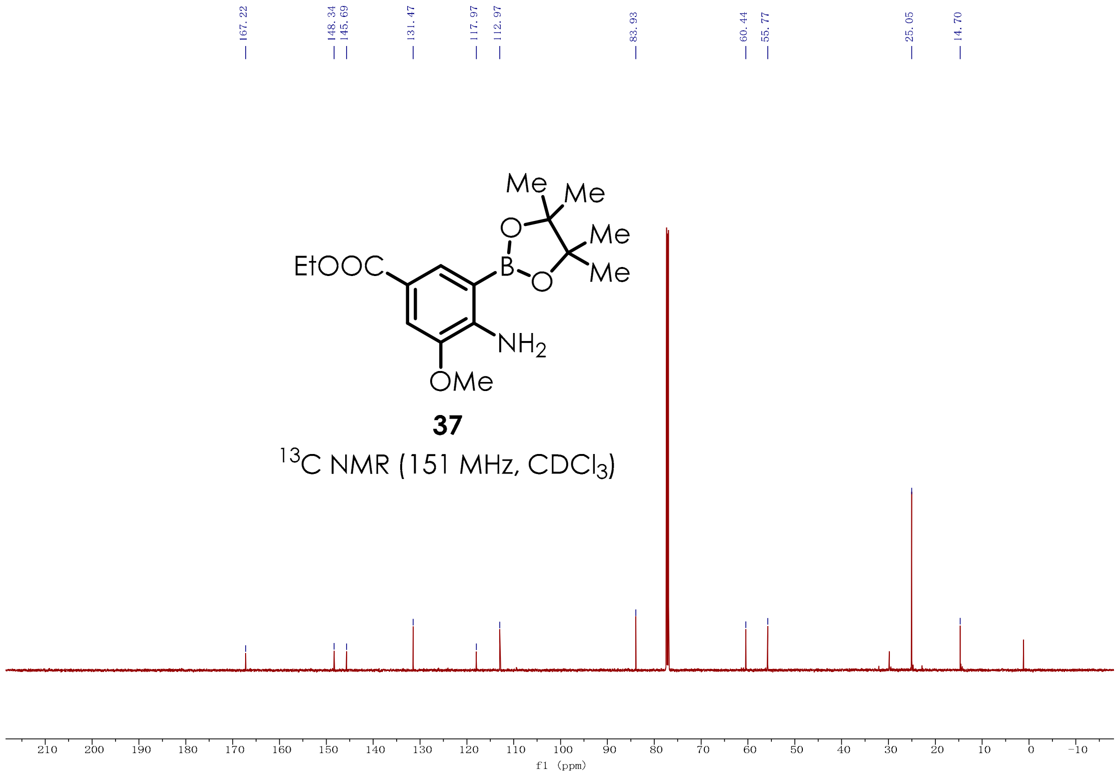
167.22
148.34
145.69
131.47
117.97
112.97
83.93
60.44
55.77
25.05
14.70
Me
Me
O
Me
EtOOC
B
O
Me
NH2
OMe
37
13C NMR (151 MHz, CDCl3)
210
200
190
180
170
160
150
140
130
120
110
100
90
80
70
60
50
40
30
20
10
0
-10
f1 (ppm)

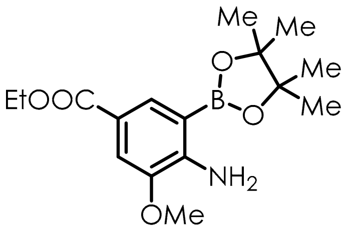

37

^{11}B NMR (193 MHz, CDCl$_3$)

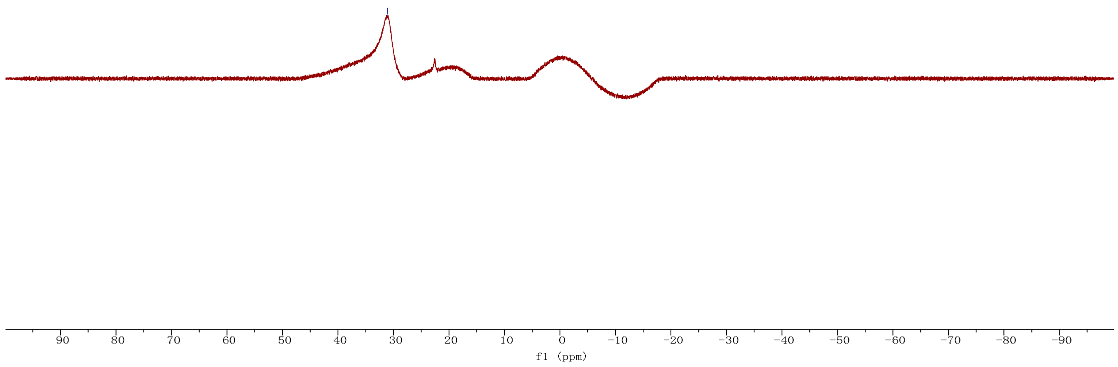

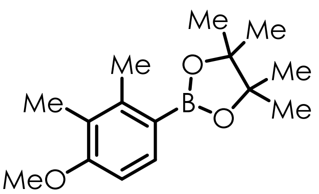

38

^{1}H NMR (400 MHz, CDCl$_3$)

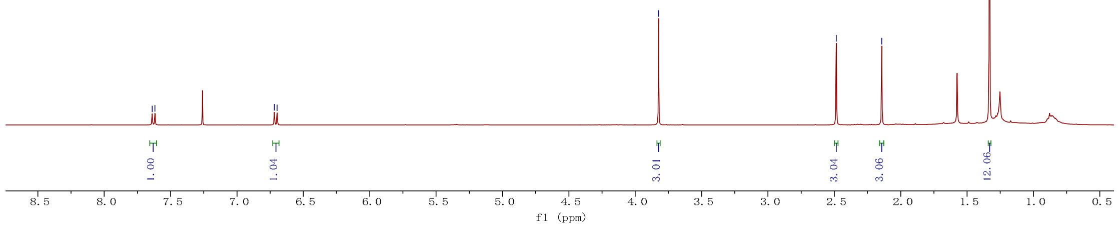

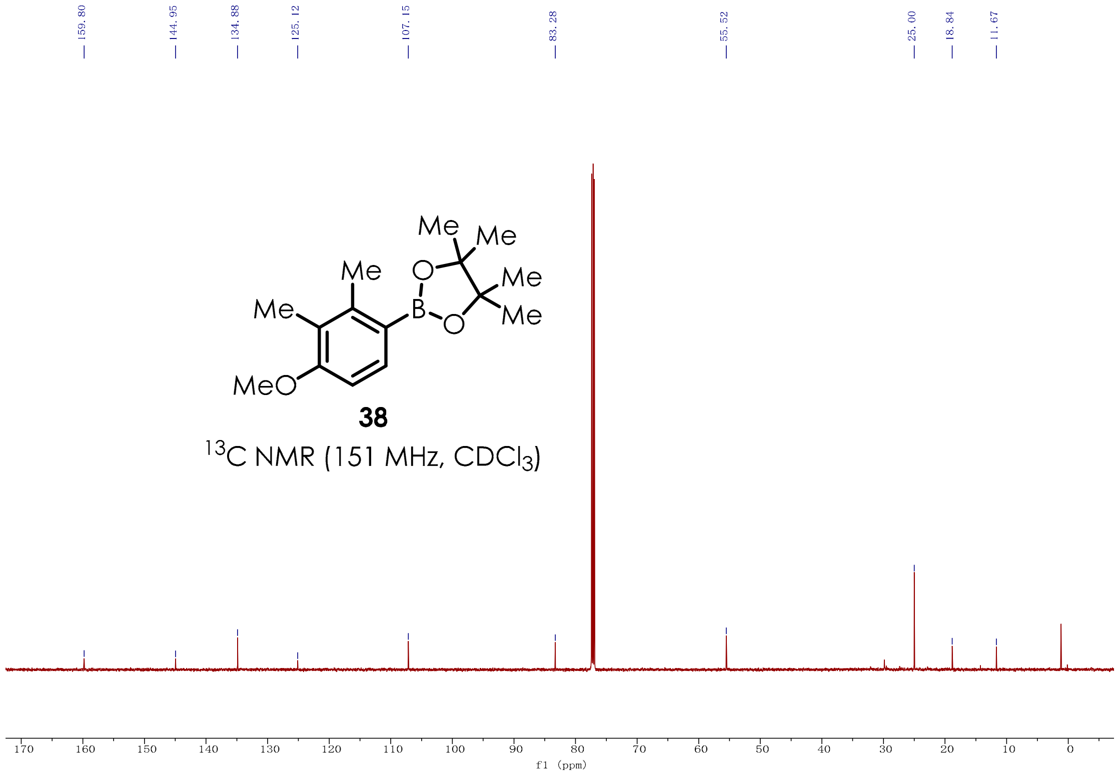
159.80
144.95
134.88
125.12
107.15
83.28
55.52
25.00
18.84
11.67
Me
Me
Me
Me
Me
Me
MeO
O
O
B
38
13C NMR (151 MHz, CDCl3)
f1 (ppm)

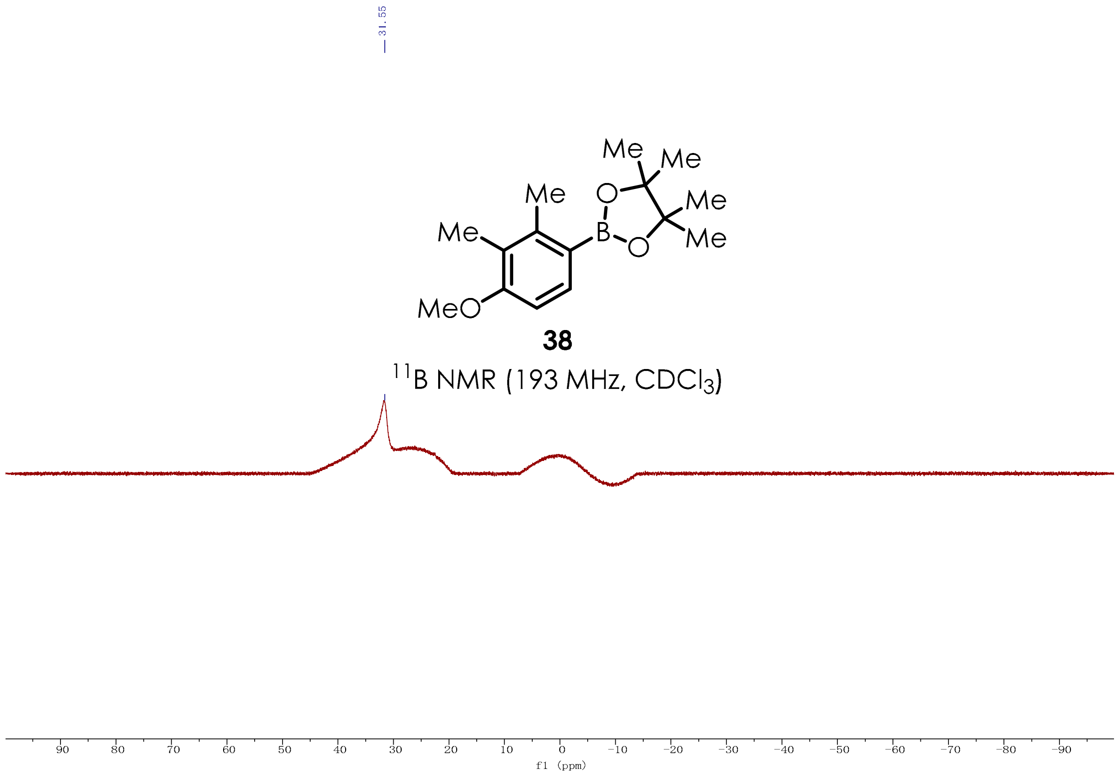
31.55
Me
Me
Me
Me
Me
Me
MeO
O
O
B
38
11B NMR (193 MHz, CDCl3)
f1 (ppm)

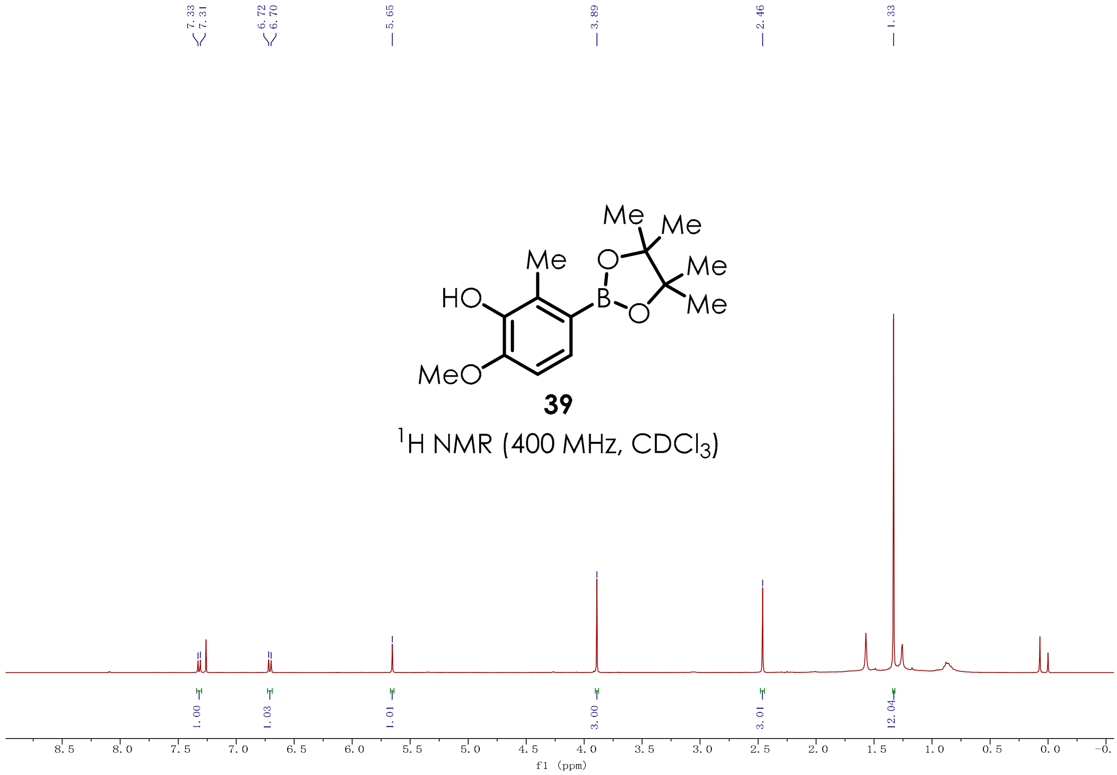

7.33
7.31
6.72
6.70
5.65
3.89
2.46
1.33
Me
Me
Me
Me
Me
HO
MeO
O
B
O
39
^{1}H NMR (400 MHz, $CDCl_3$)
1.00
1.03
1.01
3.00
3.01
12.04
8.5
8.0
7.5
7.0
6.5
6.0
5.5
5.0
4.5
4.0
3.5
3.0
2.5
2.0
1.5
1.0
0.5
0.0
f1 (ppm)

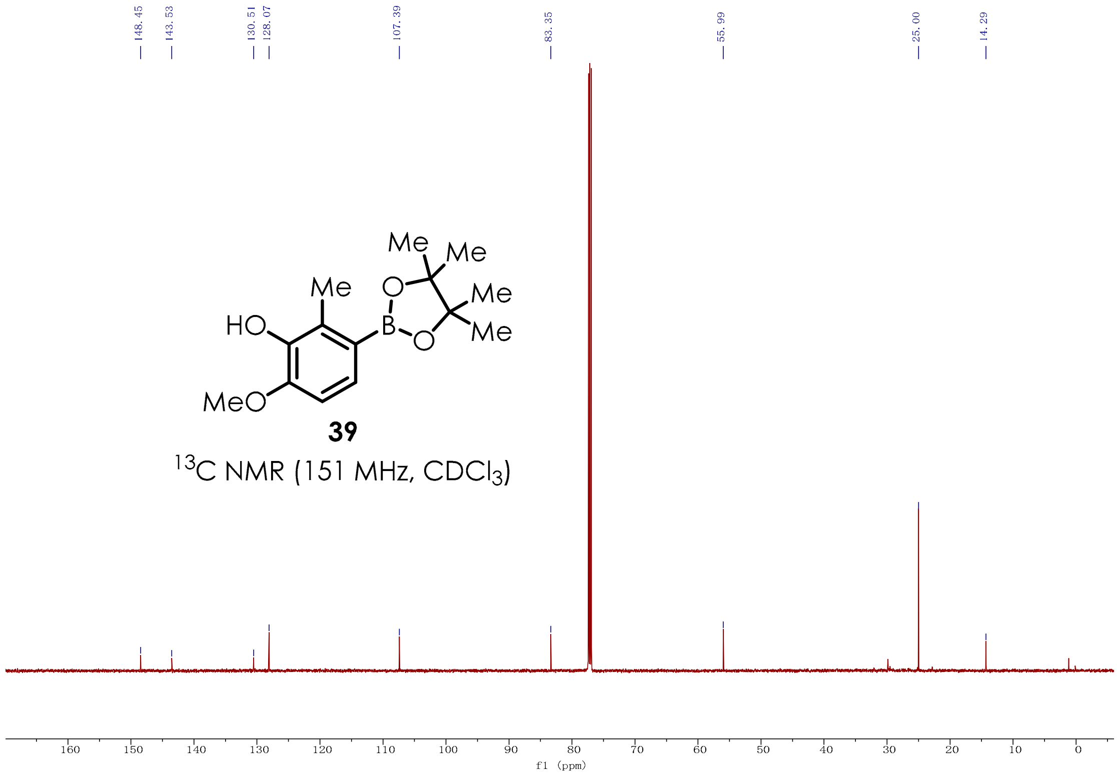

148.45
143.53
130.51
128.07
107.39
83.35
55.99
25.00
14.29
Me
Me
Me
Me
Me
HO
MeO
O
B
O
39
^{13}C NMR (151 MHz, $CDCl_3$)
160
150
140
130
120
110
100
90
80
70
60
50
40
30
20
10
0
f1 (ppm)

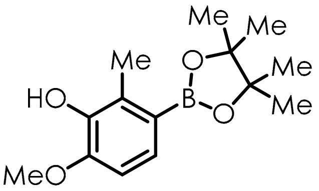

39

^{11}B NMR (193 MHz, CDCl$_3$)

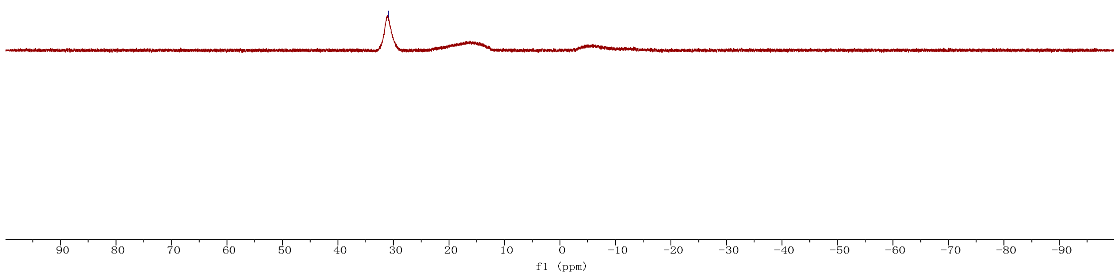

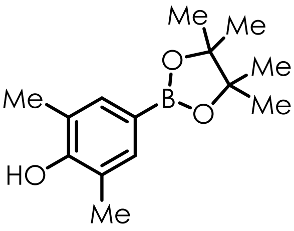

40

^{1}H NMR (400 MHz, CDCl$_3$)

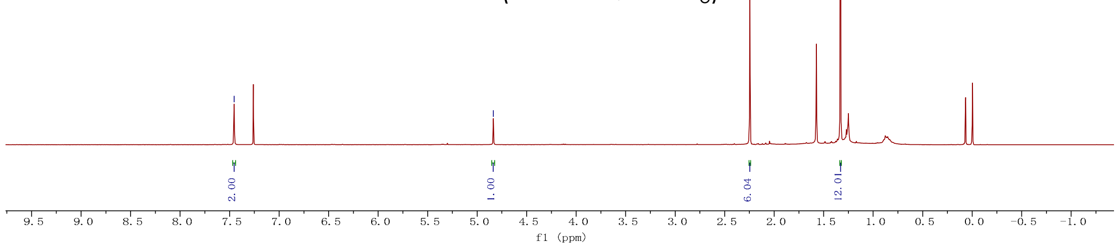

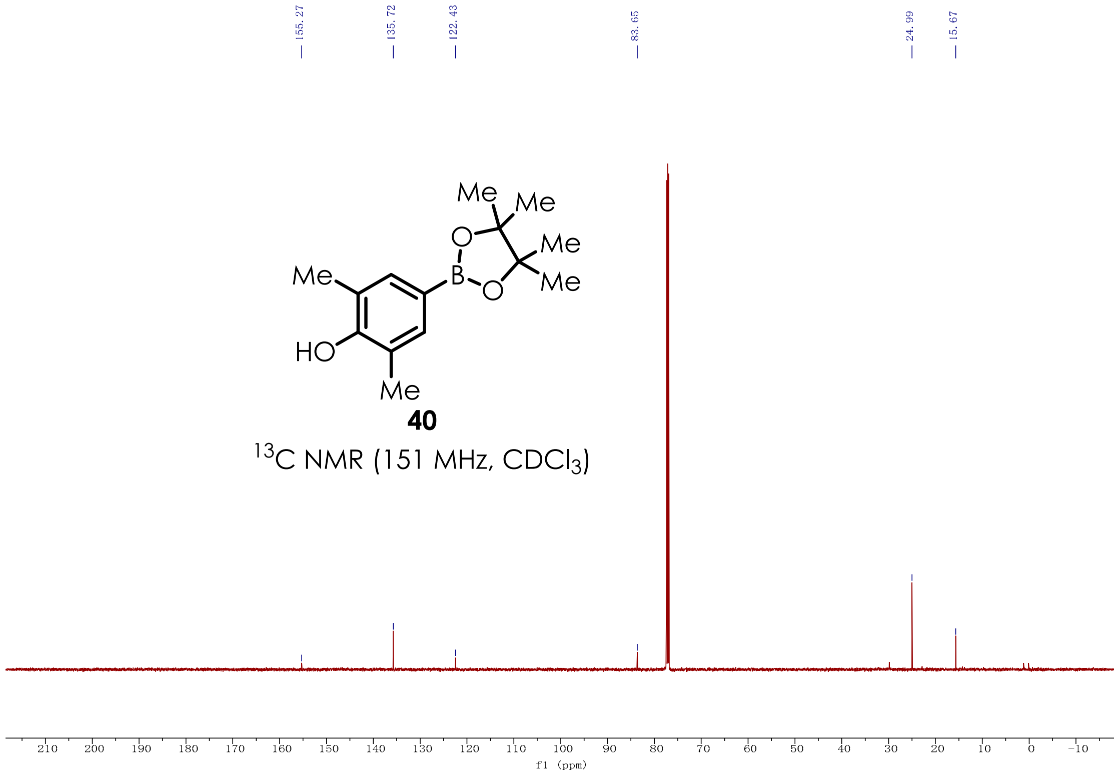
155.27
135.72
122.43
83.65
24.99
15.67
Me
Me
Me
Me
Me
HO
Me
40
13C NMR (151 MHz, CDCl3)
f1 (ppm)

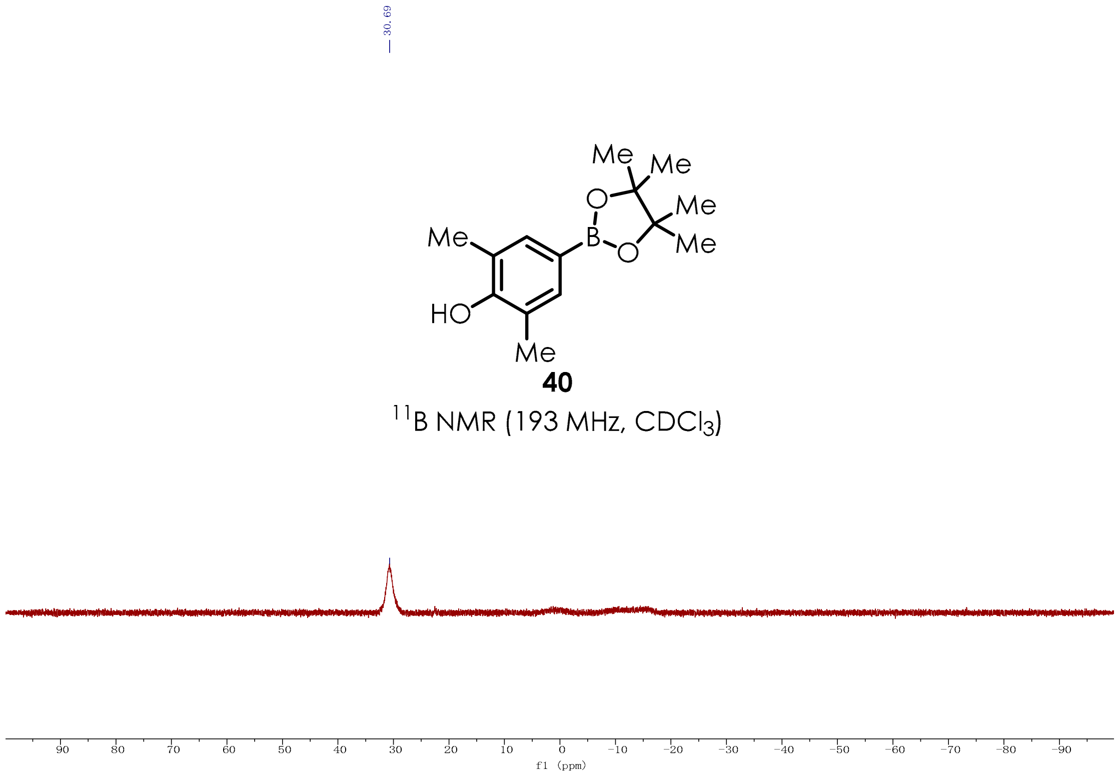
30.69
Me
Me
Me
Me
Me
HO
Me
40
11B NMR (193 MHz, CDCl3)
f1 (ppm)

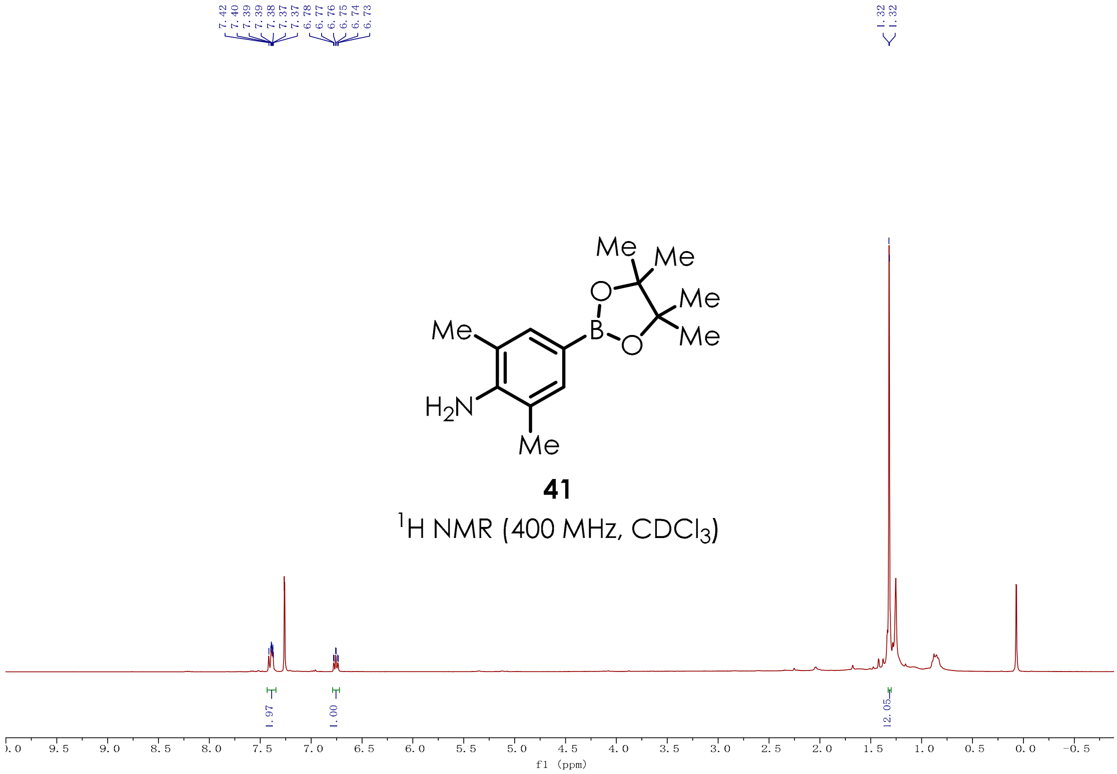
7.42
7.40
7.39
7.38
7.37
7.37
6.78
6.77
6.76
6.75
6.74
6.73
1.32
1.32
Me
Me
Me
Me
Me
Me
O
B
O
H2N
41
1H NMR (400 MHz, CDCl3)
1.97
1.00
12.05
f1 (ppm)

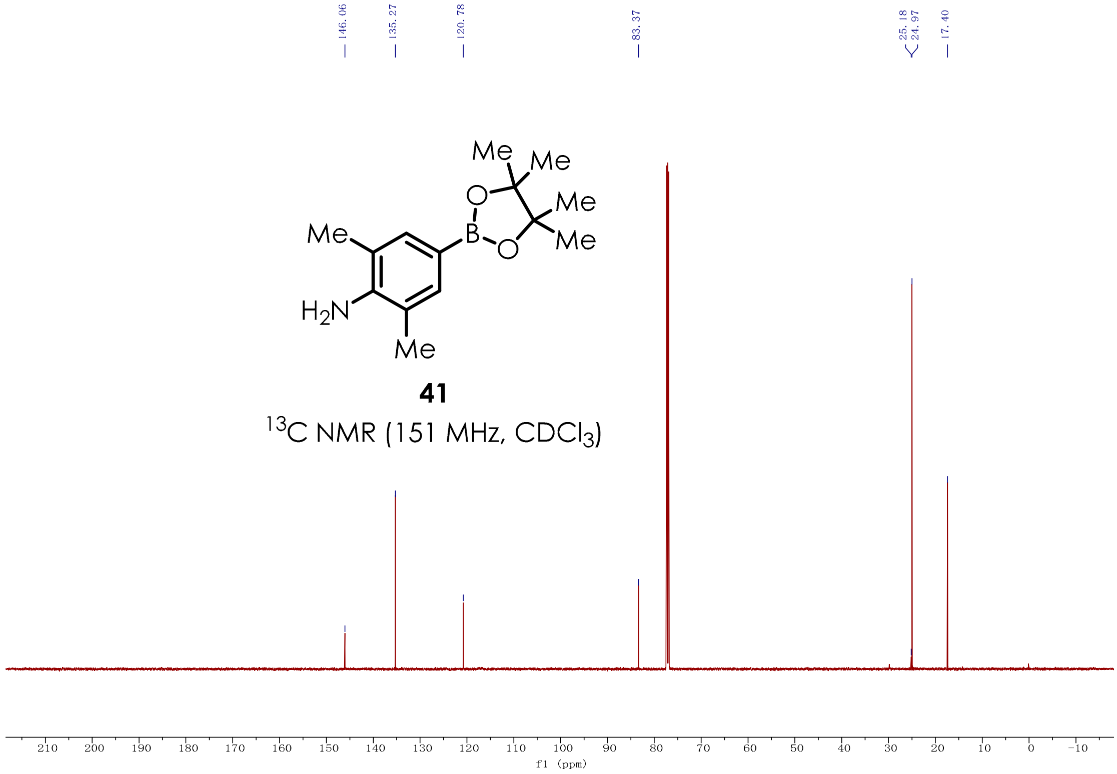
146.06
135.27
120.78
83.37
25.18
24.97
17.40
Me
Me
Me
Me
Me
Me
O
B
O
H2N
41
13C NMR (151 MHz, CDCl3)
f1 (ppm)

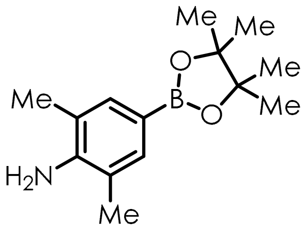

41

^{11}B NMR (193 MHz, $CDCl_3$)

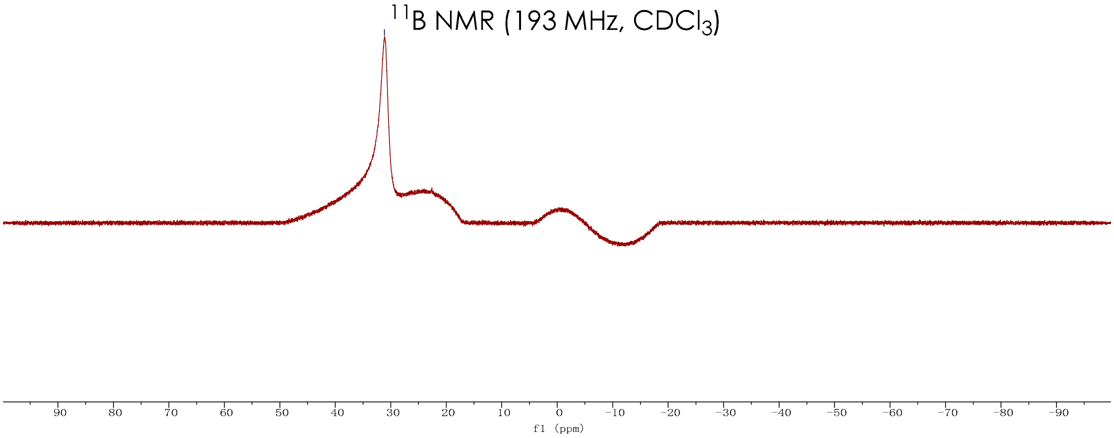

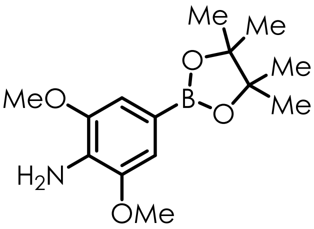

42

^{1}H NMR (400 MHz, $CDCl_3$)

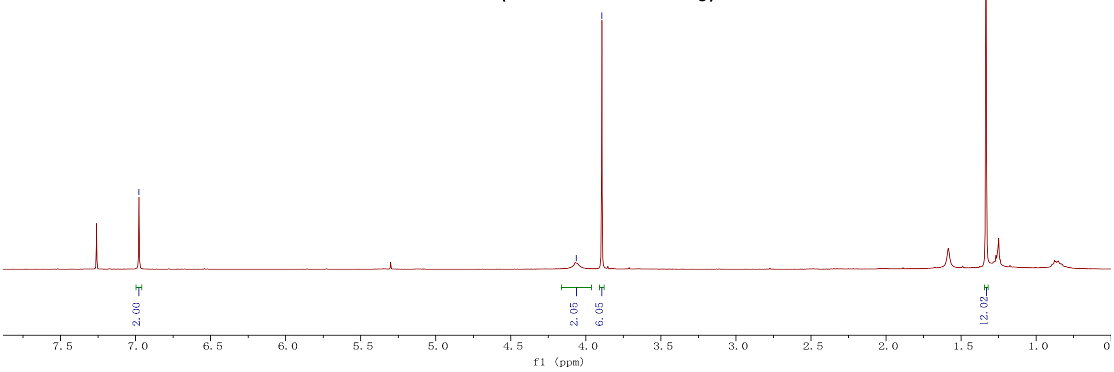

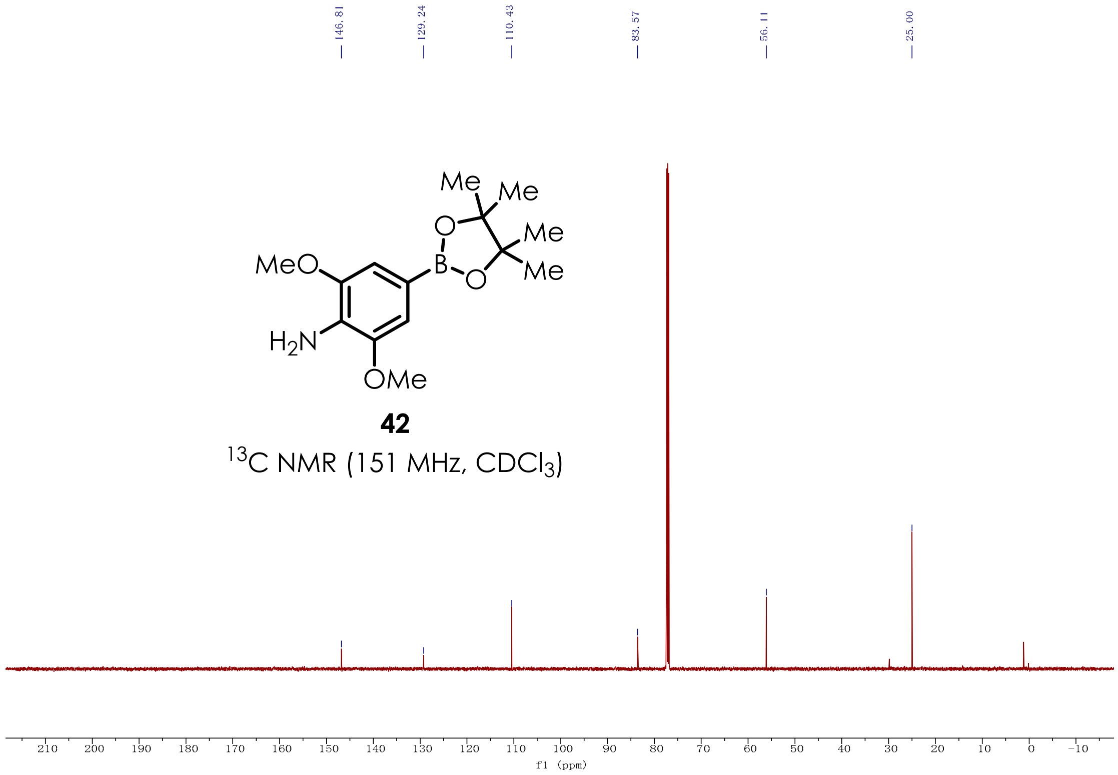
146.81
129.24
110.43
83.57
56.11
25.00
MeO
H2N
OMe
Me
Me
Me
Me
B
O
O
42
13C NMR (151 MHz, CDCl3)
210 200 190 180 170 160 150 140 130 120 110 100 90 80 70 60 50 40 30 20 10 0 -10
f1 (ppm)

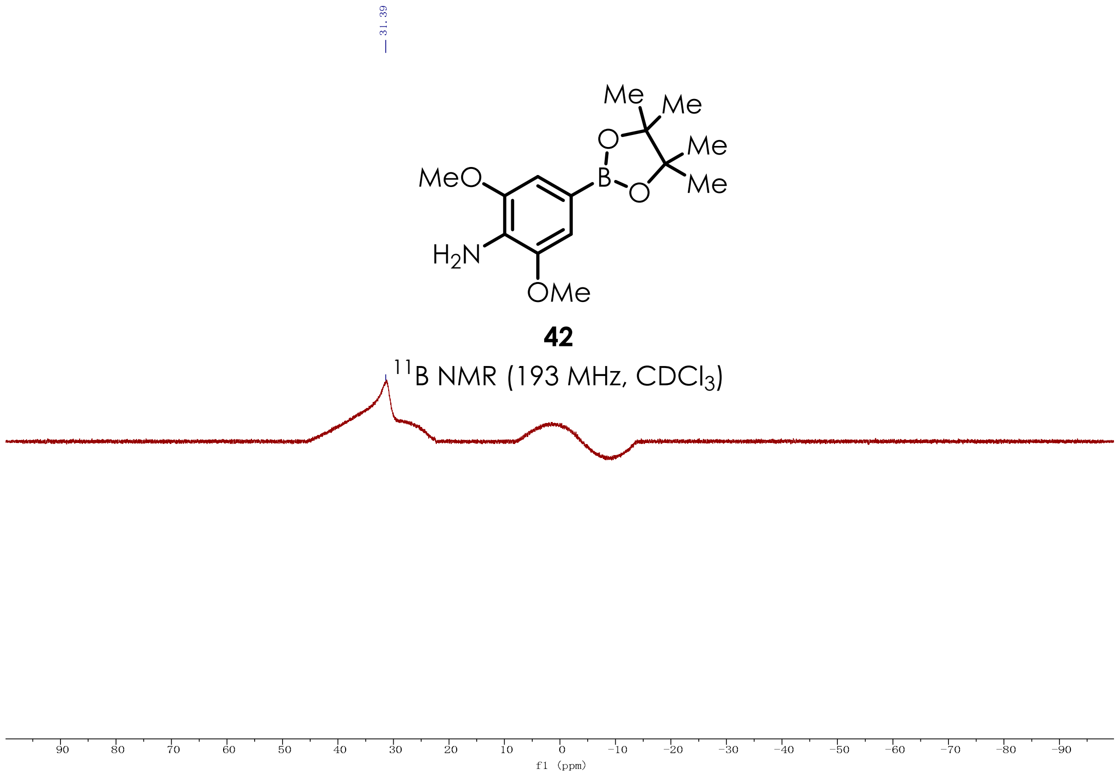
31.39
MeO
H2N
OMe
Me
Me
Me
Me
B
O
O
42
11B NMR (193 MHz, CDCl3)
90 80 70 60 50 40 30 20 10 0 -10 -20 -30 -40 -50 -60 -70 -80 -90
f1 (ppm)

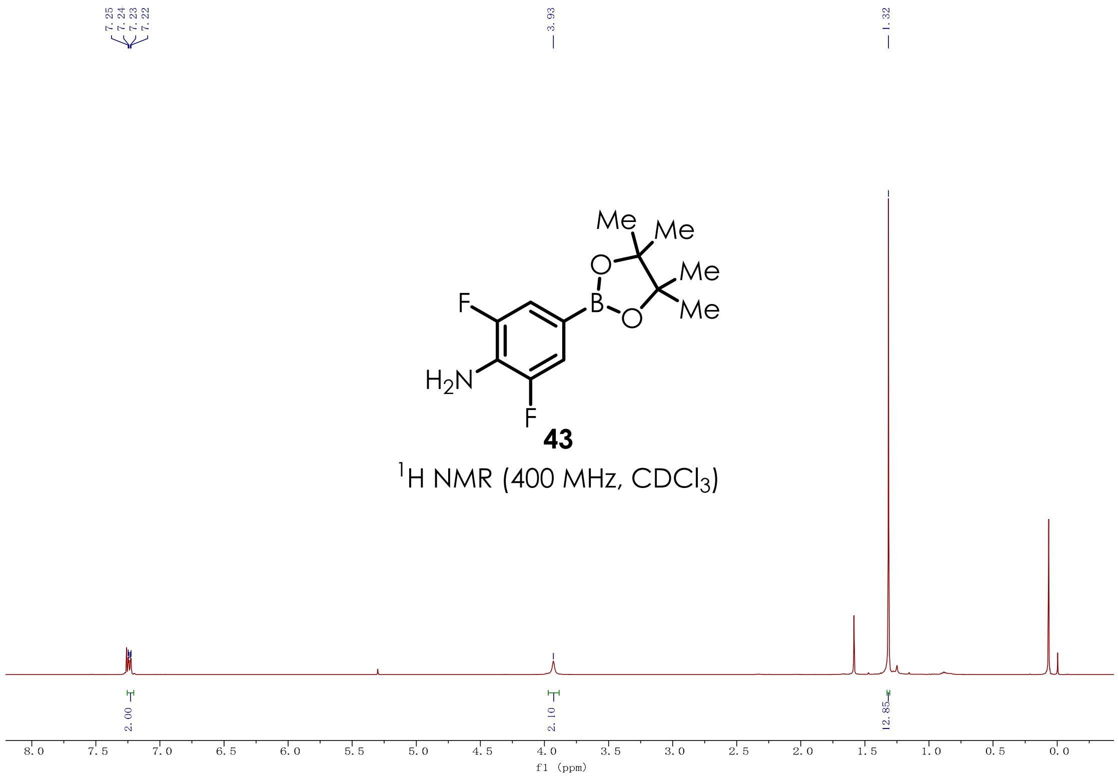
7.25
7.24
7.23
7.22
3.93
1.32
Me
Me
Me
Me
O
O
B
F
F
H2N
43
1H NMR (400 MHz, CDCl3)
2.00
2.10
12.85
8.0
7.5
7.0
6.5
6.0
5.5
5.0
4.5
4.0
3.5
3.0
2.5
2.0
1.5
1.0
0.5
0.0
f1 (ppm)

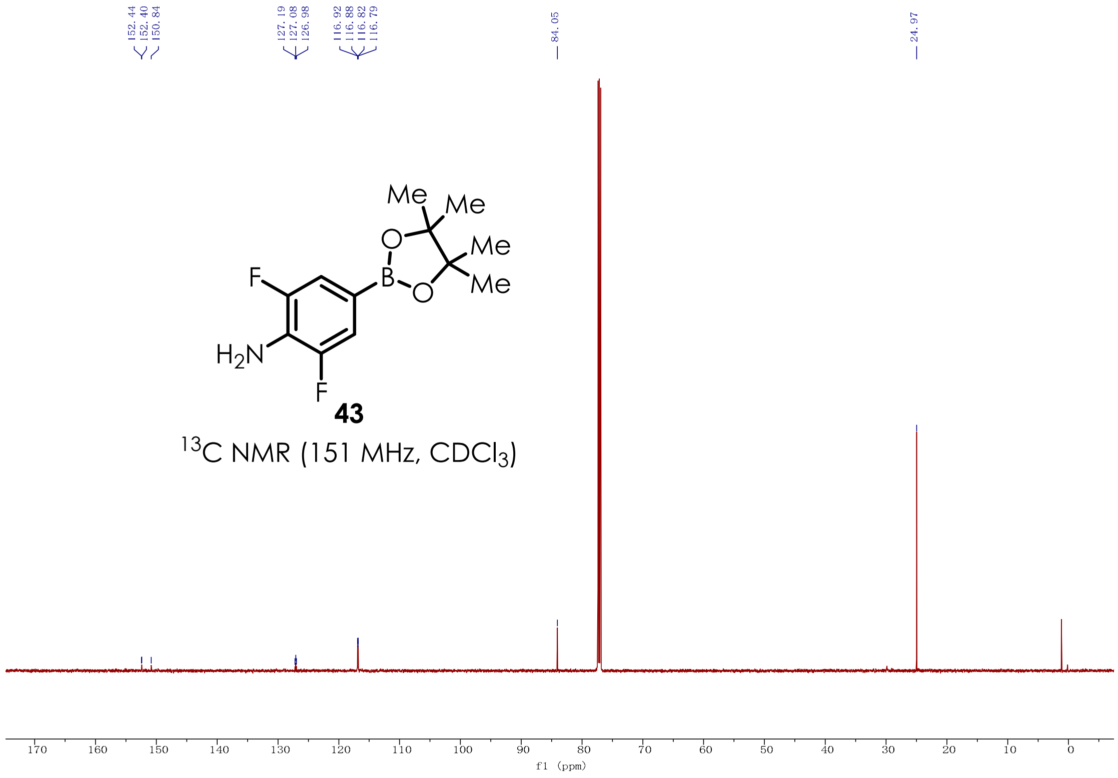
152.44
152.40
150.84
127.19
127.08
126.98
116.92
116.88
116.82
116.79
84.05
24.97
Me
Me
Me
Me
O
O
B
F
F
H2N
43
13C NMR (151 MHz, CDCl3)
170
160
150
140
130
120
110
100
90
80
70
60
50
40
30
20
10
0
f1 (ppm)

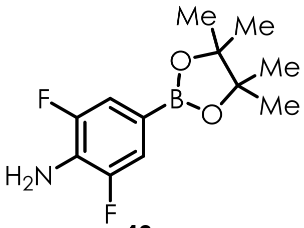

43

^{11}B NMR (193 MHz, $CDCl_3$)

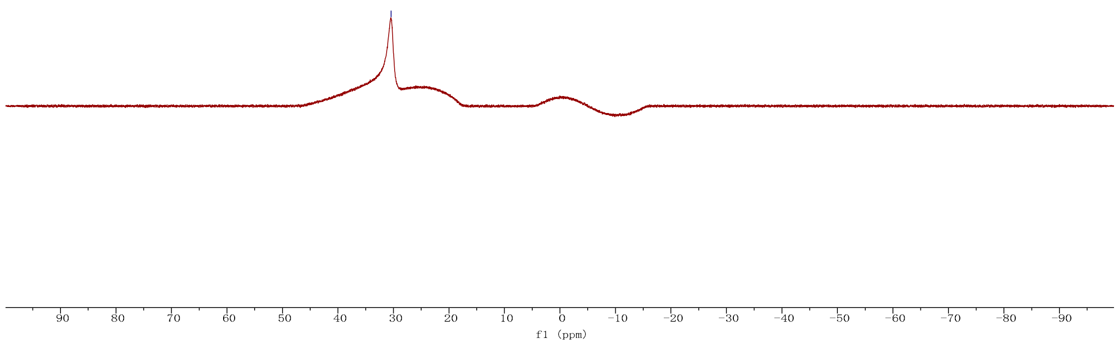

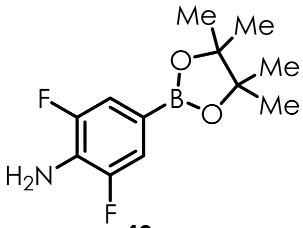

43

^{19}F NMR (377 MHz, $CDCl_3$)

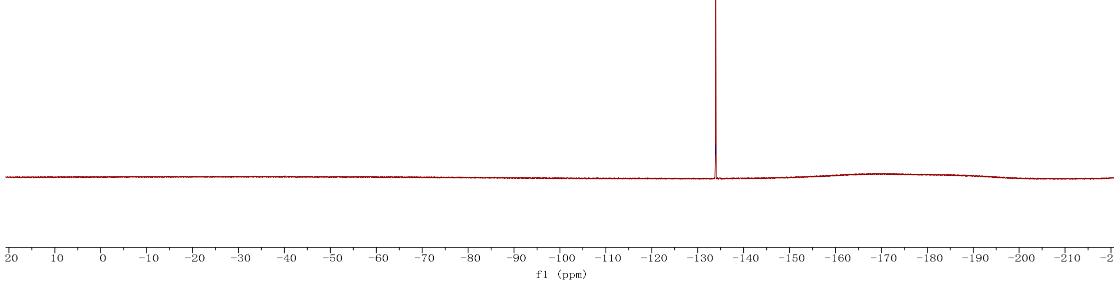

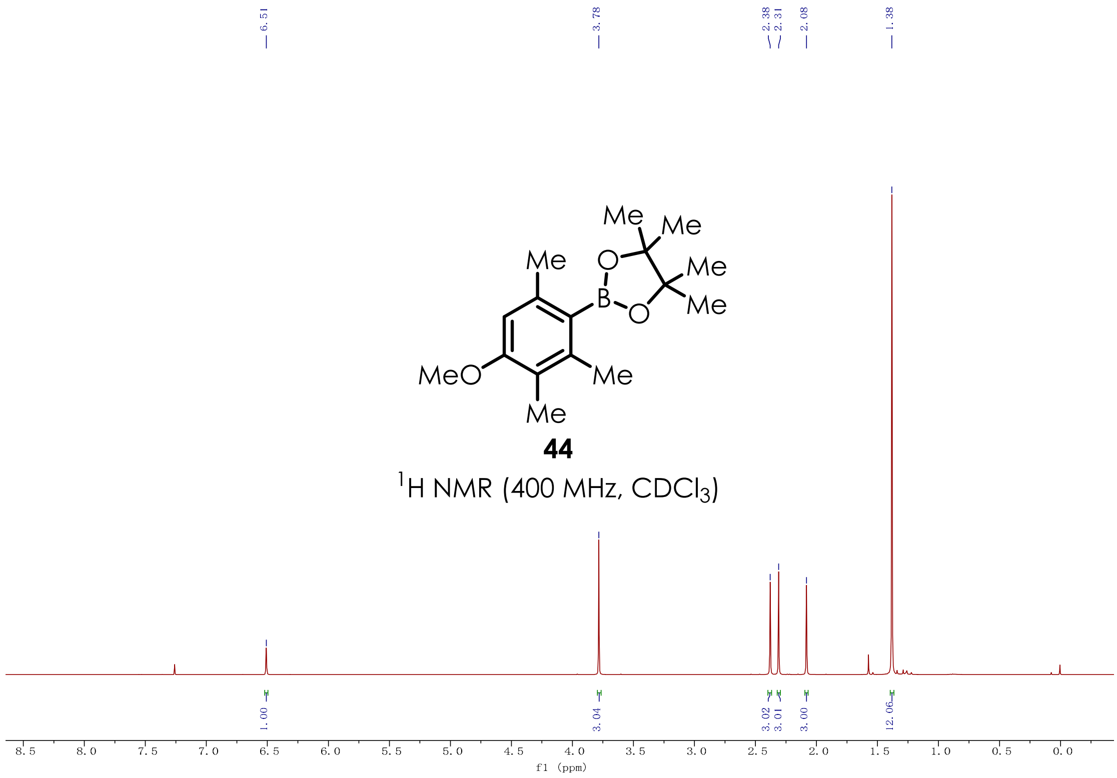

6.51
3.78
2.38
2.31
2.08
1.38
44
1H NMR (400 MHz, CDCl3)
1.00
3.04
3.02
3.01
3.00
12.06
f1 (ppm)

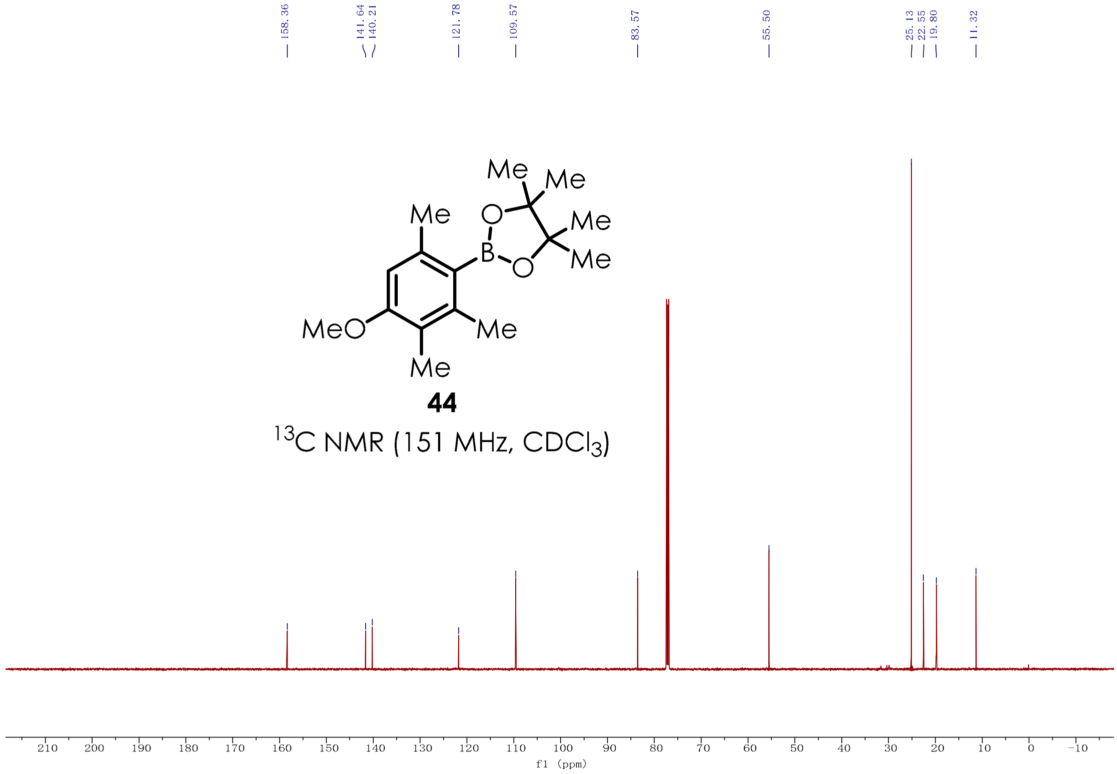

158.36
141.64
140.21
121.78
109.57
83.57
55.50
25.13
22.55
19.80
11.32
44
13C NMR (151 MHz, CDCl3)
f1 (ppm)

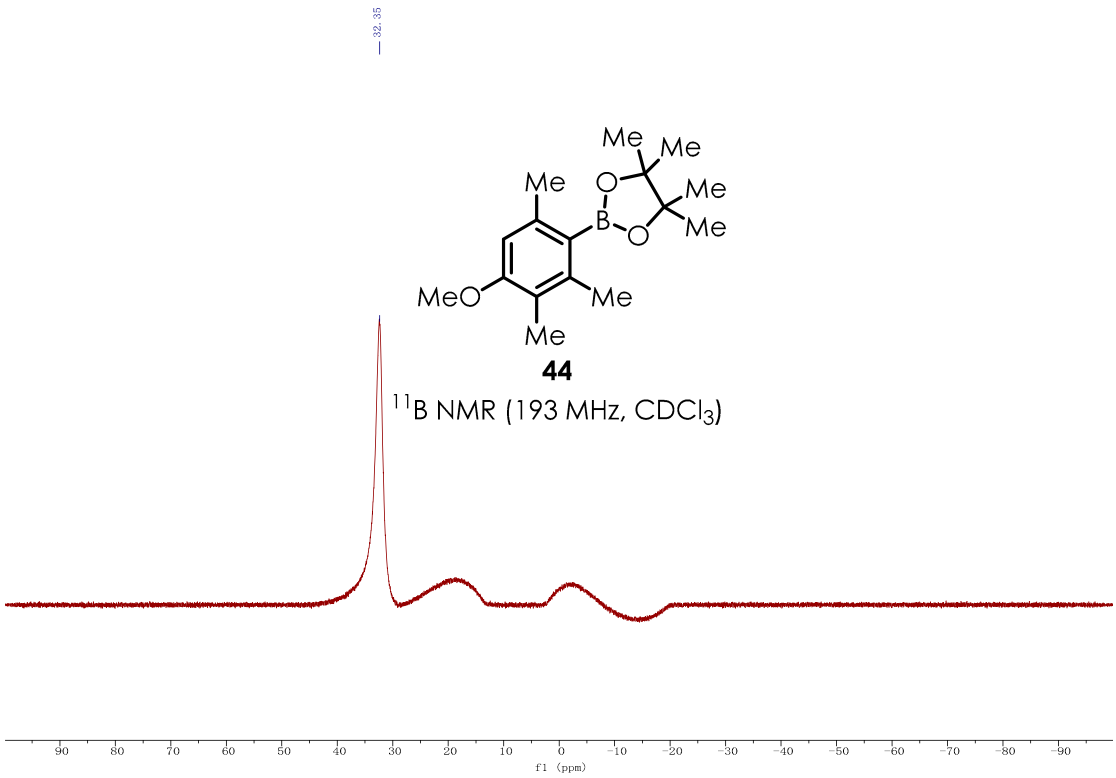

44

^{11}B NMR (193 MHz, $CDCl_3$)

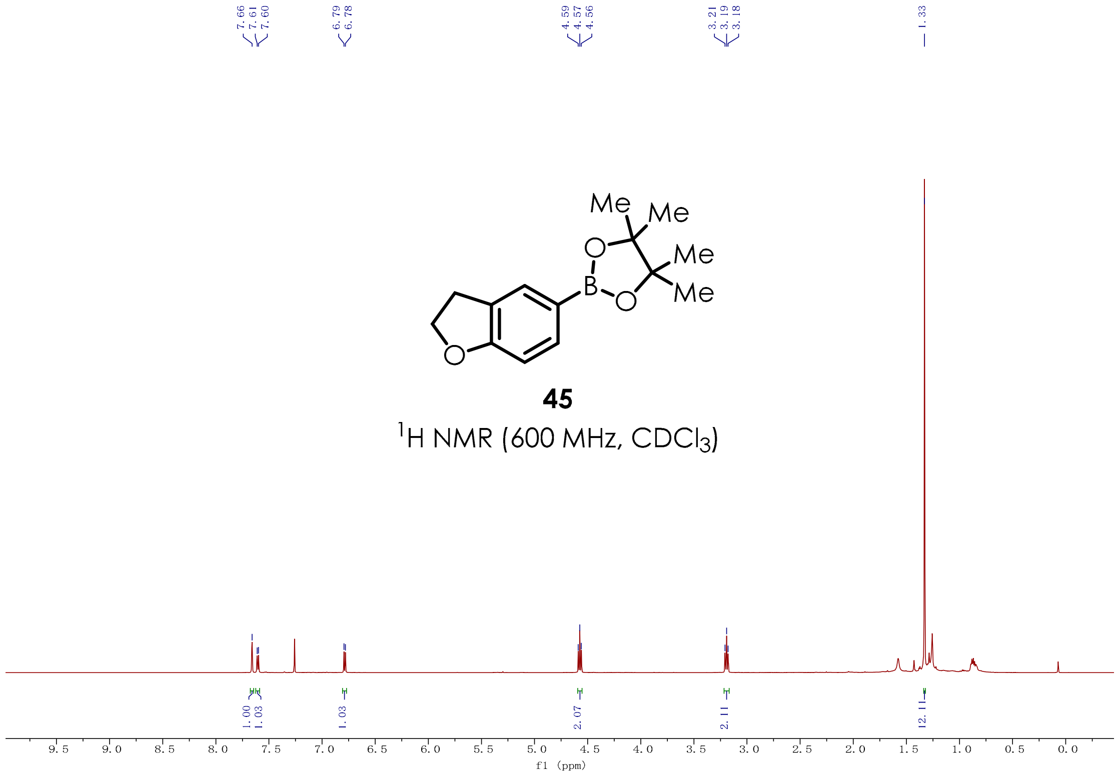

45

^{1}H NMR (600 MHz, $CDCl_3$)

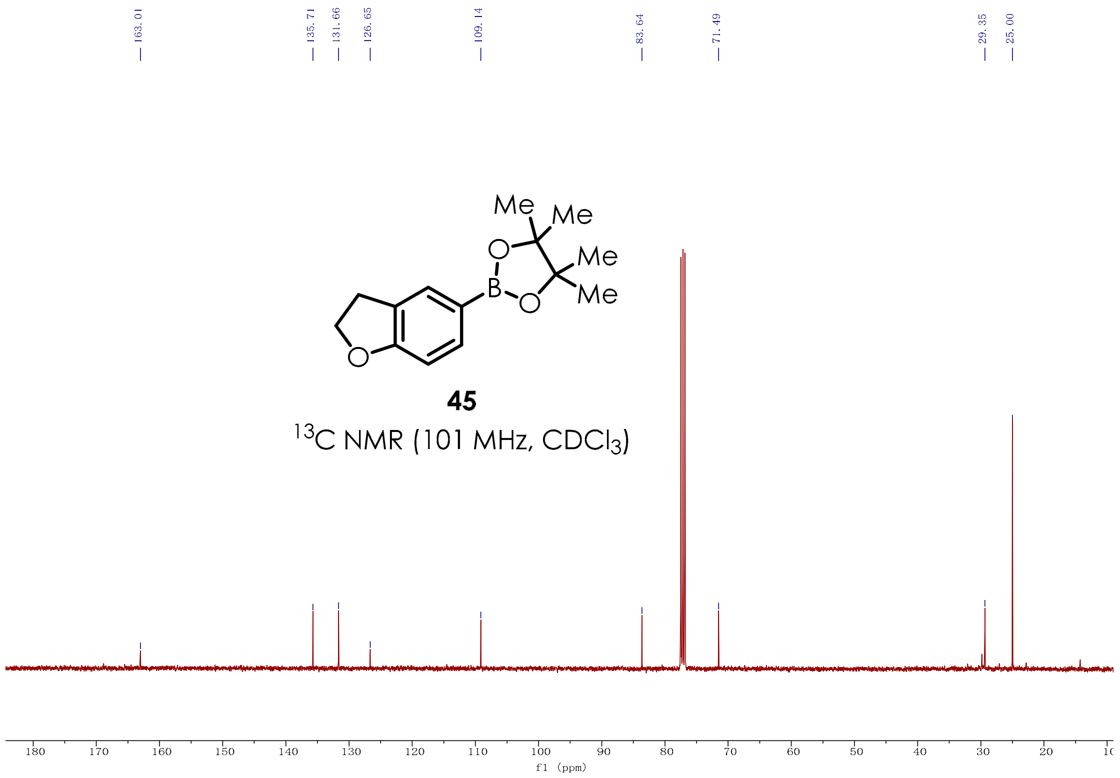

163.01
135.71
131.66
126.65
109.14
83.64
71.49
29.35
25.00
Me
Me
Me
Me
O
O
B
O
45
13C NMR (101 MHz, CDCl3)
180
170
160
150
140
130
120
110
100
90
80
70
60
50
40
30
20
f1 (ppm)

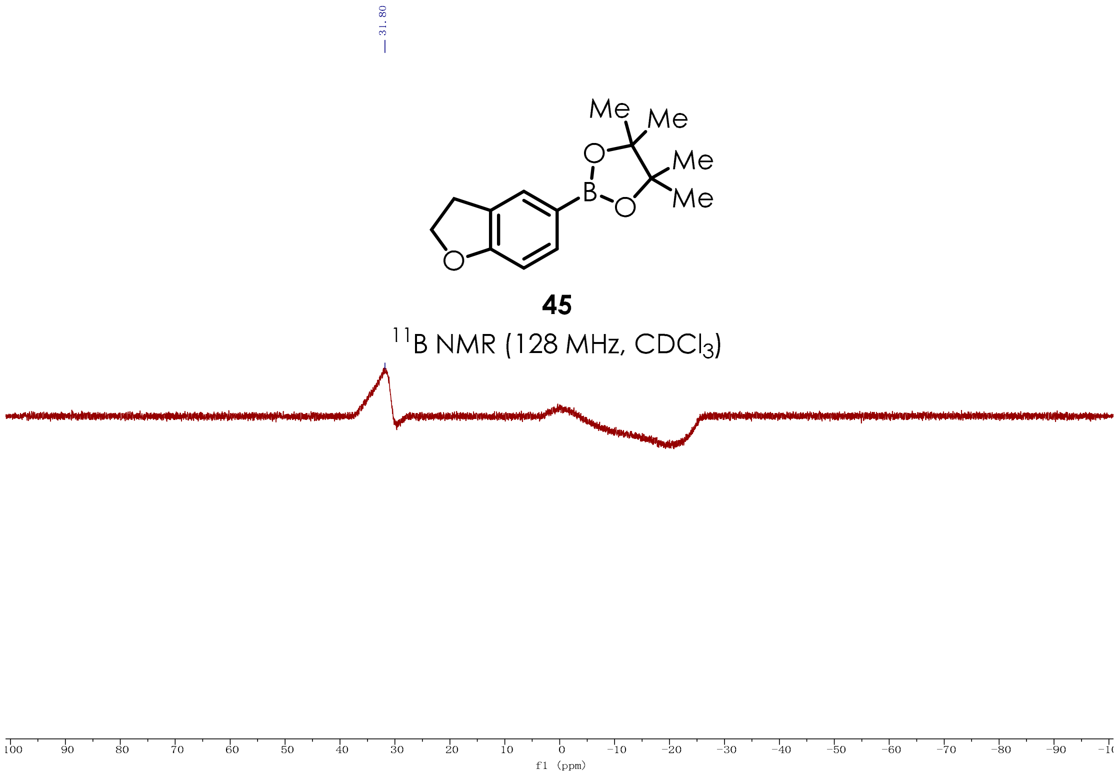

31.80
Me
Me
Me
Me
O
O
B
O
45
11B NMR (128 MHz, CDCl3)
100
90
80
70
60
50
40
30
20
10
0
-10
-20
-30
-40
-50
-60
-70
-80
-90
f1 (ppm)

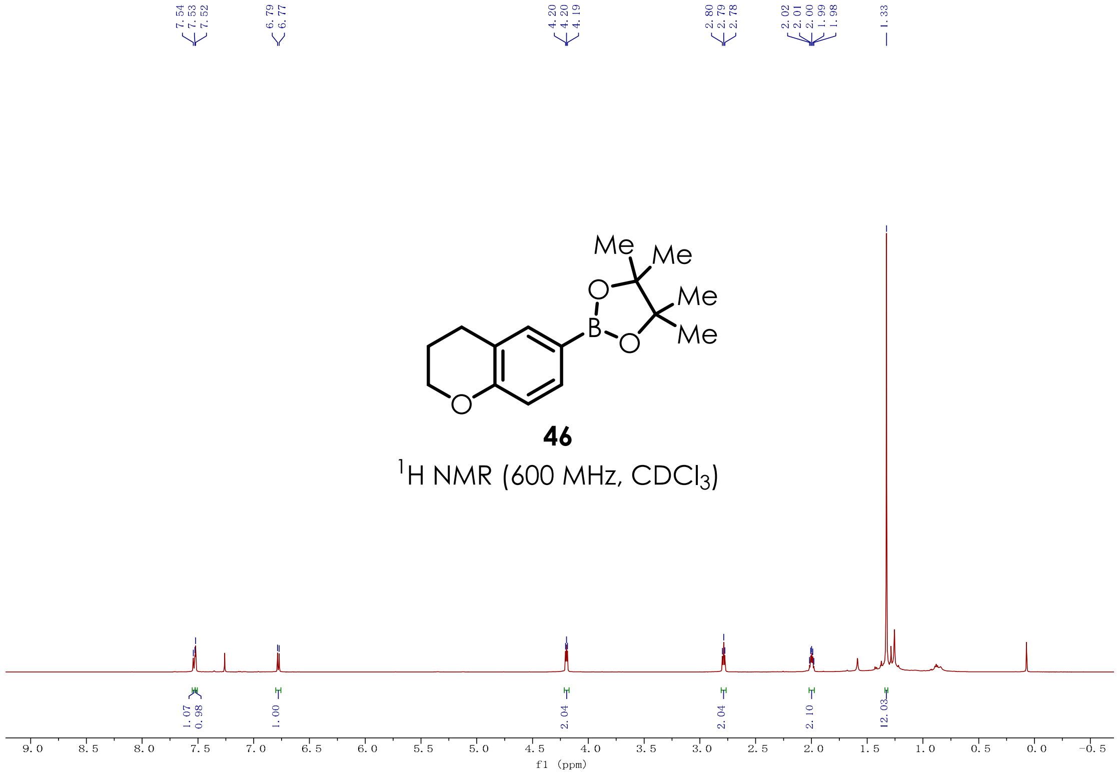
7.54
7.53
7.52
6.79
6.77
4.20
4.20
4.19
2.80
2.79
2.78
2.02
2.01
2.00
1.99
1.98
1.33
Me
Me
Me
Me
O
B
O
O
46
1H NMR (600 MHz, CDCl3)
1.07
0.98
1.00
2.04
2.04
2.10
12.03
9.0 8.5 8.0 7.5 7.0 6.5 6.0 5.5 5.0 4.5 4.0 3.5 3.0 2.5 2.0 1.5 1.0 0.5 0.0 -0.5
f1 (ppm)

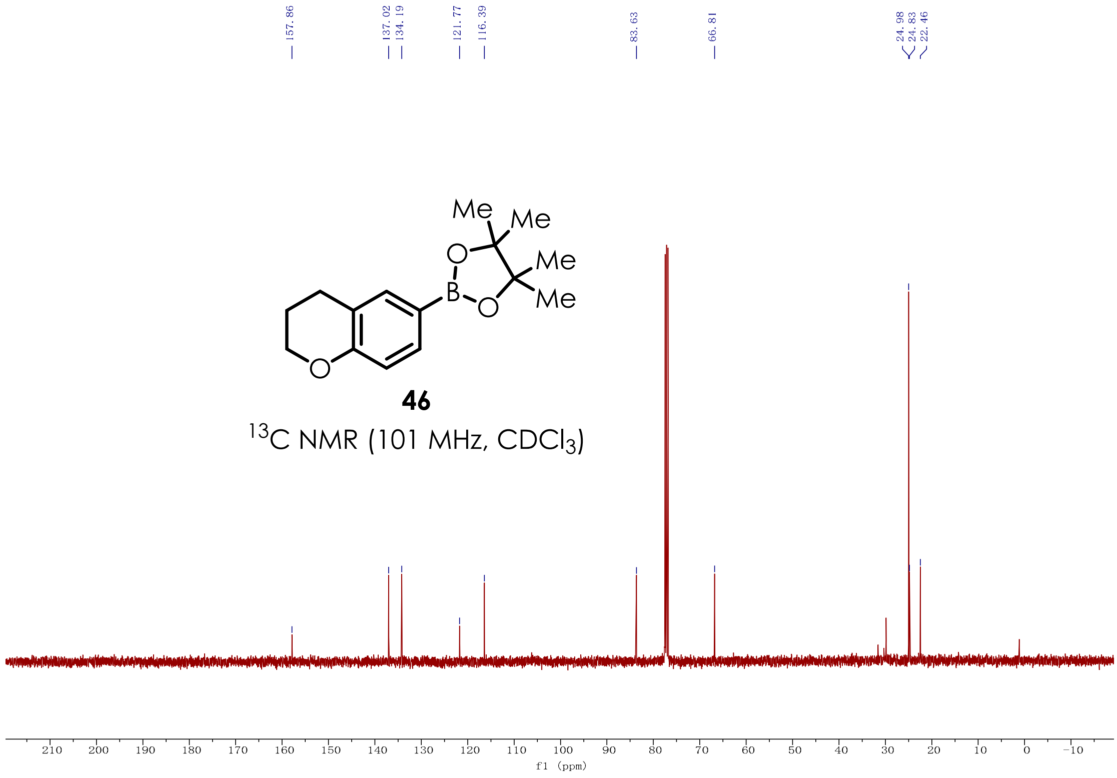
157.86
137.02
134.19
121.77
116.39
83.63
66.81
24.98
24.83
22.46
Me
Me
Me
Me
O
B
O
O
46
13C NMR (101 MHz, CDCl3)
210 200 190 180 170 160 150 140 130 120 110 100 90 80 70 60 50 40 30 20 10 0 -10
f1 (ppm)

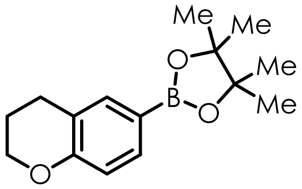

46

^{11}B NMR (193 MHz, $CDCl_3$)

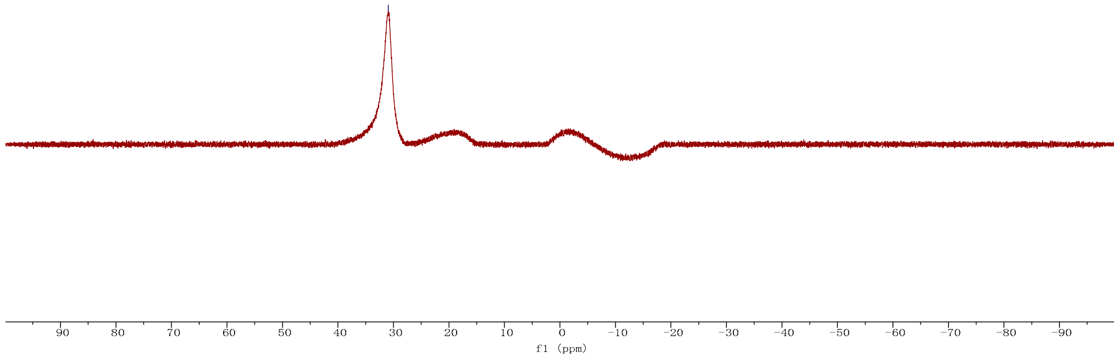

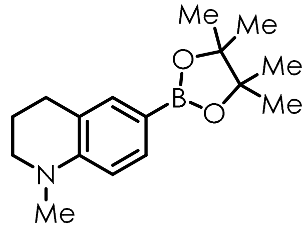

47

^{1}H NMR (400 MHz, $CDCl_3$)

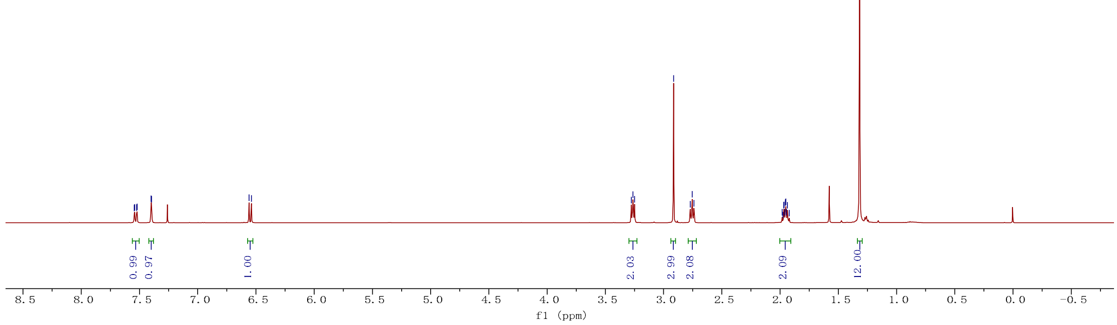

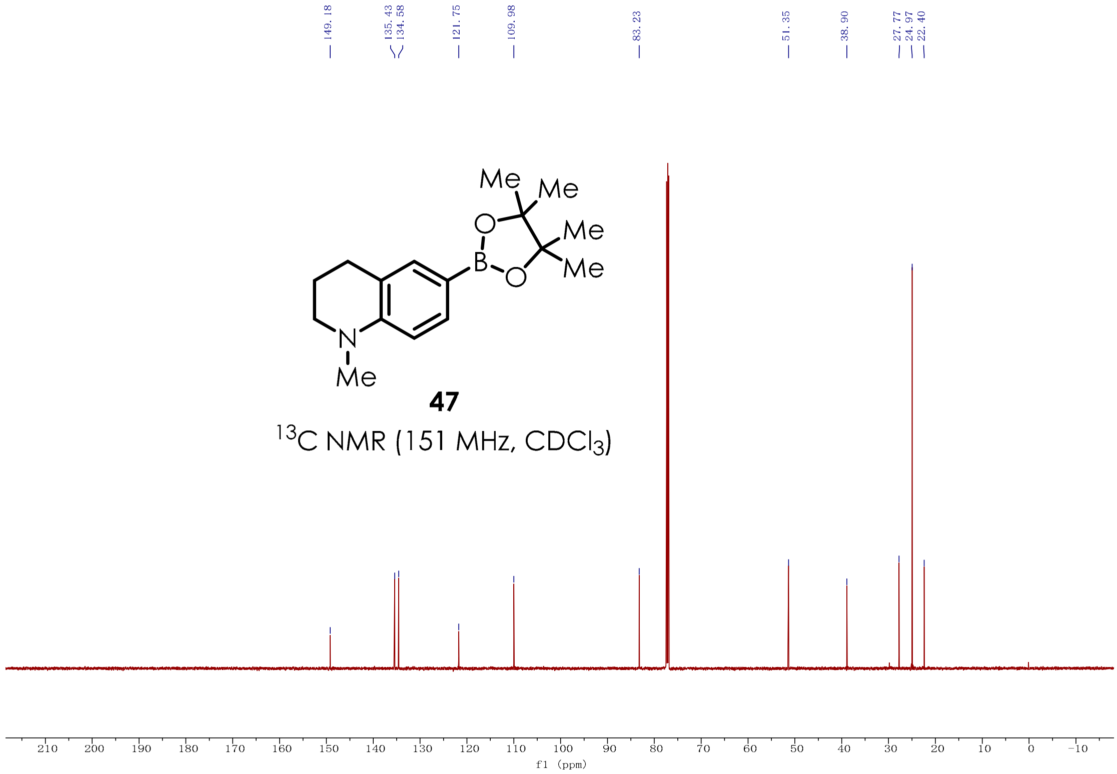
149.18
135.43
134.58
121.75
109.98
83.23
51.35
38.90
27.77
24.97
22.40
Me
Me
Me
Me
O
B
O
N
Me
47
13C NMR (151 MHz, CDCl3)
f1 (ppm)

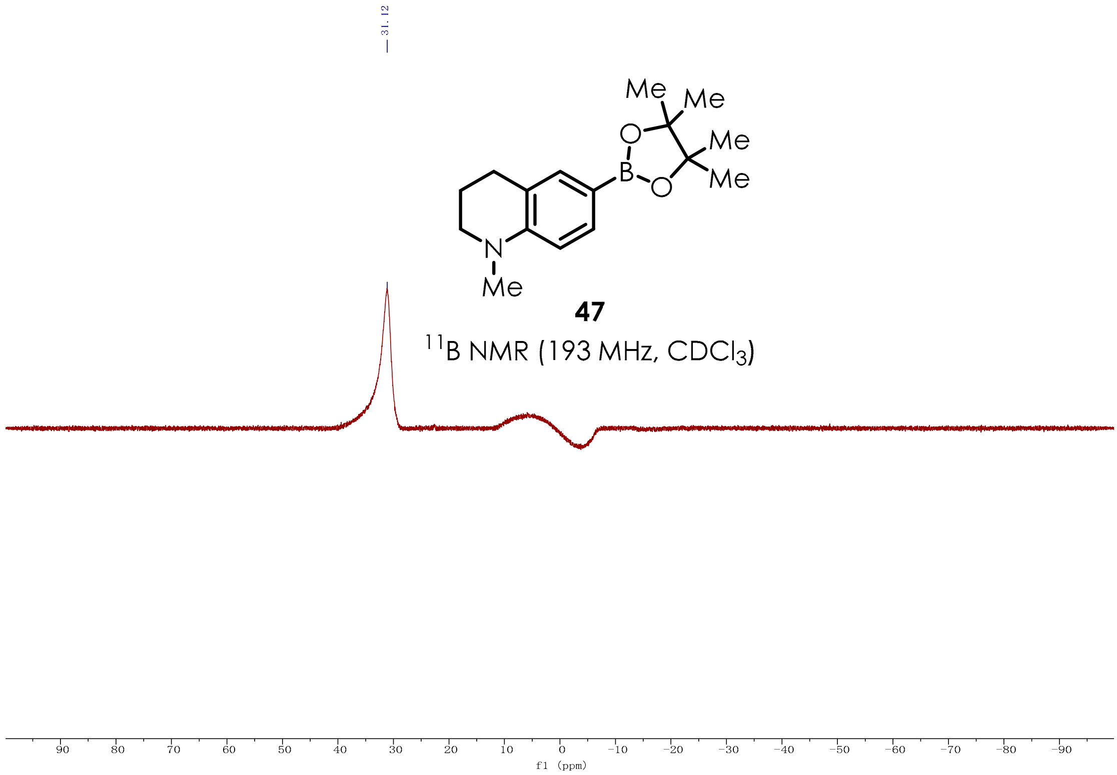
31.12
Me
Me
Me
Me
O
B
O
N
Me
47
11B NMR (193 MHz, CDCl3)
f1 (ppm)

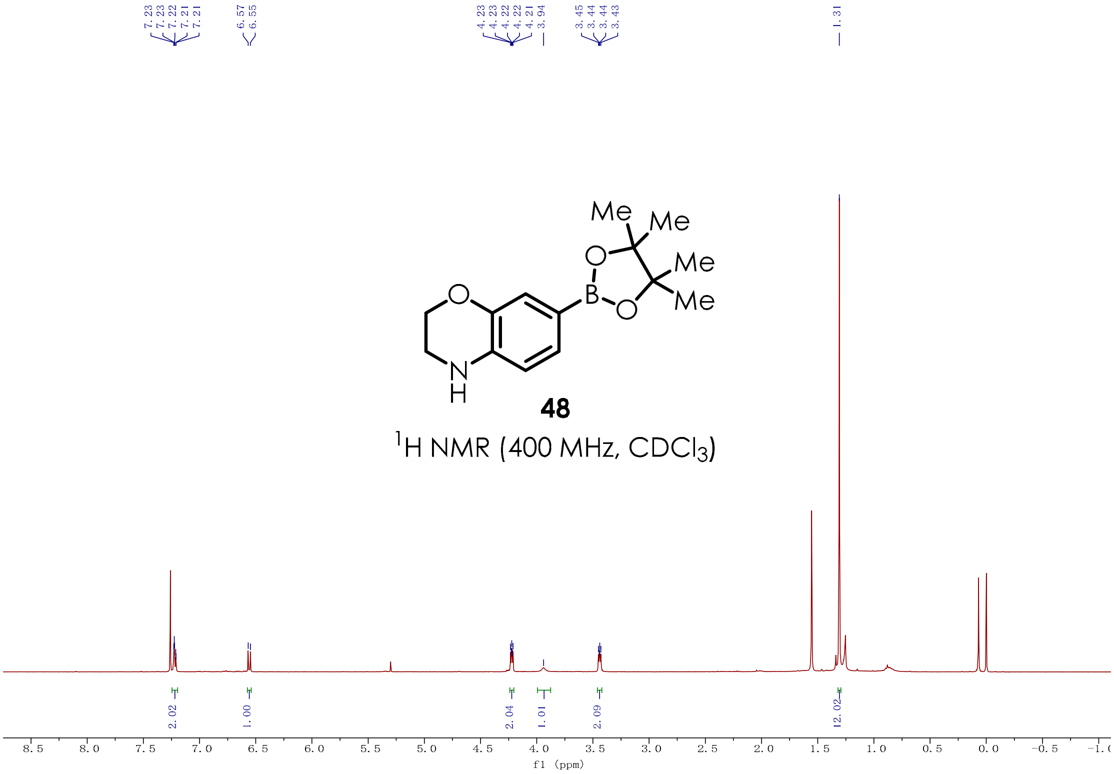

48

^{1}H NMR (400 MHz, CDCl$_3$)

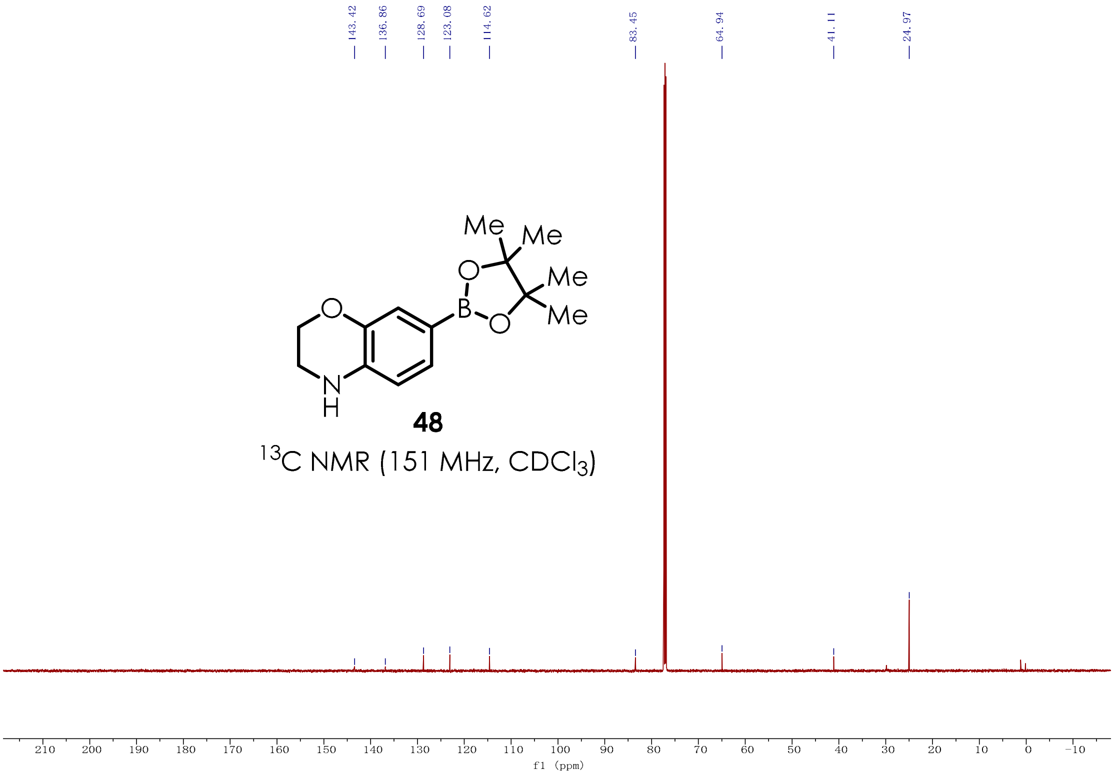

48

^{13}C NMR (151 MHz, CDCl$_3$)

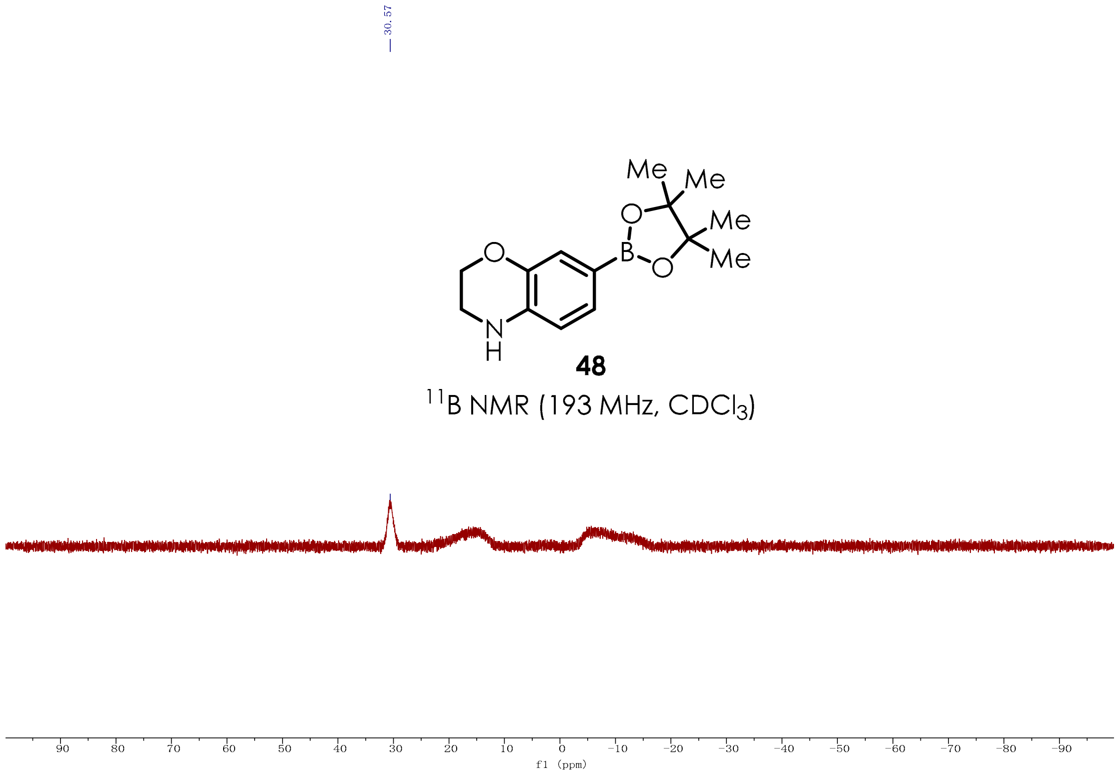

^{11}B NMR (193 MHz, $CDCl_3$)

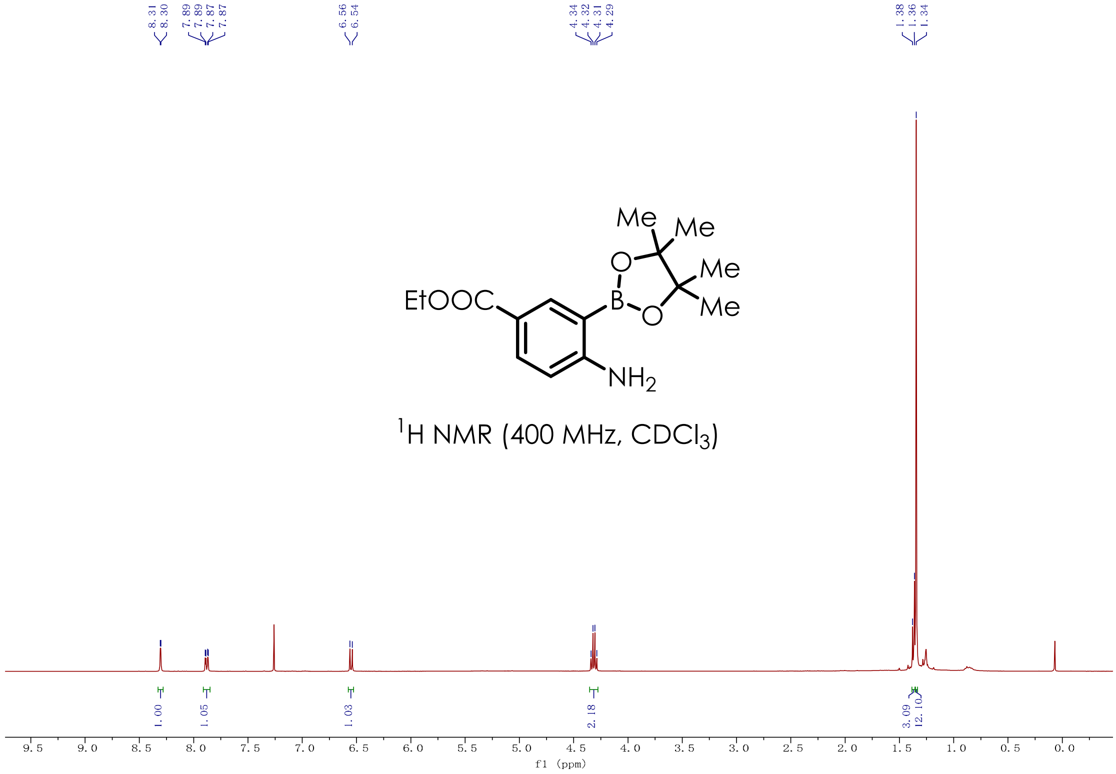

^{1}H NMR (400 MHz, $CDCl_3$)

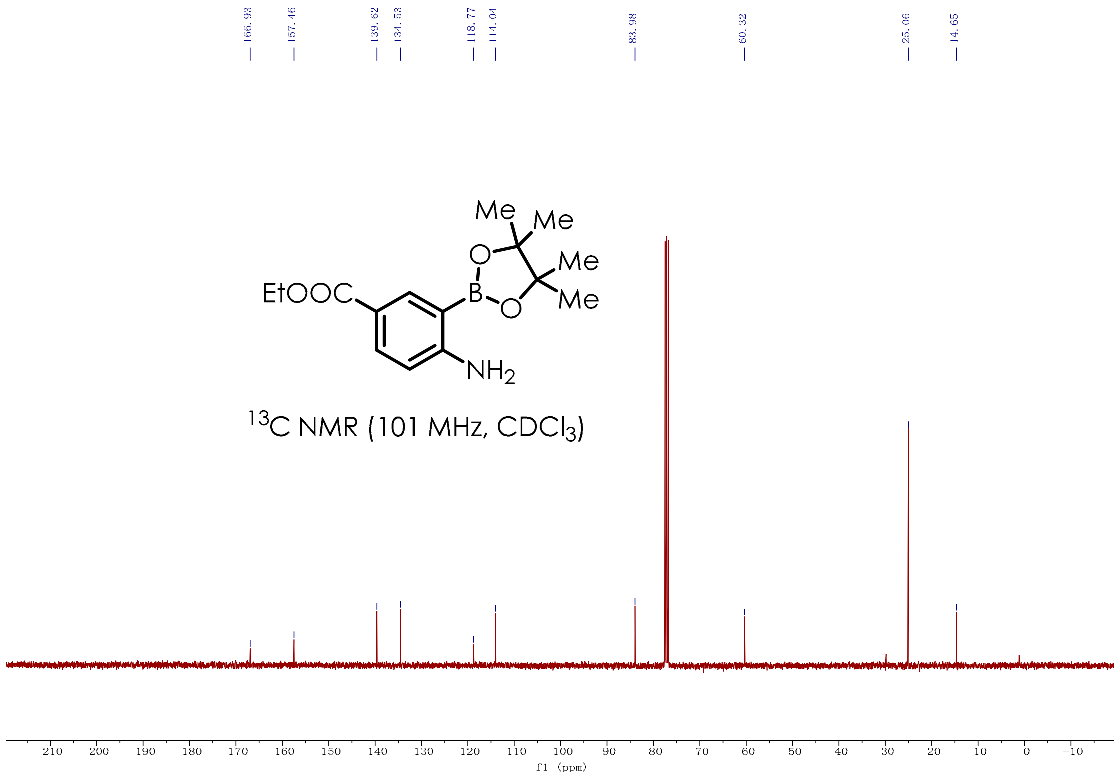
166.93
157.46
139.62
134.53
118.77
114.04
83.98
60.32
25.06
14.65
Me
Me
Me
Me
EtOOC
B
O
O
NH2
13C NMR (101 MHz, CDCl3)
f1 (ppm)

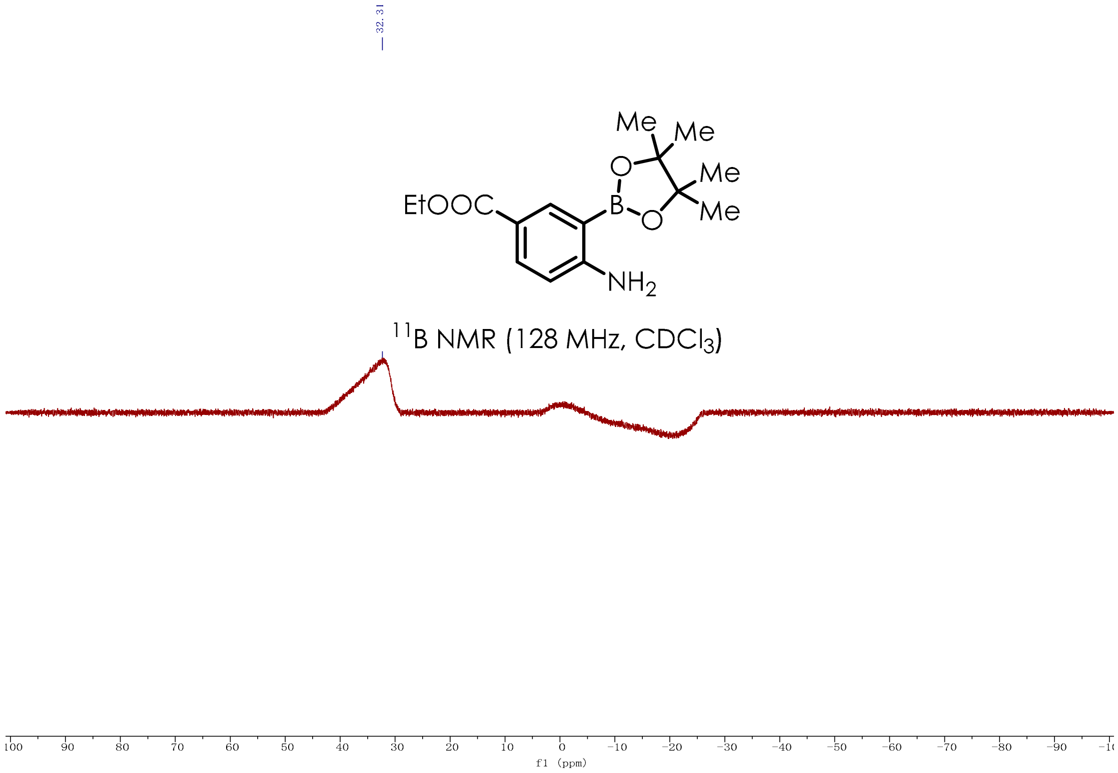
32.31
Me
Me
Me
Me
EtOOC
B
O
O
NH2
11B NMR (128 MHz, CDCl3)
f1 (ppm)

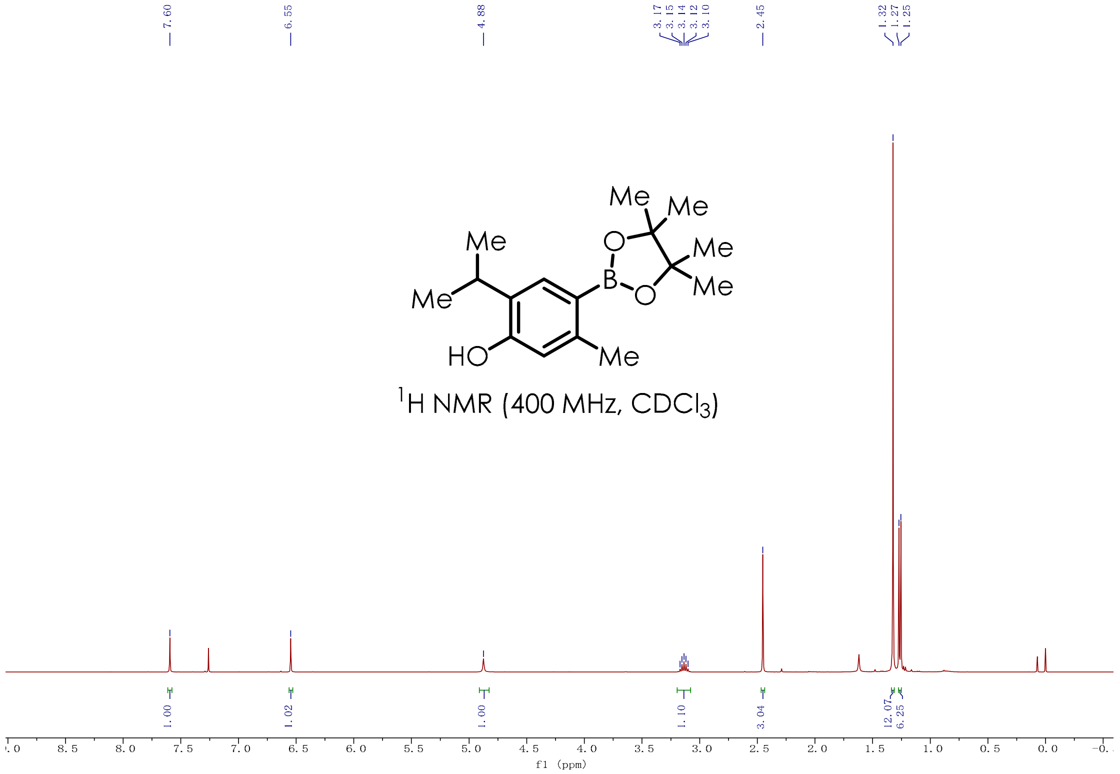
7.60
6.55
4.88
3.17
3.15
3.14
3.12
3.10
2.45
1.32
1.27
1.25
Me
Me
O
O
B
Me
Me
Me
Me
HO
Me
1H NMR (400 MHz, CDCl3)
1.00
1.02
1.00
1.10
3.04
12.07
6.25
f1 (ppm)

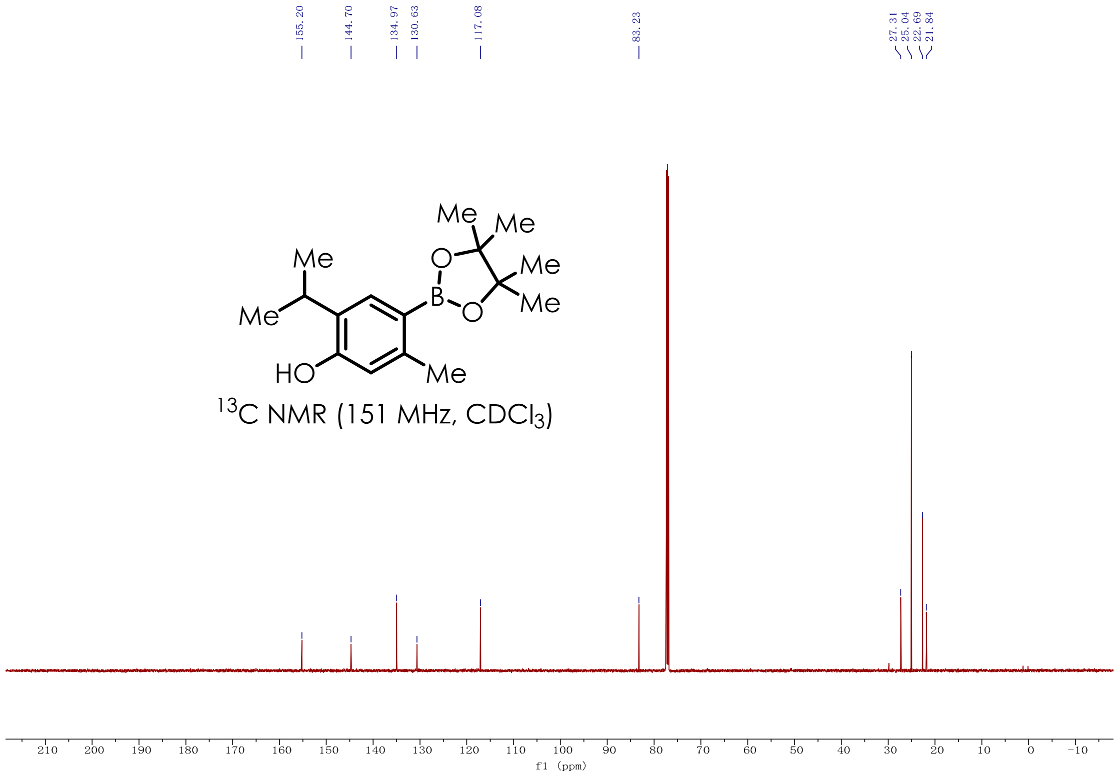
155.20
144.70
134.97
130.63
117.08
83.23
27.31
25.04
22.69
21.84
Me
Me
O
O
B
Me
Me
Me
Me
HO
Me
13C NMR (151 MHz, CDCl3)
f1 (ppm)

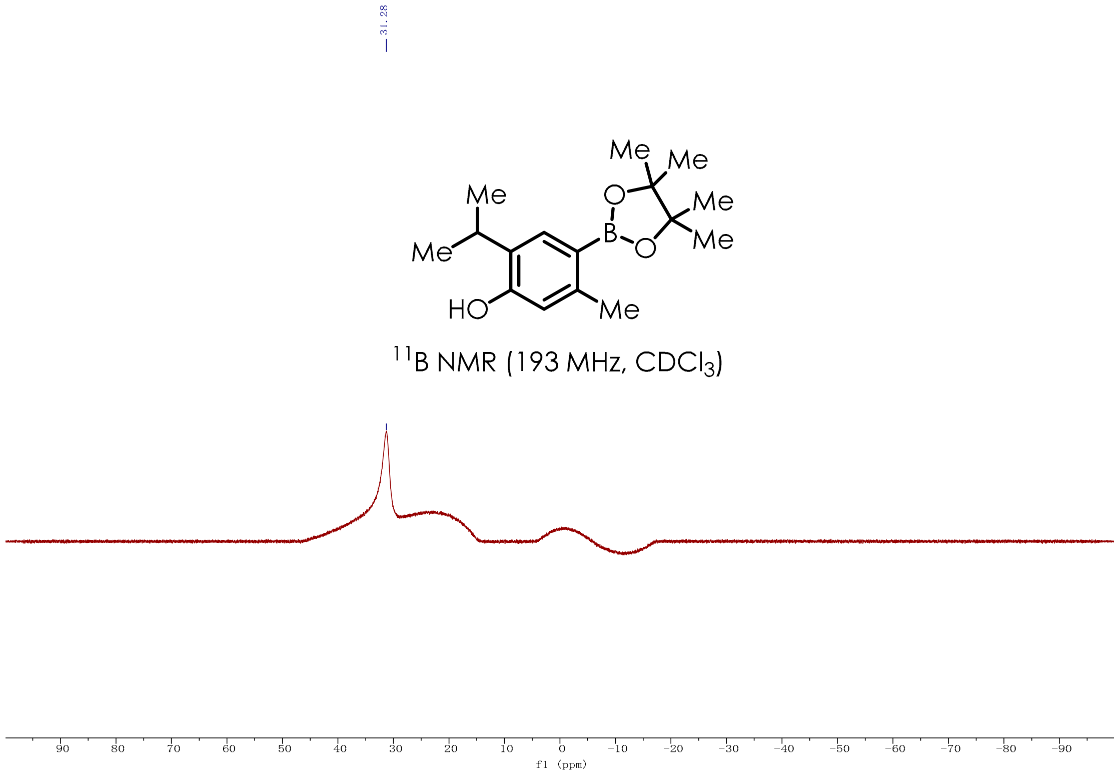

31.28
Me
Me
Me
Me
Me
Me
HO
Me
B
O
O
^{11}B NMR (193 MHz, CDCl$_3$)
f1 (ppm)

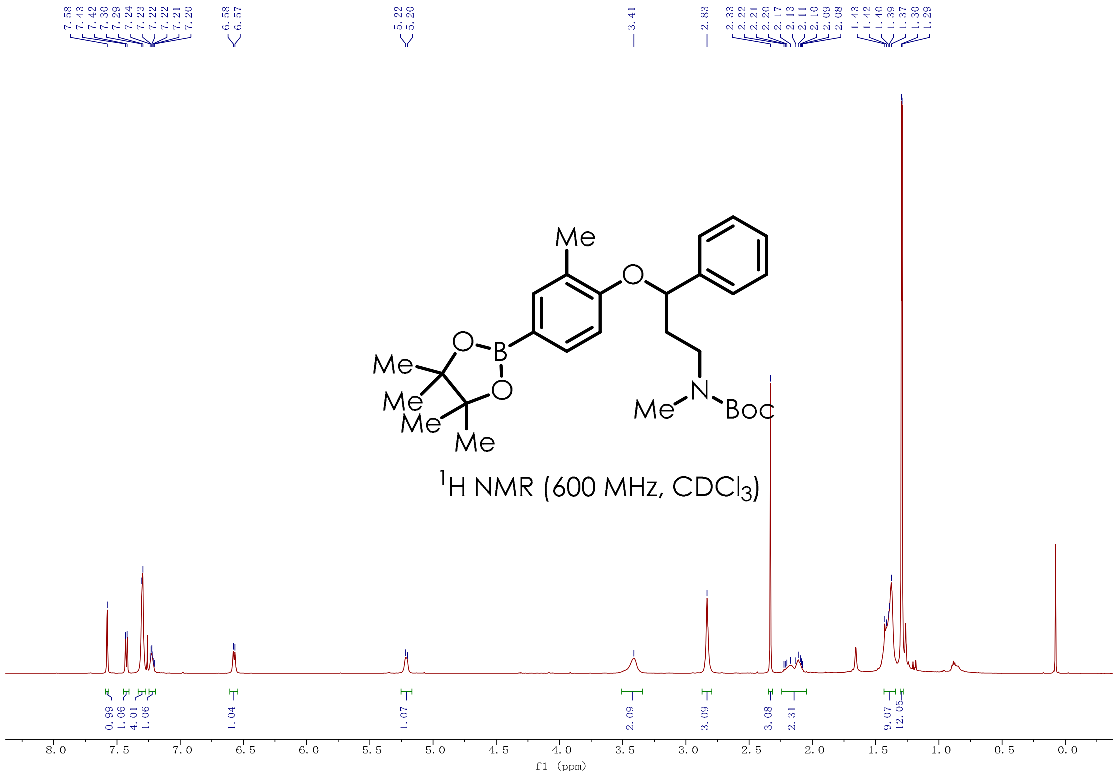

Me
O
B
O
O
Me
Me
Me
Me
Me
N
Boc
^{1}H NMR (600 MHz, CDCl$_3$)
f1 (ppm)

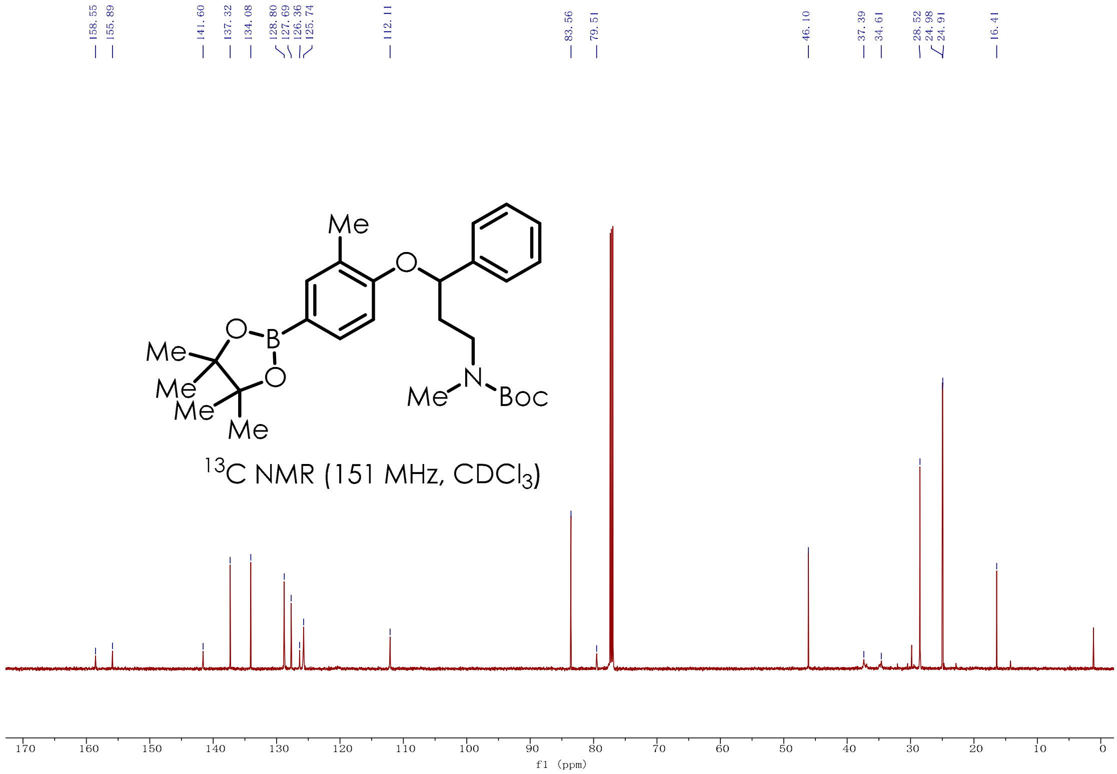

158.55
155.89
141.60
137.32
134.08
128.80
127.69
126.36
125.74
112.11
83.56
79.51
46.10
37.39
34.61
28.52
24.98
24.91
16.41
Me
O
B
Me
Me
Me
Me
N
Boc
^{13}C NMR (151 MHz, CDCl$_3$)
f1 (ppm)

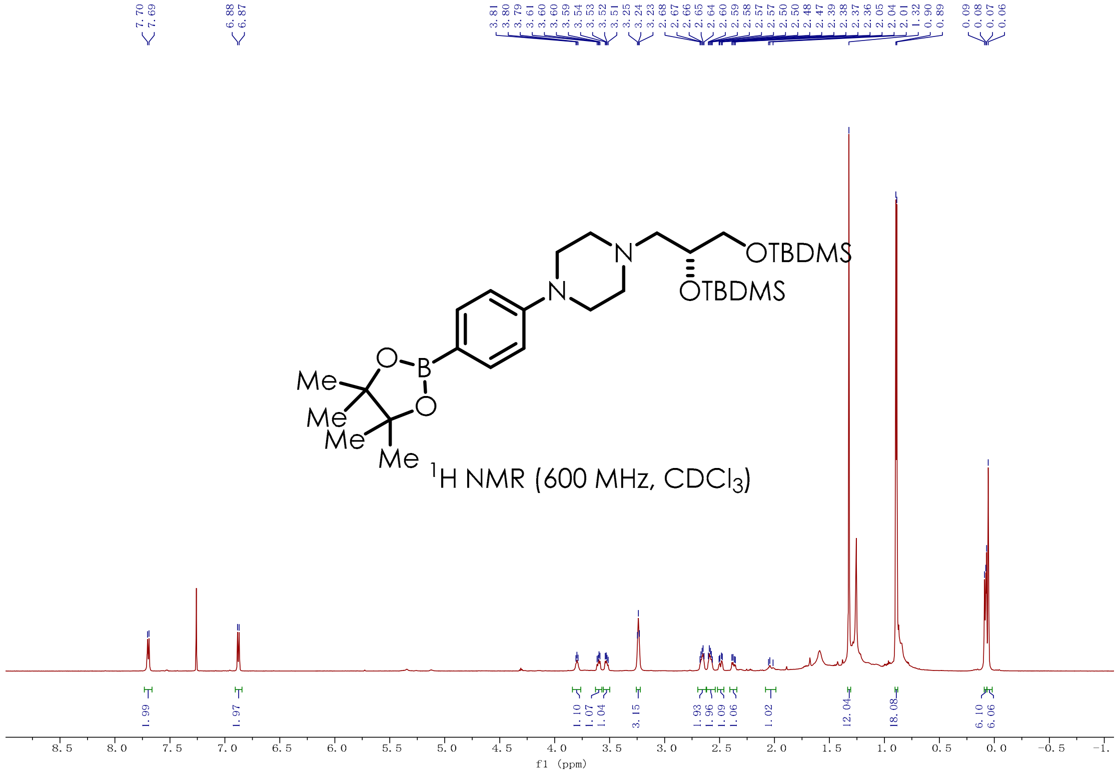

7.70
7.69
6.88
6.87
N
OTBDMS
OTBDMS
O
B
Me
Me
Me
Me
^{1}H NMR (600 MHz, CDCl$_3$)
1.99
1.97
1.10
1.07
1.04
3.15
1.93
1.96
1.09
1.06
1.02
12.04
18.08
6.10
6.06
f1 (ppm)

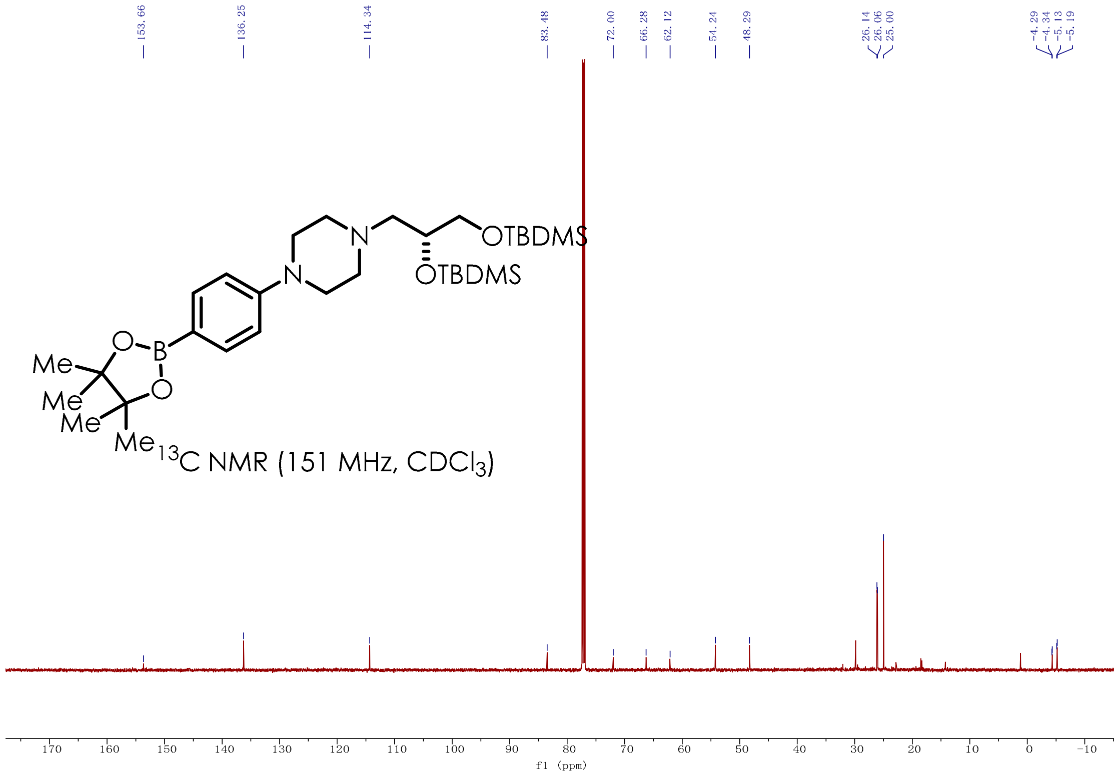

153.66
136.25
114.34
83.48
72.00
66.28
62.12
54.24
48.29
26.14
26.06
25.00
-4.29
-4.34
-5.13
-5.19
OTBDMS
OTBDMS
N
N
B
O
O
Me
Me
Me
Me
13C NMR (151 MHz, CDCl3)
f1 (ppm)

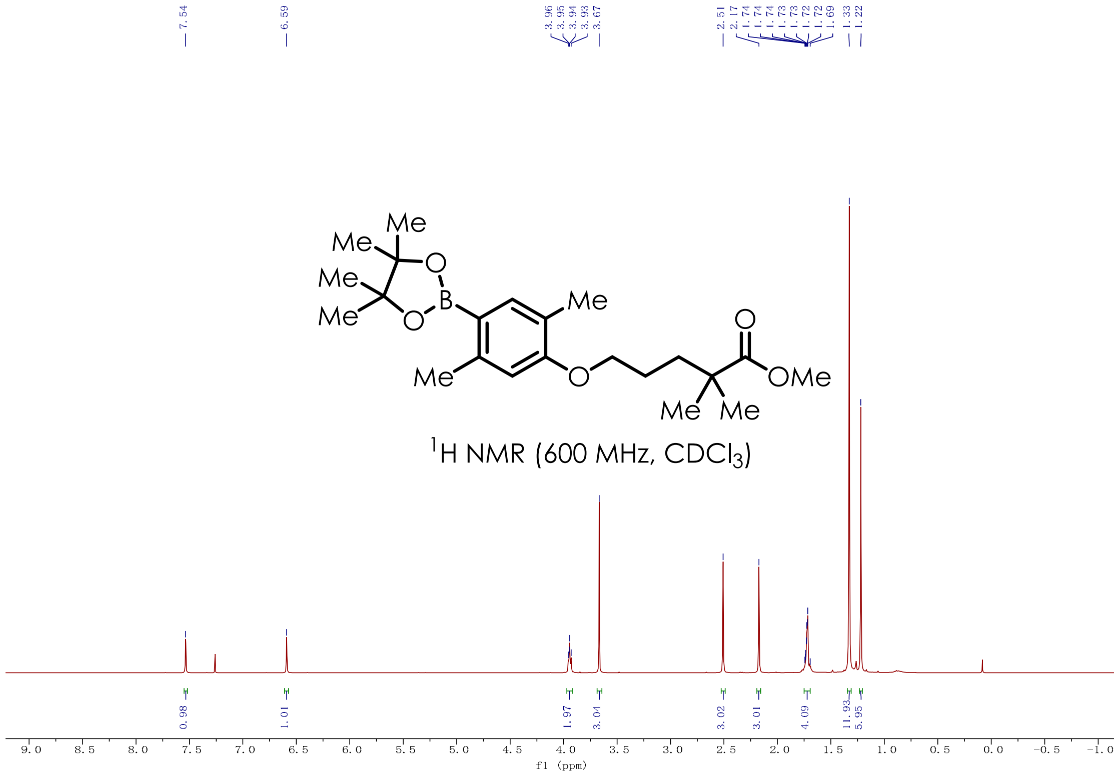

7.54
6.59
3.96
3.95
3.94
3.93
3.67
2.51
2.17
1.74
1.74
1.73
1.73
1.72
1.72
1.69
1.33
1.22
Me
Me
Me
Me
B
O
O
Me
Me
O
O
OMe
Me
Me
1H NMR (600 MHz, CDCl3)
0.98
1.01
1.97
3.04
3.02
3.01
4.09
11.93
5.95
f1 (ppm)

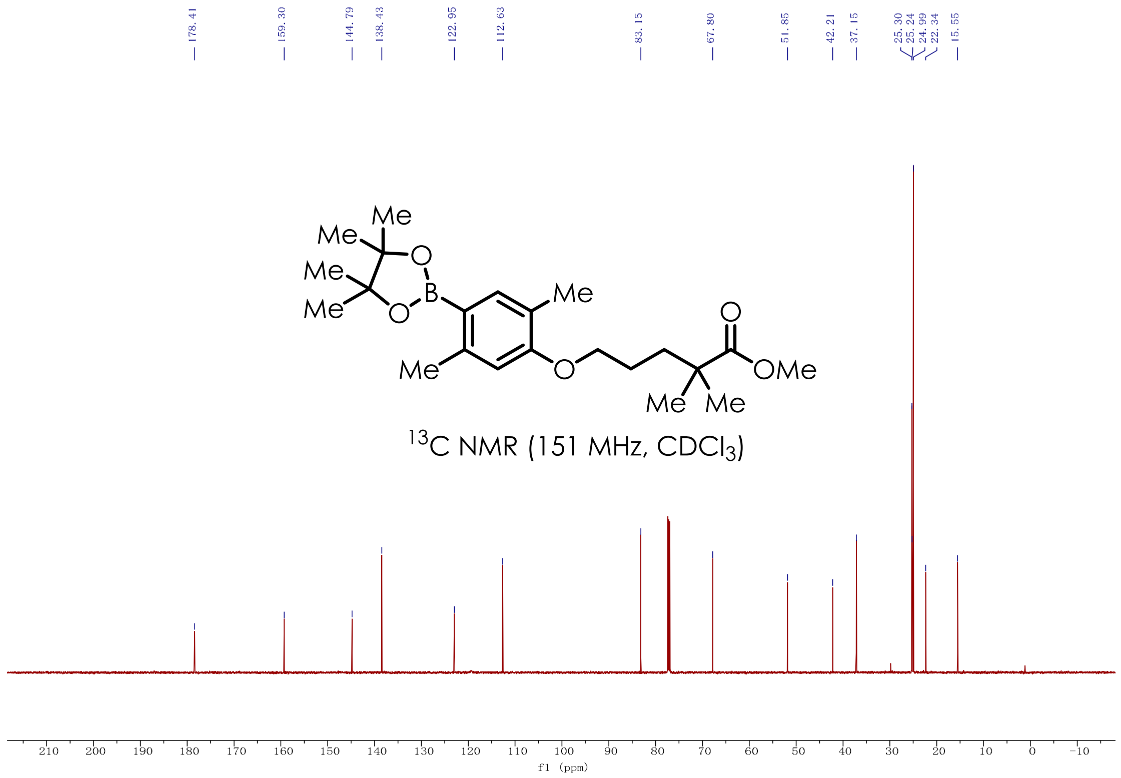
178.41
159.30
144.79
138.43
122.95
112.63
83.15
67.80
51.85
42.21
37.15
25.30
25.24
24.99
22.34
15.55
Me
Me
Me
Me
O
B
O
Me
Me
O
O
OMe
Me Me
13C NMR (151 MHz, CDCl3)
210 200 190 180 170 160 150 140 130 120 110 100 90 80 70 60 50 40 30 20 10 0 -10
f1 (ppm)

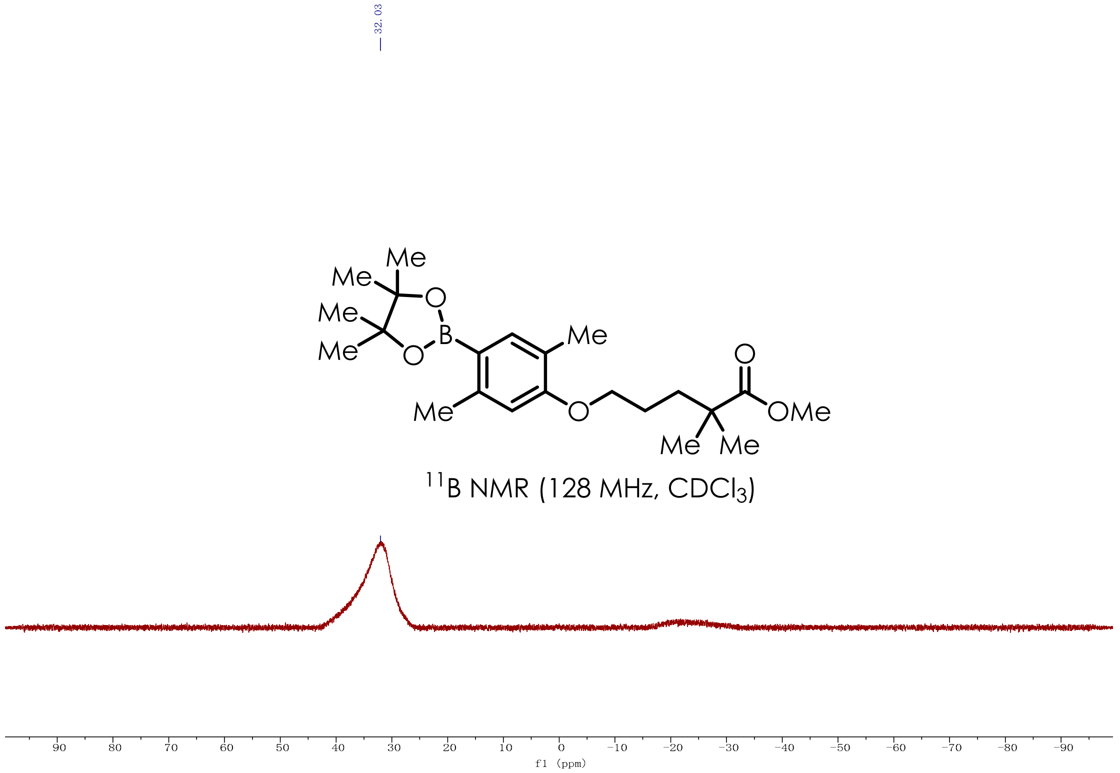
32.03
Me
Me
Me
Me
O
B
O
Me
Me
O
O
OMe
Me Me
11B NMR (128 MHz, CDCl3)
90 80 70 60 50 40 30 20 10 0 -10 -20 -30 -40 -50 -60 -70 -80 -90
f1 (ppm)

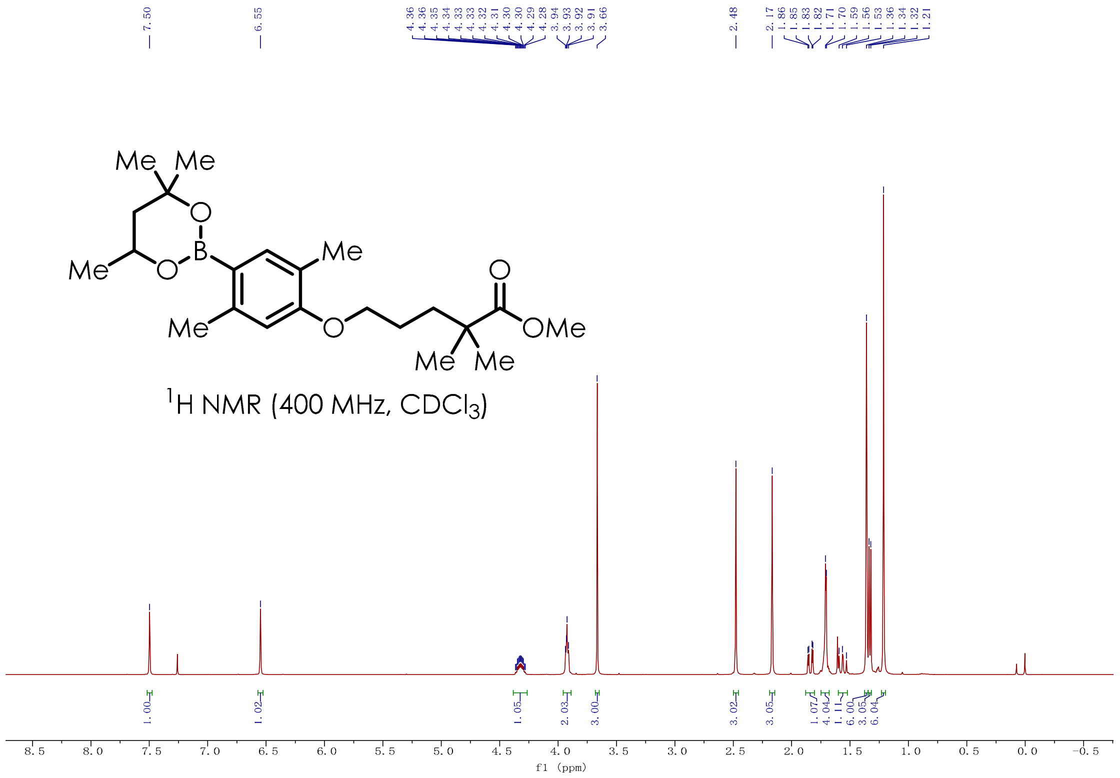

7.50
6.55
4.36 4.36 4.35 4.34 4.33 4.33 4.32 4.31 4.30 4.30 4.29 4.28 3.94 3.93 3.92 3.91 3.66
2.48
2.17 1.86 1.85 1.83 1.82 1.71 1.70 1.59 1.56 1.53 1.36 1.34 1.32 1.21
Me Me
O
B
O
Me
Me
Me
O
O
OMe
Me Me
^{1}H NMR (400 MHz, CDCl$_3$)
1.00
1.02
1.05
2.03
3.00
3.02
3.05
1.07
4.04
1.11
6.00
3.05
6.04
f1 (ppm)

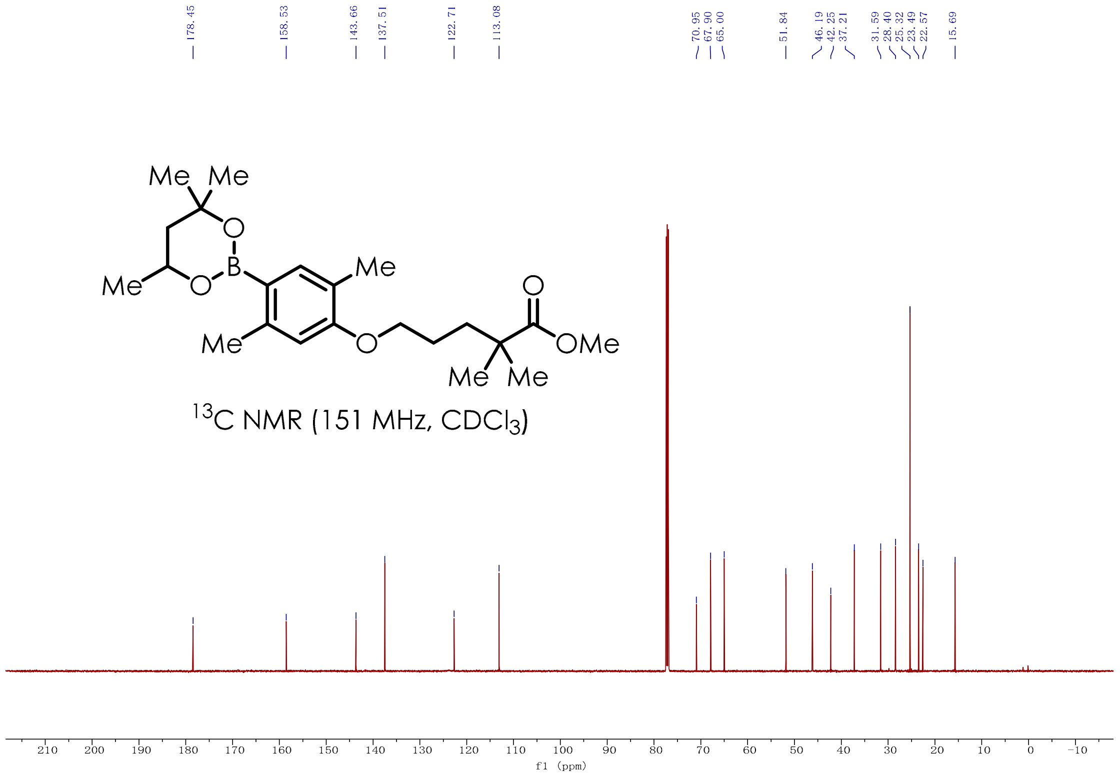

178.45
158.53
143.66
137.51
122.71
113.08
70.95 67.90 65.00
51.84
46.19 42.25 37.21
31.59 28.40 25.32 23.49 22.57
15.69
Me Me
O
B
O
Me
Me
Me
O
O
OMe
Me Me
^{13}C NMR (151 MHz, CDCl$_3$)
f1 (ppm)

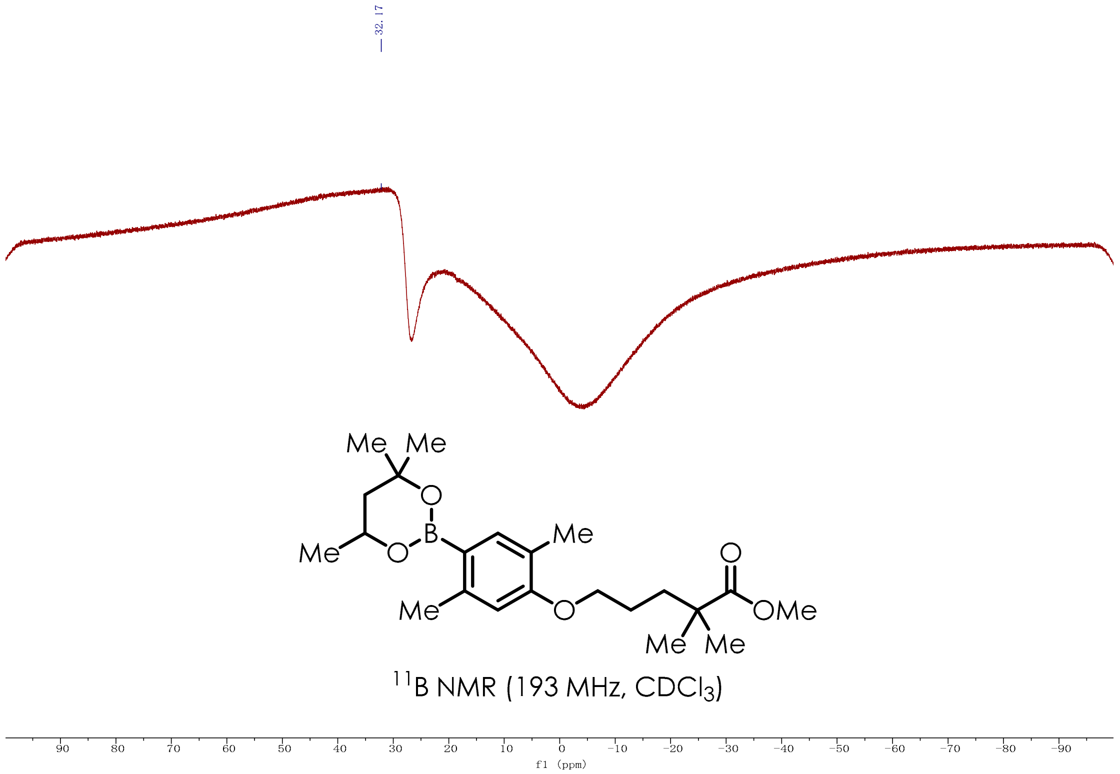
32.17
Me
Me
O
B
O
Me
Me
Me
O
O
OMe
Me
Me
11B NMR (193 MHz, CDCl3)
f1 (ppm)

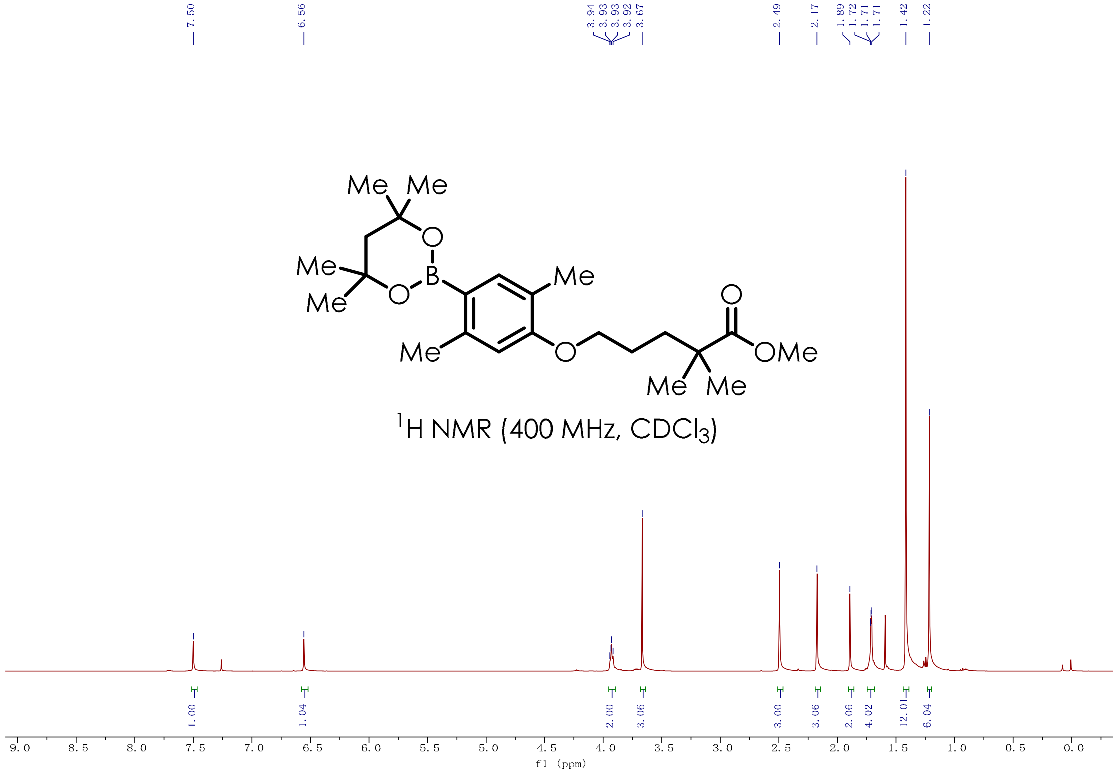
7.50
6.56
3.94
3.93
3.93
3.92
3.67
2.49
2.17
1.89
1.72
1.71
1.71
1.42
1.22
1H NMR (400 MHz, CDCl3)
1.00
1.04
2.00
3.06
3.00
3.06
2.06
4.02
12.01
6.04
f1 (ppm)

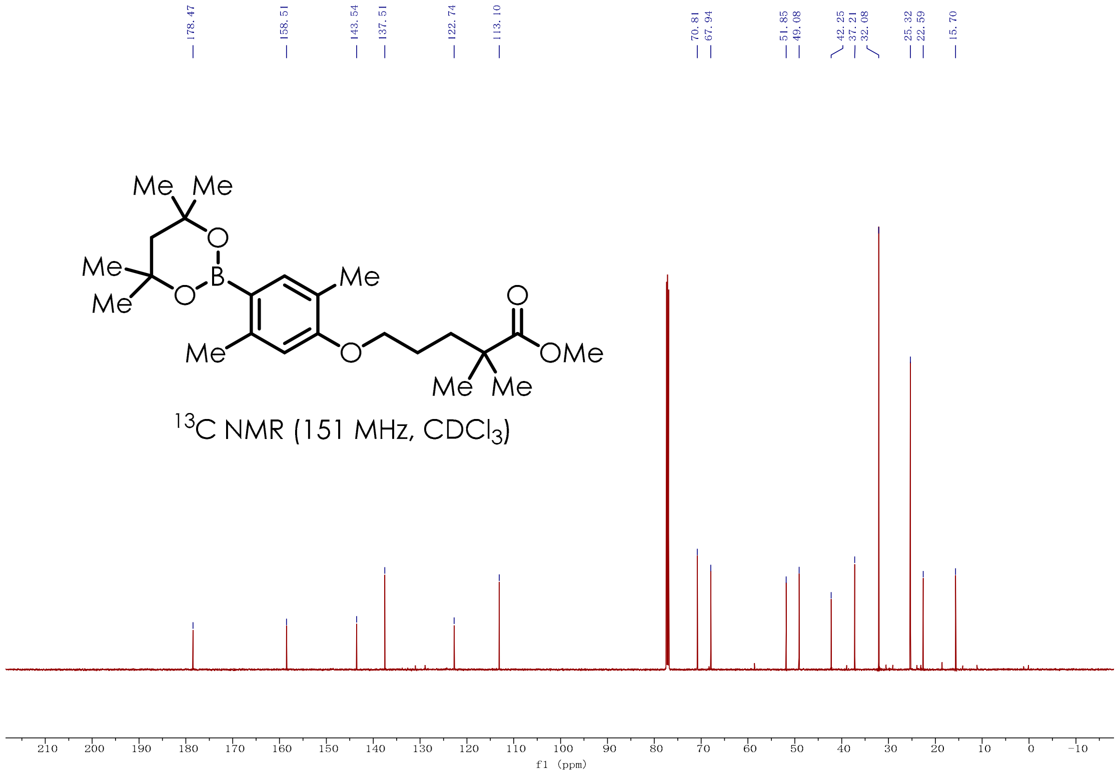
178.47
158.51
143.54
137.51
122.74
113.10
70.81
67.94
51.85
49.08
42.25
37.21
32.08
25.32
22.59
15.70
Me Me
O
Me
B
Me
O
Me
O
Me
O
OMe
Me Me
13C NMR (151 MHz, CDCl3)
f1 (ppm)

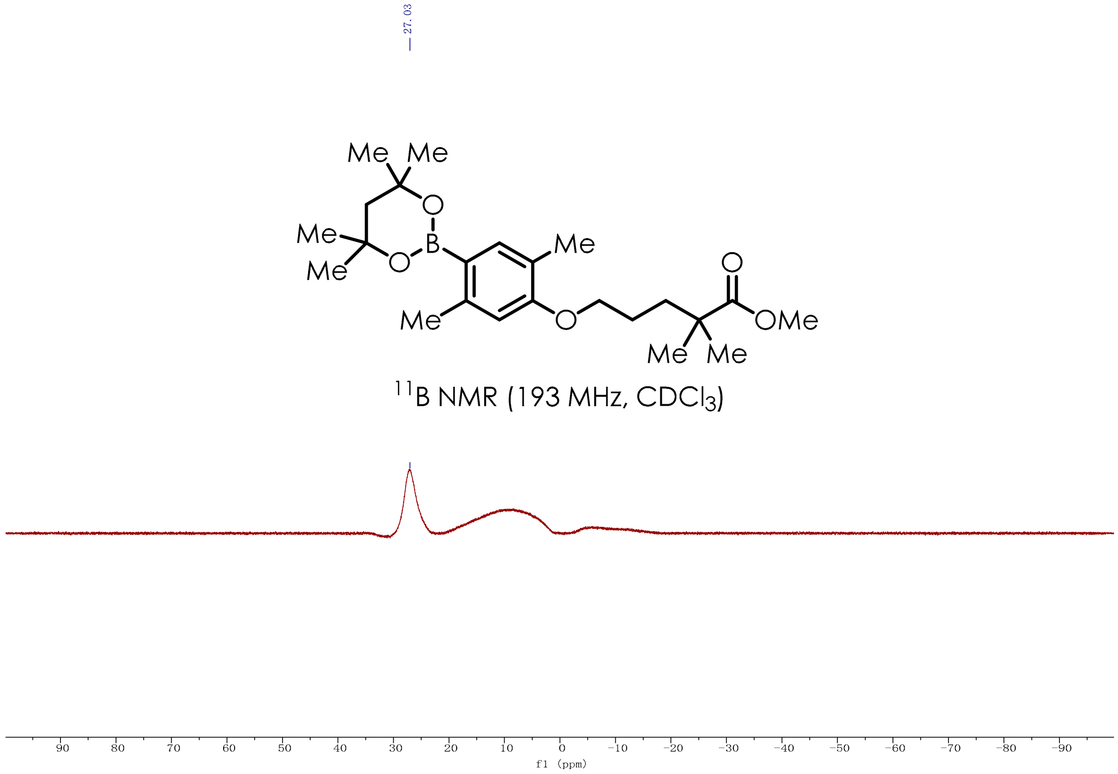
27.03
Me Me
O
Me
B
Me
O
Me
O
Me
O
OMe
Me Me
11B NMR (193 MHz, CDCl3)
f1 (ppm)

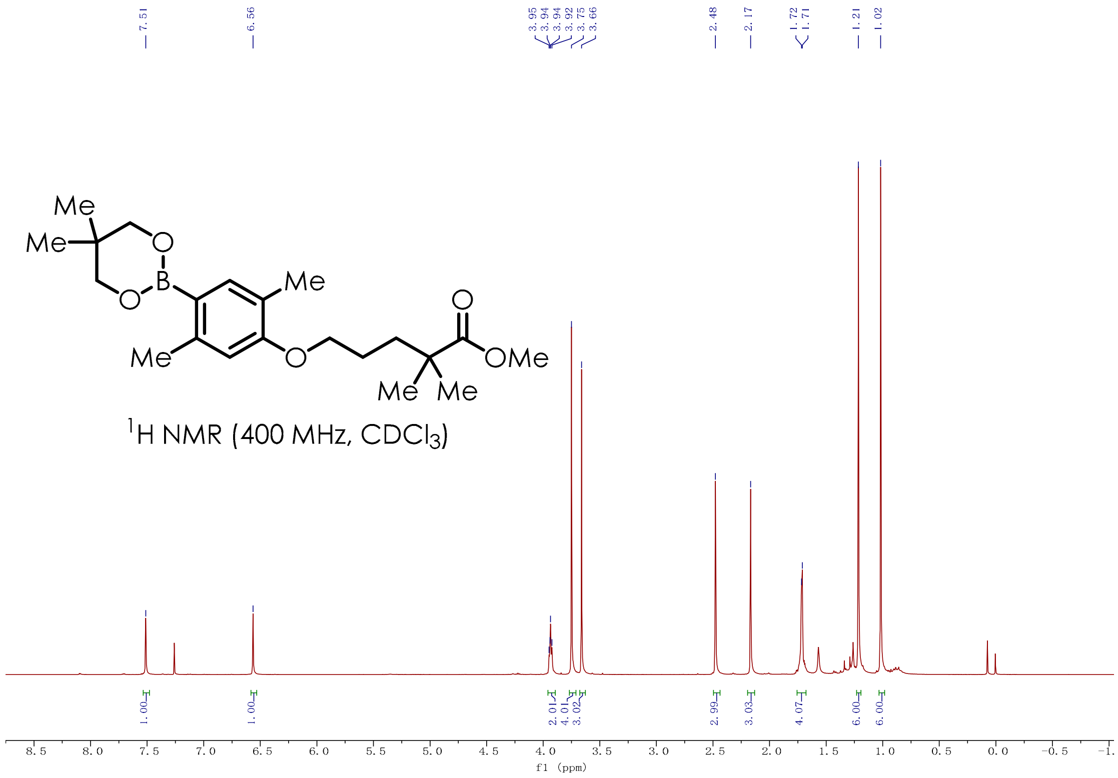
7.51
6.56
3.95
3.94
3.94
3.92
3.75
3.66
2.48
2.17
1.72
1.71
1.21
1.02
Me
Me
O
B
O
Me
Me
O
O
OMe
Me Me
1H NMR (400 MHz, CDCl3)
1.00
1.00
2.01
4.01
3.02
2.99
3.03
4.07
6.00
6.00
f1 (ppm)

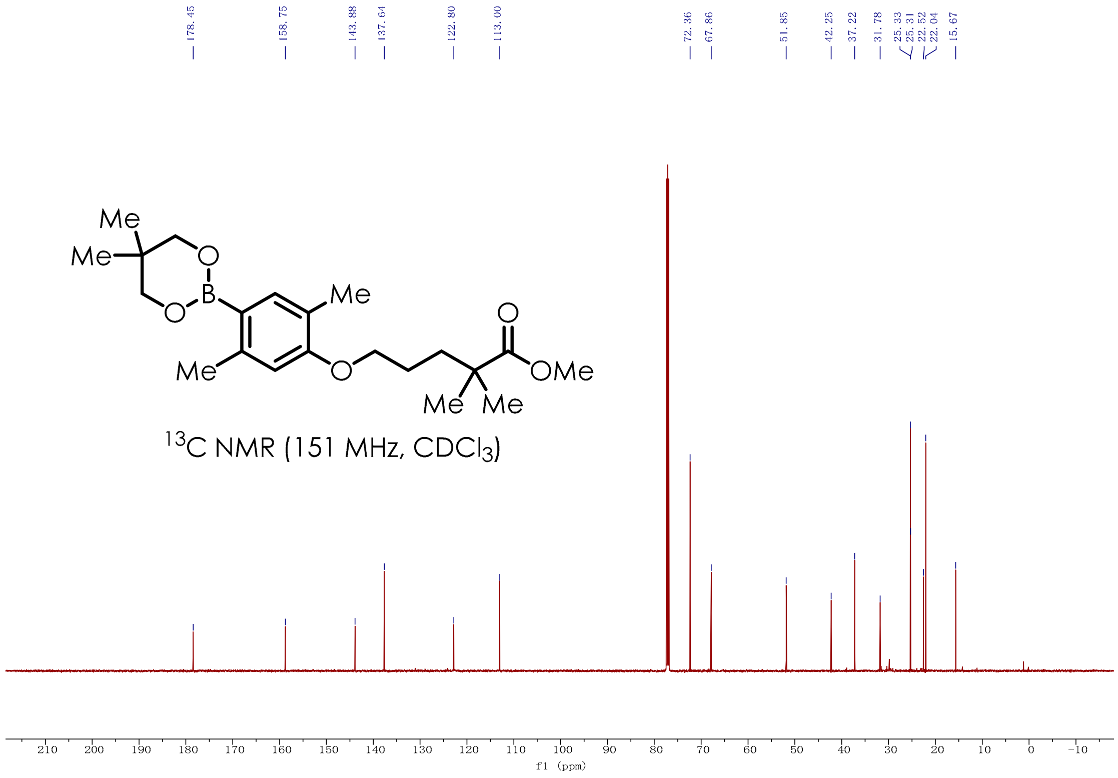
178.45
158.75
143.88
137.64
122.80
113.00
72.36
67.86
51.85
42.25
37.22
31.78
25.33
25.31
22.52
22.04
15.67
Me
Me
O
B
O
Me
Me
O
O
OMe
Me Me
13C NMR (151 MHz, CDCl3)
f1 (ppm)

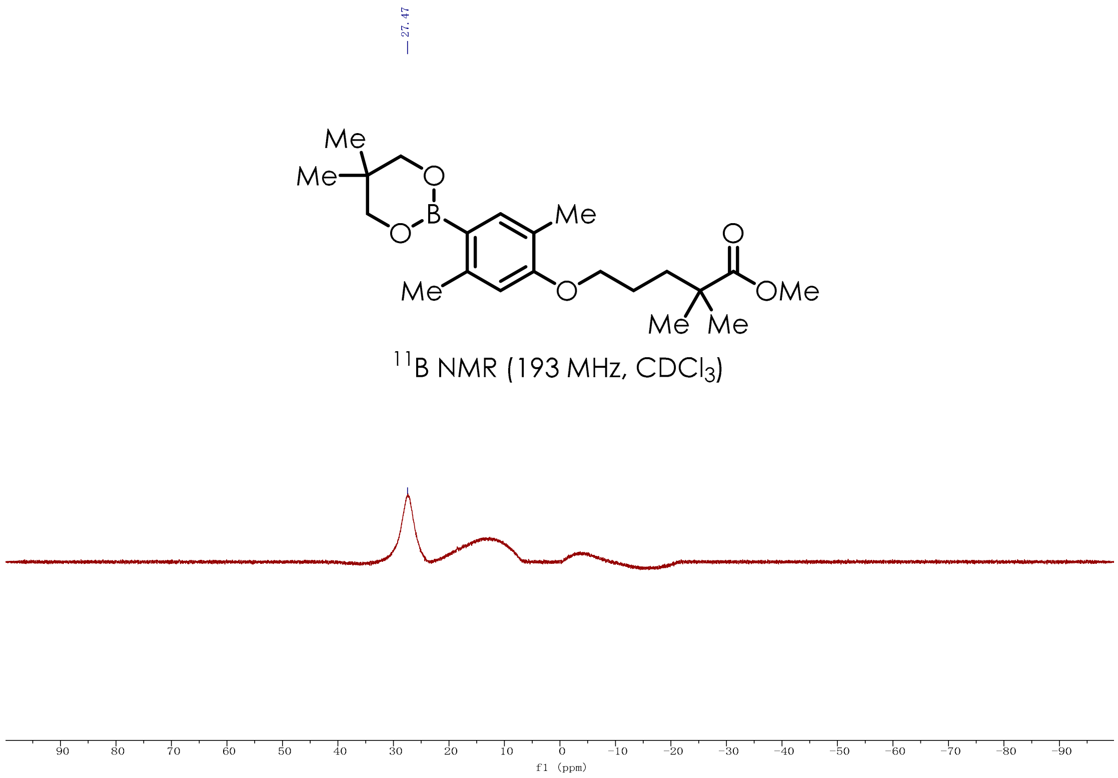

27.47
Me
Me
O
B
O
Me
Me
O
O
OMe
Me Me
^{11}B NMR (193 MHz, CDCl$_3$)
90 80 70 60 50 40 30 20 10 0 -10 -20 -30 -40 -50 -60 -70 -80 -90
f1 (ppm)

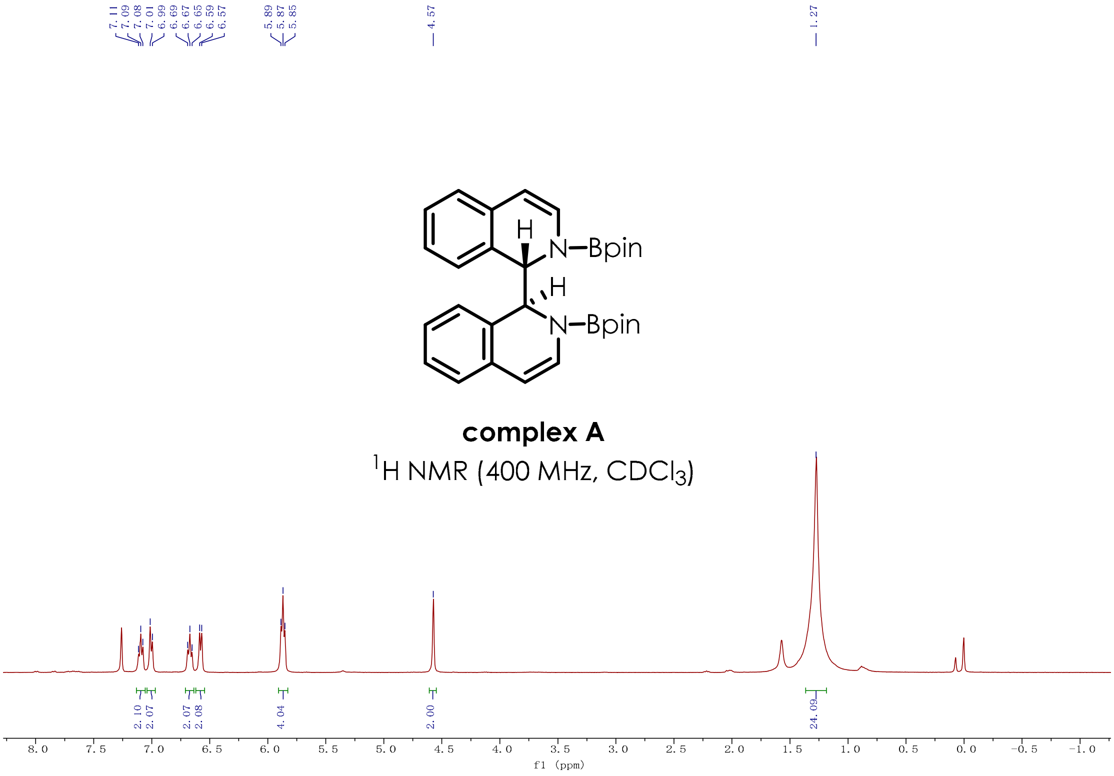

7.11 7.09 7.08 7.01 6.99 6.69 6.67 6.65 6.59 6.57
5.89 5.87 5.85
4.57
1.27
H
N–Bpin
H
N–Bpin
complex A
^{1}H NMR (400 MHz, CDCl$_3$)
2.10 2.07 2.07 2.08 4.04 2.00 24.09
8.0 7.5 7.0 6.5 6.0 5.5 5.0 4.5 4.0 3.5 3.0 2.5 2.0 1.5 1.0 0.5 0.0 -0.5 -1.0
f1 (ppm)

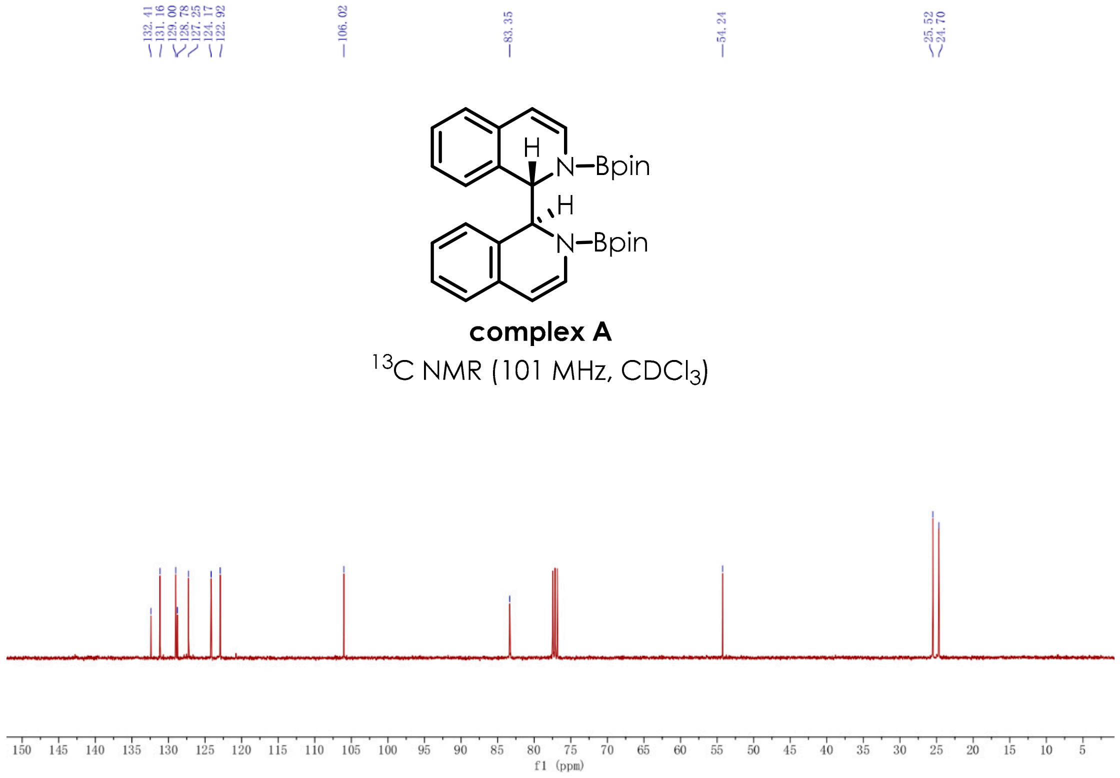
132.41
131.16
129.00
128.78
127.25
124.17
122.92
106.02
83.35
54.24
25.52
24.70
H
N–Bpin
H
N–Bpin
complex A
13C NMR (101 MHz, CDCl3)
150 145 140 135 130 125 120 115 110 105 100 95 90 85 80 75 70 65 60 55 50 45 40 35 30 25 20 15 10 5
f1 (ppm)

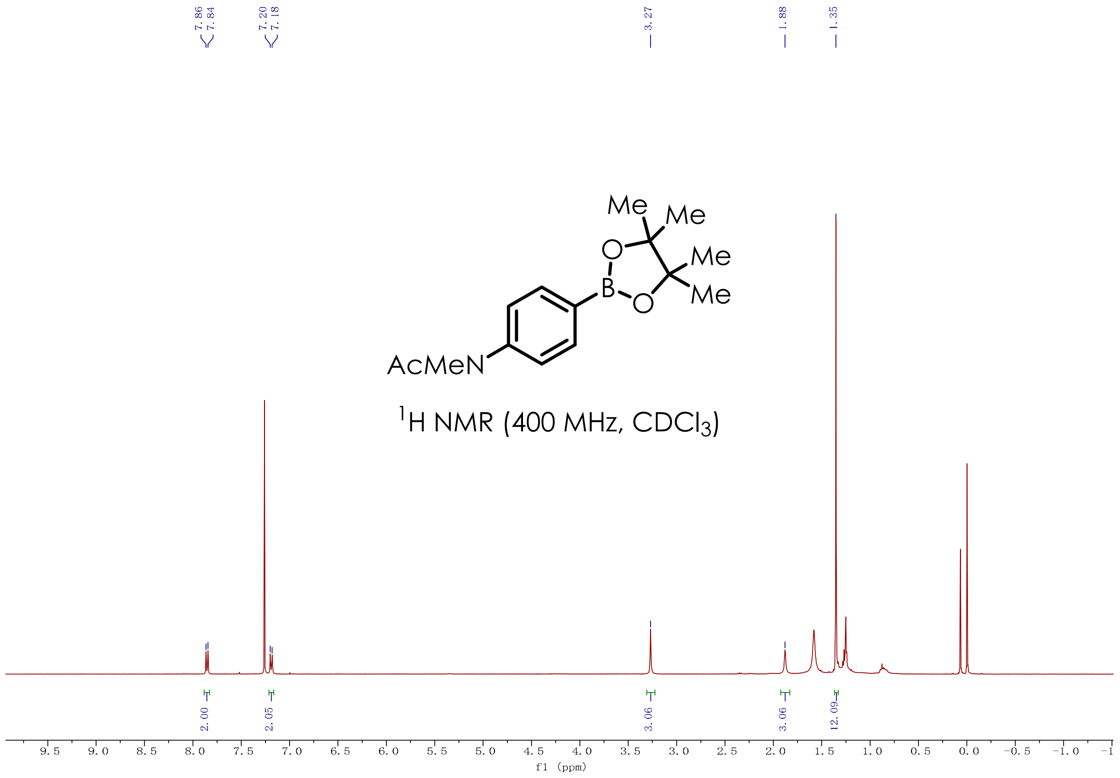
7.86
7.84
7.20
7.18
3.27
1.88
1.35
Me
Me
Me
Me
O
B
O
AcMeN
1H NMR (400 MHz, CDCl3)
2.00
2.05
3.06
3.06
12.09
9.5 9.0 8.5 8.0 7.5 7.0 6.5 6.0 5.5 5.0 4.5 4.0 3.5 3.0 2.5 2.0 1.5 1.0 0.5 0.0 -0.5 -1.0
f1 (ppm)
